# Transfer RNA Derived Small RNAs Targeting Defense Responsive Genes Are Induced during *Phytophthora capsici* Infection in Black Pepper (*Piper nigrum* L.)

**DOI:** 10.3389/fpls.2016.00767

**Published:** 2016-06-01

**Authors:** Srinivasan Asha, Eppurath V. Soniya

**Affiliations:** Plant Molecular Biology, Rajiv Gandhi Centre for BiotechnologyThiruvananthapuram, India

**Keywords:** black pepper, *Piper nigrum*, tRFs, tRNA derived small RNA, *Phytophthora capsici*, NPR1

## Abstract

Small RNAs derived from transfer RNAs were recently assigned as potential gene regulatory candidates for various stress responses in eukaryotes. In this study, we report on the cloning and identification of tRNA derived small RNAs from black pepper plants in response to the infection of the quick wilt pathogen, *Phytophthora capsici*. 5′tRFs cloned from black pepper were validated as highly expressed during *P. capsici* infection. A high-throughput systematic analysis of the small RNAome (sRNAome) revealed the predominance of 5′tRFs in the infected leaf and root. The abundance of 5′tRFs in the sRNAome and the defense responsive genes as their potential targets indicated their regulatory role during stress response in black pepper. The 5′Ala^CGC^ tRF mediated cleavage was experimentally mapped at the tRF binding sites on the mRNA targets of Non-expresser of pathogenesis related protein (NPR1), which was down-regulated during pathogen infection. Comparative sRNAome further demonstrated sequence conservation of 5′Ala tRFs across the angiosperm plant groups, and many important genes in the defense response were identified *in silico* as their potential targets. Our findings uncovered the diversity, differential expression and stress responsive functional role of tRNA-derived small RNAs during *Phytophthora* infection in black pepper.

## Introduction

Eukaryotic organisms respond to environmental stress with a specific gene expression programme at the mRNA and protein level. Also, small RNAs (sRNAs) are produced as modulators of gene expression during stress responses. They are short, non-coding RNAs of 16–35 nt long that regulate diverse biological functions by guiding sequence-specific gene silencing at the transcriptional or post-transcriptional level (Bartel, 2004; Vaucheret, 2006; Padmanabhan et al., 2009). Based on the unique biogenesis pathways, precursor structure, and mode of action, two major categories of sRNAs, including microRNAs (miRNAs) and small interfering RNAs (siRNAs), were identified in plants (Rajagopalan et al., 2006; Phillips et al., 2007; Axtell, 2013). The advances in high-throughput sequencing technologies had greatly accelerated the discovery of new types of small non-coding RNAs of diverse origin such as tRNAs (Cole et al., 2009; Loss-Morais et al., 2013; Hackenberg et al., 2015), snoRNAs (Chen and Wu, 2009; Taft et al., 2009; Li et al., 2012), and rRNAs (Wei et al., 2013). Despite the growing catalog of sRNAs, many new classes were under exploration. Although most of the sRNA types differed from one another, they were suggested to be interconnected in their mode of action (Morris and Mattick, 2014) and regulatory role in multitude of cellular processes in development, stress responses, metabolism, and maintenance of genome integrity of organisms (Ghildiyal and Zamore, 2009; Wan et al., 2014).

Host sRNAs and RNA silencing machinery was stated as the critical layer of defense during plant-pathogen interactions (Katiyar-Agarwal and Jin, 2010). Plants could respond to diverse strategies of pathogen infection by means of a two-branched innate immune system that recognizes and responds to the extracellular and intracellular pathogen signatures (Jones and Dangl, 2006; Yang and Huang, 2014). It was suggested that in the first phase, the transmembrane pattern recognition receptors of plant cell could respond to microbial or pathogens associated molecular patterns (MAMPs or PAMPs) and trigger the basal defense, known as PAMP Triggered Immunity (PTI) (Kwon et al., 2008; Luna et al., 2011). In the second phase, the successful pathogen could produce effectors that contribute to pathogen virulence. Subsequently, the plant might elicit a more rapid and robust immune defense response to counteract the effectors that were produced by successful pathogens. This effector triggered immunity (ETI) was reported to be mediated by the production of polymorphic NB-LRR proteins, that were encoded by *R* genes (Jones and Dangl, 2006; Li et al., 2010; Luna et al., 2011). The massive reprogramming of the transcriptome was triggered by both PTI and ETI (Navarro et al., 2006), and the post transcriptional events that shape this defense-related transcriptome were mostly comprised of functional sRNAs derived from a diverse set of non-coding RNAs. Small RNA pathways were modulated upon recognition of PAMPs or pathogen effectors to regulate intricate defense responses against pathogens (Ruiz-Ferrer and Voinnet, 2009; Seo et al., 2013). Plant miRNAs were reported to play vital roles in various developmental processes and stress responses (Sunkar and Zhu, 2004; Axtell and Bartel, 2005; Verma et al., 2014; Wu et al., 2014). The massive amounts of data generated from next generation sRNA sequencing revealed subclasses of miRNAs derived by alternate biosynthesis pathways (Miyoshi et al., 2010). These non-canonical miRNAs could partially meet the classical definition and were derived from the genomic loci containing repeat sequences (Xie et al., 2004), small nucleolar RNA (snoRNA) (Taft et al., 2009), transfer RNA (tRNA) (Lee et al., 2009), and ribosomal RNA (rRNA) (Chen et al., 2011; Wang et al., 2011; Wei et al., 2013). The sRNA fragments that were ~20–35 nt and derived from the 5′ and 3′ ends of tRNA were broadly termed as tRNA derived RNA fragments (tsRNA/tRFs; Li et al., 2008, 2012). The tRFs were reported as precisely generated functional sRNAs, evolutionarily conserved in all domains of life and associated with argonaute proteins (Kumar et al., 2014). The tRFs were generally classified into longer RNA species of 35 nt generated by the cleavage at the anticodon loop and microRNA-like (20 nt) fragments that were often produced by a cleavage in the D or T loops. The former category involved both 5′ and 3′ halves of mature tRNAs, while the latter involved the 5′ end of tRNAs and those generated from 3′ end of the mature tRNAs with a CCA sequence (3′ CCA tRFs) (Pederson, 2010; Sobala and Hutvagner, 2011). It was also proposed that there might be crosstalk between tRFs and the canonical sRNA pathways (Loss-Morais et al., 2013). In plants, the existence of tRFs was reported recently. The 5′tRFs of Asp^GTC^ tRNA and 5′ and 3′ CCA tRFs of Gly^TCC^ tRNAs were found to be over-expressed in the root tissues of *Arabidopsis thaliana* during phosphate deprivation (Hsieh et al., 2009). The differential expression of 5′Ala^AGC^ and Pro^CGG^ tRFs were demonstrated in the callus and leaves of *Oryza sativa* (Chen et al., 2011), while in barley, the His^GTG^tRF was identified as the most abundant of all the sRNAs (Hackenberg et al., 2013). The possible association of tRFs with the AGO proteins and their contribution in the RNAi pathway during the biotic and abiotic stress response was identified in *A. thaliana* (Loss-Morais et al., 2013). The up-regulation of tRF during stress conditions such as drought (Hackenberg et al., 2015) and pathogen infection (Visser et al., 2014) was also reported in plants.

Black pepper (*Piper nigrum* L.) is the most extensively cultivated economic spice crop from the family Piperaceae. The spicy berries of this perennial vine are an unavoidable ingredient in global cuisines. Foot rot, also known as quick wilt, is the devastating disease of black pepper caused by the oomycete *Phytophthora capsici* and affects plant growth at any stage, resulting in a drastic reduction of plant population. The pathogen also infects a broad range of perennials as well as vegetable crops, and its broad host range, long-lived dormant sexual spores, extensive genetic diversity, and explosive asexual disease cycle makes it a worst-case scenario to farmers (Lamour et al., 2012). A critical step toward understanding the molecular basis of pathogenicity and in developing improved disease management strategies to safeguard food production from *P. capsici* diseases is to study the host pathogen interactions. Endogenous sRNA-mediated gene silencing could be a general regulatory mechanism of the plant's immune response to many pathogens. Previous studies identified microsatellite derived miRNAs (Joy and Soniya, 2012) and conserved miRNAs in black pepper (Asha et al., 2013). The *de novo* transcriptome of the black pepper leaf also indicated the discovery of microsatellite-associated miRNA candidates from black pepper (Joy et al., 2013). To elucidate the role of tRNA-derived sRNAs and their functional role in stress regulation during *P. capsici* infection, we employed a systematic analysis of deep sequenced black pepper sRNA datasets.

## Materials and methods

### Plant materials and stress treatment

The black pepper variety Panniyur-1 was grown in a greenhouse at the Rajiv Gandhi Centre for Biotechnology, Thiruvananthapuram, Kerala, India. The rooted cuttings of black pepper was planted in earthen pots (30 cm diameter) filled with potting media (soil, sand, and cow manure 1:1:1) during May–June, and transferred to naturally ventilated greenhouse, with natural lighting, temperature (27 ± 2°C), and humidity conditions (75–80%). After 3 months of growth, the plants were inoculated with the pathogen. For this, the virulent culture of *P. capsici* was obtained from the Department of Plant Pathology, College of Agriculture, Thiruvananthapuram and maintained by continuous sub-culture on potato dextrose agar (PDA) medium. For pathogen induction, *P. capsici* was cultured in PDA medium at 28°C for 48 h and 10 mm mycelium discs were used. The fully expanded young leaves and collar region of three plants were inoculated. The mycelia discs were placed on the abaxial side of the leaves and the collar region of plants, after giving mild pinpricks and incubated for 24 h. Moist cotton was placed over the disc and the plants were covered with polythene bags to ensure high humidity, necessary for pathogen infection. The uninfected systemic upper leaves and roots were harvested from pathogen-infected plants at 24 h after inoculation.

### Microscopic studies to identify the fungal infection in black pepper

*P. capsici* infected black pepper leaves were microscopically checked by trypan blue staining (Heese et al., 2007). The infected and control leaves were boiled in lacto phenol trypan blue stain (6 vol of ethanol, 1 vol of water, 1 vol of lactic acid, 1 vol of glycerol, 1 vol of phenol, 0.067% wt/vol trypan blue) for 2 min. After cooling for 1 h at room temperature, the trypan blue was replaced with the chloral hydrate solution and the leaves were incubated overnight at 28°C. The lesions were visualized and size of the lesions were analyzed. The pathogen infected necrotic sections of the whole leaf was captured from the Leica EZ4 D digital stereomicroscope (Leica, Wetzlar, Germany). The pathogen structures were further analyzed from the thin sections of the infected parts, at 20x and 40x magnification in a fluorescence microscope (Nikon Eclipse E600) and the images were captured.

### Small RNA library construction and sequence analysis

Small RNA (≤ 200 nt) was isolated from the leaf and roots of pathogen infected *P. nigrum* plants using mirVana miRNA isolation kit (Ambion), according to the manufacturer's instructions. The sRNA samples with OD260/OD280 ~2.0 were used for the cloning. The overall work flow for the small RNA cloning was shown in Figure S1. In brief, sRNAs were separated using flash PAGE (Applied Biosystems), and size fractions corresponding to ≤ 45 nt were recovered and purified. The sRNA fractions were dephosphorylated to remove the 5′PO_4_, purified and ethanol precipitated. Dephosphorylated RNA was ligated with 3′ adapter:5′pCTGTAACTCGGGTCAATddC3′ (Abnova kit). The ligation products were recovered on a 20% polyacrylamide gel, 5′phosphorylated, and purified. Reverse transcription was performed using SMART technology (Clontech), which relies on a 3′ ligated sRNA template to switch to 5′SMART IV oligo (5′AAGCAGTGGTATCAACGCAGAGTGGCCATTACGGCCG GG3′). In brief, 7 μL phosphorylated 3′ linker ligated RNA, 2.5 μL SMART IV oligo (25 μM), and 0.5 μL 3′RT primer (5′ATTGACCCGAGTTACAG3′) were mixed together and incubated for 20 min and then cooled for 2 min. To this, 4 μL first strand buffer (5x), 2 μL dTT (20 mM), 2 μL dNTP (10 mM), and 2 μLSMARTScribe™ Reverse Transcriptase (Clontech, Mountain View, CA, USA) were added and incubated at 42°C for 60 min, followed by 70°C for 15 min. Subsequent PCR amplification was carried out in 25 μL reaction volume using adapter specific primers: FP 5′AAGCAGTGGTATCAACGCAGAGT3′ and RP: 5′ATTGACCCGAGTTACAG3′. The reaction consisted of 2.5 μL 10x Advantage^®^2 polymerase buffer, 0.5 μL Advantage^®^2 polymerase mix (Clontech), 0.5 μL each forward and reverse primer (10 μM), 0.5 μL first strand cDNA template, 0.5 μL dNTP (50x), and 20 μL nuclease free water. The thermal profile followed for amplification was: initial denaturation at 95°C:1 min, 26 cycles of denaturation at 95°C:10 s, primer annealing at 50°C:60 s, primer extension at 72°C:20 s; final extension at 72°C:10 min. The amplified products were analyzed by visualization in 2% agarose gel. The PCR amplicons of 75–90 nt size were purified using GFX™ PCR DNA and Gel Band Purification Kit (GE Healthcare, USA) and cloned into pGEM^®^-T Easy vector (50 ng) (pGEM^®^-T Easy kit, Promega). In brief, the 10 μL reaction volume of ligation was prepared with 1 μL pGEM^®^-T Easy vector, 3 μL of the purified PCR product, 5 μL Rapid ligation buffer (2x) and 1 μL T4 DNA ligase (3 U/μL), and incubated overnight at 4°C. Followed by the transformation of recombinant pGEM^®^-T plasmids to chemically competent *Escherichia coli* strain JM 109, white colonies were selected from LB Agar+X-gal/IPTG plates. To identify the insert sequences, plasmid DNA was isolated from the positive clones by alkali lysis method (Birnboim and Doly, 1979) and sequenced using BigDye Terminator v3.1 Cycle Sequencing Kit (Applied Biosystems) with vector specific T7 (5′ TAATACGACTCACTATAGGG3′) and SP6 primers (5′ TATTTAGGTGACACTATAG3′) in ABI PRISM^®^ 3700 DNA Analyzer (ABI). The thermal profile consisted of initial denaturation at 96°C for 2 min; 25 cycles of denaturation at 96°C for 30 s, primer annealing at 50°C for 30 s and primer extension at 60°C for 4 min. The read data were analyzed using Sequence scanner v.1.0 software (Applied Biosystems) and sequences were extracted. After manually trimming the adapter sequences, the small RNAs in the size range of 15–30 nt were selected and categorized to different annotation classes by BLASTn search in the different RNA databases such as miRBase (www.mirbase.org/), genomic tRNA database (http://gtrnadb.ucsc.edu/), plant tRNA database (http://plantrna.ibmp.cnrs.fr/), and fRNADb (http://www.ncrna.org/).

### Real time qRT-PCR expression analysis and validation of small RNAs

Small RNA validation and expression profiling was performed by stem-loop RT-PCR (Varkonyi-Gasic et al., 2007). In brief, sRNA enriched total RNA was isolated from the leaf (Pn_IL) and root (Pn_IR) tissues of *P. capsici* infected black pepper plants using the mirVana miRNA isolation kit (Ambion) as per the manufacturer's instructions. The RNA integrity was checked from 1% agarose gel by ethidium bromide staining. The quantity and quality of the samples were further assessed from the Nano Drop™1000 spectrophotometer (Thermo Scientific, Wilmington, DE) and samples with OD260/280 absorption ratio ~2.0 were used for the cDNA synthesis. Uninfected, mock inoculated plants were used as the control. First-strand cDNA synthesis was conducted by one-tube Stem-loop RT-PCR (Shen et al., 2010). Each 15 μL reaction contained 1 μg of total RNA, 0.5 μL each of the appropriate stem-loop primers (10 μM), 0.15 μL of the dNTP mix (100 mM), 1 μL MultiScribe™ MuLV RT enzyme (50 U/μL) (TaqMan microRNA reverse transcription kit, ABI), 1.5 μL Reverse Transcription buffer (10x), 0.19 μL RNase inhibitor (20 U/μL), and nuclease free water. The quantitative Real-Time PCR was performed with the Applied Biosystems7900 HT Fast Real-Time system using the Power SYBR Green qPCR Master Mix (ABI, Life Technologies, USA). Three technical replicates were performed for each sRNA candidate. 5.8S rRNA was used as the endogenous reference. The relative expression was calculated by the Pfaffl method (Pfaffl, 2001), that took into account the PCR efficiency and CT values. The sequences of primers used for the Stem-loop RT-PCR, and PCR amplification efficiency (Bustin et al., 2009) of each TRF specific primers calculated by means of calibration curves were presented in Table S1. PCR specificity was examined by melting curve analysis from 65 to 95°C, and the products were checked in a 3% agarose gel. The tRF amplification was further confirmed by the stem-loop end-point RT-PCR (Varkonyi-Gasic et al., 2007) using tRF specific forward primer and universal reverse primer, at 94°C for 2 min, followed by 25 cycles of 94°C for 15 s, 60°C for 1 min. The amplicons of ~60 bp detected from the agarose gel were cloned topGEM^®^-T Easy Vector (Promega) and sequenced using T7 and SP6 primers as mentioned in 2.3.

### Characterization of tRFs from high-throughput sRNA data of black pepper

The recently sequenced sRNA transcriptome of pathogen stressed black pepper plants were analyzed for the presence of tRFs. The sRNA reads of the black pepper control leaf (PnCL) (Asha et al., 2016), pathogen stressed leaf (PnIL) and root (PnIR) libraries (manuscript under preparation) were first aligned to the tRNA sequences from Rfam database, and tRFs were extracted. The tRFs were further analyzed to identify their length, distribution pattern and abundance. 5′tRFs with ≤ 100 reads were selected, and their relative cloning frequency from each sRNA library was calculated (Wang Q. et al., 2013). The sequence data supporting the results is available in the NCBI GEO repository with the accession numbers GSM1606153-GSM1606156. Microsoft Excel was used to generate the bar diagrams. The read count of each tRF variant was represented as the normalized read count from the corresponding small RNA library.

### Prediction of tRF targets and validation of target cleavage

The high complementarity of the plant sRNA with its target genes allowed their computational prediction. The putative targets of tRFs were *in silico* predicted from black pepper transcriptome sequences (NCBI Sequence Read Archive under accession no. SRX853366 and SRX856639) using psRNATarget (plantgrn.noble.org/psRNATarget; Dai and Zhao, 2011). To determine whether this sRNA mediates the cleavage of target messenger RNAs (mRNAs), a modified 5′RLM RACE (RNA Ligase mediated Rapid Amplification of cDNA Ends) experiment was carried out (Asha et al., 2013). In brief, the GeneRacer™RNA oligoadapter (5′CGACUGGAGCACGAGGACACUGACAUGGACUGAAGG AGUAGAAA3′) (Gene racer kit, Invitrogen) was ligated to total RNA without an alkaline phosphatase treatment. Then the ligated RNAs were reverse transcribed using GeneRacer™oligodT primer and SuperScript™ III Reverse Transcriptase (Gene racer kit, Invitrogen). The 20 μL reaction mixture consisted of 10 μL of ligated RNA sample, 5 μL First Strand Buffer (5x), 1 μL DTT (0.1 M), 1 μL RNaseOut™ (40 U/μl), 1 μL of SuperScript™ III RT (200 U/μl), 1 μL primer, and 1 μL dNTP mix. Followed by the incubation at 50°C for 60 min, the RT reaction was inactivated at 70°C for 15 min. Then 1 μL of RNase H (2 U) to the reaction mix and incubated at 37°C for 20 min. The PCR amplification of the cDNA ends were performed using a forward primer specific to the 5′adapter sequences:5′CGACTGGAGCACGAGGACACTGA3′ and gene-specific reverse primers: NPR1 RP 5′GTCTCAGCCAGT GGATTTCTTC3′ and E3ULRP 5′ACAGCCGA CTTAGTGATACCTC3′ at a thermal profile: 94°C/2 min (1 cycle); 94°C/30 s, 72°C/1 min (5 cycles); 94°C/30 s, 70°C/1 min (5 cycles); 94°C/30 s, 65°C/30 s, 72°C/1 min (25 cycles); 72°C/10 min (1 cycle). A separate reaction mixture of 50 μL was set up for each of the target genes, and consisted of 4.5 μL GeneRacer™ 5′ Primer (10 μM), 1.5 μL Gene Specific Reverse Primer (10 μM), 1 μL RT template, 0.125 μL AmpliTaq Gold^®^ DNA Polymerase (5 U/μL) (ABI, CA)1.5 μL dNTP mix (10 mM), and 5 μL PCR buffer (10x). The amplified products were purified (Gel Band Purification Kit, GE Healthcare, USA), cloned into a PCR™4 –TOPO vector (TOPO^®^ TA CloningKit for Sequencing, Invitrogen, USA) and transformed into competent *E. coli* strain DH5-Alpha. The plasmid were isolated from the positive clones using alkaline lysis method (Birnboim and Doly, 1979) and sequenced with BigDye Terminator v3.1 Cycle Sequencing Kit (Applied Biosystems), using M13 forward primer (5′GTAAAACGACGGCCAG-3′) and M13 reverse primer (5′CAGGAAACAGCTATGAC3′) with the cycling parameter such as initial denaturation at 96°C for 2 min followed by 25 cycles of denaturation at 96°C for 30 s, primer annealing at 50°C for 30 s and primer extension at 60°C for 4 min.

### Quantitative real time analysis of NPR1 mRNAs

Total RNA was isolated from the leaf and root tissues of control uninfected plants and *P. capsici* infected black pepper plants as mentioned in 2.4. The quality and quantity of RNA was checked from Nano Drop™1000 spectrophotometer (Thermo Scientific, Wilmington, DE) and samples with OD260/280 reading ~2.0 was used for the analysis of gene expression. The first strand cDNA was synthesized from 2 μg of RNA using a high capacity cDNA reverse transcription kit (Applied Biosystems, Life Technologies, USA). In brief, the reverse transcription reactions were carried out in a total volume of 10 μL containing 2 μg RNA, 2 μL random primers (10x), 1 μL MultiScribe reverse transcriptase (50 U/μL), 2 μL RTbuffer (10x), 0.8 μL dNTPmix (25x), and 1 μL RNase inhibitor. Real time primers were designed by Primer3 software (http://primer3.ut.ee/) and the primers used for the NPR1 gene were as follows: NPR1 FP, 5′GAGGTTGACAAGGGCAAAGGA3′ and NPR1 RP, 5′GTCTCAGCCAGTGGATTTCTTC3′. For quantitative PCR analysis, the cDNAs were diluted and combined with the Power SYBR Green qPCR Master Mix (ABI, Life Technologies, USA). PCR reactions were performed in triplicate using Applied Biosystems7900 HT Fast Real-Time system. The quantitative PCR results were analyzed using 5.8SrRNA as the endogenous control.

### Analysis of sequence conservation and targets of 5′Ala tRFs from different plant species

The Alanine-tRNA sequences of different plant species were accessed from the plant tRNA database and genomic tRNA database. The high-throughput leaf sRNA libraries of plant species such as *A. thaliana* (GSM707679), *Solanum tuberosum* (GSM803582*), Populus trichocarpa* (GSM717875), *Medicago truncatula* (GSM769277), *Zea mays* (GSM433620), *Sorghum bicolor* (GSM803128), and *O. sativa* (GSM361264) were accessed from the NCBI GEO database, and the sequence diversity of 5′Ala tRFs were analyzed. The target sequences of 5′Ala^CGC^tRFs were further predicted from different plant species using psRNATarget (plantgrn.noble.org/psRNATarget).

## Results

### Preliminary cloning and computational characterization of small RNAs from *P. capsici* infected black pepper

**Figure 1:**
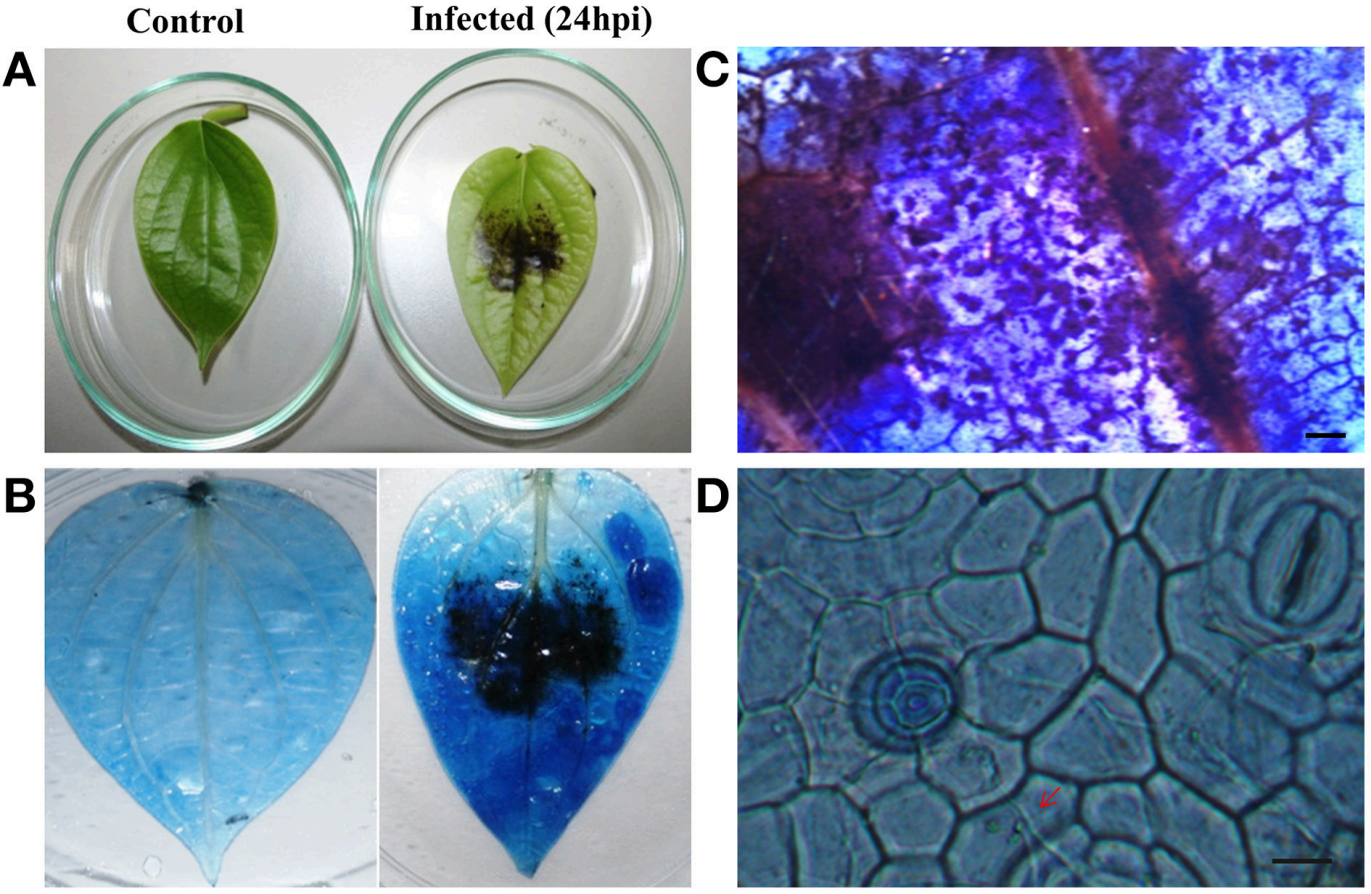
**Disease symptoms of *P. capsici* infection in black pepper leaves**. **(A)** Water soaked lesions of cell death observed in the infected leaf. Pathogen-induced cell death was visualized by trypan blue staining in the **(B)** whole leaf and **(C)** leaf sections (5X). Pictures were taken 24 h post-inoculation. **(D)** Fungal hyphae emerging through stomata pores and penetrating the cells (40X). Scale bars in **(C)** represent 1 mm and **(D)** represents 100 μM. Red arrow points the fungal hyphae penetrating the cells.

**Figure 2:**
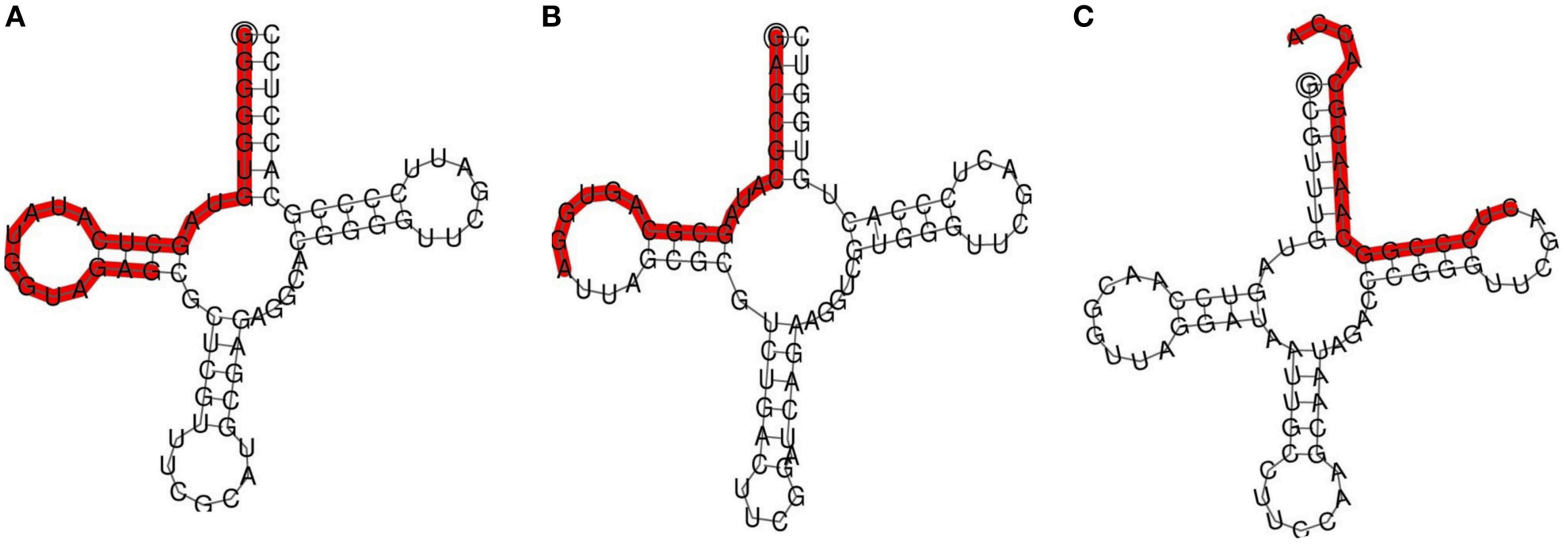
**TRNA derived small RNAs cloned from black pepper: (A) 5′Ala^CGC^ tRF and (B) 5′Arg^TCG^ tRF, and (C) 3′Gly^TCC^ tRF**. TRFs were highlighted on the secondary structures of the mature tRNA.

To explore the role of sRNAs produced in response to *P. capsici* infection in black pepper, we generated sRNA libraries from pathogen-infected plants. Typical water soaked lesions with a mean diameter of 10 mm were observed in black pepper leaves at 24 hpi of *P. capsici* (Figure 1A). *P. capsici* infection induced hypersensitive cell death responses in leaves (Figure 1B), and progression of the pathogen infection was studied by trypan blue staining (Figures 1C,D). To avoid the presence of oomycete RNA in black pepper leaf samples, the uninfected systemic leaves were used for small RNA isolation. Compared with the root tissues (1.4 μg/μL), a high concentration of sRNA fractions (≤ 200 nt) was obtained from the leaf (3.4 μg/μL). Four hundred clones were randomly selected and sequenced from the leaf and root sRNA libraries of *Phytophthora* infected black pepper plants. A total of 105 sequences, constituting ~26.5% of the total sequenced clones, were within the size range of 17–27 nt. The final 40 non-redundant sequences were categorized to different types of non-protein coding RNAs (ncRNAs) (Table S2). By further analysis from genomic tRNA database and tRNAdb, two sRNAs of 23 and 19 nucleotide length were identified as the 5′tRFs of Ala-tRNA^CGC^ and Arg-tRNA^TCG^, respectively (Figures 2A,B), while one sRNA candidate was identified as 3′tRF cleaved from the 3′end of Gly-tRNA^TCC^ with a typical 3′CCA end (Table 1) (Figure 2C).

**Table 1:** **Transfer RNA derived small RNAs cloned from *P. capsici* infected black pepper**.

**Seq Id**	**Sequences**	**Sequence length**	**Annotation**
Pn_TRF1	GGGGGUGUAGCUCAUAUGGUAGA	23	5′TRF (Ala-tRNA^CGC^)
Pn_TRF2	GACCGCAUAGCGCAGUGGA	19	5′TRF (Arg-tRNA^TCG^)
Pn_TRF3	CUCCCGGCAAACGCACCA	18	3′TRF (Gly-tRNA^TCC^)

### Real-time qRT-PCR expression studies of tRFs

**Figure 3:**
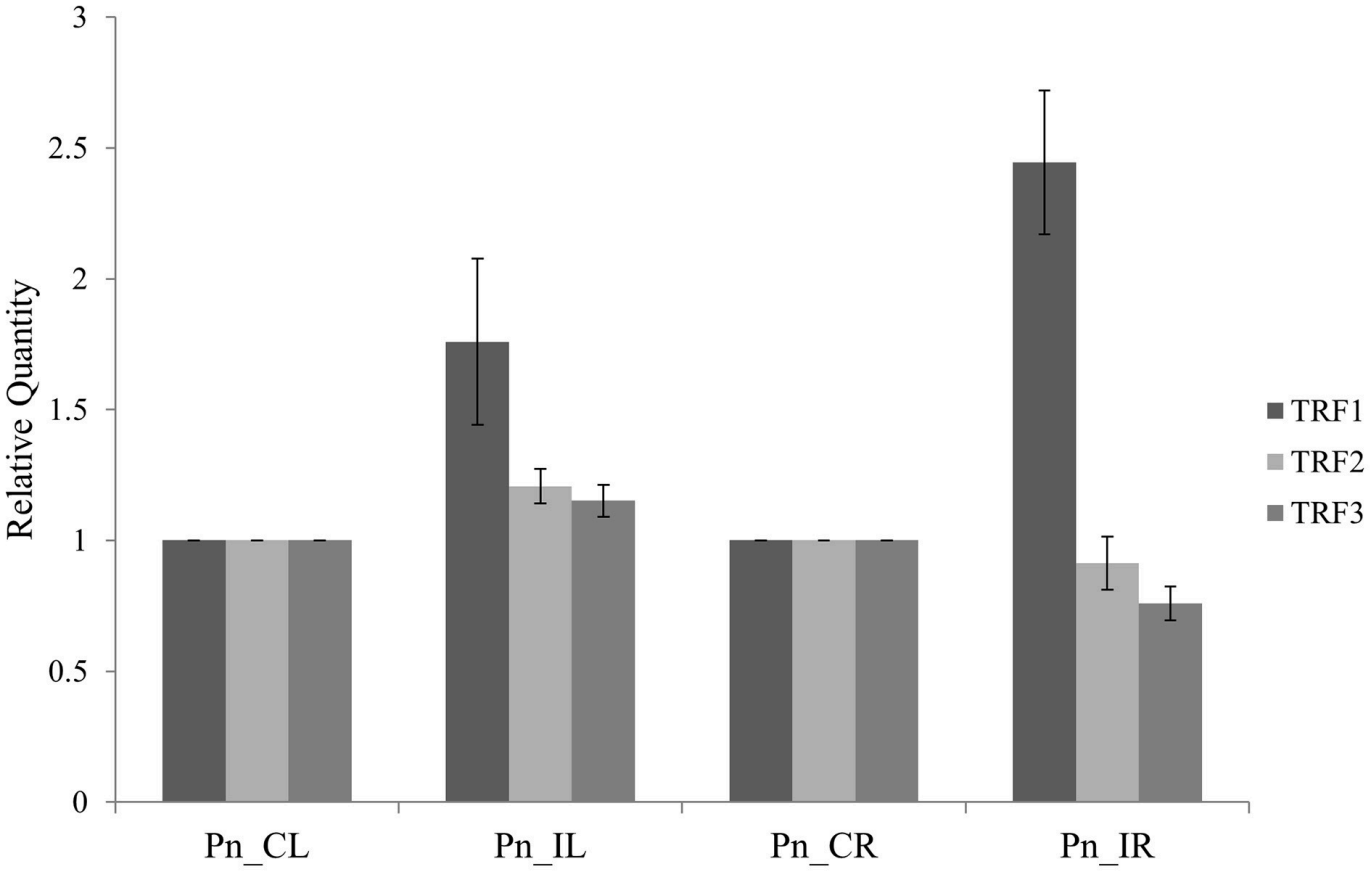
**The expression analysis of tRNA derived small RNAs using Stem-Loop Quantitative Real-Time RT-PCR: TRF1 (5′Ala^CGC^ tRF) and TRF2 (5′Arg^TCG^ tRF) and TRF3 (3′Gly^TCC^ tRF)**. Pn_IL, Pathogen infected leaf; Pn_IR, Pathogen infected root; Pn_CL, Control Leaf; Pn_CR, Control Root. All samples were normalized with 5.8S rRNA as the endogenous control and expression was compared with that of leaf and root of control uninfected plants. Three technical replicates were analyzed and mean SD was represented as the error bars.

The three tRFs, 5′Ala-tRNA^CGC^, 5′Arg-tRNA^TCG^, and 5′Gly-tRNA^TCC^ were confirmed by stem-loop RT PCR. The PCR product of ~60 bp, occurrence of single peak in the qRT-PCR dissociation curves (Figure S2) and sequences of the cloned PCR amplicons further validated the real time primers for tRFs. The relative gene expression from the stem loop qRT-PCR as calculated by the Pfaffl method (Pfaffl, 2001) showed higher expression of all the three tRFs in the leaf of *P. capsici* infected plant (Pn_IL) compared to the uninfected leaf of mock inoculated, control plants (Pn_CL) (Figure 3). In infected root (Pn_IR) also, the expression of 5′Ala^CGC^tRF was up-regulated, while 5′Arg^TCG^tRF and 5′Gly^TCC^tRF were down-regulated compared to the control uninfected root (Pn_CR).

### Analysis of tRF repertoire from deep sequenced sRNA libraries of black pepper

**Figure 4:**
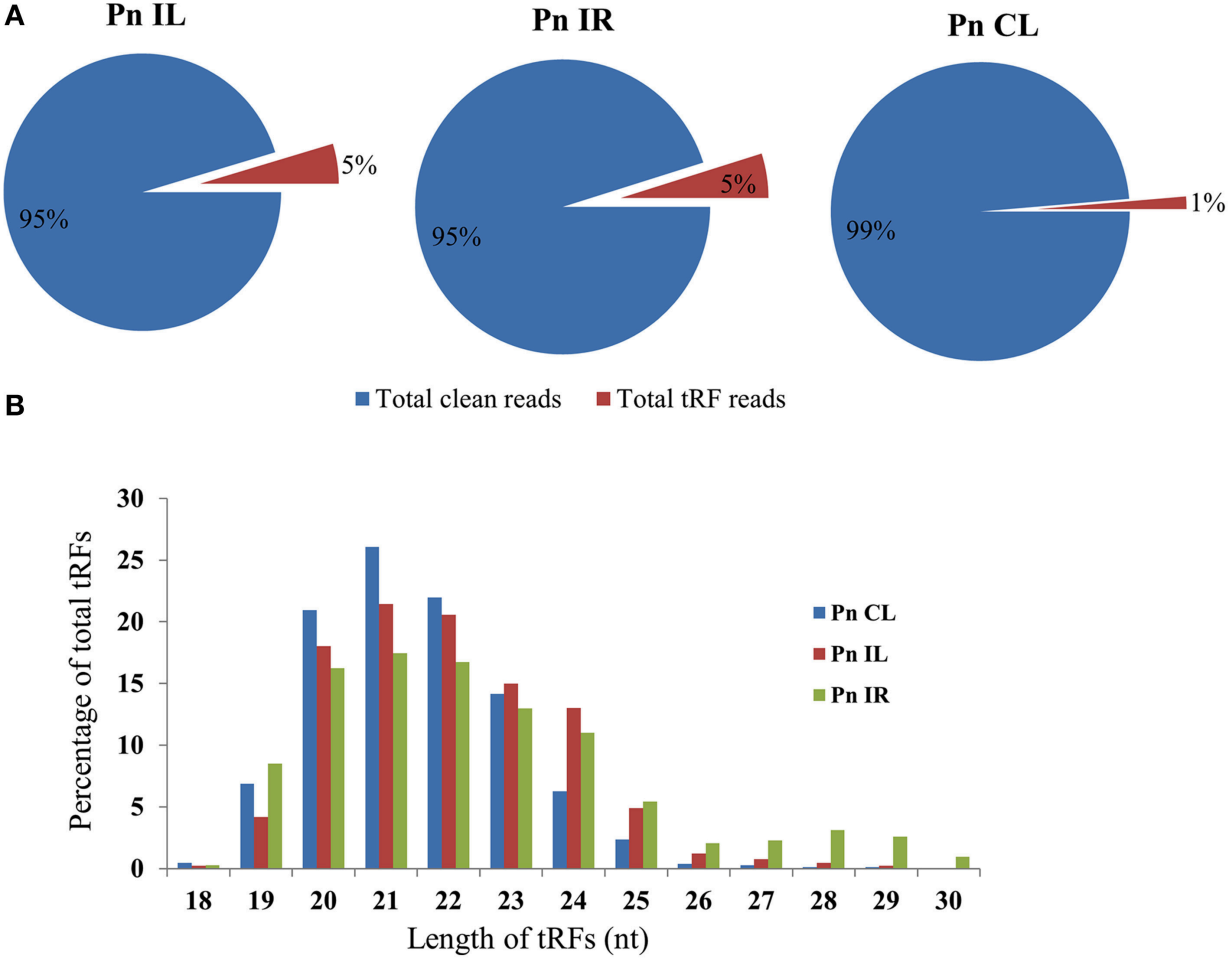
**(A)** Proportion of tRFs in the deep sequenced sRNA datasets of Pn CL, Pn IL, and Pn IR of black pepper. The tRF reads were represented as the percentage of total reads from each library. **(B)** Size distribution of small RNA reads mapped to tRNAs in Pn CL, Pn IL, and Pn IR sRNA libraries. The frequency of each tRF length variants were represented as percentage of total tRF reads in the sRNA libraries of Pn CL, Pn IL, and Pn IR.

**Figure 5:**
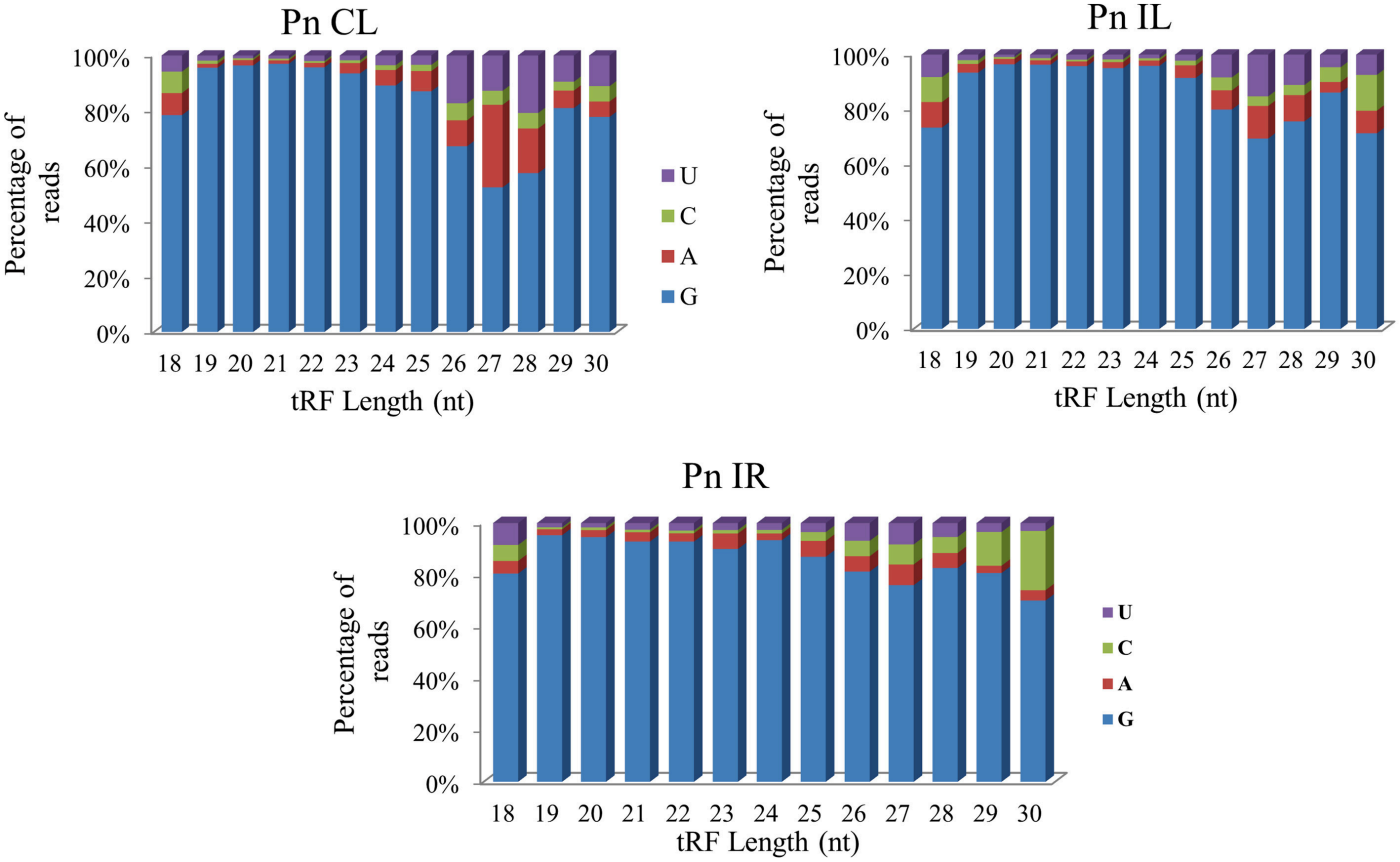
**The 5′terminal nucleotide base identity of tRNA derived small RNAs from Pn CL, Pn IL, and Pn IR sRNA libraries**. G was detected as the prominent 5′nucleotide in all the length groups. The frequency of each terminal nucleotide was plotted as the percentage of the total tRF reads of each length group.

**Figure 6:**
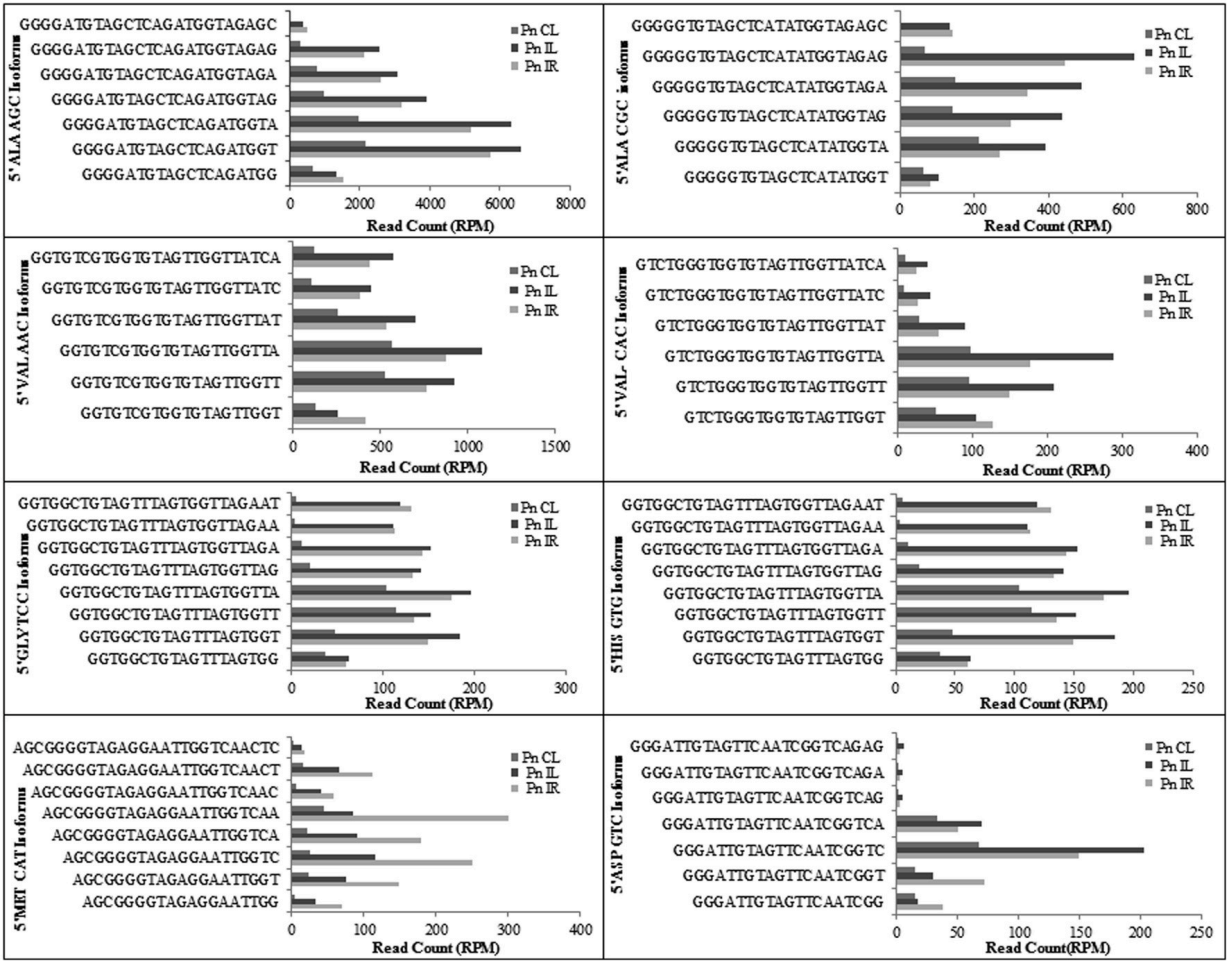
**The frequency of most prominent 5′tRF length variants of different tRNAs from black pepper sRNA libraries**. The normalized read counts (RPM) were represented.

**Figure 7:**
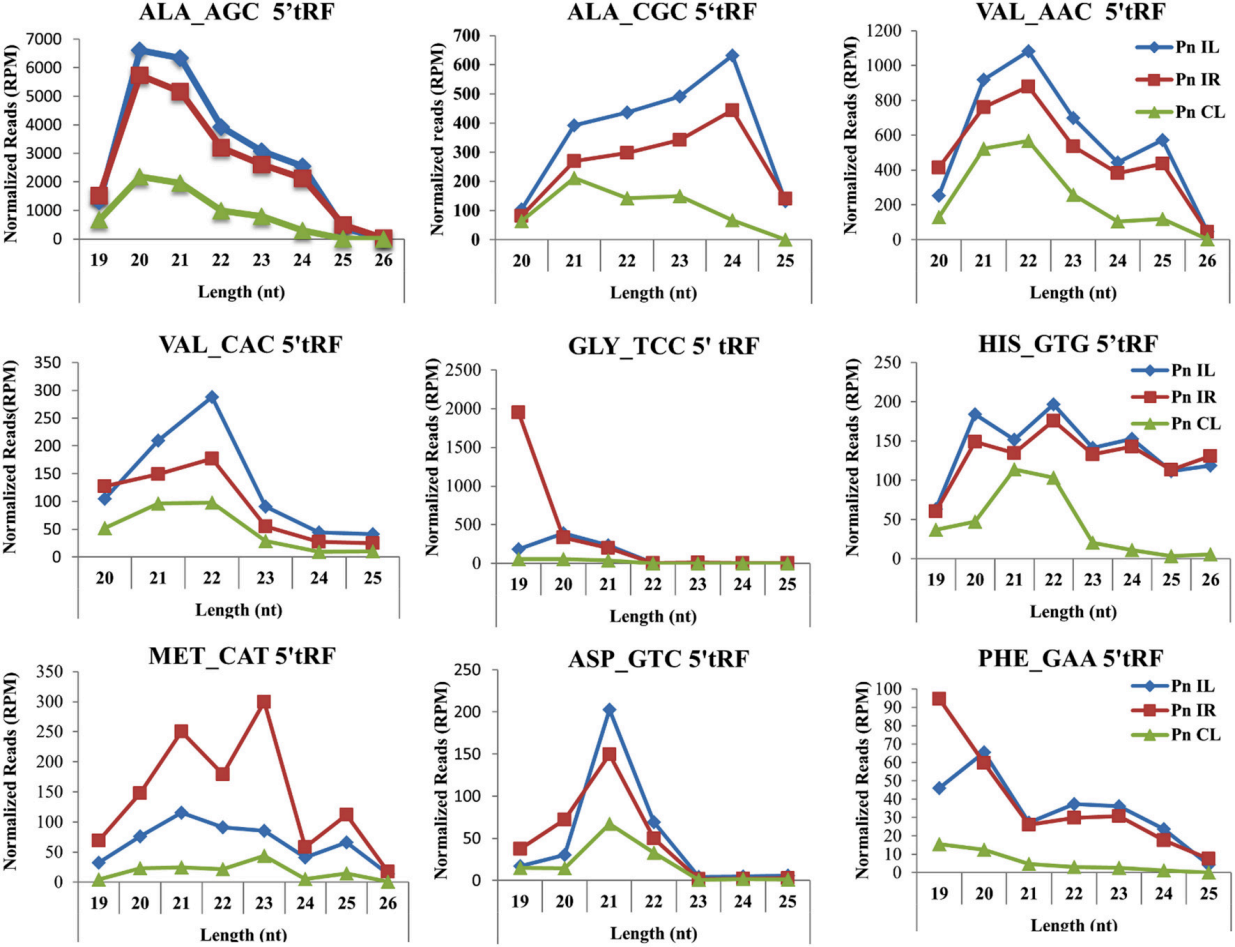
**Frequency of length variants of 5′tRFs of diverse tRNAs identified from Pn CL, Pn IL, and Pn IR sRNA libraries**. The normalized read count of each 5′tRF length variants in Reads per Million (RPM) was represented. The spread of data was represented as standard deviation of the RPM values for each length group in the three libraries.

**Figure 8:**
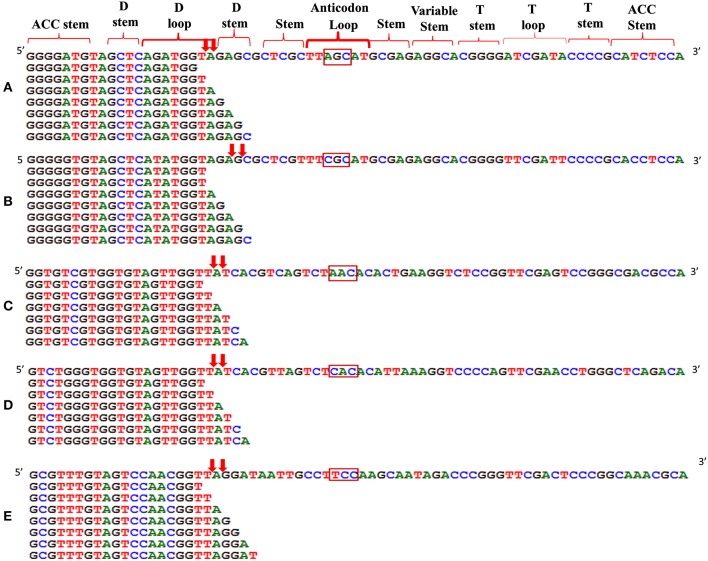
**Alignment of 5′tRF isoforms to the mature tRNAs of (A) Ala-tRNA^AGC^, (B) Ala-tRNA^CGC^, (C) Val-tRNA^AAC^, (D) Val-tRNA^CAC^, and (E) Gly-tRNA^TCC^**. The cleavage sites were designated as downward arrows. The anticodon of each tRNAs was represented in the rectangle. The cleavage occurs at the D stem/loop region in the tRNAs.

We analyzed a deep sequenced sRNA dataset of black pepper to systematically analyse tRFs. A total of 18.63, 12.62, and 12.95 million reads were generated from PnCL, PnIL, and PnIR respectively (Table 2). Mapping to the sequences of Rfam database revealed that 5% of the total small RNA reads of Pn IL and Pn IR were tRFs, whereas it constituted 1% from Pn CL (Figure 4A). The number of unique tRF sequences identified from PnCL, PnIL, and PnIR libraries were 7001, 11479, and 23746, respectively (Supplementary Datas 1–3). The length distribution showed a similar trend in all the three sRNA libraries, with 21 nt being the most prominent length group, and it constituted respectively 26.06, 21.43, and 17.46 percentage of the total tRF reads in the PnCL, PnIL, and PnIR libraries (Figure 4B). 5′G was identified as the prominent terminal nucleotide for black pepper tRFs in all of the length classes, with an average of 84.7% of total tRFs possessing G as 5′terminal nucleotide (Figure 5). The 5′tRFs occupied the predominant group compared to internal and 3′tRFs in the sRNA libraries (Figure S3). A summary of the 5′tRFs derived from diverse tRNA precursors represented from each sRNA library was shown in Table S3 and Figure 6. Among the tRFs, 5′tRFs of Ala-tRNA^AGC^ showed a higher abundance in all of the datasets and the normalized read count (Reads per Million) was greatest for its 20 nt 5′tRFs. 5′Ala-tRNA^CGC^ was observed to be the second most abundant tRF and the normalized read was observed to be higher for its 24 nt variants. Along with this, the 5′tRFs of Val^AAC^, Val^CAC^, Asp^GTC^, and Gly^TCC^ were also found. The 5′tRFs of Ala-tRNA^CGC^ displayed higher expression in Pn IL and Pn IR, compared with the normalized read counts of Pn CL. The length distribution of 5′tRFs showed a similar trend in all of the three sRNA libraries with a higher abundance of 19–25 nt variants (Figure 7). Most of the 5′tRFsexhibited high expression in the PnIL compared with other two libraries. While 5′Met^CAT^tRFs, 19 nt 5′tRFs of Gly^TCC^ and Phe^GAA^ and 20 nt Val^AAC^ 5′tRF had shown higher read counts in the Pn IR compared with Pn CL and Pn IL. The major cleavage of the 5′tRFs was identified at 11 or 12 nucleotides upstream of the corresponding anticodon, especially at T or A nucleotides in the D loop/stem of the particular tRNA (Figure 8). The abundance of 5′tRFs, their precise cleavage from the anticodon loop and differential expression indicated the functional role of 5′tRFs during defense response in black pepper.

**Table 2:** **Distribution of tRNA-derived small RNAs in the black pepper sRNA libraries**.

**Small RNA library**	**Total clean reads**	**Total tsRNA/tRF**	**Unique tsRNA/tRF**
Pn CL	18,629,003	256,369	7001
Pn IL	12,621,368	616,263	11,479
Pn IR	12,948,539	662,399	23,746

### Prediction of target genes and mapping of tRF mediated cleavage

**Figure 9:**
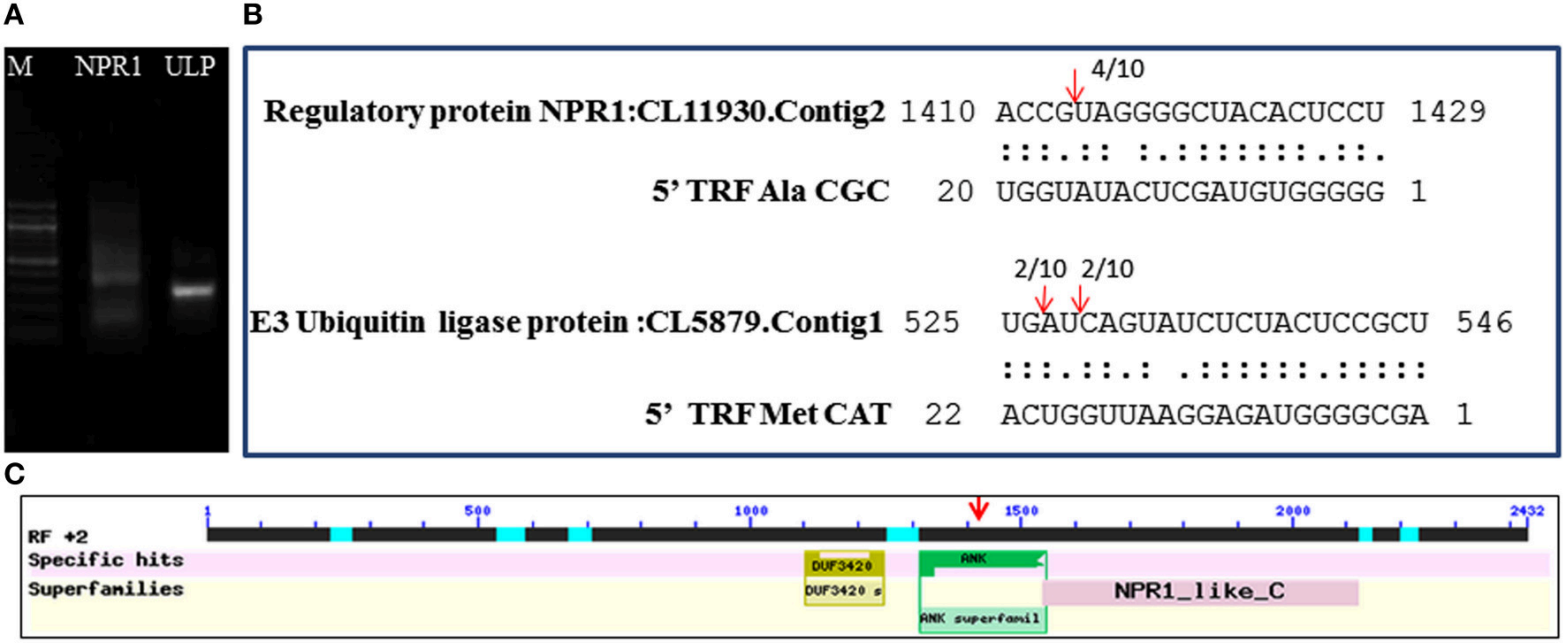
**Mapping of the cleavage sites on the targets identified from the black pepper transcriptome by 5′RLM-RACE experiments**. **(A)** 5′ RLM RACE Amplified product of cleaved mRNAs of NPR1 and E3 Ubiquitin ligase protein (UPL) in 1.5% agarose gel. M indicated 100 bp DNA ladder (New England BioLabs). The expected size of UPL1 and NPR1 amplicons, such as 320 and 300 bp respectively were detected from the gel. **(B)** Cleavage sites were mapped on the tRF aligned regions in the target mRNAs of NPR1 and E3 Ubiquitin ligase. **(C)** The cleavage site was mapped at the region coding for ankyrin repeat domain of the NPR1 mRNAs.

The mechanism and characteristics of target recognition of plant tRFs were proposed to be similar to microRNAs (Loss-Morais et al., 2013). The targets of 5′tRFs were predicted from the stress responsive mRNA transcriptome that consisted of 73,112 assembled sequences (manuscript under preparation). Most of the predicted targets possessed 2 to 12 nt seed matches, as proposed for the miRNAs, and the mechanism of inhibition in most of the target alignments was predicted to be cleavage (Table S4). The predicted targets of these functional sRNAs included a broad range of genes representing growth conditions, developmental stages and stress responses (Table 3). The two mRNA homologs of Non-expresser of pathogenesis related proteins (NPR1) such as CL11930.contig2 and CL5879.contig1 were identified as the putative target of 5′Ala^CGC^tRF. NPR1 was reported as a key regulator of salicylic acid-dependent gene expression during systemic acquired resistance (SAR) (Dong, 2004; Maier et al., 2011). The DNA replication complex GINS protein PSF2 and the vesicle transport protein SFT2B were predicted as targets for 5′Ala^AGC^tRF, while pectinesterase-2 precursor, pentatricopeptide repeat protein, and a probable polygalacturonase were predicted for 5′tRF Val^AAC^. The mRNAs coding for calmodulin binding protein, ubiquitin ligase protein and ABC transporter G family proteins were identified as targets for 5′ Met^CAT^tRF. The transcriptome assembled sequence of the target NPR1 variants such as CL11930.contig2 and CL5879.contig1 consisted of 2431 and 695 nucleotides respectively, and both the transcripts shared very high nucleotide similarity (Figure S4). The 5′tRF Ala^CGC^ and 5′tRF Met^CAT^ mediated cleavage on the respective target mRNAs of NPR1 (CL11930.Contig2) and E3 ubiquitin ligase protein (CL5879) was validated by modified 5′RLM RACE experiments (Figure 9). The cleavage sites were mapped on the 5′Ala^CGC^tRF aligned regions in the coding region of the ankyrin repeat domain, which is the conserved protein-protein interaction motif in NPR1.

**Table 3:** **The predicted targets of different 5′tRFs from black pepper**.

**TRF Acc**.	**Target gene**	**Target ID**	**Inhibition**
Ala CGC	NPR1-like protein	CL11930.Contig2	Cleavage
		Unigene19317	
	Unnamed protein product	CL6217.Contig1	Cleavage
Ala AGC	DNA replication complex GINS protein PSF2	CL843.Contig4	Cleavage
	Hypothetical protein VITISV_037210	CL1619.Contig2	Translation
	Vesicle transport protein SFT2B	Unigene248	Cleavage
Val AAC	Predicted protein	CL6118.Contig1	Cleavage
	Pectinesterase-2 precursor, putative	CL11276.Contig2	Cleavage
	Pentatricopeptide repeat protein	Unigene14666	Cleavage
	Probable polygalacturonase non-catalytic subunit JP630-like	Unigene11670	Cleavage
		Unigene29565	
		CL3980.Contig2	
		Unigene12808	
		Unigene19404	
Val CAC	Pherophorin-dz1 protein	CL6779.Contig3	Cleavage
	Hypothetical protein ARALYDRAFT_493093	Unigene1307	Cleavage
	UDP-glucosyltransferase	Unigene6425	Cleavage
	ATP binding protein, putative	CL8978.Contig3	Cleavage
	PREDICTED: uncharacterized protein LOC100267290	CL7095.Contig1	Cleavage
Asp GTC	PREDICTED: uncharacterized protein LOC100782887	CL7172.Contig2	Cleavage
HisGTG	UPF0614 protein C14orf102 homolog	CL9959.Contig1	Cleavage
	ATP synthase CF1 alpha subunit	CL5021.Contig2	Cleavage
Met_CAT	Conserved hypothetical protein	Unigene4870	Cleavage
	Hypothetical protein VOLCADRAFT_92962	Unigene17473	Cleavage
	Calmodulin binding protein, putative	CL11955.Contig1	Translation
	Uncharacterized protein LOC100776993	CL11955.Contig2	Translation
	PREDICTED: ABC transporter G family member 15-like	Unigene10972	Cleavage
	Ubiquitin ligase protein	CL5879.Contig1	Cleavage
		CL5879.Contig2	
	Carboxylic ester hydrolase, putative	CL5622.Contig1	Translation
Gly_TCC	PREDICTED: protein TRANSPARENT TESTA 12-like	CL10606.Contig1	Cleavage
		CL10606.Contig2	
	Protein binding protein	Unigene8302	Cleavage
Phe_GAA	Binding protein	CL12716.Contig2	Cleavage
	DEAD-box ATP-dependent RNA helicase	CL1422.Contig2	Translation
	Predicted protein	Unigene21029	Cleavage
	Soluble starch synthase 3	Unigene328	Cleavage
Ser_GCT	Photosystem II protein K	Unigene9945	Cleavage
	Copper/zinc superoxide dismutase	CL6300.Contig5	Cleavage
		CL6300.Contig6	
		CL6300.Contig7	
		CL6300.Contig8	
	Probable anion transporter 3,	Unigene12023	Cleavage
	Glycerol-3-phosphate dehydrogenase-like, transcript variant 1	CL8788.Contig2	Cleavage
	Transmembrane protein TPARL, putative	Unigene8037	Translation
	Serine/threonine-protein kinase sepA-like	CL11327.Contig1	Cleavage
		CL11327.Contig4	
	NEDD8-like protein RUB1	Unigene2954	Translation

### Expression profiling of target mRNAs of NPR1

**Figure 10:**
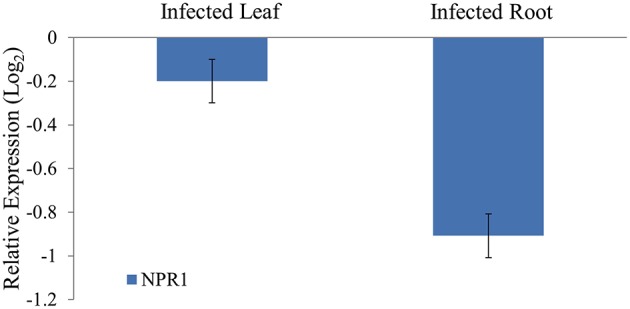
**Quantitative real time analysis of the NPR1 mRNAs from black pepper during *P. capsici* infection**. The expression was normalized with 5.8S rRNA as the endogenous control and calibrated with the expression of leaf and root of control uninfected plants. For each tissue, triplicates were analyzed and standard deviation was represented as the error bar. NPR1 mRNAs were down-regulated in the pathogen infected root compared to the control root.

To study the differential expression of the NPR1 mRNAs in *Phytophthora* infected black pepper plants, quantitative real time RT-PCR was performed. The expression of the NPR1 mRNAs was found to be reduced both in the pathogen stressed leaf and root tissues compared to the control leaf and root. In the *Phytophthora* infected root tissues, the expression of the NPR1 mRNAs was down regulated (Figure 10). It is possible that the down-regulation of defense genes such as NPR1 attributed to the susceptibility of black pepper to the infection of *P. capsici*. As NPR1 mRNAs were predicted to be targeted by sRNAs such as 5′Ala^CGC^tRF, they might act as the regulators of these genes, and the higher expression of 5′Ala^CGC^tRFs in turn responsible for the cleavage of these target mRNAs and hence, the reduced expression of NPR1 during pathogen infection in black pepper.

### Sequence diversity of 5′Ala^CGC^tRFs and their targets from other plants

**Figure 11:**
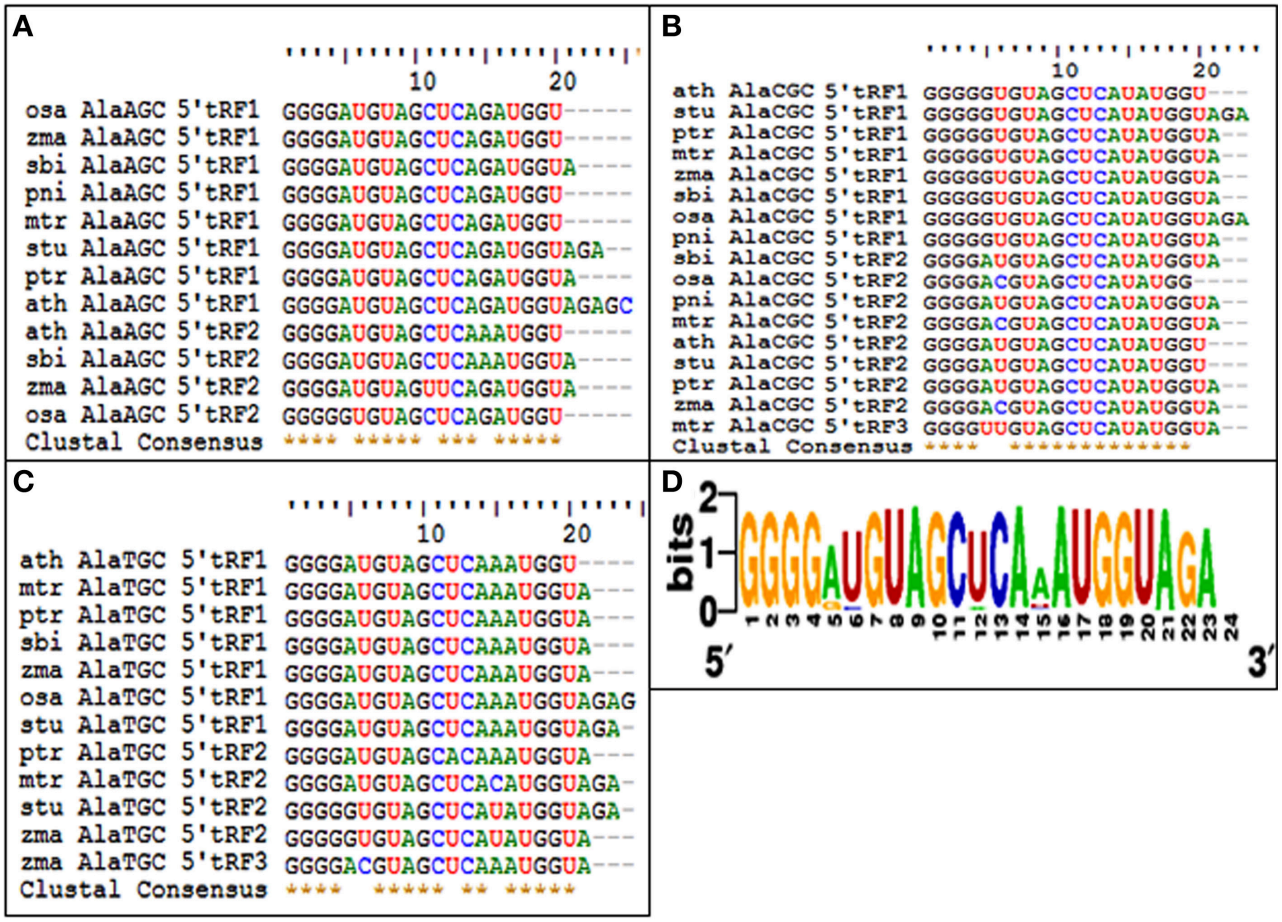
**The conservation of 5′tRFs from Alanine tRNAs among the angiosperm plants**. Sequence alignments of **(A)** 5′Ala^AGC^ tRFs, **(B)** 5′Ala^CGC^ tRFs, and **(C)** 5′Ala^TCG^ tRFs. **(D)** Multiple alignments of all the 5′AlatRFs were represented as sequence logo. ath, *A. thaliana*; mtr, *M. truncatula*; zma, *Z. mays*; ptr, *P. trichocarpa*; osa, *O. sativa*; sbi, *S. bicolor*; stu, *S. tuberosum*; and pni, *P. nigrum*.

The 5′tRFs of the Alanine tRNAs were analyzed from the high-throughput sRNAome of diverse plant species. The conservation analysis of the most prominent variants of the 5′Ala-tRFsfrom each sRNA library (Supplementary Data 4) revealed their conservation pattern among the land plants (Figure 11) and the sequence alignment of different 5′Ala-tRFs were represented as a sequence logo. Most of the plant species possessed the highly conserved tRFs generated from the 5′end of each of the Alanine tRNAs. Although there was variation observed among the different 5′Ala-tRF sequences, 5′Ala^CGC^tRF1 with the sequence GGGGGUGUAGCUCAUAUGGU, 5′Ala^AGC^tRF1 with the sequence “GGGGAUGUAGCUCAGAUGGU” and 5′Ala^TGC^tRF1 with the sequence “GGGGAUGUAGCUCAAAUGGU” was present in all of the angiosperm plants. Several defense responsive genes, such as the NBS LRR disease resistance protein, the CBS domain containing protein and the LRR receptor like serine threonine kinase were predicted as targets of 5′Ala^CGC^tRF1. The NPR1 homologs were not identified as potential targets from any of the other plant species studied (Table S5). This result indicates that the 5′Ala^CGC^tRF1 plays an important role in the stress response of plants and NPR1 may be a specific target of 5′Ala^CGC^tRF1 from black pepper that belong to magnoliids, the lower angiosperm plants.

## Discussion

The foot rot or quick wilt is a major disease of black pepper and, similar to *Phytophthora* diseases of many other crops, it causes extensive loss in the pepper plantations. The identification of sRNAs generated during *P. capsici* infection has great potential for elucidating the mechanisms of stress response, as well as adopting effective disease resistance strategies in black pepper. The preliminary cloning and sequencing of the sRNA enriched library, from *Phytophthora* infected black pepper plants, revealed three tRNA-derived small RNAs such as 5′Arg^TCG^tRNA, 5′Ala^AGC^tRNA, and 3′Gly^TCC^tRNA. The sRNAs from non-coding structural RNAs, such as tRNAs and rRNAs have long been regarded as degradation products. However, recently it was reported that abundantly expressed sRNAs from tRNA (Haussecker et al., 2010; Garcia-Silva et al., 2012; Jagadeeswaran et al., 2012), rRNA (Zywicki et al., 2012) or snoRNAs (Brameier et al., 2011) play a significant physiological role in the stress response of eukaryotes, ranging from yeast to higher organisms. The occurrence of 5′ and 3′ tRNA fragments in yeast cells, plants, and mammalian cell lines during oxidative stress suggested the conserved feature of oxidative stress-induced tRNA cleavage in eukaryotes (Li et al., 2008; Thompson et al., 2008; Cole et al., 2009; Lee et al., 2009). *P. capsici* interaction leads to oxidative stress and a significant increase in enzyme activities and phenolic compounds in the host plants (Koç et al., 2011). The tRFs from the 5′ ends of Gly-tRNA and Asp-tRNA were reported during phosphate deficiency from *Arabidopsis* (Hsieh et al., 2009). Consistent with previous reports, the 18 nt 3′tRF from the Gly^TCC^ also had a typical 3′CCA end (Sobala and Hutvagner, 2011) that was added during the maturation of tRNA by tRNA nucleotidyl transferase, before its export from the nucleus to the cytoplasm (Xiong and Steitz, 2006). Among the three tRFs studied, the 5′Ala^CGC^tRF showed higher expression in the leaf and root of *P. capsici* infected plants. The 5′tRFs associated with argonaute proteins (AGO2) were reported strongly induced during *Pseudomonas syringae* infection and abiotic stress treatments in *Arabidopsis* (Loss-Morais et al., 2013). The 19 nt 5′tRF from Gly-tRNA^TCC^ was reported to highly accumulate in *Arabidopsis* root tissues under phosphate starved stress conditions, whereas the differential expression of 5′Ala^AGC^ and Pro^CGG^ tRFs was demonstrated in the rice callus and leaves (Chen et al., 2011).

The deep sequenced sRNA libraries such as PnIL and PnIR was further analyzed to identify tRF abundance, distribution and their functional role during pathogen infections. In both the PnIL and PnIR sRNA libraries, the tRF reads constitute a higher proportion compared to the control leaf library. As stated previously (Cole et al., 2009; Lee et al., 2009; Loss-Morais et al., 2013), the up-regulation of tRFs during stress conditions was suggested to be a common mechanism that exists between plants and animals. The biogenesis and function of tRFs are still unclear, however their positional specificity, abundance, and spatio-temporal expression variation indicates their selective processing from tRNAs. Although tRFs could be formed from different sources of tRNAs, such as organelles (chloroplast) or the nucleus, they were coordinatively regulated and their processing shares the same mechanism in plants (Hackenberg et al., 2015). The processing and 5′maturation of tRNA involved RNAse P, a ubiquitous endonuclease present in all the eukaryotes (Jarrous and Gopalan, 2010; Gobert et al., 2013). The protein only Ribonuclease P enzymes called PROP were found to support RNAse P activity in both the organelles and the nucleus in plants (Gutmann et al., 2012). Moreover, the tRFs association to argonaute (AGO) proteins (Kumar et al., 2014) and translation inhibition roles of certain tRFs (Zhang et al., 2009) indicated their potential role in the RNAi pathway.

We observed the unequal distribution of different tRNA sequences in the sRNA dataset with a higher predominance of 5′tRFs. As reported in *Arabidopsis* (Loss-Morais et al., 2013), a 5′G was the prominent terminal nucleotide for tRFs in different length classes from all the three sRNA libraries. 5′Ala^AGC^tRFs and 5′Ala^CGC^tRFs were identified as the most prevalent tRFs in all three datasets. Individual 5′tRFs from each tRNA showed higher abundance in a narrow size distribution from 19 to 24 nt. Previously, in *Arabidopsis* (Loss-Morais et al., 2013), the size distribution pattern of AGO-associated tRFs revealed a predominance of 18–22 nt sequences in which 19 nt 5′tRFs were reported as the prominent group. In most of the deep sequencing experiments, these non-coding structural RNA-derived sRNAs are filtered away during the initial steps of analysis. However, the abundance of a certain set of tRNAs, their cleavage from the anticodon loop and their differential expression during pathogen infection established their functional role in the context of defense response in black pepper. The preferences in the nucleotide cleavage from the 5′end of mature tRNAs further supported the fact that tRFs were not randomly degraded, but produced via a specific pathway.

The critical target genes predicted for the 5′Ala^CGC^tRFs included NPR1, DNA replication complex GINS protein PSF2, vesicle transport protein SFT2B, Pectinesterase-2 precursor, Protein kinase APK1B, pentatricopeptide repeat protein, probable polygalacturonase, calmodulin binding protein ubiquitin ligase protein, and ABC transporter G family proteins. These genes were reported to be expressed during *Phytophthora* interactions in *A. thaliana* and *S. tuberosum* (Sandhu et al., 2009; Bos et al., 2010). During the pathogen infection, the expression of NPR1 mRNAs was down-regulated in the black pepper plants. NPR1 was also reported to act as key regulatory component in the crossroads of multiple defense pathways (Dong, 2004). NPR1 protein could act as a receptor of the plant defense hormone salicylic acid (Maier et al., 2011; Wu et al., 2012), and regulate profound transcriptional changes to broad spectrum plant immune responses, such as SAR. The induction of SAR further led to the expression of pathogenesis related (PR) protein and protect the plant against bacterial, fungal, and viral infections (Dong, 2004). PR proteins, generally coded by the host plant were induced specifically during pathological situations, and instrumental in impeding further pathogen ingress and multiplication (Van Loon and Van Strien, 1999; van Loon et al., 2006). To prevent the untimely activation of SAR in the absence of pathogen challenge, NPR1 was continuously cleared from the nucleus by the proteasome (Spoel et al., 2009). In *Theobroma cacao* plants knocked down with a synthetic miRNA targeting TcNPR3 mRNA, a repressor of NPR1 was reported to be more resistant to the infection of *P. capsici* (Shi et al., 2013). NPR1 protein was reported to interact with a TGA class of basic domain leucine zipper transcription factors that regulates salicylic acid responsive elements in the promoters of Pathogenesis Related (PR) protein genes (Després et al., 2003). Resistance against the infection of *P. sojae* in soybean (Sugano et al., 2013), *P. capsici* in *Arabidopsis* (Wang Y. et al., 2013), and *T. cacao* (Shi et al., 2013) was reported to be mediated by the salicylic acid signaling in which NPR1 was the key component. Therefore, the reduced expression of NPR1 in the leaf and root tissues of the infected plants might lead to the susceptibility of the black pepper to *Phytophthora* infection. Modified 5′RLM RACE analysis revealed cleavage on the predicted target site of NPR1 mRNA and the cleavage was mapped at the ankyrin repeat domain, which is one of the conserved protein-protein interaction motifs in the NPR1 proteins (Maier et al., 2011).

The target cleavage was also identified in the mRNAs of ubiquitin ligase by 5′Met^CAT^tRF, which was highly expressed in PnIR. All steps of plant immune responses were reported to be modulated by the ubiquitin ligase family of enzymes (Duplan and Rivas, 2014). It was also suggested that these enzymes induced during both PTI and ETI, could regulate the expression of defense related signaling in plants (Craig et al., 2009). The ubiquitin/proteasome system were reported as extensively involved in the downstream signaling processes of pathogen perception and targeting the proteins for degradation (Craig et al., 2009). The detection of cleavage products of tRFs supported that post transcriptional silencing could be one of the regulatory mechanism of these functional sRNAs.

## Conclusions

Our results demonstrated the existence of small RNAs derived from tRNAs and their potential gene regulatory role during the *P. capsici* infection of black pepper. Small RNAs derived from the 5′ end of mature tRNAs (5′tRFs) were highly expressed under pathogen stress in black pepper. 5′Ala^CGC^tRFs and 5′Met^CAT^tRFs were found to target defense-responsive mRNAs, such as NPR1 and ubiquitin ligase. These mRNAs are critical in signaling responses leading to the activation of pathogenesis related (PR) proteins, which are the key players in plant pathogen interactions. Although 5′AlatRF from other plant species showed very high sequence conservation to *P. nigrum*, the NPR1 homologs were not identified as 5′Ala-tRF targets from other plant species, and instead, defense genes, such as NBS-LRR kinases, were predicted to be potential target genes. The tRF binding sites in the black pepper NPR1 gene, the mRNA cleavage pattern and the subsequent down regulation suggested a functional role of 5′AlatRFs in stress signaling pathways in black pepper, the unique magnoliid plant. More research is needed to understand tRF biogenesis, the molecular mechanisms underlying their gene silencing functions and their coordinated activity with a diverse set of regulatory sRNAs to fine-tune gene expression. The evaluation of these candidate sRNAs in plant pathogen interaction will lead to advanced disease resistant strategies with wider application in the improvement of stress tolerance in plants.

## Author contributions

Design of the Work: ES and SA; the acquisition, analysis, or interpretation of data for the work: SA. Drafting the work and revising it critically for important intellectual content: SA and ES. Final approval of the version to be published: ES and SA.

### Conflict of interest statement

The authors declare that the research was conducted in the absence of any commercial or financial relationships that could be construed as a potential conflict of interest.

## Abbreviations

sRNA: small RNA
tRNA: transfer RNA
tRF: tRNA derived small RNA fragments
NPR1: Non-expresser of pathogenesis related protein
PAMP: pathogen associated molecular patterns.
