# Supplementary material for: Transfer RNA Derived Small RNAs Targeting Defense Responsive Genes Are Induced during Phytophthora capsici Infection in Black Pepper (Piper nigrum L.)
Source: Front Plant Sci. 2016 Jun 1;7:767. doi: 10.3389/fpls.2016.00767 (PMC4887504; doi:10.3389/fpls.2016.00767)
Supplement: Supplementary file 1 [file Table1.pdf]

Transfer RNA derived small RNAs targeting defence responsive genes are induced during *Phytophthora capsici* infection in black pepper (*Piper nigrum* L.)

Table S1: List of primers used for the stem-loop qRT-PCR. The number inside the brackets represents PCR amplification efficiency of the primers .

Primer ID	Sequence (5′-3′)
Pni TRF1 SLP	GTCGTATCCAGTGCAGGGTCCGAGGTATTCGCACTGGATACGACTCTACC
Pni TRF2 SLP	GTATCCAGTGCAGGGTCCGAGGTATTCGCACTGGATACGACTCCACTGCG
Pni TRF3 SLP	GTCGTATCCAGTGCAGGGTCCGAGGTATTCGCACTGGATACGACTGGTGC
Pni TRF1 FP	TGTTCGCTGTGACCGCATAG (1.7476)
Pni TRF2 FP	TCTGGGGGTGTAGCTCATAT (1.4051)
Pni TRF3 FP	CCTTCGCTCTCCCGGCAAAC (1.5978)
URP	GTGCAGGGTCCGAGGT

Table S2: Small RNAs identified from *Phytophthora capsici* infected black pepper plants by preliminary cloning and sequencing

Seq ID	Sequences (5′-3′)	Sequence Length	Annotation
Pn_TRF1	GGGGGUGUAGCUCAUAUGGUAGA	23	5′TRF(Ala-tRNA^{CGC})
Pn_TRF2	GACCGCAUAGCGCAGUGGA	19	5′TRF(Arg-tRNA$^{TCG)}$
Pn_TRF3	CUCCCGGCAAACGCACCA	18	3′TRF (Gly-tRNA^{TCC})
Pn_rRF1	UACGACUCUCGGCAACGGAUA	21	Pn_rRF1
Pn_rRF2	GGGUAAAAGCUCGUUUGAUUCU	22	Pn_rRF2
Pn_rRF3	GACCCUGUUGAGCUUGAC	18	Pn_rRF3
Pn_rRF4	GGGAAAAGGAUUGGCUCUGAGGGC	24	Pn_rRF4
Pn_rRF5	CAAUCUAAAUCCCUUAACGAG	21	Pn_rRF5
Pn_rRF6	GGAGUAAUGAUUAACAGG	18	Pn_rRF6
Pn_rRF7	GGACGGACUGGGAACGGUUC	20	Pn_rRF7
Pn_rRF8	GAGGGGAACAACAGCUGGA	19	Pn_rRF8
Pn_rRF9	GGGUGCGAUCAUACCAGCA	19	Pn_rRF9
Pn_rRF10	GCCCGGACCCUGUUGCUCCA	20	Pn_rRF10
Pn_rRF11	GACUGAGGACUUCAGCACG	19	Pn_rRF12
Pn_rRF12	CCCUUUGUCGCACAGAUUCGA	21	Pn_rRF13
Pn_15	UAUGGAAGCGGAGCUACCUC	20	Unannotated
Pn_16	UGAGGUAGUAGGUUGUAU	18	Unannotated
Pn_17	ACGCUGUAUCUUAUGUA	17	Unannotated
Pn_18	CCAUUACGGCCGGGCACUCAGGUGG	25	Unannotated
Pn_19	AUGGCCACUCUGCGUGACCACUGGU	25	Unannotated
Pn_20	UCCGCCCGAGGCUUUAACUCGGGUCA	26	Unannotated
Pn_21	GUUGGGGAAGGUCACUAGGGGGUCA	25	Unannotated
Pn_22	AGGAUGUGGUGAGUCGACCGCGGC	24	Unannotated
Pn_23	CUAUGGACCUUUGCCAGC	18	Unannotated
Pn_24	CUCGUGACCUUAGCUGA	17	Unannotated
Pn_25	AAUGGCCACUCUGCGUUGCCAU	22	Unannotated

Pn_26	UCCCUAGAGGGUAAUGGCCA	20	Unannotated
Pn_27	GUAACAGUGCCGGCAAGCUUCGGCGG	26	Unannotated
Pn_28	AUGGCCACUCUGCGUGCCAA	20	Unannotated
Pn_29	GGCCAAUGAAUUCACUCAGGUGG	23	Unannotated
Pn_30	AUGGCCACUCUGCGUUGUCCAU	22	Unannotated
Pn_31	AUGGCCACUCUGCGUUACCUG	21	Unannotated
Pn_32	GAGCAGGACUAAGCUUCCCCC	21	Unannotated
Pn_33	CUUCAGAGUCCUUAUGGCCACUCUGGU	27	Unannotated
Pn_34	GAAGCACCUUCGACGAGUCGAGU	23	Unannotated
Pn_35	CAUUUUAGGAAAAUCACAAU	20	Unannotated
Pn_36	AAGAUGUGGUGAGUCGACGCGC	22	Unannotated
Pn_37	GACUGAGGACUUCAGCACG	19	Unannotated
Pn_38	GAAGCACCUUCGACGAG	17	Unannotated
Pn_39	AUGGCCACUCUGCGUUGAUCCAUGU	25	Unannotated
Pn_40	GUUCCAAUGAAUUCACUCAGGUGG	24	Unannotated

Table S3. Summary of 5′ tRF reads from sRNA libraries of *P. nigrum* (Relative cloning frequency is calculated as percentage of the total sequencing reads).

Sequence	Length	Origin	Relative Cloning Frequency (%)		
			Pn IL	Pn IR	Pn CL
GGGGATGTAGCTCAGATGG	19	AlaAGC-5′	0.1329	0.1514	0.0675
GGGGATGTAGCTCAGATGGT	20	AlaAGC-5′	0.6604	0.5733	0.2180
GGGGATGTAGCTCAGATGGTA	21	AlaAGC-5′	0.6342	0.5157	0.1961
GGGGATGTAGCTCAGATGGTAG	22	AlaAGC-5'	0.3923	0.3189	0.0988
GGGGATGTAGCTCAGATGGTAGA	23	AlaAGC-5′	0.3078	0.2612	0.0789
GGGGATGTAGCTCAGATGGTAGAG	24	AlaAGC-5'	0.2548	0.2122	0.0294
GGGGATGTAGCTCAGATGGTAGAGC	25	AlaAGC-5'	0.0365	0.0488	0.0009
GGGGGTGTAGCTCATATGGT	20	AlaCGC-5'	0.0104	0.0082	0.0064
GGGGGTGTAGCTCATATGGTA	21	AlaCGC-5'	0.0392	0.0269	0.0212
GGGGGTGTAGCTCATATGGTAG	22	AlaCGC-5'	0.0437	0.0298	0.0142
GGGGGTGTAGCTCATATGGTAGA	23	AlaCGC-5'	0.0491	0.0342	0.0150
GGGGGTGTAGCTCATATGGTAGAG	24	AlaCGC-5'	0.0631	0.0444	0.0067
GGGGGTGTAGCTCATATGGTAGAGC	25	AlaCGC-5'	0.0132	0.0141	0.0001
GGTGTCGTGGTGTAGTTGGT	20	Val AAC-5'	0.0254	0.0415	0.0130
GGTGTCGTGGTGTAGTTGGTT	21	Val AAC-5'	0.0920	0.0762	0.0524
GGTGTCGTGGTGTAGTTGGTTA	22	Val AAC-5'	0.1084	0.0878	0.0567
GGTGTCGTGGTGTAGTTGGTTAT	23	Val AAC-5'	0.0699	0.0536	0.0257
GGTGTCGTGGTGTAGTTGGTTATC	24	Val AAC-5'	0.0444	0.0381	0.0104
GGTGTCGTGGTGTAGTTGGTTATCA	25	Val AAC-5'	0.0573	0.0435	0.0120
GTCTGGGTGGTGTAGTTGGT	20	Val CAC-5'	0.0105	0.0127	0.0052

GTCTGGGTGGTGTAGTTGGTT	21	Val CAC-5'	0.0209	0.0149	0.0097
GTCTGGGTGGTGTAGTTGGTTA	22	Val CAC-5'	0.0288	0.0177	0.0098
GTCTGGGTGGTGTAGTTGGTTAT	23	Val CAC-5'	0.0091	0.0055	0.0029
GTCTGGGTGGTGTAGTTGGTTATC	24	Val CAC-5'	0.0044	0.0027	0.0010
GTCTGGGTGGTGTAGTTGGTTATCA	25	Val CAC-5'	0.0041	0.0025	0.0010
GCGTTTGTAGTCCAACGGT	19	Gly TCC5'	0.0185	0.1950	0.0054
GCGTTTGTAGTCCAACGGTT	20	Gly TCC5'	0.0390	0.0332	0.0053
GCGTTTGTAGTCCAACGGTTA	21	Gly TCC5'	0.0231	0.0202	0.0036
GCGTTTGTAGTCCAACGGTTAG	22	Gly TCC5'	0.0005	0.0006	0.0001
GGTGGCTGTAGTTTAGTGG	19	His GTG5'	0.0063	0.0060	0.0037
GGTGGCTGTAGTTTAGTGGT	20	His GTG5'	0.0184	0.0149	0.0048
GGTGGCTGTAGTTTAGTGGTT	21	His GTG5'	0.0152	0.0135	0.0114
GGTGGCTGTAGTTTAGTGGTTA	22	His GTG5'	0.0197	0.0176	0.0104
GGTGGCTGTAGTTTAGTGGTTAG	23	His GTG5'	0.0141	0.0133	0.0020
GGTGGCTGTAGTTTAGTGGTTAGA	24	His GTG5'	0.0153	0.0143	0.0011
GGTGGCTGTAGTTTAGTGGTTAGAA	25	His GTG5'	0.0111	0.0113	0.0004
GGTGGCTGTAGTTTAGTGGTTAGAAT	26	His GTG5'	0.0119	0.0131	0.0006
AGCGGGGTAGAGGAATTGG	19	Met CAT 5'	0.0032	0.0069	0.0005
AGCGGGGTAGAGGAATTGGT	20	Met CAT 5'	0.0076	0.0148	0.0023
AGCGGGGTAGAGGAATTGGTC	21	Met CAT 5'	0.0115	0.0251	0.0025
AGCGGGGTAGAGGAATTGGTCA	22	Met CAT 5'	0.0091	0.0179	0.0021
AGCGGGGTAGAGGAATTGGTCAA	23	Met CAT 5'	0.0086	0.0300	0.0044
AGCGGGGTAGAGGAATTGGTCAAC	24	Met CAT 5'	0.0041	0.0058	0.0005
AGCGGGGTAGAGGAATTGGTCAACT	25	Met CAT 5'	0.0066	0.0112	0.0015
AGCGGGGTAGAGGAATTGGTCAACTC	26	Met CAT 5'	0.0014	0.0018	0.0001
GGGATTGTAGTTCAATCGG	19	Asp GTC5'	0.0017	0.0037	0.0015
GGGATTGTAGTTCAATCGGT	20	Asp GTC5'	0.0030	0.0072	0.0015
GGGATTGTAGTTCAATCGGTC	21	Asp GTC5'	0.0203	0.0150	0.0067
GGGATTGTAGTTCAATCGGTCA	22	Asp GTC5'	0.0069	0.0050	0.0033
GCGGGGATAGCTCAGTTGG	19	Phe GAA5'	0.0046	0.0095	0.0016
GCGGGGATAGCTCAGTTGGG	20	Phe GAA5'	0.0065	0.0060	0.0012
GCGGGGATAGCTCAGTTGGGA	21	Phe GAA5'	0.0027	0.0026	0.0005
GCGGGGATAGCTCAGTTGGGAG	22	Phe GAA5'	0.0037	0.0030	0.0003
GCGGGGATAGCTCAGTTGGGAGA	23	Phe GAA5'	0.0036	0.0031	0.0003
GCGGGGATAGCTCAGTTGGGAGAG	24	Phe GAA5'	0.0024	0.0018	0.0001
GGAGAGATGGCTGAGTGGAC	20	Ser GCT5'	0.0015	0.0008	0.0001
GGAGAGATGGCTGAGTGGACT	21	Ser GCT5'	0.0043	0.0025	0.0006
GGAGAGATGGCTGAGTGGACTA	22	Ser GCT5'	0.0048	0.0031	0.0007
GGAGAGATGGCTGAGTGGACTAA	23	Ser GCT5'	0.0035	0.0019	0.0005

GGAGAGATGGCTGAGTGGACTAAA	24	Ser GCT5'	0.0026	0.0013	0.0002
GGAGAGATGGCTGAGTGGACTAAAG	25	Ser GCT5'	0.0017	0.0010	0.0001

Table S4: Targets predicted for 5′tRFs from black pepper.

TRF Acc.	Target Acc.	Expectation (E)	Target Accessibility (UPE)	Alignment	Target Description	Inhibition
Ala CGC	CL11930.Contig2	3.0	15.964	TRF 20 UGGUAUACUCGAUGUGGGGG 1<br>:::.:: :.:::::::.::.<br>Target 1410 ACCGUAGGGGCUACACUCCU 1429	NPR1-like protein [Musa ABB Group]	Cleavage
Ala CGC	Unigene19317	3.0	15.964	TRF 20 UGGUAUACUCGAUGUGGGGG 1<br>:::.:: :.:::::::.::.<br>Target 333 ACCGUAGGGGCUACACUCCU 352	Regulatory protein NPR1	Cleavage
Ala CGC	CL6217.Contig1	3.0	18.678	TRF 20 UGGUAUACUCGAUGUGGGGG 1<br>:::::.: ::::::.:::..<br>Target 141 ACCAUGUUAGCUACGCCCUU 160	unnamed protein product	Cleavage
Ala AGC	CL843.Contig4	3.0	20.672	TRF 20 UGGUAGACUCGAUGUAGGGG 1<br>::::::::::: ..:::::<br>Target 220 UCCAUCUGAGCUCUGUCCCC 239	DNA replication complex GINS protein PSF2	Cleavage
Ala AGC	CL1619.Contig2	3.0	21.875	TRF 20 UGGUAGACUCGAUGUAGGGG 1<br>:.:.::::: ::::.::.::<br>Target 619 AUCGUCUGAUCUACGUCUCC 638	hypothetical protein VITISV_037210	Translation
Ala AGC	Unigene248	3.0	22.293	TRF 20 UGGUAGACUCGAUGUAGGGG 1<br>:. .:::::::::.:::::.<br>Target 314 AUGGUCUGAGCUAUAUCCCU 333	PREDICTED: vesicle transport protein SFT2B	Cleavage
Val AAC	CL6118.Contig1	2.5	17.222	TRF 20 UGGUUGAUGUGGUGCUGUGG 1<br>:::::::: :.::.::::.:<br>Target 388 ACCAACUAGAUCAUGACAUC 407	predicted protein	Cleavage
Val AAC	CL11276.Contig2	3.0	10.169	TRF 20 UGGUUGAUGUGGUGCUGUGG 1<br>.::::.: .:::.:::::::<br>Target 1234 GCCAAUUCUACCGCGACACC 1253	Pectinesterase-2 precursor, putative	Cleavage
Val AAC	Unigene3879	3.0	10.637	TRF 21 UUGGUUGAUGUGGUGCUGUGG 1<br>::::: : ::::::::::::<br>Target 1225 AACCACCAUCACCACGACACC1245	uncharacterized protein LOC100253879	Cleavage
Val AAC	Unigene14666	2.5	1.041	TRF 20 UGGUUGAUGUGGUGCUGUGG 1<br>::::::: :::::: :::::<br>Target 154 ACCAACUCCACCACCACACC 173	pentatricopeptide repeat protein	Cleavage
Val AAC	Unigene11670	3.0	18.017	TRF 20 UGGUUGAUG-UGGUGCUGUGG 1<br>::::::::: ::::::.::.:<br>Target 674 ACCAACUACGACCACGGCAUC 694	probable polygalacturonase non-catalytic subunit JP630-like	Cleavage

Val AAC	Unigene29565	3.0	21.13	TRF 20 UGGUUGAUG-UGGUGCUGUGG 1<br>::::::::: ::::::.::.:<br>Target 564 ACCAACUACGACCACGGCAUC 584	PREDICTED: probable polygalacturonase non-catalytic subunit JP630	Cleavage
Val AAC	CL3980.Contig2	3.0	21.146	TRF 20 UGGUUGAUG-UGGUGCUGUGG 1<br>::::::::: ::::::.::.:<br>Target 541 ACCAACUACGACCACGGCAUC 561	PREDICTED: probable polygalacturonase non-catalytic subunit JP630	Cleavage
Val AAC	Unigene12808	3.0	21.146	TRF 20 UGGUUGAUG-UGGUGCUGUGG 1<br>::::::::: ::::::.::.:<br>Target 597 ACCAACUACGACCACGGCAUC 617	PREDICTED: probable polygalacturonase non-catalytic subunit JP630	Cleavage
Val AAC	Unigene19404	3.0	21.146	TRF 20 UGGUUGAUG-UGGUGCUGUGG 1<br>::::::::: ::::::.::.:<br>Target 706 ACCAACUACGACCACGGCAUC 726	PREDICTED: probable polygalacturonase non-catalytic subunit JP630	Cleavage
Val CAC	CL6779.Contig3	2.5	6.89	TRF 20 UGGUUGAUGUGGUGGGUCUG 1<br>::::.:: :::::::.::.:<br>Target 421 ACCAGCUCCACCACCUAGGC 440	Pherophorin-dz1 protein	Cleavage
Val CAC	Unigene1307	2.5	19.919	TRF 20 UGGUUGAUGUGGUGGGUCUG 1<br>::::.:: ::::.:::.:::<br>Target 177 ACCAGCUCCACCGCCCGGAC 196	hypothetical protein ARALYDRAFT_493093	Cleavage
Val CAC	Unigene6425	3.0	12.117	TRF 20 UGGUUGAUGUGGUGGGUCUG 1<br>:::: :::::.:::: ::::<br>Target 163 ACCACCUACAUCACCAAGAC 182	UDP-glucosyltransferase	Cleavage
Val CAC	CL8978.Contig3	3.0	15.335	TRF 20 UGGUUGAUGUGGUGGGUCUG 1<br>.::::::::..:::::: ::<br>Target 99 GCCAACUACGUCACCCAAAC 118	ATP binding protein, putative	Cleavage
Val CAC	CL7095.Contig1	3.0	16.978	TRF 20 UGGUUGAUGUGGUGGGUCUG 1<br>:..: .::.:::::::::::<br>Target 852 AUUAUUUAUACCACCCAGAC 871	PREDICTED: uncharacterized protein LOC100267290	Cleavage
Asp GTC	CL7172.Contig2	3.0	16.678	TRF 20 UGGCUAACUUGAUGUUAGGG 1<br>:: ::::::.:.:::::::<br>Target 1972 CCCUAUUGAAUUGCAAUCCC 1991	PREDICTED: uncharacterized protein LOC100782887	Cleavage
His GTG	CL9959.Contig1	2.5	10.27	TRF 20 UGGUGAUUUGAUGUCGGUGG 1<br>::::::::.::.::::.::<br>Target 234 UCCACUAAAUUAUAGCCGCC 253	UPF0614 protein C14orf102 homolog	Cleavage
His GTG	CL5021.Contig2	3.0	5.023	TRF 20 UGGUGAUUUGAUGUCGGUGG 1<br>::::::::::::.: ::.:.<br>Target 7385 ACCACUAAACUAUACCCGCU 7404	ATP synthase CF1 alpha subunit	Cleavage

Met CAT	Unigene4870	0.0	14.066	TRF 20 UGGUUAAGGAGAUGGGGCGA 1<br>::::::::::::::::::::<br>Target 521 ACCAAUUCCUCUACCCCGCU 540	Conserved hypothetical protein	Cleavage
Met CAT	Unigene17473	2.0	10.523	TRF 20 UGGUUAAGGAGAUGGGGCGA 1<br>::: :::::::::::::::<br>Target 318 ACCCCUUCCUCUACCCCGCU 337	hypothetical protein VOLCADRAFT_92962	Cleavage
Met CAT	CL11955.Contig1	3.0	8.073	TRF 20 UGGUUAAGGAGAUGGGGCGA 1<br>::::::::::. ::::::.<br>Target 953 ACCAAUUCCUUCACCCCGUC 972	calmodulin binding protein, putative	Translation
Met CAT	CL11955.Contig2	3.0	8.073	TRF 20 UGGUUAAGGAGAUGGGGCGA 1<br>::::::::::. ::::::.<br>Target 530 ACCAAUUCCUUCACCCCGUC 549	PREDICTED: uncharacterized protein LOC100776993	Translation
Met CAT	Unigene10972	3.0	11.982	TRF 20 UGGUUAAGGAGAUGGGGCGA 1<br>:.:: :::::::::..::.:<br>Target 101 AUCACUUCCUCUACUUCGUU 120	PREDICTED: ABC transporter G family member 15-like	Cleavage
Met CAT	CL5879.Contig1	3.0	14.731	TRF 20 UGGUUAAGGAGAUGGGGCGA 1<br>:.::.: .::::::.:::::<br>Target 527 AUCAGUAUCUCUACUCCGCU 546	Ubiquitin ligase protein	Cleavage
Met CAT	CL5879.Contig2	3.0	14.731	TRF 20 UGGUUAAGGAGAUGGGGCGA 1<br>:.::.: .::::::.:::::<br>Target 565 AUCAGUAUCUCUACUCCGCU 584	Ubiquitin ligase protein	Cleavage
Met CAT	CL5622.Contig1	3.0	15.407	TRF 20 UGGUUAAGGAGAUGGGGCGA 1<br>:::::::: :: ::.:.:::<br>Target 31 ACCAAUUCAUCGACUCUGCU 50	Carboxylic ester hydrolase, putative	Translation
Gly TCC	CL10606.Contig1	2.5	18.079	TRF 20 UUGGCAACCUGAUGUUUGCG 1<br>::.::: ::.:::::.::::<br>Target 1736 AAUCGUAGGGCUACAGACGC 1755	PREDICTED: protein TRANSPARENT TESTA 12-like	Cleavage
Gly TCC	CL10606.Contig2	2.5	18.1	TRF 20 UUGGCAACCUGAUGUUUGCG 1<br>::.::: ::.:::::.::::<br>Target 1757 AAUCGUAGGGCUACAGACGC 1776	PREDICTED: protein TRANSPARENT TESTA 12-like	Cleavage
Gly TCC	Unigene8302	3.0	13.026	TRF 20 UUGGCAACCUGAUGUUUGCG 1<br>:: :::::::::::::: :.<br>Target 335 AAACGUUGGACUACAAAAGU 354	Protein binding protein, putative	Cleavage
Phe GAA	CL12716.Contig2	2.5	18.182	TRF 20 GGGUUGACUCGAUAGGGGCG 1<br>::: :::::::: :::.:::<br>Target 72 CCCCACUGAGCUCUCCUCGC 91	Binding protein	Cleavage
Phe GAA	CL1422.Contig2	3.0	14.762	TRF 20 GGGUUGACUCGAUAGGGGCG 1<br>:::::.::: :::::.:::<br>Target 1160 CCCAAUUGAACUAUCUCCGA 1179	DEAD-box ATP-dependent RNA helicase	Translation

Phe GAA	Unigene21029	3.0	18.777	TRF 20 GGGUUGACUCGAUAGGGGCG 1<br>:.:::: :::::::.:..::<br>Target 238 CUCAACGGAGCUAUUCUUGC 257	Predicted protein	Cleavage
Phe GAA	Unigene328	3.0	22.123	TRF 20 GGGUUGACUCGAUAGGGGCG 1<br>:..::: ::::::::.:.::<br>Target 3932 CUUAACAGAGCUAUCUCUGC 3951	PREDICTED: soluble starch synthase 3	Cleavage
Ser GCT	Unigene9945	0.0	16.329	TRF 20 CAGGUGAGUCGGUAGAGAGG 1<br>::::::::::::::::::::<br>Target 1101 GUCCACUCAGCCAUCUCUCC 1120	Photosystem II protein K	Cleavage
Ser GCT	CL6300.Contig5	2.5	5.669	TRF 20 CAGGUGAGUCGGUAGAGAGG 1<br>:.:::::::.:::::.:::<br>Target 28 AUUCACUCAGUCAUCUUUCC 47	Copper/zinc superoxide dismutase	Cleavage
Ser GCT	CL6300.Contig6	2.5	5.669	TRF 20 CAGGUGAGUCGGUAGAGAGG 1<br>:.:::::::.:::::.:::<br>Target 28 AUUCACUCAGUCAUCUUUCC 47	Copper/zinc superoxide dismutase	Cleavage
Ser GCT	CL6300.Contig7	2.5	5.669	TRF 20 CAGGUGAGUCGGUAGAGAGG 1<br>:.:::::::.:::::.:::<br>Target 28 AUUCACUCAGUCAUCUUUCC 47	Copper/zinc superoxide dismutase	Cleavage
Ser GCT	CL6300.Contig8	2.5	5.669	TRF 20 CAGGUGAGUCGGUAGAGAGG 1<br>:.:::::::.:::::.:::<br>Target 28 AUUCACUCAGUCAUCUUUCC 47	Copper/zinc superoxide dismutase	Cleavage
Ser GCT	Unigene12023	2.5	18.154	TRF 20 CAGGUGAGUCGGUAGAGAGG 1<br>::.:..::::.:::::.:::<br>Target 138 GUUCGUUCAGUCAUCUUUCC 157	Probable anion transporter 3	Cleavage
Ser GCT	CL8788.Contig2	3.0	7.367	TRF 20 CAGGUGAGUCGGUAGAGAGG 1<br>: :::. :::::::.:::::<br>Target 176 GCCCAUCCAGCCAUUUCUCC 195	Glycerol-3-phosphate dehydrogenase-like, transcript variant 1	Cleavage
Ser GCT	Unigene8037	3.0	10.189	TRF 20 CAGGUGAGUCGGUAGAGAGG 1<br>::: :::: ::::::::::<br>Target 74 UUCCUCUCAUCCAUCUCUCC 93	Transmembrane protein TPARL, putative	Translation
Ser GCT	CL11327.Contig1	3.0	12.671	TRF 20 CAGGUGAGUCGGUAGAGAGG 1<br>::..::::::::.:::.:..<br>Target 3316 GUUUACUCAGCCGUCUUUUU 3335	Serine/threonine-protein kinase sepA-like	Cleavage
Ser GCT	CL11327.Contig4	3.0	12.671	TRF 20 CAGGUGAGUCGGUAGAGAGG 1<br>::..::::::::.:::.:..<br>Target 3712 GUUUACUCAGCCGUCUUUUU 3731	Serine/threonine-protein kinase sepA-like	Cleavage
Ser GCT	Unigene2954	3.0	23.67	TRF 20 CAGGUGAGUCGGUAGAGAGG 1<br>:::::::: :: :::::::<br>Target 30 CUCCACUCACCCCUCUCUCC 49	NEDD8-like protein RUB1	Translation

Table S5: Targets of 5’Ala tRFs predicted from different plant species

Plant species	Target database	Target Acc.	Expectation (E)	Target Accessibility (UPE)	Alignment	Target Description	Inhibition
Arabidopsis thaliana	DFCI Gene Index (AGI), version 15	TC392728	2.5	17.902	tRF 20 UGGUAUACUCGAUGUGGGGG 1<br>::::::: ::::.::::..:<br>Target 132 ACCAUAUCAGCUGCACCUUC 151	Classical arabinogalactan protein 26 precursor	Cleavage
		TC381137	2.5	17.822	tRF 20 UGGUAUACUCGAUGUGGGGG 1<br>::::::: ::::.::::..:<br>Target 132 ACCAUAUCAGCUGCACCUUC 151	Cluster: Classical arabinogalactan protein 26 precursor	Cleavage
		EG439050	3.0	20.906	tRF 20 UGGUAUACUCGAUGUGGGGG 1<br>.::::.::.::: ::::.::<br>Target 118 GCCAUGUGGGCUUCACCUCC 137	UniRef100_Q6DBG4 Cluster: At1g54575	Cleavage
		TC386636	3.0	21.103	tRF 20 UGGUAUACUCGAUGUGGGGG 1<br>.::::.::.::: ::::.::<br>Target 180 GCCAUGUGGGCUUCACCUCC 199	-	Cleavage
		TC383076	3.0	21.103	tRF 20 UGGUAUACUCGAUGUGGGGG 1<br>.::::.::.::: ::::.::<br>Target 845 GCCAUGUGGGCUUCACCUCC 864	At3g08955 [*Arabidopsis thaliana*]	Cleavage
		NP237738	3.0	19.359	tRF 20 UGGUAUACUCGAUGUGGGGG 1<br>:.:: :::.:.:::::::.:<br>Target 569 AUCAAAUGGGUUACACCCUC 588	GB|AB007648.1|gene_id:MKD15.6~unknown protein	Cleavage
		TC367453	3.0	19.359	tRF 20 UGGUAUACUCGAUGUGGGGG 1<br>:.:: :::.:.:::::::.:<br>Target 592 AUCAAAUGGGUUACACCCUC 611	similar to UniRef100_A7Q7L7	Cleavage
Oryza sativa	TIGR genome cDNA OSA1 Release 5(OSA1R5), version 5	LOC_Os12g30290.1	2.5	17.804	tRF 20 UGGUAUACUCGAUGUGGGGG 1<br>::::: :: ::::::::.::<br>Target 51 ACCAUUUGUGCUACACCUCC 70	cDNA|retrotransposon protein	Cleavage
		LOC_Os03g26791.1	3.0	13.703	tRF 20 UGGUAUACUCGAUGUGGGGG 1<br>:::::::: ::::.:.:..:<br>Target 333 ACCAUAUGUGCUAUAUCUUC 352	cDNA|expressed protein	Cleavage
		LOC_Os07g31100.1	3.5	11.505	tRF 20 UGGUAUACUCGAUGUGGGGG 1<br>:::::.::::::.::. .::<br>Target 174 ACCAUGUGAGCUGCAUAUCC 193	transposon protein, CACTA, En/Spm sub-class	Cleavage

<table>
<tr><td rowspan="3">Oryza sativa</td><td rowspan="3">TIGR genome cDNA OSA1 Release 5(OSA1R5), version 5</td><td>LOC_Os05g 19720.1</td><td>3.5</td><td>12.363</td><td>tRF 20 UGGUAUACUCGAUGUGGGGG 1
:::::.:::::::.::. .::
Target 783 ACCAUGUGAGCUGCAUAUCC 802</td><td>cDNA|transposon protein, putative, CACTA, En/Spm sub-class, expressed</td><td>Cleavage</td></tr>
<tr><td>LOC_Os02g 02210.1</td><td>3.5</td><td>20.269</td><td>tRF 20 UGGUAUACUCGAUGUGGGGG 1
:::::::::::.::. ::::..
Target 1282 ACCAUAUGAGUUAUUCCCUU
1301</td><td>cDNA|aminotrans ferase, putative, expressed</td><td>Cleavage</td></tr>
<tr><td>LOC_Os08g 16460.1</td><td>3.5</td><td>14.961</td><td>tRF 20 UGGUAUACUCGAUGUGGGGG 1
::::::: .:::: :.::::
Target 603 ACCAUAUAGGCUAAAUCCCC 622</td><td>cDNA|NBS-LRR disease resistance protein, putative, expressed</td><td>Cleavage</td></tr>
<tr><td rowspan="6">Oryza sativa</td><td rowspan="6">Oryza sativa (rice), transcript, JGI genomic project, Phytozome, phytozome v7.0,</td><td>CI651705</td><td>3.0</td><td>23.65</td><td>tRF 20 UGGUAUACUCGAUGUGGGGG 1
::..:.::.::::::::::
Target 265 ACUGUGUGGGCUACACCCCA 284</td><td>POT family protein, expressed</td><td>Cleavage</td></tr>
<tr><td>FG966991</td><td>3.5</td><td>12.415</td><td>tRF 20 UGGUAUACUCGAUGUGGGGG 1
::::::: :::::. :.:::
Target 188 ACCAUAUUAGCUAUUCUCCC 207</td><td>NADH-ubiquinone oxidoreductase chain 2</td><td>Cleavage</td></tr>
<tr><td>TC501424</td><td>3.5</td><td>13.027</td><td>tRF 20 UGGUAUACUCGAUGUGGGGG 1
: ::: ::::::::.::::
Target 1356 UCAAUAGGAGCUACAUCCCC1375</td><td>CBS domain containing protein</td><td>Cleavage</td></tr>
<tr><td>BM421123</td><td>3.5</td><td>16.209</td><td>tRF 20 UGGUAUACUCGAUGUGGGGG 1
:::::.: : ::.:.:::::
Target 56 ACCAUGUCACCUGCGCCCCC 75</td><td>Phosphoenolpyru vate carboxylase ryza sativa subsp</td><td>Translation</td></tr>
<tr><td>TC487530</td><td>3.5</td><td>18.672</td><td>tRF 20 UGGUAUACUCGAUGUGGGGG 1
.:::::: :::: ..:::::
Target 1612 GCCAUAUCAGCUCUGCCCCC1631</td><td>Plant disease resistance polyprotein-like</td><td>Cleavage</td></tr>
<tr><td>NP13032627</td><td>3.5</td><td>18.672</td><td>tRF 20 UGGUAUACUCGAUGUGGGGG 1
.:::::: :::: ..:::::
Target 412 GCCAUAUCAGCUCUGCCCCC 431</td><td>Protein kinase domain containing protein</td><td>Cleavage</td></tr>
</table>

Populus trichocarpa	*Populus trichocarpa* (poplar), unigene, DFCI Gene Index (PPLGI), version 5	TC160436	3.0	11.483	tRF 20 UGGUAUACUCGAUGUGGGGG 1<br>::::::: ::.::::.:::<br>Target 115 ACCAUAUCAGUUACAUCCCG 134	Probable LRR receptor-like serine/threonine-protein kinase	Cleavage
		CK100253	3.0	17.462	tRF 20 UGGUAUACUCGAUGUGGGGG 1<br>.::::.: :::::::.:::.<br>Target 20 GCCAUGUCAGCUACAUCCCU 39	UniRef100_A7PHK3 Cluster: Chromosome chr17 scaffold_16	Cleavage
		TC169670	3.0	15.54	tRF 20 UGGUAUACUCGAUGUGGGGG 1<br>:::: ::::::::::.:.<br>Target 1333 ACCAGUUGAGCUACACCUCU1352	Type II chlorophyll a/b binding protein from photosystem I precursor	Cleavage
		TC159341	3.5	17.223	tRF 20 UGGUAUACUCGAUGUGGGGG 1<br>:.::::: . .:::::::::<br>Target 639 AUCAUAUAGUUUACACCCCC 658	Serine/threonine protein phosphatase	Translation
		TC139299	3.5	12.979	tRF 20 UGGUAUACUCGAUGUGGGGG 1<br>:::::. :. ::.:::::::<br>Target 217 ACCAUGGGGACUGCACCCCC 236	DNA binding with one finger 5 protein	Translation
Solanum tuberosum	*Solanum tuberosum* (potato), unigene, DFCI Gene Index (STGI), version 13	TC194599	3.0	10.586	tRF 20 UGGUAUACUCGAUGUGGGGG 1<br>::. ::::::::::.:::::<br>Target 2268 ACUUGAUGAGCUACGCCCCC2287	Potato late blight resistance protein R3a	Cleavage
		TC224342	3.5	14.429	tRF 20 UGGUAUACUCGAUGUGGGGG 1<br>:..:::::::: .::::..:<br>Target 354 AUUAUAUGAGCUCUACCUUC 373	NBS-LRR resistance protein-like protein)	Cleavage
		TC206564	3.5	15.498	tRF 20 UGGUAUACUCGAUGUGGGGG 1<br>:..:::::::: .::::..:<br>Target 6 AUUAUAUGAGCUCUACCUUC 25	NBS-LRR resistance protein-like protein	Cleavage
		TC219922	3.5	20.779	tRF 20 UGGUAUACUCGAUGUGGGGG 1<br>:..:::::::: .::::..:<br>Target 467 AUUAUAUGAGCUCUACCUUC 486	NBS-LRR resistance protein-like protein	Cleavage
		TC199214	3.5	12.396	tRF 20 UGGUAUACUCGAUGUGGGGG 1<br>::.:: ::: .::::.::::<br>Target 1003 ACUAUUUGAAUUACAUCCCC1022	Chloroplast RNA binding protein precursor	Translation
