# Supplementary material for: Transfer RNA Derived Small RNAs Targeting Defense Responsive Genes Are Induced during Phytophthora capsici Infection in Black Pepper (Piper nigrum L.)
Source: Front Plant Sci. 2016 Jun 1;7:767. doi: 10.3389/fpls.2016.00767 (PMC4887504; doi:10.3389/fpls.2016.00767)
Supplement: Supplementary file 2 [file DataSheet1.PDF]

Transfer RNA derived small RNAs targeting defence responsive genes are induced during *Phytophthora capsici* infection in black pepper (*Piper nigrum* L.)

Supplementary Data1

TRNA mapped small RNAs from conrol leaf library (Pn CL) of black pepper (Upto 10 read counts)

Seq. ID	Length	Read Count	Sequence
t0000011	20	40605	GGGGATGTAGCTCAGATGGT
t0000013	21	36540	GGGGATGTAGCTCAGATGGTA
t0000025	22	18412	GGGGATGTAGCTCAGATGGTAG
t0000033	23	14695	GGGGATGTAGCTCAGATGGTAGA
t0000038	19	12570	GGGGATGTAGCTCAGATGG
t0000042	22	10555	GGTGTCGTGGTGTAGTTGGTTA
t0000047	21	9758	GGTGTCGTGGTGTAGTTGGTT
t0000065	22	6846	GGGGATGTAGCTCAAACGGTAG
t0000083	24	5472	GGGGATGTAGCTCAGATGGTAGAG
t0000092	23	4787	GGTGTCGTGGTGTAGTTGGTTAT
t0000102	23	4137	GGGGATGTAGCTCAAACGGTAGA
t0000108	21	3947	GGGGGTGTAGCTCATATGGTA
t0000182	23	2791	GGGGGTGTAGCTCATATGGTAGA
t0000191	21	2724	GGTGGCTGTAGTTTAGTGGTA
t0000195	22	2654	GGGGGTGTAGCTCATATGGTAG
t0000221	20	2427	GGTGTCGTGGTGTAGTTGGT
t0000228	21	2384	GGGGATGTAGCTCAAATGGTA
t0000246	25	2238	GGTGTCGTGGTGTAGTTGGTTATCA
t0000270	21	2122	GGTGGCTGTAGTTTAGTGGTT
t0000283	22	2077	GGTGTCGTCGTGTAGTTGGTTA
t0000294	22	2026	GGGGTTGTAGCTCAAATGGTAG
t0000300	22	2008	GGGGATGTAGCTCAAATGGTAG
t0000313	24	1936	GGTGTCGTGGTGTAGTTGGTTATC
t0000316	22	1929	GGTGGCTGTAGTTTAGTGGTTA
t0000345	22	1831	GTCTGGGTGGTGTAGTTGGTTA
t0000354	21	1801	GTCTGGGTGGTGTAGTTGGTT
t0000370	23	1762	GGGGATGTAGCTCAAATGGTAGA
t0000454	23	1518	GGGGTTGTAGCTCAAATGGTAGA
t0000590	24	1257	GGGGGTGTAGCTCATATGGTAGAG
t0000592	21	1252	GGGATTGTAGTTCAATCGGTC
t0000637	20	1201	GGGGGTGTAGCTCATATGGT
t0000775	24	1055	GGGGATGTAGCTCAAACGGTAGAG
t0000809	19	1015	GCGTTTGTAGTCCAACGGT
t0000850	20	979	GCGTTTGTAGTCCAACGGTT
t0000853	20	977	GTCTGGGTGGTGTAGTTGGT
t0000929	22	926	GGTGTCGTTGTGTAGTTGGTTA
t0000979	20	885	GGTGGCTGTAGTTTAGTGGT
t0001102	23	816	AGCGGGGTAGAGGAATTGGTCAA
t0001165	20	780	GGGGATGTAGCTCAAATGGT
t0001181	23	766	GGTGTCGTCGTGTAGTTGGTTAT
t0001195	24	757	GGGGATGTAGCTCAAATGGTAGAG
t0001250	25	733	GGTGTCGTCGTGTAGTTGGTTATCA
t0001278	24	718	GGGGTTGTAGCTCAAATGGTAGAG
t0001340	19	690	GGTGGCTGTAGTTTAGTGG
t0001361	21	678	GCGTTTGTAGTCCAACGGTTA
t0001379	24	674	GGTGTCGTCGTGTAGTTGGTTATC
t0001508	19	629	GGTGTCGTGGTGTAGTTGG
t0001544	22	618	GGGATTGTAGTTCAATCGGTCA
t0001829	21	543	GTCTGGGTGGTGTAGTTGGTC
t0001880	23	534	GTCTGGGTGGTGTAGTTGGTTAT
t0002039	25	501	GGTGGCTGTAGTTTAGTGGTAAGAA
t0002188	22	475	GTCTGGGTGGTGTAGTTGGTCA
t0002279	21	460	AGCGGGGTAGAGGAATTGGTC
t0002509	20	427	AGCGGGGTAGAGGAATTGGT
t0002553	20	422	GCGTTTGTAGTCCAACGGTA
t0002629	22	412	TCCATTGTCGTCTAGTCCGGTT
t0002737	22	400	AGCGGGGTAGAGGAATTGGTCA
t0002910	23	379	GGTGGCTGTAGTTTAGTGGTTAG

t0003086	23	361	GGTGTCGTTGTGTAGTTGGTTAT
t0003166	22	352	GTCTGGGTGGTGTAGTCGGTTA
t0004033	18	291	GCGGGGATAGCTCAGTTG
t0004073	19	289	GCGGGGATAGCTCAGTTGG
t0004076	21	288	TCCATTGTCGTCTAGTCCGGT
t0004221	19	280	GGGATTGTAGTTCAATCGG
t0004278	25	277	AGCGGGGTAGAGGAATTGGTCAACT
t0004366	20	272	GGGATTGTAGTTCAATCGGT
t0005019	22	244	GTGGTCGTGCCGGAGTGGTTAT
t0005035	25	243	GGTGTCGTTGTGTAGTTGGTTATCA
t0005317	20	232	GCGGGGATAGCTCAGTTGGG
t0005385	21	230	GGGGATGTAGCTCAGATGGGA
t0005640	20	221	AGGGATATAACTCAGCGGTA
t0005994	21	209	GCGTTTGTAGTCCAACGGTAA
t0006091	24	207	GGTGTCGTTGTGTAGTTGGTTATC
t0006132	24	206	GGTGGCTGTAGTTTAGTGGTTAGA
t0006304	22	200	GTCTGGGTTGTGTAGTTGGTTA
t0006505	23	195	TCCATTGTCGTCTAGTCCGGTTA
t0006618	25	193	GTCTGGGTGGTGTAGTTGGTTATCA
t0006643	20	192	GTCGATATGTCCGAGTGGTT
t0006918	19	184	GTCTGGGTGGTGTAGTTGG
t0006987	24	182	GTCTGGGTGGTGTAGTTGGTTATC
t0007389	25	174	GGGGATGTAGCTCAGATGGTAGAGC
t0007710	21	168	GGGGATGTAGCTCAGATGGTT
t0008075	21	161	GCGGATATGGTCGAATGGTAA
t0008436	22	154	TCCGTTGTAGTCTAGTTGGTTA
t0009259	22	142	GTGGACGTGCCGGAGTGGTTAT
t0009469	20	139	GCTGGAATAGCTCAGTTGGT
t0010034	20	132	GTCGTTGTAGTATAGTGGTA
t0010462	22	127	GGAGAGATGGCTGAGTGGACTA
t0010628	20	126	GGGGATGTAGCTCAGATGGG
t0010652	22	125	GGTGTCGTAGTGTAGTTGGTTA
t0011404	19	118	GGGGGTGTAGCTCATATGG
t0012069	21	111	GGAGAGATGGCTGAGTGGACT
t0012135	18	111	GTTGAGATGGCCGAGTTG
t0012136	24	111	GGGGATGTAGCTCAGATGGTAGAA
t0012894	24	105	GGGGATGTAGCTCAAACGGTAGAA
t0012987	18	104	GCGTTTGTAGTCCAACGG
t0013082	20	103	GCACCAGTGGTCTAGTGGTA
t0013087	26	103	GGTGGCTGTAGTTTAGTGGTTAGAAT
t0013200	24	102	AGCGGGGTAGAGGAATTGGTCAAC
t0013388	20	101	GCGTTTGTAGTCCAACGGTC
t0013497	23	101	GGAGAGATGGCTGAGTGGACTAA
t0013637	29	100	GGGGCTGTAGCTCAGCTGGGAGAGCACCT
t0013653	20	99	AGTCCCGTAGCTCAGTTGGT
t0013821	20	98	GCGGATATGGTCGAATGGTA
t0014213	20	96	GGGATTGTAGTTCAATCGGA
t0014474	20	94	TCCATTGTCGTCTAGTCCGG
t0014492	23	94	GTCTGGGTGGTGTAGTCGGTTAT
t0014704	20	93	TGGGATGTAGCTCAGATGGT
t0014845	19	92	GTCAGGATGGCCGAGTGGT
t0014863	19	92	GCGCCTGTAGCTCAGTGGA
t0014908	27	92	GGGATTGTAGTTCAATCGGTCAGAGCA
t0014932	20	91	GTTGAGATGGCCGAGTTGGT
t0015159	24	90	GGGGATGTAGCTCAGATGGTAGAT
t0015194	19	90	AGCGGGGTAGAGGAATTGG
t0015221	21	90	CGCGGGGTGGAGCAGTTCGGT
t0015392	21	89	GCGGGGATAGCTCAGTTGGGA
t0015472	27	89	ACGGACTGTAAATTCGTTGACGATATG
t0015589	23	88	GGGGATGTAGCTCAGATGGGAGA
t0015680	21	87	GTCGATATGTCCGAGTGGTTA
t0016300	20	84	GGGATGTAGCTCAGATGGTA
t0016509	20	83	AGGGATGTAGCGCAGCTTGG

t0016735	21	82	GTCGTTGTAGTATAGTGGTAA
t0016891	22	81	AGGGATGTAGCGCAGCTTGGTA
t0017074	26	81	GGTGTCGTGGTGTAGTTGGTTATCAC
t0017660	22	78	GCGGATATGGTCGAATGGTAAA
t0017737	24	78	AAGGTTGTGGGTTCAAATCATGCC
t0017854	22	77	GGGGATGTAGCTCAGATGGTAA
t0017951	21	77	TGGGATGTAGCTCAGATGGTA
t0018152	25	76	GTCTGGGTGGTGTAGTTGGTCATCA
t0018200	23	76	AGGGATGTAGCGCAGCTTGGTAG
t0018635	22	74	GGGGATGTAGCTCAGATGGTAT
t0018822	20	73	GCGGGTGTAGTTTAGTGGTA
t0018979	25	73	GGGGATGTAGCTCAAACGGTAGAGC
t0019970	25	69	GGGGATGTAGCTCAGATGGTAAAAA
t0020592	22	67	GGGGATGTAGCTCAGATGGGAG
t0020641	21	67	GCGTTTGTAGTCCAACGGTCA
t0020763	22	66	GTTGTCGTGGTGTAGTTGGTTA
t0020889	25	66	GGTGGCTGTAGTTTAGTGGTTAGAA
t0021043	22	65	GTCTGGGTCGTGTAGTTGGTTA
t0021100	24	65	AGGGATGTAGCGCAGCTTGGTAGC
t0021246	24	65	GGGGATGTAGCTCAGATGGTAAAA
t0021700	24	63	TCCATTGTCGTCTAGTCCGGTTAG
t0021799	19	63	GGGATGTAGCTCAGATGGT
t0021821	20	63	GTTGGTTAGGATACTCGGCT
t0021980	24	63	GGGGGTGTAGCTCATATGGTAGAA
t0022107	22	62	GGTGTCGTGGTGTAGTTGGTTT
t0022427	23	61	GGTGGCTGTAGTTTAGTGGTTAA
t0022432	21	61	GTGGATGTAGCTCAGATGGTA
t0022790	24	60	GTCGTTGTAGTATAGTGGTAAGTA
t0023067	19	60	GGGGATGTAGCTCAAATGG
t0023542	20	58	GGGGATGTAGCTCAGATGGA
t0023839	23	58	GTCTGGGTTGTGTAGTTGGTTAT
t0024498	22	56	GCGGGGATAGCTCAGTTGGGAG
t0024799	20	56	GTGGATGTAGCTCAGATGGT
t0025224	21	55	GGGGATGTAGCTCAGATGGAA
t0025416	24	54	GGGCCTGTAGCTCAGAGGATTAGA
t0025521	23	54	GGGGATGTAGCTCAGATGGTAGT
t0025559	19	54	GCACCAGTGGTCTAGTGGT
t0025871	22	53	TGTGTCGTGGTGTAGTTGGTTA
t0026137	23	53	GGTGTCGTAGTGTAGTTGGTTAT
t0026355	23	52	GTGGTCGTGCCGGAGTGGTTATC
t0027396	23	50	GCGGGGATAGCTCAGTTGGGAGA
t0027700	26	50	TGGTAGAGCATTTGACTGCAGATCAA
t0028137	19	49	TCCATTGTCGTCTAGTCCG
t0028403	20	48	GTCAAGATGGCCGAGTTGGT
t0028548	21	48	GGCGGATGTGGCCAAGTGGAA
t0028804	23	48	GTCGTTGTAGTATAGTGGTAAGT
t0028843	21	48	GCGGAAGTAGTTCAGTGGTAG
t0029326	18	47	GGGGATGTAGCTCAGATG
t0029585	24	46	GGGGATGTAGCTCAAACGGTAGAT
t0029802	20	46	TGGTAGAGCATTTGACTGCA
t0029907	22	46	GGGGATGTAGCTCAGATGGTTT
t0030649	22	45	GTCGTTGTAGTATAGTGGTAAG
t0030791	25	45	GGGGATATGGCGAAATTGGTAGACG
t0031249	20	44	GGGGATGTAGCTCAGCTGGT
t0031611	22	43	TCCGTTGTAGTCTAGGTGGTTA
t0031631	27	43	GGGCCTGTAGCTCAGAGGATTAGAGCA
t0031796	25	43	GGGCCTGTAGCTCAGAGGATTAGAG
t0031837	23	43	GGGGATGTAGCTCAGATGGTATA
t0031854	19	43	TTGGTTAGGATACTCGGCT
t0032259	24	42	GGTGTCGTAGTGTAGTTGGTTATC
t0032401	24	42	GGAGAGATGGCTGAGTGGACTAAA
t0032507	21	42	GTTGTCGTGGTGTAGTTGGTT
t0032526	22	42	GTCTGGGTAGTGTAGTTGGTTA

t0032876	21	42	AGGGATATAACTCAGCGGTAG
t0033140	22	41	CCGACCTTAGCTCAGTTGGCAG
t0033147	21	41	GACCGCATAGCGCAGTGGATT
t0033375	21	41	GTGGTAGAGCATTTGACTGCA
t0034132	20	40	GTCGTTGTAGTATAGTGGTG
t0034321	21	40	CTAGCGGTTAGGACATTGGAC
t0034354	22	40	AGTGGTAGAGCATTTGACTGCA
t0034415	20	40	CGCGGGGTAGAGCAGTTTGG
t0034671	19	39	GTCGTTGTAGTATAGTGGT
t0034690	22	39	CGCGGGGTGGAGCAGTTCGGTA
t0034711	21	39	GGGGATATGGCGAAATTGGTA
t0035176	23	39	CCGACCTTAGCTCAGTTGGCAGA
t0035194	23	39	CGCGGGGTAGAGCAGTTTGGTAG
t0035346	21	39	TGTGTCGTGGTGTAGTTGGTT
t0035385	22	39	TGGGATGTAGCTCAGATGGTAG
t0035452	20	39	GGGGATGTAGCTCAGGTGGT
t0035490	23	39	GGGCCTGTAGCTCAGAGGATTAG
t0036189	21	38	GGGATGTAGCTCAGATGGTAG
t0036549	24	37	GTGGACGTGCCGGAGTGGTTATCG
t0036871	23	37	GCGGATATGGTCGAATGGTAAAA
t0036935	20	37	GCGGAAGTAGTTCAGTGGTA
t0037115	21	37	GTCGTTGTAGTATAGTGGTGA
t0037198	25	37	GGGGATGTAGCTCAGATGGTAGATT
t0037365	25	37	GGTGTCGTAGTGTAGTTGGTTATCA
t0037765	21	36	GGGGATGTAGCTCATATGGTA
t0037883	25	36	GTCTGGGTGGTGTAGTCGGTTATCA
t0038109	18	36	GGTGTCGTGGTGTAGTTG
t0038267	23	36	GCACCAGTGGTCTAGTGGTAGAA
t0038484	21	35	GTGTCGTGGTGTAGTTGGTTA
t0038770	24	35	GGGATTGTAGTTCAATCGGTCAGA
t0039394	23	35	GGGGATGTAGCTCAGATGGTAAA
t0040202	20	34	GTGTCGTGGTGTAGTTGGTT
t0040244	24	34	TGGTAGAGCATTTGACTGCAGATC
t0040581	22	34	GGTGTCGTGGGGTAGTTGGTTA
t0041042	22	33	GTCGATATGTCCGAGTGGTTAA
t0041553	22	33	GGGGATATGGCGAAATTGGTAG
t0041586	23	33	GGGGATGTAGCTCAAACGGTAGT
t0042005	21	32	GGTGATGTAGCTCAGATGGTA
t0042117	21	32	CCGACCTTAGCTCAGTTGGCA
t0042133	23	32	GTCGTTGTAGTATAGTGGTGAGT
t0042261	20	32	GACCGCATAGCGCAGTGGAT
t0042283	24	32	CGCGGGGTAGAGCAGTTTGGTAGC
t0042548	21	32	GGGGATGTAGCTCAGATGGCA
t0043314	25	31	GTCGTTGTAGTATAGTGGTAAGTAT
t0043465	21	31	CCGACCTTAGCTCAGTTGGTA
t0044564	25	31	TGGTAGAGCATTTGACTGCAGATCA
t0044922	24	30	GGGGATGTAGCTCAAATGGTAGAA
t0045790	28	30	GGGATTGTAGTTCAATCGGTCAGAGCAC
t0045832	22	30	GCACCAGTGGTCTAGTGGTAGA
t0046434	20	29	GGTTCTATGGTGTAGTGGTT
t0046526	20	29	TGGGGCGTGGCCAAGCGGTA
t0046793	22	29	GTGGATGTAGCTCAGATGGTAG
t0046987	22	29	GTCGTTGTAGTATAGTGGTGAG
t0047162	27	29	GGTAGAGCTGAGGACTGTAGATCCTTA
t0047163	22	29	CGCGGGGTAGAGCAGTTTGGTA
t0048237	20	28	GCGCCTGTAGCTCAGTGGAT
t0048869	21	28	GGGGATGTAGCTCAGGTGGTA
t0049280	21	28	GGCGGATGTAGCCAAGTGGAT
t0049358	24	28	GTCTGGGTGGTGTAGTCGGTTATC
t0049473	19	27	TGGGATGTAGCTCAGATGG
t0049595	24	27	TCAGTGGTAGAGCATTTGACTGCA
t0049600	23	27	GTGGACGTGCCGGAGTGGTTATC
t0049656	19	27	TGGTTAGGATACTCGGCTC

t0049729	20	27	GGTGATGTAGCTCAGATGGT
t0049759	21	27	GCGGGTGTAGTTTAGTGGTAA
t0050303	25	27	GGGATTGTAGTTCAATCGGTCAGAG
t0050380	24	27	TCAGCCGGTAGAGCGCATGGCTTT
t0050956	20	27	CGCGGGGTGGAGCAGTTCGG
t0051010	22	27	GGGGATGTAGCTCATATGGTAG
t0051096	22	27	ATTTGAAATCTGTTGGGCTTCG
t0052330	23	26	GTGGATGTAGCTCAGATGGTAGA
t0052476	23	26	TGGGATGTAGCTCAGATGGTAGA
t0052494	20	26	GGGGATGTAGCTCATATGGT
t0052616	25	26	CGCGGGGTAGAGCAGTTTGGTAGCT
t0052745	22	26	CCGACCTTAGCTCAGTTGGTAG
t0052812	23	26	CAGTGGTAGAGCATTTGACTGCA
t0052948	20	26	GTGGCTGTAGTTTAGTGGTT
t0053188	25	25	TCCATTGTCGTCTAGTCCGGTTAGG
t0053568	21	25	GGGATTGTAGTTCAATCGGAA
t0053694	24	25	TCCGTTGTAGTCTAGTTGGTTAGG
t0053708	23	25	CTAGTTGGTTAGGATACTCGGCT
t0053827	21	25	GTGGCTGTAGTTTAGTGGTTA
t0053887	21	25	GGGGATGTAGCTCAGATGGTC
t0053889	20	25	GGGGATGTAGTTCAGATGGT
t0053968	22	25	GGGATGTAGCTCAGATGGTAGA
t0054233	24	25	GGGGATGTAGCTCAGATGGTAGTT
t0054345	19	25	CTAGCGGTTAGGACATTGG
t0054608	26	25	GCGTTTGTAGTCCAACGGTTAGGATA
t0054817	20	25	GGGGCTGTAGCTCAGATGGG
t0055012	19	25	GCGGATATGGTCGAATGGA
t0055569	26	24	GGGGATGTAGCTCAGATGGTAGATTT
t0055868	19	24	GCGGGTGTAGTTTAGTGGT
t0055945	25	24	GTCTGGGTTGTGTAGTTGGTTATCA
t0056917	23	24	TGTGTCGTGGTGTAGTTGGTTAT
t0056940	25	24	GGGGGTGTAGCTCATATGGTAGAGC
t0057052	25	24	GTCGTTGTAGTATAGTGGTGAGTAT
t0057076	19	24	GCGGAAGTAGTTCAGTGGT
t0057172	23	24	AGATTTGAAATCTGTTGGGCTTC
t0057596	22	23	GCGTTTGTAGTCCAACGGTTAG
t0057696	23	23	GTTGTCGTGGTGTAGTTGGTTAT
t0057754	19	23	TGGGGCGTGGCCAAGCGGT
t0057819	23	23	TCCGTTGTAGTCTAGTTGGTTAG
t0058170	29	23	GGGATTGTAGTTCAATCGGTCAGAGCACC
t0058249	19	23	AGGGATATAACTCAGCGGT
t0058326	18	23	GCGGATATGGTCGAATGG
t0058405	21	23	GGGGATGTAGCTCAGCTGGTA
t0058457	18	23	GTCGTTGTAGTATAGTGG
t0059083	23	23	AGTGGTAGAGCATTTGACTGCAG
t0059748	29	23	GGGGCTGTAGCTCAGATGGGAGAGCGCCG
t0060080	19	22	GACAGTTTGGCCGAGTGGT
t0060492	24	22	GGGGATGTAGCTCAAACGGTAGTT
t0061131	20	22	GGGGATATGGCGAAATTGGT
t0061394	22	22	GTGTCGTGGTGTAGTTGGTTAT
t0061636	24	22	GTCGTTGTAGTATAGTGGTGAGTA
t0061845	26	22	GTGGTAGAGCATTTGACTGCAGATCA
t0062123	20	22	GGTCCCGTAGCTCAGTTGGT
t0062321	21	22	AGTCCCGTAGCTCAGTTGGTT
t0062339	21	22	GGGGATGTAGCTCAGAGGTAG
t0062898	19	21	GTGGCTGTAGTTTAGTGGT
t0062965	20	21	GGGGGTGTAGCTCAGATGGT
t0063040	21	21	GGGGGTGTAGCTCATATGGGA
t0063141	23	21	GGGGATGTAGCTCAGATGGCAGA
t0063503	22	21	GGGGATGTAGCTCAGAGGTAGA
t0064094	27	21	GTGGTAGAGCATTTGACTGCAGATCAA
t0064399	24	21	GCGGATATGGTCGAATGGTAAAAT
t0065238	24	21	GCGGGGATAGCTCAGTTGGGAGAG

t0065294	26	21	GGGCCTGTAGCTCAGAGGATTAGAGC
t0065301	21	21	AGGGATGTAGCGCAGCTTGGT
t0065351	18	21	GGTGGTGTAGTTGGTTAT
t0065511	26	21	GGTGTCGTCGTGTAGTTGGTTATCAC
t0065714	23	20	GGGGATGTAGCTCAGATGGAAGA
t0065722	22	20	GGGGGGGTAGCTCATATGGTAG
t0066298	20	20	GGCGGATGTGGCCAAGTGGA
t0066837	18	20	GGGATGTAGCTCAGATGG
t0066877	24	20	GCGTTTGTAGTCCAACGGTTAGGA
t0066899	21	20	GCCCGTCTAGCTCAGTTGGTA
t0068041	26	20	GGTTCTATGGTCTAGCGGTTAGGACA
t0068173	19	20	GGGGATGTAGCTCAGAGGT
t0068349	27	20	AGGACATTGGACTCTGAATCCAGTAAC
t0068592	22	20	GTCTGGGGGGTGTAGTTGGTTA
t0068868	21	19	GGGGATGTAGCTCAAATGGGA
t0069202	25	19	ACGGACTGTAAATTCGTTGACGATA
t0069534	19	19	GGTTCTATGGTGTAGTGGT
t0069603	23	19	CCGACCTTAGCTCAGTTGGTAGA
t0070032	26	19	GGGATTGTAGTTCAATCGGTCAGAGC
t0070245	21	19	GGTGTCGTGGTGTAGTTGGGT
t0070462	23	19	GGGGGGGTAGCTCATATGGTAGA
t0070510	20	19	GGGGATGTAGCTCAGATGTT
t0070600	22	19	AGTTGGTTAGGATACTCGGCTC
t0070797	22	19	TAGTTGGTTAGGATACTCGGCT
t0071062	18	19	TGGTTAGGATACTCGGCT
t0071100	23	19	GGGGATGTAGCTCAGATGGTAGC
t0071226	23	19	GGGGATATGGCGAAATTGGTAGA
t0071332	23	19	AGCGGGGGAGAGGAATTGGTCAA
t0071347	24	19	AGATTTGAAATCTGTTGGGCTTCG
t0071500	28	19	TGGTAGAGCTGAGGACTGTAGATCCTTA
t0071688	19	19	CCGACCTTAGCTCAGTTGG
t0072082	20	19	GCGGATGTAGCTCAGATGGT
t0072249	24	19	GTCTGGGTTGTGTAGTTGGTTATC
t0073450	21	18	GGGGATATAGCTCAGTTGGTA
t0073784	22	18	TGGTAGAGCATTTGACTGCAGA
t0074090	26	18	AGCGGGGTAGAGGAATTGGTCAACTC
t0074114	21	18	AGCGGGGTAGAGGAATTGGTT
t0074286	21	18	TGGGGTGTAGCTCATATGGTA
t0074604	25	18	GGGGATGTAGCTCAGATGGTAGAAA
t0074686	22	18	GGGGATGGAGCTCAGATGGTAG
t0074867	24	18	GGGGTTGTAGCTCAAATGGTAGAA
t0074936	20	18	GTGGCTGTAGTTTAGTGGTA
t0075223	28	18	GGGCCTGTAGCTCAGAGGATTAGAGCAC
t0075328	20	18	AGCAGAAGGCCGTAGGTTCG
t0075677	22	18	GGTGATGTAGCTCAGATGGTAG
t0075996	24	18	TCTAGTTGGTTAGGATACTCGGCT
t0076235	27	18	AGCGGGGTAGAGGAATTGGTCAACTCA
t0076612	20	18	GGGGACTTAGCTTAGTTGGT
t0076818	18	17	CGAAAGGGCGTGGGTTCA
t0076941	19	17	GGGGATGTAGCTCATGGTA
t0077667	20	17	GGAGAGATGGCTGAGTGGAC
t0077696	24	17	AGGAAGCAGTGCTAGAGCATCTCG
t0077865	24	17	GCACCAGTGGTCTAGTGGTAGAAT
t0078138	23	17	GGCGGATGTGGCCAAGTGGATCA
t0078273	20	17	GGCGGATGTAGCCAAGTGGA
t0078288	21	17	GTGGCTGTAGTTTAGTGGTAA
t0078437	19	17	GTTGAGATGGCCGAGTTGG
t0078685	26	17	ACGGACTGTAAATTCGTTGACGATAT
t0078729	25	17	GAGCGGAGGACTGTAGATCCTTAGG
t0079454	23	17	ATATTTGAACCCACAACCTTGAG
t0080041	24	17	AGATTGAGGTTCTGGTCCGAAAGG
t0080233	30	17	GGGCCTGTAGCTCAGAGGATTAGAGCACGT
t0081593	18	16	GTCTGGTGTAGTTGGTTA

t0081672	23	16 GGGATTGTAGTTCAATCGGTCAG
t0081849	25	16 TCCGTTGTAGTCTAGTTGGTTAGGA
t0082331	22	16 TAAGCAGAAGGCCGTAGGTTCG
t0082369	20	16 GGGGCTGTAGCTCAGATGGT
t0082396	25	16 GGCGGATGTAGCCAAGTGGATCAAG
t0082607	21	16 GGGATTGTAGTTCAATCGGTA
t0082621	26	16 TGGTAGAATAGTACCCTGCCACGGTA
t0083057	23	16 ATTTGAAATCTGTTGGGCTTCGC
t0083350	20	16 TCAGCCGGTAGAGCGCATGG
t0083606	25	16 AGTGGTAGAGCATTTGACTGCAGAT
t0083618	20	16 GGTGGCTGTAGTTTAGTGGA
t0084053	21	16 GGTTAGGATACTCGGCTCTCA
t0084159	22	16 GCGGAAGTAGTTCAGTGGTAGA
t0084191	20	16 GGGGATGTATCTCAGATGGT
t0085217	19	16 CGAAAGGGCGTGGGTTCAT
t0085527	22	16 GGGGATGTAGCTCAAATGGGAG
t0086002	22	16 GGGGATGTAGCTCAGATGGTCG
t0086815	23	15 GCGTTTGTAGTCCAACGGTTAGG
t0086878	22	15 GTGGTAGAGCATTTGACTGCAG
t0086900	20	15 GGGGATGAAGCTCAGATGGT
t0086911	25	15 GGGGTTGTAGCTCAAATGGTAGAGC
t0087042	24	15 CTGGACTTGAACCAGAGACCTCGC
t0087197	22	15 GGGGATGTAGCTCAAATGGTAA
t0087263	20	15 GCGGATATGGTCGAATGGAA
t0087302	24	15 GTGGTAGAGCATTTGACTGCAGAT
t0087429	18	15 GGATGTAGCTCAGATGGT
t0087526	23	15 GGGGATGGAGCTCAGATGGTAGA
t0087585	23	15 GTCGATATGTCCGAGTGGTTAAG
t0088248	25	15 GTTGTCGTGGTGTAGTTGGTTATCA
t0088344	24	15 GGCGGATGTAGCCAAGTGGATCAA
t0088383	20	15 GGACTAGACGACAATGGAAA
t0089166	22	15 GTCAGGATGGCCGAGTGGTCTA
t0089202	20	15 GTTAGGATACTCGGCTCTCA
t0090224	23	15 GATTTGAAATCTGTTGGGCTTCG
t0091157	24	15 GGTAGAGCATTTGACTGCAGATCA
t0091414	22	15 GGGGACTTAGCTTAGTTGGTAG
t0093116	23	14 GGTGTCGTGGGGTAGTTGGTTAT
t0093152	21	14 GGGGGTGTAGCTCAAATGGTA
t0093364	21	14 GGTTCTATGGTGTAGTGGTTA
t0093427	20	14 TCAAGGCAGTGGATTGTGAA
t0093624	21	14 AGGGATGTAGCTCAGATGGTA
t0093730	24	14 AACCGGACTAGACGACAATGGAAA
t0093775	25	14 GCGGATATGGTCGAATGGTAAAATT
t0093982	21	14 TGGTAGAGCATTTGACTGCAG
t0094521	25	14 GGGGATGTAGCTCAGATGGTAGATC
t0095038	22	14 ACATTGGACTCTGAATCCAGTA
t0095588	30	14 GGGGCTGTAGCTCAGCTGGGAGAGCACCTG
t0096015	21	14 GCGGATGTAGCTCAGATGGTA
t0096098	24	14 TGTGTCGTGGTGTAGTTGGTTATC
t0096185	20	14 GGGGATGTAGCTCAGATGGC
t0096539	18	14 AGGGATGTAGCGCAGCTT
t0096843	23	14 GGGGATGTAGCTCATATGGTAGA
t0097018	23	14 TGGTAGAGCTGAGGACTGTAGAT
t0097867	20	14 AGGATACTCGGCTCTCACCC
t0098637	22	14 GGGGATGTAGCTCAGATGGCAG
t0098784	24	14 GTGGATGTAGCTCAGATGGTAGAG
t0098883	27	14 AGTGGTAGAGCATTTGACTGCAGATCA
t0099218	20	13 CGGGATGTAGCTCAGATGGT
t0099378	20	13 GCACCAGTTGTCTAGTGGTA
t0099495	18	13 AGGGATATAACTCAGCGG
t0099863	23	13 GGTGGCTGTAATTTAGTGGTTAG
t0100102	25	13 GGAGAGATGGCTGAGTGGACTAAAG
t0100141	22	13 GGGGATGTAGCTCAGATGGAAG

t0100967	23	13	TTGGTAGAGCTGAGGACTGTAGA
t0100973	19	13	GTCTGGTGTAGTTGGTTAT
t0102038	23	13	GGCGGATGTAGCCAAGTGGATCA
t0102761	22	13	GACCGCATAGCGCAGTGGATTA
t0103197	24	13	AGATGGCTGAGTGGACTAAAGCGG
t0103404	28	13	GGGGCTGTAGCTCAGCTGGGAGAGCACC
t0103489	21	13	GGGATTGTAGTTCAATCGGTT
t0103897	21	13	GTTGGTTAGGATACTCGGCTC
t0104470	23	13	GTCTGGGTCGTGTAGTTGGTTAT
t0104477	22	13	AGGGATATAACTCAGCGGTAGA
t0105179	20	13	GGCGATGTAGCTCAGATGGT
t0105445	21	13	GTTGGCTGTAGTTTAGTGGTT
t0105868	24	13	CAGTGGTAGAGCATTTGACTGCAG
t0106253	25	13	GGGGATGTAGCTCAAATGGTAGAGC
t0106303	21	13	GTTAGGATACTCGGCTCTCAC
t0107369	24	12	GTCGATATGTCCGAGTGGTTAAGG
t0108591	28	12	GGGTCTGTAGCTCAGTCGGTTAGAGCAC
t0108895	20	12	CGAAAGGGCGTGGGTTCAGA
t0109228	19	12	GCGGATATGGTCGAATGGT
t0109909	25	12	TGGTAGAGCATTTGACTGCACATCA
t0110370	22	12	GGGGATGTAGCTCAGATGGTGA
t0111346	22	12	CAGTTGGTAGAGCTGAGGACTG
t0112101	22	12	GATTTGAAATCTGTTGGGCTTC
t0112668	24	12	GGGGATATGGCGAAATTGGTAGAC
t0112708	22	12	GGGTATGTAGCTCAGATGGTAG
t0113319	26	12	TCAGTGGTAGAGCATTTGACTGCAGA
t0113333	24	12	TGGGATGTAGCTCAGATGGTAGAG
t0113734	21	12	GCGGGTGTAGCTCAGTCGGTT
t0114662	24	12	GTGGTCGTGCCGGAGTGGTTATCG
t0114754	18	12	GTGGCTGTAGTTTAGTGG
t0115003	21	12	GCGGATGTGGCCAAGTGGATC
t0115372	23	12	TGGTAGAGCATTTGACTGCAGAT
t0115458	23	12	AGGGATATAACTCAGCGGTAGAG
t0115547	19	12	GTGATGTAGCTCAGATGGT
t0116201	29	12	GGGGCTGTAGCTCAGATGGGAGAGCGCTG
t0116653	24	11	AGTGGTAGAGCATTTGACTGCAGA
t0117222	28	11	TAGGACATTGGACTCTGAATCCAGTAAC
t0117666	20	11	GTCAGGATGGCCGAGTGGTC
t0117711	19	11	GGGGATGTAGCTCAGCTGG
t0118024	26	11	GGAGAGATGGCTGAGTGGACTAAAGC
t0118293	22	11	AGCGGGGGAGAGGAATTGGTCA
t0118521	20	11	CCGACCTTAGCTCAGTTGGT
t0118770	21	11	GCGCTCTTAGTTCAGTTCGGA
t0119422	20	11	CAGAGGATTAGAGCACGTGG
t0119785	22	11	GGGGGTGTAGCTCAAATGGTAG
t0120164	22	11	GGGGATGTAGATCAGATGGTAG
t0120441	22	11	GGCGGATGTAGCCAAGTGGATC
t0120922	24	11	GGGGATGTAGCTCAGATGGGAGAG
t0121187	19	11	GGATGTAGCTCAGATGGTA
t0121533	25	11	GGGGGTGTAGCTCATATGGTAGAAA
t0121606	23	11	GGGGATGTAGCTCAGATGGTAGG
t0122254	20	11	GGGGATATAGCTCAGTTGGT
t0122302	20	11	GCGGGTGTAGCTCAGTCGGT
t0123040	26	11	GGCGGATGTAGCCAAGTGGATCAAGG
t0123870	21	11	TCGAATCCTGCTGTCGACGCC
t0124228	23	11	TAGTTGGTTAGGATACTCGGCTC
t0124511	23	11	AGTTGGTTAGGATACTCGGCTCT
t0124636	23	11	ACTTCTAATCAGGCGATTGTGGG
t0124698	21	11	GGGGATGTAGCTCAGATGGTG
t0125247	22	11	GGGGTGTAGCTCATATGGTAGA
t0125308	21	11	GGGGACTTAGCTTAGTTGGTA
t0125437	22	11	TTTGAAATCTGTTGGGCTTCGC
t0125685	21	11	GCGCCTGTAGCTCAGTGGAGA

t0126299	28	11	ACGGACTGTAAATTCGTTGACGATATGT
t0126302	23	11	TCCGTTGTAGTCTAGGTGGTTAG
t0126462	20	11	CCGACCTTAGCTCAGTTGGC
t0126621	20	11	GTTGTCGTGGTGTAGTTGGT
t0126647	22	11	GGCGTCGTGGTGTAGTTGGTTA
t0127161	23	11	CGCGGGGTGGAGCAGTTCGGTAG
t0127305	21	11	TCGTGAGAGGGCGTGGGTTCA
t0127440	20	11	GTGATGTAGCTCAGATGGTA
t0127629	22	11	TTCGAATCCTGCTGTCGACGCC
t0128815	24	10	GGGGATGTAGCTCAAATGGTAAAA
t0128928	23	10	GTCTGGGTAGTGTAGTTGGTTAT
t0129070	20	10	GGGATTGTAGTTCAATCGGG
t0129405	21	10	GGGGGTGTAGCTCAGATGGTA
t0129483	26	10	GGGACTGTAGTTCAATTGGTTAGAGC
t0129491	21	10	TTGGTAGACGCTACGGACTTA
t0129642	22	10	CGTGTCGTGGTGTAGTTGGTTA
t0129766	21	10	TGGGATGTAGCTCAAATGGTA
t0129916	21	10	TCAGAGGATTAGAGCACGTGG
t0130321	24	10	GTGTCGTGGTGTAGTTGGTTATCA
t0130419	19	10	GGGGATGTAGCTCAGATGT
t0131373	19	10	GTCGATATGTCCGAGTGGT
t0131668	20	10	GGGGATGTAGCTCAGAGGGT
t0132493	19	10	ACACTGAAGGTCTCCGGTT
t0132740	20	10	GGGTCCATAGCTCAGTGGTA
t0133473	18	10	GGGGGTGTAGCTCATATG
t0133480	22	10	GGCGGATGTGGCCAAGTGGATC
t0133579	22	10	GGGGATGTAGCTCAGATGTTAG
t0133710	23	10	GGGGATGTAGCTCAGAGGTAGAG
t0135440	21	10	GGGGATGTAGTTCAGATGGTA
t0136003	26	10	TGGTAGAGCATTTGACTGCACATCAA
t0136069	21	10	CGGGATGTAGCTCAGATGGTA
t0136202	23	10	TGGGGTGTAGCTCATATGGTAGA
t0136273	20	10	TGTGTCGTGGTGTAGTTGGT
t0136330	21	10	GGAGAGATGGCCGAGTGGTTG
t0136519	20	10	TCTAGCGGTTAGGACATTGG
t0136522	26	10	GGGGATGTAGCTCAGATGGTAGAGCT
t0136678	19	10	GGTGGTGTAGTTGGTTATC
t0137114	19	10	GGGGATGTAGTTCAGATGG
t0137221	22	10	GGTTCTATGGTCTAGCGGTTAG
t0137321	25	10	GGTAGAGCATTTGACTGCAGATCAA
t0137356	23	10	CGGGATGTAGCTCAGATGGTAGA
t0137601	21	10	AGCTGGTTAGGATACTCGGCT
t0137813	23	10	GTGTCGTGGTGTAGTTGGTTATC
t0137963	21	10	GGGGATGAAGCTCAGATGGTA
t0138086	22	10	GGGGATGTAGCTCAGCTGGTAG
t0138382	22	10	TCAGTTGGTAGAGCTGAGGACT
t0138506	22	10	GCGGGTGTAGTTTAGTGGTAAA
t0138837	22	10	GGGGGTGTAGCTCATATGGTAA
t0139096	20	10	GCGGATGTGGCCAAGTGGAT
t0139599	23	10	GGGTATGTAGCTCAGATGGTAGA
t0140019	22	10	GCGCCTGTAGCTCAGTGGATAG
t0140295	24	10	GGGGATGTAGCTCATATGGTAGAG
t0140911	27	10	TGGTAGAGCATTTGACTGCAGATCAAG
t0141304	23	10	GGGGATGTAGATCAGATGGTAGA
