# Supplementary material for: Transfer RNA Derived Small RNAs Targeting Defense Responsive Genes Are Induced during Phytophthora capsici Infection in Black Pepper (Piper nigrum L.)
Source: Front Plant Sci. 2016 Jun 1;7:767. doi: 10.3389/fpls.2016.00767 (PMC4887504; doi:10.3389/fpls.2016.00767)
Supplement: Supplementary file 3 [file DataSheet2.PDF]

Transfer RNA derived small RNAs targeting defence responsive genes are induced during *Phytophthora capsici* infection in black pepper (*Piper nigrum* L.)

Supplementary Data 2

TRNA mapped small RNAs from *Phytophthora capsici* infected leaf library (Pn IL) of black pepper

Seq. ID	Length	Read Count	Sequence
t0000004	20	83347	GGGGATGTAGCTCAGATGGT
t0000005	21	80049	GGGGATGTAGCTCAGATGGTA
t0000008	22	49518	GGGGATGTAGCTCAGATGGTAG
t0000010	23	38843	GGGGATGTAGCTCAGATGGTAGA
t0000013	24	32159	GGGGATGTAGCTCAGATGGTAGAG
t0000030	22	17851	GGGGATGTAGCTCAAACGGTAG
t0000031	19	16768	GGGGATGTAGCTCAGATGG
t0000037	22	13680	GGTGTCGTGGTGTAGTTGGTTA
t0000040	23	12841	GGGGATGTAGCTCAAACGGTAGA
t0000043	21	11613	GGTGTCGTGGTGTAGTTGGTT
t0000046	24	10512	GGGGATGTAGCTCAAACGGTAGAG
t0000055	23	8821	GGTGTCGTGGTGTAGTTGGTTAT
t0000062	24	7966	GGGGGTGTAGCTCATATGGTAGAG
t0000073	25	7226	GGTGTCGTGGTGTAGTTGGTTATCA
t0000082	23	6197	GGGGGTGTAGCTCATATGGTAGA
t0000087	24	5607	GGTGTCGTGGTGTAGTTGGTTATC
t0000090	22	5512	GGGGGTGTAGCTCATATGGTAG
t0000091	21	5465	GGGGATGTAGCTCAGATGGTC
t0000098	22	5116	GGGGTTGTAGCTCAAATGGTAG
t0000101	24	4969	GGGGTTGTAGCTCAAATGGTAGAG
t0000103	21	4946	GGGGGTGTAGCTCATATGGTA
t0000106	20	4921	GCGTTTGTAGTCCAACGGTT
t0000111	23	4703	GGGGTTGTAGCTCAAATGGTAGA
t0000113	24	4643	GGGGATGTAGCTCAAATGGTAGAG
t0000114	25	4610	GGGGATGTAGCTCAGATGGTAGAGC
t0000115	22	4406	GGGGATGTAGCTCAGATGGTCG
t0000117	23	4354	GGGGATGTAGCTCAAATGGTAGA
t0000140	22	3679	GGGGATGTAGCTCAAATGGTAG
t0000141	22	3635	GTCTGGGTGGTGTAGTTGGTTA
t0000145	21	3505	GGGGATGTAGCTCAAATGGTA
t0000161	20	3203	GGTGTCGTGGTGTAGTTGGT
t0000171	21	2913	GCGTTTGTAGTCCAACGGTTA
t0000185	21	2643	GTCTGGGTGGTGTAGTTGGTT
t0000188	21	2562	GGGATTGTAGTTCAATCGGTC
t0000190	25	2533	GGTGTCGTCGTGTAGTTGGTTATCA
t0000192	22	2484	GGTGGCTGTAGTTTAGTGGTTA
t0000197	21	2416	GGTGGCTGTAGTTTAGTGGTA
t0000203	19	2340	GCGTTTGTAGTCCAACGGT
t0000204	20	2325	GGTGGCTGTAGTTTAGTGGT
t0000206	25	2286	GGGGATGTAGCTCAAACGGTAGAGC
t0000224	22	2108	GGTGTCGTCGTGTAGTTGGTTA
t0000243	24	1931	GGTGGCTGTAGTTTAGTGGTTAGA
t0000245	21	1915	GGTGGCTGTAGTTTAGTGGTT
t0000248	20	1864	GCGTTTGTAGTCCAACGGTA
t0000251	24	1842	GGTGTCGTCGTGTAGTTGGTTATC

t0000255	23	1783	GGTGGCTGTAGTTTAGTGGTTAG
t0000276	25	1670	GGGGGTGTAGCTCATATGGTAGAGC
t0000328	26	1500	GGTGGCTGTAGTTTAGTGGTTAGAAT
t0000345	21	1456	AGCGGGGTAGAGGAATTGGTC
t0000366	25	1406	GGTGGCTGTAGTTTAGTGGTTAGAA
t0000394	20	1326	GTCTGGGTGGTGTAGTTGGT
t0000404	20	1310	GGGGGTGTAGCTCATATGGT
t0000422	23	1266	GGTGTCGTCGTGTAGTTGGTTAT
t0000463	22	1175	GTCTGGGTGGTGTAGTCGGTTA
t0000478	22	1153	AGCGGGGTAGAGGAATTGGTCA
t0000481	23	1150	GTCTGGGTGGTGTAGTTGGTTAT
t0000486	22	1139	GGTGTCGTTGTGTAGTTGGTTA
t0000501	20	1119	GGGGATGTAGCTCAAATGGT
t0000527	21	1081	GGAGATGTAGCTCAGATGGTA
t0000528	23	1080	AGCGGGGTAGAGGAATTGGTCAA
t0000590	20	990	GGAGATGTAGCTCAGATGGT
t0000618	20	960	AGCGGGGTAGAGGAATTGGT
t0000692	22	883	TCCATTGTCGTCTAGTCCGGTT
t0000699	22	876	GGGATTGTAGTTCAATCGGTCA
t0000736	21	845	TCCATTGTCGTCTAGTCCGGT
t0000757	25	833	AGCGGGGTAGAGGAATTGGTCAACT
t0000767	20	826	GCGGGGATAGCTCAGTTGGG
t0000784	25	815	GGTGTCGTTGTGTAGTTGGTTATCA
t0000805	19	797	GGTGGCTGTAGTTTAGTGG
t0000852	19	768	GGTGTCGTGGTGTAGTTGG
t0000857	22	766	GTCTGGGTGGTGTAGTTGGTCA
t0000931	21	720	GCGTTTGTAGTCCAACGGTAA
t0000938	23	716	GGTGTCGTTGTGTAGTTGGTTAT
t0001021	22	676	GGAGATGTAGCTCAGATGGTAG
t0001143	21	620	GTCTGGGTGGTGTAGTTGGTC
t0001184	26	606	GGTGTCGTGGTGTAGTTGGTTATCAC
t0001187	22	605	GGAGAGATGGCTGAGTGGACTA
t0001216	24	593	GGTGTCGTTGTGTAGTTGGTTATC
t0001252	22	585	GTGGACGTGCCGGAGTGGTTAT
t0001261	19	582	GCGGGGATAGCTCAGTTGG
t0001296	25	570	GGGGATGTAGCTCAAATGGTAGAGC
t0001329	24	558	GTCTGGGTGGTGTAGTTGGTTATC
t0001374	21	541	GGAGAGATGGCTGAGTGGACT
t0001390	27	536	GGGCCTGTAGCTCAGAGGATTAGAGCA
t0001393	25	535	GGTGGCTGTAGTTTAGTGGTAAGAA
t0001440	25	523	GGGGTTGTAGCTCAAATGGTAGAGC
t0001459	24	518	AGCGGGGTAGAGGAATTGGTCAAC
t0001465	25	517	GTCTGGGTGGTGTAGTTGGTTATCA
t0001528	23	501	GGAGATGTAGCTCAGATGGTAGA
t0001564	22	491	GGGGGTGTAGCTCATATGGTCG
t0001578	23	487	TCCATTGTCGTCTAGTCCGGTTA
t0001636	22	473	GCGGGGATAGCTCAGTTGGGAG
t0001686	22	463	GTGGTCGTGCCGGAGTGGTTAT
t0001709	23	457	GCGGGGATAGCTCAGTTGGGAGA
t0001761	20	443	AGGGATATAACTCAGCGGTA

t0001776	23	441	GGAGAGATGGCTGAGTGGACTAA
t0001784	24	439	GGAGATGTAGCTCAGATGGTAGAG
t0001790	20	438	GCGTTTGTAGTCCAACGGTC
t0001897	22	419	GTCTGGGTTGTGTAGTTGGTTA
t0001982	19	406	AGCGGGGTAGAGGAATTGG
t0002010	18	401	GTTGAGATGGCCGAGTTG
t0002126	21	387	GGTGATGTAGCTCAGATGGTA
t0002151	20	383	GGGATTGTAGTTCAATCGGT
t0002257	29	370	GGGGCTGTAGCTCAGCTGGGAGAGCACCT
t0002311	25	363	GGGGATGTAGCTCAGATGGTCGAGC
t0002369	20	355	GGTGATGTAGCTCAGATGGT
t0002424	21	347	GGGGGTGTAGCTCATATGGTC
t0002443	21	345	GCGGGGATAGCTCAGTTGGGA
t0002477	21	341	CAAGCAAGGACTCTACCACGC
t0002536	28	335	GGGCCTGTAGCTCAGAGGATTAGAGCAC
t0002555	24	333	GGAGAGATGGCTGAGTGGACTAAA
t0002574	23	331	GTCTGGGTGGTGTAGTCGGTTAT
t0002750	22	314	GGGGATGTAGCTCAAATGGTCG
t0002892	24	301	GCGGGGATAGCTCAGTTGGGAGAG
t0002914	21	299	GGGGATATGGCGAAATTGGTA
t0002921	24	299	GGGCCTGTAGCTCAGAGGATTAGA
t0002933	21	298	GCGTTTGTAGTCCAACGGTCA
t0003069	25	288	GGGCCTGTAGCTCAGAGGATTAGAG
t0003107	20	284	GTTGAGATGGCCGAGTTGGT
t0003167	19	280	GTCTGGGTGGTGTAGTTGG
t0003253	26	273	GGGCCTGTAGCTCAGAGGATTAGAGC
t0003255	23	273	GGGCCTGTAGCTCAGAGGATTAG
t0003325	21	267	GTCGTTGTAGTATAGTGGTAA
t0003449	20	258	GCACCAGTGGTCTAGTGGTA
t0003471	21	256	GCGGATATGGTCGAATGGTAA
t0003485	29	256	GGGGCTGTAGCTCAGATGGGAGAGCGCCG
t0003587	26	249	GGGGATGTAGCTCAAACGGTAGAGCT
t0003625	20	247	GTCGTTGTAGTATAGTGGTA
t0003632	26	247	GGGGATGTAGCTCAGATGGTAGAGCT
t0003672	21	245	GGGGATGTAGCTCAAATGGTC
t0003930	20	231	GGGGATATGGCGAAATTGGT
t0003988	22	227	TCCGTTGTAGTCTAGTTGGTTA
t0004105	25	221	GGGGATATGGCGAAATTGGTAGACG
t0004191	28	217	GGGGCTGTAGCTCAGATGGGAGAGCGCT
t0004197	19	216	GGGATTGTAGTTCAATCGG
t0004235	27	215	GGGATTGTAGTTCAATCGGTCAGAGCA
t0004239	25	215	GTCTGGGTGGTGTAGTTGGTCATCA
t0004276	22	213	AGGGATGTAGCGCAGCTTGGTA
t0004296	25	212	GGAGAGATGGCTGAGTGGACTAAAG
t0004303	20	212	GCGGATATGGTCGAATGGTA
t0004331	21	211	GCGTTTGTAGTCCAACGGTTC
t0004414	22	207	GGGGATGTAGCTCAGATGGTAT
t0004468	22	205	GGTGATGTAGCTCAGATGGTAG
t0004877	20	191	GTCGATATGTCCGAGTGGTT
t0004940	19	189	GGAGATGTAGCTCAGATGG

t0005004	23	186	GTGGACGTGCCGGAGTGGTTATC
t0005036	20	185	GGAGAGATGGCTGAGTGGAC
t0005140	23	182	GGTGATGTAGCTCAGATGGTAGA
t0005170	25	180	GTCGTTGTAGTATAGTGGTAAGTAT
t0005186	26	180	GCGTTTGTAGTCCAACGGTTAGGATA
t0005187	24	180	AGGGATGTAGCGCAGCTTGGTAGC
t0005213	22	179	GGTGTCGTAGTGTAGTTGGTTA
t0005299	26	177	AGCGGGGTAGAGGAATTGGTCAACTC
t0005328	24	176	TCCATTGTCGTCTAGTCCGGTTAG
t0005384	19	174	GCACCAGTGGTCTAGTGGT
t0005654	26	166	GGAGAGATGGCTGAGTGGACTAAAGC
t0005708	21	165	GGTGGCTGTAGTTTAGTGGTC
t0005762	26	163	GGCGGATGTAGCCAAGTGGATCAAGG
t0005978	20	158	TCCATTGTCGTCTAGTCCGG
t0006060	23	156	GTGGTCGTGCCGGAGTGGTTATC
t0006180	20	153	GGCGATGTAGCTCAGATGGT
t0006289	27	151	AGCGGGGTAGAGGAATTGGTCAACTCA
t0006418	18	148	GCGGGGATAGCTCAGTTG
t0006425	21	148	GTGGTAGAGCATTTGACTGCA
t0006440	21	147	GGCGATGTAGCTCAGATGGTA
t0006506	25	146	GGGGGTGTAGCTCATATGGTCGAGC
t0006573	24	145	GGTGATGTAGCTCAGATGGTAGAG
t0006593	22	144	GGGGATATGGCGAAATTGGTAG
t0006625	22	143	GTCTGGGTCGTGTAGTTGGTTA
t0006817	23	140	GCGCCTGTAGCTCAGTGGATAGA
t0006826	25	140	GTCGTTGTAGTATAGTGGTGAGTAT
t0006831	26	140	TGGTAGAGCATTTGACTGCAGATCAA
t0006911	27	138	GCGTTTGTAGTCCAACGGTTAGGATAA
t0007026	24	136	GTCGTTGTAGTATAGTGGTAAGTA
t0007050	19	136	GGGGGTGTAGCTCATATGG
t0007143	23	134	GTCTGGGTTGTGTAGTTGGTTAT
t0007161	20	134	GGGGATGTAGCTCAGATGGG
t0007176	25	133	TCCATTGTCGTCTAGTCCGGTTAGG
t0007221	23	133	GGGGATGTAGCTCAGATGGTATA
t0007318	21	131	GTCGTTGTAGTATAGTGGTGA
t0007343	20	131	GTCGTTGTAGTATAGTGGTG
t0007380	22	130	AGTGGTAGAGCATTTGACTGCA
t0007489	26	128	GTCGTTGTAGTATAGTGGTAAGTATT
t0007493	20	128	AAGCAAGGACTCTACCACGC
t0007541	27	127	GGTAGAGCTGAGGACTGTAGATCCTTA
t0007575	23	127	GTCGTTGTAGTATAGTGGTAAGT
t0007642	21	126	GGGGATGTAGCTCAGATGGTT
t0007701	25	125	CGCGGGGTAGAGCAGTTTGGTAGCT
t0007758	20	124	AGGGATGTAGCGCAGCTTGG
t0007954	27	121	TCCATTGTCGTCTAGTCCGGTTAGGAT
t0008049	21	120	GGGGATGTAGCTCAGATGGGA
t0008105	24	119	GGGGATATGGCGAAATTGGTAGAC
t0008107	22	119	GGGAATGTAGCTCAGATGGTAG
t0008145	25	119	GGTGTCGTAGTGTAGTTGGTTATCA
t0008191	24	118	GGGGATGTAGCTCAGATGGTAGAA

t0008408	23	115	GGGGATATGGCGAAATTGGTAGA
t0008694	26	112	GGTGTCGTCGTGTAGTTGGTTATCAC
t0008711	25	111	GGCGGATGTGGCCAAGTGGATCAAG
t0008723	23	111	AGGGATGTAGCGCAGCTTGGTAG
t0008727	20	111	GTCAAGATGGCCGAGTTGGT
t0008779	24	111	GTCTGGGTGGTGTAGTCGGTTATC
t0008865	23	110	GGTGTCGTAGTGTAGTTGGTTAT
t0008891	23	109	GGGAATGTAGCTCAGATGGTAGA
t0008902	22	109	TCCGTTGTAGTCTAGGTGGTTA
t0008949	27	109	TGGTAGAGCATTTGACTGCAGATCAAG
t0008956	24	108	GGTGTCGTAGTGTAGTTGGTTATC
t0008989	25	108	GGCGGATGTAGCCAAGTGGATCAAG
t0009035	20	108	TGGTAGAGCATTTGACTGCA
t0009144	20	106	GGTTCTATGGTGTAGTGGTT
t0009162	24	106	CGCGGGGTAGAGCAGTTTGGTAGC
t0009241	23	105	AGTGGTAGAGCATTTGACTGCAG
t0009262	23	105	GGCGGATGTGGCCAAGTGGATCA
t0009272	19	105	GTTGAGATGGCCGAGTTGG
t0009332	28	104	GGGATTGTAGTTCAATCGGTCAGAGCAC
t0009361	22	104	GGCGATGTAGCTCAGATGGTAG
t0009404	24	103	GTGGACGTGCCGGAGTGGTTATCG
t0009456	19	103	GGGGATGTAGCTCAAATGG
t0009510	22	102	GGTGTCGTGGTGTAGTTGGTTC
t0009595	24	102	GGGGATGTAGCTCAAACGGTAGAA
t0009597	21	101	GTCGATATGTCCGAGTGGTTA
t0009600	19	101	GTCAGGATGGCCGAGTGGT
t0009638	20	101	AGCAGAAGGCCGTAGGTTCG
t0009650	22	101	GTCGTTGTAGTATAGTGGTAAG
t0009694	25	101	GTCTGGGTGGTGTAGTCGGTTATCA
t0009721	22	100	CCGACCTTAGCTCAGTTGGCAG
t0009799	27	99	GTGGTAGAGCATTTGACTGCAGATCAA
t0009828	26	99	GCGGATATGGTCGAATGGTAAAATTT
t0009845	24	99	GGGGATGTAGCTCAGATGGTAGAT
t0010005	20	97	GTTGGTTAGGATACTCGGCT
t0010023	23	97	CGCGGGGTAGAGCAGTTTGGTAG
t0010038	20	97	TGGGGCGTGGCCAAGCGGTA
t0010097	19	96	GTCGTTGTAGTATAGTGGT
t0010119	20	96	GGGATGTAGCTCAGATGGTA
t0010142	25	96	GCGGATATGGTCGAATGGTAAAATT
t0010211	24	95	GGCGGATGTGGCCAAGTGGATCAA
t0010316	22	94	GGTAGAGCTGAGGACTGTAGAT
t0010450	23	93	GTCGTTGTAGTATAGTGGTGAGT
t0010469	24	93	GGGAATGTAGCTCAGATGGTAGAG
t0010506	26	93	GGGGATATGGCGAAATTGGTAGACGC
t0010539	23	92	GCGTTTGTAGTCCAACGGTTAGG
t0010665	22	91	GTGGTAGAGCATTTGACTGCAG
t0010672	21	91	TGGGATGTAGCTCAGATGGTA
t0010806	21	90	CTAGCGGTTAGGACATTGGAC
t0010875	24	90	GCGTTTGTAGTCCAACGGTTAGGA
t0010934	23	89	TCCGTTGTAGTCTAGTTGGTTAG

t0011090	20	88	TGGGATGTAGCTCAGATGGT
t0011140	22	88	GGCGGATGTGGCCAAGTGGATC
t0011216	28	87	TGGTAGAGCTGAGGACTGTAGATCCTTA
t0011219	24	87	AGATTGAGGTTCTGGTCCGAAAGG
t0011314	26	86	GGCGGATGTGGCCAAGTGGATCAAGG
t0011339	24	86	GTCGTTGTAGTATAGTGGTGAGTA
t0011368	28	86	AGCGGGGTAGAGGAATTGGTCAACTCAT
t0011390	20	86	GCTGGAATAGCTCAGTTGGT
t0011396	19	86	AGGGATATAACTCAGCGGT
t0011427	22	86	GGTGATGTAGCTCAAACGGTAG
t0011518	22	85	GTCTGGGTAGTGTAGTTGGTTA
t0011539	21	85	GGCGGATGTAGCCAAGTGGAT
t0011541	25	85	TGGTAGAGCATTTGACTGCAGATCA
t0011593	23	84	CCGACCTTAGCTCAGTTGGCAGA
t0012003	19	82	GGAGAGATGGCTGAGTGGA
t0012005	25	82	GGGGATGTAGCTCAGATGGTAAAAA
t0012147	19	81	GGGATGTAGCTCAGATGGT
t0012196	25	80	GCGCCTGTAGCTCAGTGGATAGAGC
t0012217	22	80	GGGGATGTAGCTCAGATGGTAA
t0012346	24	79	GCGGATATGGTCGAATGGTAAAAT
t0012545	21	78	TGGTAGAGCATTTGACTGCAG
t0012600	21	78	GTCAGGATGGCCGAGTGGTCT
t0012650	26	77	GTCGTTGTAGTATAGTGGTGAGTATT
t0012683	22	77	CGCGGGGTAGAGCAGTTTGGTA
t0012690	23	77	GGTGTCGTGGTGTAGTTGGTTCT
t0012707	21	77	GGGATGTAGCTCAGATGGTAG
t0012926	26	76	TCCATTGTCGTCTAGTCCGGTTAGGA
t0013004	25	75	GGGATTGTAGTTCAATCGGTCAGAG
t0013077	27	75	TGGGTTCGTGCCCCACGGTGGGCGCCA
t0013115	21	75	GCGAGAGGTACGGGGATCGTT
t0013121	25	75	GCGTTTGTAGTCCAACGGTTAGGAT
t0013123	19	75	GGGGATATGGCGAAATTGG
t0013264	24	74	GGCGGATGTAGCCAAGTGGATCAA
t0013331	27	74	GGGGATGTAGCTCAGATGGTAGAGCTC
t0013568	24	72	GGAGTTGTAGCTCAAATGGTAGAG
t0013578	19	72	GGTTCTATGGTGTAGTGGT
t0013642	28	72	GCGGACGTAGCTCAGTTGGTAGAGCGCA
t0013733	24	71	TGGTAGAGCATTTGACTGCAGATC
t0013834	22	71	TGGGATGTAGCTCAGATGGTAG
t0013837	25	71	GTCTGGGTTGTGTAGTTGGTTATCA
t0013877	20	71	GGTGGCTGTAGTTTAGTGGA
t0013895	27	71	AGTGGTAGAGCATTTGACTGCAGATCA
t0013920	22	70	GGAGTTGTAGCTCAAATGGTAG
t0014089	29	70	GGGCCTGTAGCTCAGAGGATTAGAGCACG
t0014292	22	69	GCGGATATGGTCGAATGGTAAA
t0014609	22	67	TAAGCAGAAGGCCGTAGGTTCG
t0014687	23	67	TCCGTTGTAGTCTAGGTGGTTAG
t0014699	23	67	AGGGATATAACTCAGCGGTAGAG
t0014747	22	66	GTCGTTGTAGTATAGTGGTGAG
t0014766	30	66	GGGGCTGTAGCTCAGATGGGAGAGCGCCGC

t0014886	29	66	GGGATTGTAGTTCAATCGGTCAGAGCACC
t0014910	22	66	CCGACCTTAGCTCAGTTGGTAG
t0014980	22	66	GTCAGGATGGCCGAGTGGTCTA
t0015011	28	65	GGTGTCGTGGTGTAGTTGGTTATCACTT
t0015124	27	65	GGGGATGTAGCTCAAACGGTAGAGCGC
t0015128	27	65	GCGGATATGGTCGAATGGTAAAATTTC
t0015442	22	63	GCGTTTGTAGTCCAACGGTTAG
t0015649	21	63	GGCGGATGTGGCCAAGTGGAA
t0015787	21	62	GTGGATGTAGCTCAGATGGTA
t0015898	19	62	TTGGTTAGGATACTCGGCT
t0015963	25	61	GGTGGCTGTAGTTTAGTGGTCAGAA
t0016011	24	61	GGAGATGTAGCTCAAATGGTAGAG
t0016165	29	61	GGGGCTGTAGCTCAGATGGGAGAGCGCTG
t0016286	28	60	GCGTTTGTAGTCCAACGGTTAGGATAAT
t0016292	23	60	GTCGATATGTCCGAGTGGTTAAG
t0016304	23	60	TGGGATGTAGCTCAGATGGTAGA
t0016338	21	60	AGGGATATAACTCAGCGGTAG
t0016417	19	60	GCGAGAGGTACGGGGATCG
t0016444	24	60	TCCGTTGTAGTCTAGGTGGTTAGG
t0016566	25	59	GGTAGAGCATTTGACTGCAGATCAA
t0016612	24	59	GGGATTGTAGTTCAATCGGTCAGA
t0016618	28	59	GGGGATGTAGCTCAAACGGTAGAGCGCT
t0016674	23	59	CAGTGGTAGAGCATTTGACTGCA
t0016726	27	59	ACGGACTGTAAATTCGTTGACGATATG
t0016799	20	58	GCGGGTATAGTTTAGTGGTA
t0016860	22	58	GGAGATGTAGCTCAAATGGTAG
t0016910	24	58	GGTAGAGCATTTGACTGCAGATCA
t0016972	21	58	GGGGATGTAGCTCAGAGGTAG
t0017166	26	57	GTGGTAGAGCATTTGACTGCAGATCA
t0017235	23	57	GCACCAGTGGTCTAGTGGTAGAA
t0017420	18	56	GGTGTCGTGGTGTAGTTG
t0017460	21	56	GGAGATGTAGCTCAAATGGTA
t0017481	21	56	AGCAGAAGGCCGTAGGTTCGT
t0017551	23	56	GGGGATGTAGCTCAGAGGTAGAG
t0017554	24	56	ACTTCTAATCAGGCGATTGTGGGT
t0017632	23	55	GGGATTGTAGTTCAATCGGTCAG
t0017747	26	55	GGTTCTATGGTCTAGCGGTTAGGACA
t0017831	23	55	GGCGGATGTAGCCAAGTGGATCA
t0017839	23	55	TGGTAGAGCATTTGACTGCAGAT
t0017969	25	54	GGAGATGTAGCTCAGATGGTAGAGC
t0017992	23	54	GGAGTTGTAGCTCAAATGGTAGA
t0018040	25	54	GCGGGGATAGCTCAGTTGGGAGAGC
t0018088	24	54	TCCGTTGTAGTCTAGTTGGTTAGG
t0018198	23	54	GGTGATGTAGCTCAAACGGTAGA
t0018254	23	53	GGTGGCTGTATTTTAGTGGTTAG
t0018293	23	53	GGAGATGTAGCTCAAATGGTAGA
t0018294	18	53	GCGTTTGTAGTCCAACGG
t0018318	23	53	CCGACCTTAGCTCAGTTGGTAGA
t0018381	27	53	GGGGATGTAGCTCAGATGGTAGAGCGC
t0018440	23	53	GGTAGAGCTGAGGACTGTAGATC

t0018539	24	52	AGTGGTAGAGCATTTGACTGCAGA
t0018541	20	52	GGTAGAGCATTTGACTGCAG
t0018575	27	52	GCGTTTGTAGTCCAACGGTAAGGATAA
t0018602	23	52	GCGGATGTAGCGCAGTTGGTAGC
t0018664	22	52	TCCGGAGCGGGAGATTGTGGGT
t0018724	20	52	CGAAAGGGCGTGGGTTCATA
t0018793	27	52	GGGGCTGTAGCTCAGATGGGAGAGCGC
t0018853	22	52	TGGTAGAGCTGAGGACTGTAGA
t0018894	24	51	GTCGATATGTCCGAGTGGTTAAGG
t0018961	24	51	GGGGATGTAGCTCAGATGGTAAAG
t0018995	22	51	AGGGATATAACTCAGCGGTAGA
t0019012	23	51	ACTTCTAATCAGGCGATTGTGGG
t0019069	28	51	GGGGCTGTAGCTCAGATGGGAGAGCGCC
t0019080	25	51	GGGGATGTAGCTCAGATGGTAGATC
t0019137	24	51	GTCTGGGTTGTGTAGTTGGTTATC
t0019151	25	51	AGTGGTAGAGCATTTGACTGCAGAT
t0019313	21	50	CCGACCTTAGCTCAGTTGGCA
t0019378	21	50	GCGTTTGTAGTCCAACGGTAC
t0019543	21	50	GCCCGTCTAGCTCAGTTGGTA
t0019579	20	50	CTAGCGGTTAGGACATTGGA
t0019612	23	49	CTTCTAATCAGGCGATTGTGGGT
t0019702	21	49	CCGACCTTAGCTCAGTTGGTA
t0019805	18	49	GGGGATATGGCGAAATTG
t0019953	20	49	GTGGATGTAGCTCAGATGGT
t0019968	27	49	GGTGTCGTGGTGTAGTTGGTTATCACT
t0020214	25	48	ACTTCTAATCAGGCGATTGTGGGTT
t0020216	22	48	TGTGTCGTGGTGTAGTTGGTTA
t0020229	21	48	GCGCCTGTAGCTCAGTGGAGA
t0020327	27	48	TTGGTAGAGCTGAGGACTGTAGATCCT
t0020475	21	47	GACCGCATAGCGCAGTGGATT
t0020574	27	47	GTAGAGCTGAGGACTGTAGATCCTTAG
t0020736	24	47	GGGGGTGTAGCTCATATGGTAGAA
t0020850	24	46	TCAGTGGTAGAGCATTTGACTGCA
t0020950	19	46	TCCATTGTCGTCTAGTCCG
t0020976	27	46	GGGGATATGGCGAAATTGGTAGACGCT
t0021086	25	46	GGGGATGTAGCTCAAATGGTCGAGC
t0021399	26	45	GGTGTCGTTGTGTAGTTGGTTATCAC
t0021435	24	45	GCACCAGTGGTCTAGTGGTAGAAT
t0021445	22	45	GTCGATATGTCCGAGTGGTTAA
t0021586	22	45	GCGCTCTTAGTTCAGTTCGGTA
t0021627	22	45	AGTTGGTTAGGATACTCGGCTC
t0021667	26	45	GCGTTTGTAGTCCAACGGTAAGGATA
t0022065	22	44	GGCGTCGTGGTGTAGTTGGTTA
t0022097	22	44	GGTAGAGCATTTGACTGCAGAT
t0022120	24	44	GCGCCTGTAGCTCAGTGGATAGAG
t0022159	23	43	GGGGATGTAGCTCAGATGGTAGT
t0022322	21	43	GGGGATATAGCTCAGTTGGTA
t0022399	22	43	TGGTAGAGCATTTGACTGCAGA
t0022409	26	43	GGGATTGTAGTTCAATCGGTCAGAGC
t0022422	21	43	TCCGGAGCGGGAGATTGTGGG

t0022433	23	43	TCCGGAGCGGGAGATTGTGGGTT
t0022470	24	43	CAGTGGTAGAGCATTTGACTGCAG
t0022526	26	43	GGGGATGTAGCTCAGATGGTAGAGCA
t0022536	28	43	GCGGATATGGTCGAATGGTAAAATTTCT
t0022561	23	43	GCGGATATGGTCGAATGGTAAAA
t0022730	24	42	GTGGTAGAGCATTTGACTGCAGAT
t0022747	19	42	TGGTTAGGATACTCGGCTC
t0022861	26	42	CGCGGGGTAGAGCAGTTTGGTAGCTC
t0022917	27	42	GGCGGATGTGGCCAAGTGGATCAAGGC
t0022928	26	42	GGTAGAGCATTTGACTGCAGATCAAG
t0022973	27	42	GCGGACGTAGCTCAGTTGGTAGAGCGC
t0022995	20	42	CCGACCTTAGCTCAGTTGGC
t0023144	27	41	GGCGGATGTAGCCAAGTGGATCAAGGC
t0023287	28	41	GGCGGATGTAGCCAAGTGGATCAAGGCA
t0023290	24	41	GGGGATGTAGCTCAAACGGTAGAT
t0023329	22	41	TGCGAGAGGTACGGGGATCGTT
t0023381	19	41	CGAAAGGGCGTGGGTTCAT
t0023491	27	41	TGGTAGAGCTGAGGACTGTAGATCCTT
t0023507	22	41	GGCGGATGTAGCCAAGTGGATC
t0023634	19	40	GGTGATGTAGCTCAGATGG
t0023682	24	40	GTTCGTGCCCCACGGTGGGCGCCA
t0023745	22	40	GGGCCTGTAGCTCAGAGGATTA
t0023754	19	40	CTAGCGGTTAGGACATTGG
t0024225	24	39	GCGCGGGTGGCGGAATAGGTAGAC
t0024253	24	39	TCCGGAGCGGGAGATTGTGGGTTC
t0024266	23	39	TCAGTTGGTAGAGCTGAGGACTG
t0024505	26	39	GGTTCCATGGTCTAGCGGTTAGGACA
t0024662	22	39	GGGATGTAGCTCAGATGGTAGA
t0024747	27	38	GGGGCTGTGGCGCAGTTGGTAGCGCGT
t0024855	20	38	AGTCCCGTAGCTCAGTTGGT
t0024870	20	38	GGGGATGTAGCTCAGATGGA
t0024984	23	38	TGGTAGAGCTGAGGACTGTAGAT
t0025115	29	38	GCGGACGTAGCTCAGTTGGTAGAGCGCAA
t0025387	23	37	GGGGATGTAGCTCAGATGGGAGA
t0025416	24	37	GGTTAGGATACTCGGCTCTCACCC
t0025588	24	37	TGGGATGTAGCTCAGATGGTAGAG
t0025600	28	37	GGGGATGTAGCTCAGATGGTAGAGCGCT
t0025697	20	37	GCAGAAGGCCGTAGGTTCGT
t0025698	22	37	GCACCAGTGGTCTAGTGGTAGA
t0025703	20	37	GCGCCTGTAGCTCAGTGGAT
t0025741	23	37	CTAGTTGGTTAGGATACTCGGCT
t0025812	21	37	GGCGGATGTGGCCAAGTGGAT
t0025853	24	37	GGTGATGTAGCTCAAACGGTAGAG
t0025889	25	37	GAGCGGAGGACTGTAGATCCTTAGG
t0025926	27	37	GGGCGTTTGGTCTAGTGGTATGATTCT
t0025949	23	37	TTGCGAGAGGTACGGGGATCGTT
t0026041	22	36	GGGGATGTAGCTCAGATGGGAG
t0026056	22	36	CTCGAGAGAGGGCGTGGGTTCA
t0026065	22	36	GTTGTCGTGGTGTAGTTGGTTA
t0026182	27	36	GGAGAGATGGCTGAGTGGACTAAAGCG

t0026209	24	36	GGGGATGTAGCTCAAATGGTAGAA
t0026234	20	36	GCCCGTCTAGCTCAGTTGGT
t0026310	23	36	CTCAGTTGGTAGAGCTGAGGACT
t0026432	22	36	GCGGGGATAGCTCAGTTGGGCG
t0026459	21	36	GGGGATGTAGCTCATATGGTA
t0026510	26	36	GGGGATGTAGCTCAAACGGTAGAGCC
t0026573	23	36	GTGGTAGAGCATTTGACTGCAGA
t0026591	20	36	CCGAAAGGGCGTGGGTTCAT
t0026611	21	36	GGTAGAGCATTTGACTGCAGA
t0026781	21	35	CGGGATGTAGCTCAGATGGTA
t0026850	21	35	GGTTCTATGGTGTAGTGGTTA
t0026893	26	35	TCAGTGGTAGAGCATTTGACTGCAGA
t0027025	25	35	GTCGATATGTCCGAGTGGTTAAGGA
t0027055	22	35	GGGGATGTAGCTCAGAGGTAGA
t0027084	26	35	GGATGGATGTCTGAGCGGTTGAAAGA
t0027213	21	35	AGGGATGTAGCGCAGCTTGGT
t0027343	23	34	GGTGTTGTAGCTCAAATGGTAGA
t0027397	25	34	CAGTTGGTAGAGCTGAGGACTGTAG
t0027543	19	34	GCGCCTGTAGCTCAGTGGA
t0027558	23	34	AGTTGGTAGAGCTGAGGACTGTA
t0027577	24	34	AGGGATATAACTCAGCGGTAGAGT
t0027618	25	34	GGTAGAGCGGAGGACTGTAGATCCT
t0027685	22	34	CCGGAGCGGGAGATTGTGGGTT
t0027763	26	34	GGGGATGTAGCTCAGATGGTAGAGCC
t0027815	23	34	GGTGATGTAGCTCAAATGGTAGA
t0027847	27	34	AGGACATTGGACTCTGAATCCAGTAAC
t0027884	23	34	GGGGATGTAGCTCAGATGGTAAA
t0027926	22	34	GTGGATGTAGCTCAGATGGTAG
t0027941	20	34	GGTGCTGTGGTGTAGTGGTT
t0027955	25	34	GTAGAGCGGAGGACTGTAGATCCTT
t0028035	20	34	GGGGATGTAGCTCAGAGGTA
t0028170	28	33	GGGGCTGTAGCTCAGCTGGGAGAGCACC
t0028295	22	33	CTTCTAATCAGGCGATTGTGGG
t0028474	24	33	GACTTCTAATCAGGCGATTGTGGG
t0028517	20	33	CCGGAGCGGGAGATTGTGGG
t0028525	24	33	GGGGATGTAGCTCAGATGGTAAAA
t0028565	23	33	GGGATGTAGCTCAGATGGTAGAG
t0028622	25	33	AGGGATGTAGCGCAGCTTGGTAGCG
t0028806	26	33	GGGGCTGTAGCTTAGTGGTAAAGCCT
t0028809	24	33	TGGTAGAGCTGAGGACTGTAGATC
t0028851	24	33	GGTTCTATGGTGTAGTGGTTAGCA
t0028968	20	32	AATTCCCGTCGTTCGCCCCA
t0028983	24	32	CTTCTAATCAGGCGATTGTGGGTT
t0029048	20	32	TCCTCAGTAGCTCAGTGGTA
t0029297	20	32	CGCGGGGTAGAGCAGTTTGG
t0029347	19	32	GGGGTGGTGGCGCAGTTGG
t0029393	26	32	GCGTTTGTAGTCCAACGGTCAGGATA
t0029423	19	32	ACACTGAAGGTCTCCGGTT
t0029471	27	32	TCAGTGGTAGAGCATTTGACTGCAGAT
t0029644	22	32	CAGTTGGTAGAGCTGAGGACTG

t0029678	29	32	GGGGATGTAGCTCAGATGGTAGAGCGCTC
t0029709	26	31	GGGGCTGTAGCTCAGATGGGAGAGCG
t0029798	23	31	TTCGTGCCCCACGGTGGGCGCCA
t0029824	21	31	GCGGGGATAGCTCAGTTGGGC
t0029857	22	31	GTCAAGATGGCCGAGTTGGTCT
t0029881	26	31	ACTTCTAATCAGGCGATTGTGGGTTC
t0029885	22	31	GGGGATATAGCTCAGTTGGTAG
t0030016	27	31	GCGCCTGTAGCTCAACGGATAGAGCAT
t0030017	23	31	GGGGATGTAGCTCATATGGTAGA
t0030047	27	31	GCGGGCGTAGCTCAGGGGTAGAGCACA
t0030054	23	31	GTCTGGGTCGTGTAGTTGGTTAT
t0030092	26	31	GGGCGTTTGGTCTAGTGGTATGATTC
t0030098	24	31	GGTGGCTGTAATTTAGTGGTTAGA
t0030127	18	31	GGGGATGTAGCTCAGATG
t0030155	20	31	TCCGGAGCGGGAGATTGTGG
t0030268	24	31	GTAGAGCGGAGGACTGTAGATCCT
t0030410	26	31	GGGGATGTAGCTCAGATGGTAGAGCG
t0030437	22	31	GTTGGTAGAGCTGAGGACTGTA
t0030787	26	30	GGTAGAGCGGAGGACTGTAGATCCTT
t0030861	20	30	GCGAGAGGTACGGGGATCGT
t0030866	23	30	TAAGCAGAAGGCCGTAGGTTCGT
t0030996	27	30	GGGGCTGTGGCGCAGCTGGTAGCGCAT
t0031340	20	30	GGGGATGTAGTTCAGATGGT
t0031573	20	29	CGGGATGTAGCTCAGATGGT
t0031619	20	29	AGGTGGTTAGGATACTCGGC
t0031980	20	29	GGGGATGTAGCTCATATGGT
t0032006	21	29	GGGGATGTAGCTCAGATGGTG
t0032017	19	29	GCGGATATGGTCGAATGGT
t0032417	21	29	TCGAGAGAGGGCGTGGGTTCA
t0032449	23	29	AGATTGAGGTTCTGGTCCGAAAG
t0032546	25	28	GTGGTAGAGCATTTGACTGCAGATC
t0032617	27	28	GCGTTTGTAGTCCAACGGTCAGGATAA
t0032623	29	28	GGGGCTGTAGCTCAGCTGGGCGAGCACCT
t0032678	22	28	GTGTCGTGGTGTAGTTGGTTAT
t0032681	24	28	TGTGTCGTGGTGTAGTTGGTTATC
t0032862	26	28	TGGTAGAGCGGAGGACTGTAGATCCT
t0032930	25	28	GTGGACGTGCCGGAGTGGTTATCGG
t0033120	28	28	TCCATTGTCGTCTAGTCCGGTTAGGATA
t0033166	19	28	GGGGATGTAGCTCAGAGGT
t0033277	21	28	CCGGAGCGGGAGATTGTGGGT
t0033635	26	27	GTAGAGCGGAGGACTGTAGATCCTTA
t0033697	18	27	CGAAAGGGCGTGGGTTCA
t0033832	27	27	GCACCAGTGGTCTAGTGGTAGAATAGT
t0033891	23	27	GGGGATGTAGCTCAGATGGTGAG
t0033943	19	27	TGGGGCGTGGCCAAGCGGT
t0034011	26	27	GGGGATGTAGCTCAAACGGTAGAGCG
t0034019	24	27	AGATTTGAAATCTGTTGGGCTTCG
t0034046	24	27	CTCAGTTGGTAGAGCTGAGGACTG
t0034057	22	27	GGGGATGTAGCTCAGATGGTTT
t0034113	20	27	CGAAAGGGCGTGGGTTCAGA

t0034248	30	27	GGGCCTGTAGCTCAGAGGATTAGAGCACGT
t0034286	23	27	TGTGTCGTGGTGTAGTTGGTTAT
t0034345	25	27	TCAGTGGTAGAGCATTTGACTGCAG
t0034354	22	27	CGGGATGTAGCTCAGATGGTAG
t0034376	26	27	GCGGGTGTAGCTCAATGGTAGAGCAG
t0034420	21	27	GTTGAGATGGCCGAGTTGGTC
t0034454	25	27	AGATTTGAAATCTGTTGGGCTTCGC
t0034576	24	27	GGTGATGTAGCTCAAATGGTAGAG
t0034583	24	27	GGGGATATAGCTCAGTTGGTAGAG
t0034599	22	27	GGGGATGTAGCTCAGACGGTAG
t0034600	21	27	GGCGTCGTGGTGTAGTTGGTT
t0034844	20	26	GGGGATATAGCTCAGTTGGT
t0034910	24	26	GGGGTTGTAGCTCAAATGGTAGAA
t0035042	26	26	GGGGGTGTAGCTCATATGGTAGAGCT
t0035047	23	26	GGTGGCTGTAATTTAGTGGTTAG
t0035182	24	26	CAGTTGGTAGAGCTGAGGACTGTA
t0035256	23	26	TAGTTGGTTAGGATACTCGGCTC
t0035304	26	26	CAGTTGGTAGAGCTGAGGACTGTAGA
t0035461	27	26	GGAGGTATGGCTGAGTGGCTTAAGGCA
t0035556	22	26	GTCTGGGTGGTGTAGTTGGTTC
t0035609	25	26	CAGTGGTAGAGCATTTGACTGCAGA
t0035802	24	26	GGGGATGTAGCTCATATGGTAGAG
t0035809	28	26	GCGGGCGTAGCTCAGGGGTAGAGCACAA
t0035885	24	26	GTGGATGTAGCTCAGATGGTAGAG
t0035899	26	26	GGGCATTTGGTCTAGTGGTATGATTC
t0036057	21	25	GGTTAGGATACTCGGCTCTCA
t0036065	28	25	GGGCCTGTAGCTCAGTTGGCAGAGCATC
t0036066	21	25	GCGTTTGTAGTCCAACGGTCC
t0036131	24	25	GCGCATCTGGTGTAGTGGTATCAT
t0036176	20	25	GGGGATGTAGCTCAGATGGC
t0036177	19	25	GGCGATGTAGCTCAGATGG
t0036484	22	25	GCGGATGTAGCGCAGTTGGTAG
t0036502	18	25	GGGGTGGTGGCGCAGTTG
t0036569	21	25	ATCAGAGTGGCGCAGCGGAAG
t0036611	26	25	GTCTGGGTGGTGTAGTTGGTTATCAC
t0036628	25	25	CTAGTTGGTTAGGATACTCGGCTCT
t0036688	26	25	TCCGTTGTAGTCTAGTTGGTTAGGAT
t0036698	22	25	TCAGTTGGTAGAGCTGAGGACT
t0036824	20	25	AGGATACTCGGCTCTCACCC
t0036887	24	25	CGGGATGTAGCTCAGATGGTAGAG
t0036924	20	25	GGGTCCATAGCTCAGTGGTA
t0036954	23	25	GTTGAGATGGCCGAGTTGGTCTA
t0037089	23	25	GGAGGTATGGCTGAGTGGCTTAA
t0037234	23	25	GCGCATCTGGTGTAGTGGTATCA
t0037247	21	25	GGAGGTATGGCTGAGTGGCTT
t0037249	25	25	GGTGATGTAGCTCAGATGGTAGAGC
t0037285	21	25	AAGCAGAAGGCCGTAGGTTCG
t0037303	22	24	TAGTTGGTTAGGATACTCGGCT
t0037337	22	24	GGGGATGTAGCTCAGATGGTCT
t0037394	20	24	GCACCAGTTGTCTAGTGGTA

t0037453	29	24	CGGGGTGTAGCTCAGCCTGGTAGAGCGCT
t0037507	26	24	TCTAGTTGGTTAGGATACTCGGCTCT
t0037527	20	24	CGAGAGGTACGGGGATCGTT
t0037533	25	24	TCCGTTGTAGTCTAGTTGGTTAGGA
t0037602	26	24	GGGGATGTAGCTCAAACGGTAGAGCA
t0037643	28	24	GGGGCCGTAGCTCAGCTGGGAGAGCGCT
t0037946	20	24	GGCGGATGTGGCCAAGTGGA
t0037969	23	24	TTGGTAGAGCTGAGGACTGTAGA
t0038022	24	24	CCGACCTTAGCTCAGTTGGCAGAG
t0038041	25	24	CTCAGTGGTAGAGCATTTGACTGCA
t0038054	23	24	CTAAGCAGAAGGCCGTAGGTTCG
t0038080	21	24	GTTGTCGTGGTGTAGTTGGTT
t0038177	24	24	TCTAGTTGGTTAGGATACTCGGCT
t0038184	24	24	CCGACCTTAGCTCAGTTGGTAGAG
t0038277	24	24	GGTGTTGTAGCTCAAATGGTAGAG
t0038336	23	24	GTTGGTAGAGCTGAGGACTGTAG
t0038393	22	24	GCGCCTGTAGCTCAGTGGATAG
t0038421	22	24	GGTGTTGTAGCTCAAATGGTAG
t0038462	22	24	GGGGATGTAGCTCATATGGTAG
t0038512	23	24	GCGCCTGTAGCTCAGTGGACAGA
t0038532	25	24	GCGCGGGTGGCGGAATAGGTAGACG
t0038708	22	23	GGGGATGGAGCTCAGATGGTAG
t0038807	23	23	GTTAGGATACTCGGCTCTCACCC
t0038980	23	23	GTCTGGGTAGTGTAGTTGGTTAT
t0038984	25	23	GGGGCTGTAGCTCAGATGGGAGAGC
t0039060	22	23	GGGGATTTAGCTCAGATGGTAG
t0039113	25	23	GGTTCTATGGTCTAGCGGTTAGGAC
t0039284	22	23	GTGGTAGAGCATTTGACTGCAT
t0039423	24	23	TAGTTGGTTAGGATACTCGGCTCT
t0039471	19	23	TCGAGAGAGGGCGTGGGTT
t0039620	25	23	GCGTTTGTAGTCCAACGGTAAGGAT
t0039674	28	23	GGTAGAGCTGAGGACTGTAGATCCTTAG
t0039717	21	23	TGGGGCGTGGCCAAGCGGTAA
t0039739	28	23	AGGGCTATAGCTCAGTTAGGTAGAGCAC
t0039864	22	23	GGGGATGTAGCTCAGATGGTGG
t0039878	23	23	AGATTTGAAATCTGTTGGGCTTC
t0039977	23	23	AGTGGTAGAGCATTTGACTGCAT
t0040162	20	23	GTTAGGATACTCGGCTCTCA
t0040202	27	23	GGTAGAGCGGAGGACTGTAGATCCTTA
t0040331	29	22	GGGGATGTAGCTCAAACGGTAGAGCGCTC
t0040384	21	22	GCGCTCTTAGTTCAGTTCGGT
t0040395	19	22	TGGGATGTAGCTCAGATGG
t0040445	18	22	GTCTGGTGTAGTTGGTTA
t0040515	22	22	GGGGGTGTAGCTCATATGGTAT
t0040527	27	22	GCGGGCGTGGCGGAACTGGTAGACGCA
t0040593	22	22	GGTTCTATGGTGTAGTGGTTAG
t0040615	23	22	GGGGATGTAGCTCAGATGGTAGC
t0040671	23	22	GGTGTCGTGGGGTAGTTGGTTAT
t0040690	24	22	GACACACTGAAGGTCTCCGGTTCG
t0040934	23	22	CGGGATGTAGCTCAGATGGTAGA

t0040982	21	22	TAAGCAGAAGGCCGTAGGTTC
t0040996	27	22	GGTAGAGCATTTGACTGCAGATCAAGA
t0041019	26	22	GGGCCTGTAGCTCAGAGGATAGAGCA
t0041133	23	22	GGGGGTGTAGCTCATATGGTATA
t0041174	27	22	GGGGCTGTAGCGCAGTTGGTAGCGCGT
t0041414	19	22	CCGAAAGGGCGTGGGTTCA
t0041443	22	22	GGGGTTGTAGCTCAAATGGTAT
t0041476	21	22	GTGGCTGTAGTTTAGTGGTTA
t0041513	22	22	GGTGTCGTGGGGTAGTTGGTTA
t0041593	28	22	GGGCATTTGGTCTAGTGGTATGATTCTC
t0041783	27	22	GGGCATTTGGTCTAGTGGTATGATTCT
t0041954	22	21	CTAGTTGGTTAGGATACTCGGC
t0042057	20	21	GTGTCGTGGTGTAGTTGGTT
t0042108	21	21	GTGTCGTGGTGTAGTTGGTTA
t0042354	22	21	GGGTATGTAGCTCAGATGGTAG
t0042421	26	21	AGTGGTAGAGCATTTGACTGCAGATC
t0042470	20	21	GGGATTGTAGTTCAATCGGA
t0042500	23	21	AGCGGGGGAGAGGAATTGGTCAA
t0042660	27	21	GCGGAAATAGCTTAATGGTAGAGCATA
t0042687	26	21	CTCAGTTGGTAGAGCTGAGGACTGTA
t0042792	31	21	GGGGCTGTAGCTCAGATGGGAGAGCGCCGCA
t0042815	23	21	GGGGATGTAGCTCAGACGGTAGA
t0042869	25	21	GGTGGCTGTAATTTAGTGGTTAGAA
t0042955	28	21	TGGTAGAGCATTTGACTGCAGATCAAGA
t0043005	20	21	GGTTCCATGGTCTAGCGGTT
t0043043	21	21	TGTGTCGTGGTGTAGTTGGTT
t0043050	24	21	GGTGGCTGTATTTTAGTGGTTAGA
t0043070	26	21	TGGTAGAGCATTTGACTGCATATCAA
t0043097	22	21	GCGGGTGGCGGAATAGGTAGAC
t0043128	23	21	ATTTGAAATCTGTTGGGCTTCGC
t0043243	24	21	GTCTGGGTCGTGTAGTTGGTTATC
t0043245	20	21	GGTTAGGATACTCGGCTCTC
t0043474	21	21	AGTGGTAGAGCATTTGACTGC
t0043952	22	20	GCAGTGGATTAGCGCTTTTGAC
t0043964	25	20	GGTTCGTGCCCCACGGTGGGCGCCA
t0043974	24	20	GGGGATGTAGCTCAGATGGTAGTT
t0044056	26	20	CAGTGGTAGAGCATTTGACTGCAGAT
t0044176	20	20	GTCAGGATGGCCGAGTGGTC
t0044425	23	20	TCCATTGTCGTCTAGTCCGGTAG
t0044506	21	20	GGGGATGTAGCTCAGATGGGG
t0044746	27	20	GTCGTTGTAGTATAGTGGTAAGTATTC
t0044850	25	20	GGGGATGTAGCTCAGATGGTAGATT
t0045011	22	20	AGTTGGTAGAGCTGAGGACTGT
t0045052	28	20	GGGGCTGTGGCGCAGCTGGTAGCGCATC
t0045167	20	20	GGGGATGTAGCTCATGGTAG
t0045337	22	20	GTCTGGGGGGTGTAGTTGGTTA
t0045372	20	20	GGAGTTGTAGCTCAAATGGT
t0045421	25	20	GTCTGGGTCGTGTAGTTGGTTATCA
t0045426	18	20	CGAGAGAGGGCGTGGGTT
t0045572	25	20	GACACACTGAAGGTCTCCGGTTCGT

t0045654	21	20	GGGGATGTATCTCAGATGGTA
t0045923	26	19	CTCAGTGGTAGAGCATTTGACTGCAG
t0045980	22	19	GTTGAGATGGCCGAGTTGGTCT
t0045991	22	19	TCCGTTGTAGTCTAGCTGGTTA
t0046036	24	19	GGGGATGTAGCTCAGATGGTAGAC
t0046044	27	19	GTCGTTGTAGTATAGTGGTGAGTATTC
t0046118	26	19	TGGTAGAGCATTTGACTGCACATCAA
t0046135	18	19	GGTTCTATGGTGTAGTGG
t0046223	25	19	TGTGTCGTGGTGTAGTTGGTTATCA
t0046302	23	19	GTGGATGTAGCTCAGATGGTAGA
t0046368	23	19	GGTAGAGCATTTGACTGCAGATC
t0046470	22	19	TGGTAGAGCATTTGACTGCATC
t0046559	19	19	AGAGGGCGTGGGTTCAAAT
t0046631	22	19	GGGGATGTAGCTTAGATGGTAG
t0046761	21	19	GGGATTGTAGTTCAATCGGTA
t0046777	26	19	TGGTAGAATAGTACCCTGCCACGGTA
t0047065	24	19	TGGTAGAGCATTTGACTGCACATC
t0047213	20	19	GGCGGATGTAGCCAAGTGGA
t0047216	24	19	GGGGGGGTAGCTCATATGGTAGAG
t0047242	21	19	GGTGCTGTGGTGTAGTGGTTA
t0047356	23	19	GCGCCTGTAGCTCAGTGGAGAGC
t0047596	18	19	GTCGTTGTAGTATAGTGG
t0047607	24	19	GCGTTTGTAGTCCAACGGTAAGGA
t0047856	24	19	TCAGTTGGTAGAGCTGAGGACTGT
t0047941	25	18	GGGGATGTAGCTCAGATGGTAGAGT
t0048073	26	18	GTCTGGGTGGTGTAGTTGGTCATCAC
t0048163	22	18	GATTTGAAATCTGTTGGGCTTC
t0048173	24	18	GTTGGTAGAGCTGAGGACTGTAGA
t0048213	23	18	GGGAATGTAGCTCAAATGGTAGA
t0048227	24	18	GTCGGGATAGCTCAGCTGGTAGAG
t0048291	20	18	GTAGAGCGGAGGACTGTAGA
t0048365	24	18	AGTTGGTAGAGCTGAGGACTGTAG
t0048428	26	18	GCGCGGGTGGCGGAATAGGTAGACGC
t0048728	20	18	GTGGTAGAGCATTTGACTGC
t0048750	28	18	GTTGGTAGAGCTGAGGACTGTAGATCCT
t0048809	23	18	GGGTCCATAGCTCAGTGGTAGAG
t0048827	25	18	GCGCATCTGGTGTAGTGGTATCATA
t0048863	23	18	GGGGATGTAGCTTAGATGGTAGA
t0048924	19	18	GTAGAGCGGAGGACTGTAG
t0048963	24	18	GGGAGTGTAGCTCATATGGTAGAG
t0049022	21	18	AGCTGGTTAGGATACTCGGCT
t0049255	26	18	GCTCAGTGGTAGAGCATTTGACTGCA
t0049394	22	18	CTCGTGAGAGGGCGTGGGTTCA
t0049402	20	18	TGGTTAGGATACTCGGCTCT
t0049421	20	18	ATCAGAGTGGCGCAGCGGAA
t0049570	27	18	TGGTAGAGCATTTGACTGCATATCAAG
t0049576	22	18	GGTGGCTGTAGTTTAGTGGTTC
t0049615	22	18	GGTGTCGTGGTGTAGTTGGTTT
t0049699	22	18	GGGGATGTAGCTCAGATGGTGA
t0049912	21	18	CAGTTGGTAGAGCGGAGGACT

t0050058	19	18	GCACCAGTTGTCTAGTGGT
t0050067	26	18	ACGGACTGTAAATTCGTTGACGATAT
t0050076	27	18	GCGGAAGCGTGGTGGGCCCATAACCCA
t0050118	25	18	AGATTTAGGCTCTGGTCCGAAAGGG
t0050240	24	18	GGTGCTGTGGTGTAGTGGTTATCA
t0050303	23	18	TGGGATGTAGCTCAAACGGTAGA
t0050592	28	17	TGGTAGAGCGGAGGACTGTAGATCCTTA
t0050683	19	17	GGGGATGTAGCTCATGGTA
t0050833	23	17	GGTTAGGATACTCGGCTCTCACC
t0050869	23	17	GGTTCGATTCCGATAGCCGGCTC
t0051155	22	17	AGAGCGGAGGACTGTAGATCCT
t0051259	21	17	AGCAGAAGGCCGTAGGTTCGA
t0051328	23	17	GGGGATGGAGCTCAGATGGTAGA
t0051342	27	17	GTGGTAGAGCATTTGACTGCATATCAA
t0051500	21	17	AGGGATGTAGCTCAGATGGTA
t0051718	20	17	TTGGTTAGGATACTCGGCTC
t0051796	26	17	GCGCATCTGGTGTAGTGGTATCATAG
t0051931	26	17	GGGGCTGTAGCTCAGCTGGGAGAGCA
t0051953	22	17	GGGGATGTAGCTCAAATGGTAT
t0052088	25	17	TCCGGAGCGGGAGATTGTGGGTTCG
t0052228	20	17	GCTGGTTAGGATACTCGGCT
t0052297	20	17	GGTCCCGTAGCTCAGTTGGT
t0052442	21	17	TGGTTAGGATACTCGGCTCTC
t0052522	24	17	GCCCAGATGGCGGAATTGGTAGAC
t0052536	21	17	GGGGTGTAGCTCATATGGTAG
t0052802	21	17	TCCTCAGTAGCTCAGTGGTAG
t0053026	22	17	GGAGGTATGGCTGAGTGGCTTA
t0053156	20	17	CAAGCAAGGACTCTACCACG
t0053186	28	17	GGGGCTGTAGCTCAGATGGGCGAGCGCT
t0053206	21	17	GGGGATATGGCGAAATTGGTC
t0053208	20	17	GGTAGAGCGGAGGACTGTAG
t0053330	23	16	GTCAAGATGGCCGAGTTGGTCTA
t0053350	19	16	AGAGGTACGGGGATCGATA
t0053481	24	16	GTTGTCGTGGTGTAGTTGGTTATC
t0053491	20	16	GGGGATGTATCTCAGATGGT
t0053514	24	16	CTAGTTGGTTAGGATACTCGGCTC
t0053531	28	16	TTGGTAGAGCTGAGGACTGTAGATCCTT
t0053781	19	16	TGGTAGAGCATTTGACTGC
t0053832	29	16	ATTGGACTCTGAATCCAGTAACCCGAGTT
t0053931	23	16	CAGTTGGTAGAGCTGAGGACTGT
t0054065	19	16	GGTAGAGCGGAGGACTGTA
t0054151	28	16	GCACCAGTGGTCTAGTGGTAGAATAGTA
t0054169	20	16	GGGGATGTAGCTCAGGGTAG
t0054241	23	16	AGAGCGGAGGACTGTAGATCCTT
t0054343	24	16	GGGGCTGTAGCTCAGATGGGAGAG
t0054346	26	16	GGGGATGTAGCTCAGATGGTAGATTT
t0054441	28	16	GTCGGGATAGCTCAGCTGGTAGAGCAGA
t0054558	22	16	GGTGTCGTGGTGTAGTTGGTAT
t0054725	21	16	GGGGATGAAGCTCAGATGGTA
t0054735	23	16	AGCTGGTAGAGCAGAGGACTGAA

t0054755	18	16	TCGAGAGAGGGCGTGGGT
t0054821	24	16	AAGGTTGTGGGTTCAAATCATGCC
t0054906	20	16	GCCCCTATAGCTCAGTGGTA
t0054978	21	16	CGGAGCGGGAGATTGTGGGTT
t0055000	20	16	GGGGATGTAGCTCAGAGGGT
t0055034	25	16	GTTGTCGTGGTGTAGTTGGTTATCA
t0055143	24	16	AGCGGAAGCGTGGTGGGCCCATAA
t0055224	20	16	GCGGATGTGGCCAAGTGGAT
t0055417	23	16	AGCGGGGTAGAGGAATTGGTCCA
t0055435	28	16	GGCGGATGTGGCCAAGTGGATCAAGGCA
t0055438	23	16	GGGGATTTAGCTCAGATGGTAGA
t0055510	27	16	CTCAGTTGGTAGAGCTGAGGACTGTAG
t0055634	24	16	GGGGATGTAGCTCAGACGGTAGAG
t0055699	20	16	GCGTTTGTAGTCCAACGGGT
t0055829	25	16	GGATGGATGTCTGAGCGGTTGAAAG
t0056000	28	16	GCGTTTGTAGTCCAACGGTAAGGATAAT
t0056069	23	16	GGGAGTGTAGCTCATATGGTAGA
t0056215	25	16	GGGGATGTAGCTCAGATGGTAGAAA
t0056384	23	15	ACACACTGAAGGTCTCCGGTTCG
t0056518	25	15	GCGGATGTGGCGAAATTGGTAGACG
t0056616	23	15	AGCGGAAGCGTGGTGGGCCCATA
t0056699	22	15	GTAGAGCGGAGGACTGTAGATC
t0056710	21	15	GGGGATGTAGTTCAGATGGTA
t0056755	20	15	GGGGATGAAGCTCAGATGGT
t0056762	20	15	GTGGCTGTAGTTTAGTGGTA
t0056800	19	15	TTGGTAGAGCGGAGGACTG
t0056924	26	15	GGGGGTGTAGCTCATATGGTAGAGCA
t0057048	19	15	GTGGCTGTAGTTTAGTGGT
t0057147	25	15	GGCGTCGTGGTGTAGTTGGTTATCA
t0057201	25	15	GGGGATGTAGCTCAAACGGTAGAGT
t0057540	22	15	TCGAGAGAGGGCGTGGGTTCAT
t0057633	21	15	GTAGAGCGGAGGACTGTAGAT
t0057643	21	15	GCTGGAATAGCTCAGTTGGTT
t0057705	22	15	GGTTCTATGGTCTAGCGGTTAG
t0057734	25	15	ATGATTGGAAATCATGTGGGCTTTG
t0057771	20	15	CGGAGCGGGAGATTGTGGGT
t0057835	25	15	TGGTAGAGCATTTGACTGCATATCA
t0057910	19	15	GAAAGGGCGTGGGTTCAAA
t0057912	27	15	CTCAGTGGTAGAGCATTTGACTGCAGA
t0057935	22	15	TTCCAAGCAATAGATCCGGGTT
t0057968	22	15	CAGTGGTAGAGCATTTGACTGC
t0058064	21	15	TCAAGCAAGGACTCTACCACG
t0058082	21	15	AGTGGTATGATTCTCGCTTTG
t0058111	26	15	GGGGATGTAGCTCAGATGGTCGAGCT
t0058227	21	15	TTCAAGCAAGGACTCTACCAC
t0058270	20	15	GGGGTTGTAGCTCAGATGGT
t0058301	22	15	GTGGTAGAGCATTTGACTGCCG
t0058466	24	15	GGAGGTATGGCTGAGTGGCTTAAG
t0058535	21	15	GCGGATATGGTCGAATGGTAC
t0058653	28	15	GGTTCTATGGTCTAGCGGTTAGGACATT

t0058698	27	15	GCGGGTGTAGCTCAATGGTAGAGCAGA
t0058787	22	15	TTGGTAGAGCTGAGGACTGTAG
t0058843	23	15	GGGGATGTAGCTCAAATGGTATA
t0058996	24	15	GGTAGAGCATTTGACTGCACATCA
t0058999	28	15	GCGGGTGTAGCTCAATGGTAGAGCAGAA
t0059039	18	15	GCGGATATGGTCGAATGG
t0059204	23	15	TGCGAGAGGTACGGGGATCGTTA
t0059327	21	15	GTTAGGATACTCGGCTCTCAC
t0059445	22	15	GGTGATGTAGCTCAAATGGTAG
t0059479	26	15	GACTTCTAATCAGGCGATTGTGGGTT
t0059684	21	15	GGGGATGTAGCTCAGATGGCA
t0059791	28	15	GCGCCTGTAGCTCAGTGGATAGAGCTTC
t0059850	20	14	GTAGAGCAGAGGACTGTAGA
t0059868	21	14	CGGTACTGACCGGGGTTCGTT
t0059881	18	14	GCACCAGTGGTCTAGTGG
t0059907	23	14	GGTTCTATGGTGTAGTGGTTAGC
t0059920	22	14	ACACTGAAGGTCTCCGGTTCGT
t0060074	28	14	GCCCCTATCGTCTAGTGGTCAGGACATC
t0060169	25	14	GCACCAGTGGTCTAGTGGTAGAATA
t0060250	19	14	CGAGAGGTACGGGGATCGT
t0060271	24	14	AGTGGTAGAGCATTTGACTGCACA
t0060297	24	14	GTTAGGATACTCGGCTCTCACCCG
t0060476	24	14	GGTGTCGTGGTGTAGTTGGTTATA
t0060490	19	14	GACAGTTTGGCCGAGTGGT
t0060668	24	14	GGTAGAGCGGAGGACTGTAGATCC
t0060831	22	14	GGGGATGTAGCTCAGATGGTTG
t0060894	20	14	GGGGGTGTAGCTCAGATGGT
t0060895	26	14	GTGGACGTGCCGGAGTGGTTATCGGT
t0060927	26	14	GCAGTGGATTAGCGCTTTTGACTTCG
t0061051	28	14	GGGCGTTTGGTCTAGTGGTATGATTCTC
t0061130	20	14	AGATTTAGGCTCTGGTCCGA
t0061216	22	14	AGCGGGGGAGAGGAATTGGTCA
t0061231	28	14	AGGACATTGGACTCTGAATCCAGTAACC
t0061308	25	14	GTTGGTAGAGCTGAGGACTGTAGAT
t0061328	19	14	GGGGATGTAGTTCAGATGG
t0061355	24	14	GTGTCGTGGTGTAGTTGGTTATCA
t0061493	25	14	CTCCGGAGCGGGAGATTGTGGGTTC
t0061623	25	14	GGGCGTTTGGTCTAGTGGTATGATT
t0061792	27	14	GCGGGCGTGGCGGAACTGGTAGACGCG
t0061841	27	14	GCGCATCTGGTGTAGTGGTATCATAGT
t0061843	25	14	GGGGATATGGCGAAATTGGTCGACG
t0062100	19	14	GTCTGTGGTGTAGTTGGTT
t0062108	22	14	GGGGTGTAGCTCATATGGTAGA
t0062136	23	14	GGCGTCGTGGTGTAGTTGGTTAT
t0062151	19	14	GTGGTTAGGATACTCGGCT
t0062213	27	14	GGGCCTGTAGCTCAGAGGATAGAGCAC
t0062225	22	14	GGGATGTAGCTCAAACGGTAGA
t0062295	22	14	GTTAGGATACTCGGCTCTCACC
t0062486	18	14	GGAGAGATGGCTGAGTGG
t0062505	23	14	GCGGGGATAACTCAGTTGGGAGA

t0062518	24	14	TGGGTTGTAGCTCAAATGGTAGAG
t0062563	21	14	ACACTGAAGGTCTCCGGTTCG
t0062660	21	14	GGTGTCGTGGTGTAGTTGGGT
t0062732	19	14	GGTTAGGATACTCGGCTCT
t0062734	19	14	TCGTGAGAGGGCGTGGGTT
t0062777	23	14	GGGTATGTAGCTCAGATGGTAGA
t0062960	23	14	GGGGATGTAGCTCAAAGGTAGAG
t0063061	23	14	GGGGATGTAGCTCAGATGGTGGA
t0063068	20	14	GCGAGAGGTACGGGGATCGA
t0063100	20	14	GCGGTCGTGGCGGAATTGGT
t0063119	23	14	CTCGTGAGAGGGCGTGGGTTCAT
t0063176	23	14	GGGGGGGTAGCTCATATGGTAGA
t0063186	20	14	CACACTGAAGGTCTCCGGTT
t0063374	26	14	GGGGCCGTAGCTCAGCTGGGAGAGCG
t0063400	24	14	AGATTTAGGCTCTGGTCCGAAAGG
t0063470	22	14	TCCATTGTCGTCTAGTCCGGTA
t0063493	21	14	GCGGGGTAGAGGAATTGGTCA
t0063633	29	14	TTGGTAGAGCTGAGGACTGTAGATCCTTA
t0063681	22	14	ATTTGAAATCTGTTGGGCTTCG
t0063726	20	14	CTGGTAGAGCAGAGGACTGA
t0063771	27	14	GGTTCTATGGTCTAGCGGTTAGGACAT
t0063970	24	13	GGGGTTGTAGCTCAAATGGTATAG
t0064090	27	13	GTCGGGATAGCTCAGCTGGTAGAGCAG
t0064213	22	13	GGTGCTGTGGTGTAGTGGTTAT
t0064358	18	13	CGAGAGGTACGGGGATCG
t0064366	20	13	ACACTGAAGGTCTCCGGTTC
t0064375	22	13	GGGAGTGTAGCTCATATGGTAG
t0064476	22	13	GGGGGGGTAGCTCATATGGTAG
t0064530	25	13	GCGTTTGTAGTCCAACGGTCAGGAT
t0064635	20	13	TTGGTAGAGCGGAGGACTGT
t0064814	22	13	GGGATTGTAGCTCAAATGGTAG
t0064888	19	13	GCGGGGATAACTCAGTTGG
t0064972	27	13	TTGGTAGAGCGGAGGACTGTAGATCCT
t0064975	20	13	GGGGATATAGCTCAGATGGT
t0064982	19	13	AGCAGAAGGCCGTAGGTTC
t0065044	21	13	GGGGGTGTAGCTCAGATGGTA
t0065045	21	13	GTTGGTTAGGATACTCGGCTC
t0065049	27	13	GCGGATGTGGCGAAATTGGTAGACGTG
t0065102	21	13	GGAGAGATGGCCGAGTGGTTG
t0065130	19	13	GGAGAGATGGCCGAGTGGT
t0065221	20	13	TCTAGCGGTTAGGACATTGG
t0065227	27	13	GCGGTCGTGGCGGAACTGGTAGACGCG
t0065241	26	13	CTAGTTGGTTAGGATACTCGGCTCTC
t0065258	24	13	ACATTGGACTCTGAATCCAGTAAC
t0065390	22	13	AGGTGGTTAGGATACTCGGCTC
t0065409	19	13	AGATTTAGGCTCTGGTCCG
t0065419	20	13	GGAGATGTAGCTCAAATGGT
t0065448	27	13	TGGACTCTGAATCCAGTAACCCGAGTT
t0065454	20	13	TGGTAGAGCGGAGGACTGTA
t0065457	28	13	GCGCCTGTAGCTCAACGGATAGAGCATC

t0065564	26	13	GAGCGGAGGACTGTAGATCCTTAGGT
t0065802	25	13	GACTTCTAATCAGGCGATTGTGGGT
t0065916	20	13	GAGGATGTAGCTCAGATGGT
t0065955	24	13	GGGGATGGAGCTCAGATGGTAGAG
t0066037	22	13	AGCTGGTTAGGATACTCGGCTC
t0066119	22	13	GGTTCCATGGTCTAGCGGTTAG
t0066177	19	13	GTCTGGTGTAGTTGGTTAT
t0066205	22	13	GCGGAAGCGTGGTGGGCCCATA
t0066210	27	13	AGAGCTGAGGACTGTAGATCCTTAGGT
t0066386	19	13	TCAGGCGATTGTGGGTTCG
t0066534	22	13	GCCCGTCTAGCTCAGTTGGTAG
t0066604	23	13	GGTGCTGTGGTGTAGTGGTTATC
t0066640	22	13	TAGGATACTCGGCTCTCACCCG
t0066719	25	13	TGGTAGAGCATTTGACTGCACATCA
t0066802	21	13	TGCGAGAGGTACGGGGATCGT
t0066884	26	13	GTCTGGGTGGTGTAGTCGGTTATCAC
t0066901	22	13	AGCAGAAAGTCGTAGGTTCGGC
t0067077	24	13	GCGGGGTAGAGGAATTGGTCAACT
t0067100	26	13	GGGTTCGTGCCCCACGGTGGGCGCCA
t0067102	20	13	AGCGGGAGATTGTGGGTTCG
t0067140	24	13	GGATGGATGTCTGAGCGGTTGAAA
t0067217	22	13	GGTTAGGATACTCGGCTCTCAC
t0067269	29	13	GGGGCTGTAGCTCAGATGGGCGAGCGCCG
t0067273	22	13	TTGAAATCTGTTGGGCTTCGCC
t0067274	18	13	GGGGATGTAGCTCATGGT
t0067298	25	13	GCGCCTGTAGCTCAGTGGACAGAGC
t0067344	20	13	GCGGATGTAGCTCAGATGGT
t0067486	26	13	GGGGGTGTAGCTCATATGGTAGAGAA
t0067517	25	13	GGTGTCGTGGGGTAGTTGGTTATCA
t0067590	25	13	AGCGGGGGAGAGGAATTGGTCAACT
t0067659	26	13	TGGTTAGGATACTCGGCTCTCACCCG
t0067693	21	13	TGGGGTGTAGCTCATATGGTA
t0067819	25	13	AGTTGGTAGAGCTGAGGACTGTAGA
t0067860	22	13	GATTCGAAATCTGTTGGGCTTT
t0067943	26	13	GGGGATGTAGCTCAAATGGTAGAGCT
t0068064	22	13	ACTTCTAATCAGGCGATTGTGG
t0068080	23	13	TCAGTGGTAGAGCATTTGACTGC
t0068112	25	13	GGGGGTGTAGCTCATATGGTAGAAA
t0068241	25	13	AGGGATATAACTCAGCGGTAGAGTG
t0068309	22	13	GACCGCATAGCGCAGTGGATTA
t0068330	23	13	TGGGTTGTAGCTCAAATGGTAGA
t0068335	23	13	CTCAGTTGGTAGAGCGGAGGACT
t0068480	22	13	TTCGAATCCTGCTGTCGACGCC
t0068598	28	12	GCGCGAGTGGCGGAATAGGTAGACGCGC
t0068602	26	12	GCGTTTGTAGTCCAACGGTTCGGATA
t0068624	22	12	GTTGGTTAGGATACTCGGCTCT
t0068668	22	12	GGGGATGTAGCTCAGATGGTCA
t0068704	20	12	GGTAGAGCATTTGACTGCAT
t0068816	27	12	ATCAGAGTGGCGCAGCGGAAGCGTGGT
t0069169	24	12	GGGGATGTAGCTTAGATGGTAGAG

t0069358	24	12	GGGTGTATAGCTCAGTTGGTAGAG
t0069415	21	12	TGGTAGAGCATTTGACTGCAT
t0069437	22	12	AGATTTAGGCTCTGGTCCGAAA
t0069450	25	12	AGATTCGAAATCTGTTGGGCTTTGC
t0069490	21	12	CTGGTAGAGCAGAGGACTGAA
t0069533	25	12	ACGGACTGTAAATTCGTTGACGATA
t0069564	23	12	ATATTTGAACCCACAACCTTGAG
t0069592	20	12	GGAAATGTAGCTCAGATGGT
t0069646	22	12	AGGGATGTAGCTCAGATGGTAG
t0069768	22	12	GCGGGGATAACTCAGTTGGGAG
t0069822	23	12	GTTGTCGTGGTGTAGTTGGTTAT
t0070147	23	12	GATTTGAAATCTGTTGGGCTTCG
t0070267	24	12	GTGGTAGAGCATTTGACTGCATCA
t0070307	25	12	GCGGATATGGTCGAATGGTACAATT
t0070316	21	12	CCGAAAGGGCGTGGGTTCATC
t0070406	26	12	AGAGCGGAGGACTGTAGATCCTTAGG
t0070444	25	12	TCCGTTGTAGTCTAGGTGGTTAGGA
t0070518	21	12	CTAGGTGGTTAGGATACTCGG
t0070577	28	12	GGGGATGTAGCTCAGATGGTAGAGCTCT
t0070610	20	12	AAGCAGAAGGCCGTAGGTTC
t0070663	20	12	CCGACCTTAGCTCAGTTGGT
t0070689	27	12	GTCGCTTTGGCCGAGTGGTTAAGGCTT
t0070825	22	12	GTCGTTGTAGTATAGTGGTGCG
t0070986	24	12	GACATTGGACTCTGAATCCAGTAA
t0071181	25	12	CTCAGTTGGTAGAGCTGAGGACTGT
t0071240	23	12	GGTAGAGCGGAGGACTGTAGATC
t0071328	18	12	TCCATTGTCGTCTAGTCC
t0071335	25	12	GCGGGTGTAGCTCAGGGGTAGAGCA
t0071644	19	12	TCGAAATCTGTTGGGCTTT
t0071657	24	12	GATTTGAAATCTGTTGGGCTTCGC
t0071685	21	12	GCGCCTGTAGCTCAGTGGATA
t0071692	26	12	GCGGGCGTAGCTCAGGGGTAGAGCAC
t0071755	25	12	GGTGATGTAGCTCAAACGGTAGAGC
t0071894	22	12	TTTGAAATCTGTTGGGCTTCGC
t0072104	24	12	GTGGTCGTGCCGGAGTGGTTATCG
t0072304	22	12	GGGCATTTGGTCTAGTGGTATG
t0072330	25	12	GTCTGGGTAGTGTAGTTGGTTATCA
t0072491	23	12	GCGGAAGCGTGGTGGGCCCATAA
t0072513	22	12	GGGGGTGTAGCTCATGGTAGAG
t0072548	27	12	GCGGGCGTGGCGGAATTGGTAGACGCA
t0072556	28	12	GGGTTGCTAACTCAACGGTAGAGTACTC
t0072614	18	12	AGCGGGAGATTGTGGGTT
t0072679	28	12	ACGGACTGTAAATTCGTTGACGATATGT
t0072711	24	12	GGGAATGTAGCTCAAATGGTAGAG
t0072770	25	12	GTAGAGCAGAGGACTGTAGATCCTT
t0072828	25	12	GGGGATGTAGCTCAGATGGTAGAGA
t0072964	24	12	GGGGATGTAGCTCAGATGGTAGGC
t0072986	22	12	GGTAGAGCGGAGGACTGTAGAT
t0072995	23	12	AAGCAGAAGGCCGTAGGTTCGAC
t0073112	25	12	GCGGACGTAGCTCAGTTGGTAGAGC

t0073117	22	12	GCGGGGTAGAGGAATTGGTCAA
t0073197	23	12	GGGATGTAGCTCAAACGGTAGAG
t0073237	20	12	TCCGAAAGGGCGTGGGTTCA
t0073310	19	12	TTGCGAGAGGTACGGGGAT
t0073317	27	12	GGCAGAGCGGAGGACTGTAGATCCTTA
t0073489	23	12	GGGGTTGTAGCTCAAATGGTATA
t0073581	19	12	GCGGGGTAGAGGAATTGGT
t0073647	29	12	AGGGCTATAGCTCAGTTAGGTAGAGCACC
t0073764	23	12	CTAGGTGGTTAGGATACTCGGCT
t0073843	25	12	GCCCAGATGGCGGAATTGGTAGACG
t0073904	23	12	TGGTAGAGCATTTGACTGCACAT
t0073924	24	12	GGGCGTTTGGTCTAGTGGTATGAT
t0073961	23	12	TCCATTGTCGTCTAGTCCGGTCA
t0073995	23	12	GACTTCTAATCAGGCGATTGTGG
t0074112	22	11	CTCAGTTGGTAGAGCTGAGGAC
t0074120	24	11	GGTTCCATGGTCTAGCGGTTAGGA
t0074196	21	11	CTCGAGAGAGGGCGTGGGTTC
t0074211	27	11	GGTGTCGTGGTGTAGTTGGTTATCACA
t0074270	25	11	GATTTGAAATCTGTTGGGCTTCGCC
t0074289	22	11	TGGTAGAGCGGAGGACTGTAGA
t0074337	21	11	AGCAGAAAGTCGTAGGTTCGT
t0074344	22	11	CGGAAGCGTGGTGGGCCCATAA
t0074395	22	11	CACACTGAAGGTCTCCGGTTCG
t0074546	19	11	CCGGAGCGGGAGATTGTGG
t0074587	21	11	GGGGATGTAGCTCAGTTGGTA
t0074595	19	11	GAGCGGGAGATTGTGGGTT
t0075037	28	11	TAGGACATTGGACTCTGAATCCAGTAAC
t0075199	22	11	AGCTGGTAGAGCAGAGGACTGA
t0075572	23	11	TGGGGTGTAGCTCATATGGTAGA
t0075604	23	11	GGGGATATAGCTCAGTTGGTAGA
t0075620	20	11	TAGCGGTTAGGACATTGGAC
t0075632	28	11	AGCGGAAGCGTGGTGGGCCCATAACCCA
t0075872	22	11	GGGGATGTAGCTCAGATGGAAG
t0075980	23	11	CCGGAGCGGGAGATTGTGGGTTC
t0076055	24	11	GGGATTGTAGCTCAAATGGTAGAG
t0076058	22	11	GGTCTCGTGGTGTAGTTGGTTA
t0076091	21	11	TCGAATCCTGCTGTCGACGCC
t0076185	23	11	GTAGAGCGGAGGACTGTAGATCC
t0076186	20	11	TCAAGGCAGTGGATTGTGAA
t0076260	24	11	GGGGTTGTAGCTCATATGGTAGAG
t0076351	24	11	GGGTATGTAGCTCAGATGGTAGAG
t0076426	20	11	GCGGAAATAGCTTAATGGTA
t0076453	24	11	ACACACTGAAGGTCTCCGGTTCGT
t0076648	25	11	GACCGCATAGCGCAGTGGATTAGCG
t0076799	20	11	TAAGCAGAAGGCCGTAGGTT
t0076834	20	11	GCAGAAAGTCGTAGGTTCGT
t0076850	25	11	TCAGTTGGTAGAGCTGAGGACTGTA
t0076864	20	11	TGCGAGAGGTACGGGGATCG
t0076976	22	11	GGGCGTTTGGTCTAGTGGTATG
t0077103	22	11	GGGTCCATAGCTCAGTGGTAGA

t0077115	24	11	GGGGATGTAGCTCAAACGGTAGTT
t0077262	23	11	GGGGTGTAGCTCATATGGTAGAG
t0077652	25	11	GGGGATGTAGCTCAAATGGTAGAAA
t0077674	22	11	GGGGATGTAGCTCAATGGTAGA
t0077778	23	11	TTTGAAATCTGTTGGGCTTCGCC
t0077857	29	11	GGGGCTGTGGCGCAGCTGGTAGCGCATCT
t0077865	22	11	GGTAGAGCTGAGGACTGTAGCT
t0078022	20	11	GGGGGTGTAGCTCATGGTAG
t0078068	23	11	TGGTAGAGCATTTGACTGCATAT
t0078204	24	11	TGGTTAGGATACTCGGCTCTCACC
t0078216	23	11	TAGAGCGGAGGACTGTAGATCCT
t0078351	22	11	ACACACTGAAGGTCTCCGGTTC
t0078463	25	11	GGAGTTGTAGCTCAAATGGTAGAGC
t0078732	24	11	TGGGATGTAGCTCAAACGGTAGAG
t0078754	20	11	GGGTTGTAGCTCAAATGGTA
t0078771	21	11	TCTAGCGGTTAGGACATTGGA
t0078772	18	11	TAGCGGTTAGGACATTGG
t0078855	21	11	AGCGGGGTAGAGGAATTGGTT
t0078882	28	11	GCGCGGGTGGCGGAATAGGTAGACGCGC
t0079055	21	11	GGGCCTGTAGCTCAGAGGATT
t0079066	21	11	GGGGATGTAGCTCAGATGGAA
t0079122	24	11	TGGGGTGTAGCTCATATGGTAGAG
t0079317	19	11	GAGCGGAGGACTGTAGATC
t0079333	24	11	GCGGACGTAGCTCAGTTGGTAGAG
t0079338	21	11	AGTCCCGTAGCTCAGTTGGTT
t0079392	24	11	GCGCCTGTAGCTCAGTGGGAGAGC
t0079418	24	11	GAGCGGAGGACTGTAGATCCTTAG
t0079517	28	11	ATTGGACTCTGAATCCAGTAACCCGAGT
t0079530	19	11	GGATGTAGCTCAGATGGTA
t0079578	21	11	CCGAAAGGGCGTGGGTTCATA
t0079712	23	11	GGGATGTAGCTCAAATGGTAGAG
t0079722	21	11	GCTGGTTAGGATACTCGGCTC
t0079780	25	11	TTGGTAGAGCTGAGGACTGTAGATC
t0079790	23	11	GGGGCTGTAGCTCAGATGGGAGA
t0079807	22	11	GGGGCTATAGCTCAGGCGGTTA
t0079813	22	11	GGGGATGTATCTCAGATGGTAG
t0079902	23	11	GTGGTAGAGCATTTGACTGCACA
t0079911	23	11	GCGGGGTAGAGGAATTGGTCAAC
t0079969	22	11	TTGCGAGAGGTACGGGGATCGT
t0080208	26	11	ATTGGACTCTGAATCCAGTAACCCGA
t0080269	19	11	GTTAGGATACTCGGCTCTC
t0080284	19	11	GCGGGTATAGTTTAGTGGT
t0080315	22	11	GCGGATGTAGCTCAGATGGTAG
t0080332	22	11	GCGCATCTGGTGTAGTGGTATC
t0080611	19	10	CTGGTAGAGCAGAGGACTG
t0080847	28	10	ACGGACTGTAAATTCGTTGACGATATGA
t0081009	22	10	GGGAATGTAGCTCAAATGGTAG
t0081012	22	10	CCGACCTTAGCTCAGTTGGCCG
t0081037	22	10	CGGAATGTAGCTCAGATGGTAG
t0081051	28	10	CGCGGGGTAGAGCAGTTTGGTAGCTCGC

t0081092	24	10	ATGATTGGAAATCATGTGGGCTTT
t0081246	24	10	ATTTGAAATCTGTTGGGCTTCGCC
t0081439	24	10	CAGTTGGTAGAGCGGAGGACTGTA
t0081521	23	10	GGGGATGTAGCTCAGATGTAGAG
t0081627	27	10	GTGGTAGAGCATTTGACTGCACATCAA
t0081646	22	10	GTGGCTGTAGTTTAGTGGTTAG
t0081843	22	10	GGGGATGTAGCTCAAAGGTAGA
t0082034	22	10	GCGGACGTAGCGCAGTTGGTAG
t0082117	25	10	GACATTGGACTCTGAATCCAGTAAC
t0082121	21	10	GTCTGTGGTGTAGTTGGTTAT
t0082135	25	10	AGATTGAGGTTCTGGTCCGAAAGGG
t0082241	19	10	GGGGATGTAGCTCAGGGTA
t0082381	28	10	GGTGTCGTGGTGTAGTTGGTTATCACAT
t0082394	26	10	GCGGGTGGCGGAATAGGTAGACGCGC
t0082462	27	10	GCGTTTGTAGTCCAACGGTTCGGATAA
t0082483	18	10	AGGGATGTAGCGCAGCTT
t0082512	22	10	GTCGCTTTGGCCGAGTGGTTAA
t0082554	21	10	CAAGGCAGTGGATTGTGAATC
t0082614	24	10	CCGGAGCGGGAGATTGTGGGTTCG
t0082854	21	10	AGCAGAAGGCCGTAGGTTCGG
t0082890	19	10	GCCCCTATAGCTCAGTGGT
t0082926	21	10	GTCCGAAAGGGCGTGGGTTCA
t0082939	24	10	AGGGATGTAGCTCAGATGGTAGAG
t0082967	22	10	AGCGGAAGCGTGGTGGGCCCAT
t0083002	22	10	GCGGACGTAGCGCAGCTGGTAG
t0083050	20	10	AGCAGAAAGTCGTAGGTTCG
t0083102	26	10	GGGCCTGTAGCTCAGTTGGCAGAGCA
t0083156	27	10	TCTAGTTGGTTAGGATACTCGGCTCTC
t0083303	19	10	TCAGCTGGTAGAGCATGGC
t0083359	20	10	GCGGATGTGGCCAAGTGGAA
t0083402	22	10	AGCGGGGTAGAGGAATTGGTCC
t0083406	22	10	TCGTGAGAGGGCGTGGGTTCAT
t0083459	24	10	GTGGCTGTAGTTTAGTGGTTAGAA
t0083604	27	10	CGCGGGGTAGAGCAGTTTGGTAGCTCG
t0083676	26	10	GTGGTAGAGCATTTGACTGCACATCA
t0083720	22	10	ATCAGAGTGGCGCAGCGGAAGC
t0083756	22	10	GGGGATATAGCTCAGATGGTAG
t0083983	23	10	TCAGTTGGTAGAGCGGAGGACTG
t0084036	28	10	GGGGATGTAGCTCAGATGGTAGAGCCTC
t0084365	25	10	GGGGGTGTAGCTCATATGGTAGAGT
t0084529	28	10	GTGGGTTCGTGCCCCACGGTGGGCGCCA
t0084543	20	10	CCAAGCAATAGATCCGGGTT
t0084645	19	10	GTTGGTAGAGCGGAGGACT
t0084875	24	10	GGGATGTAGCTCAGATGGTAGAGC
t0084908	23	10	GCGGAAATAGCTTAATGGTAGAG
t0085046	26	10	AGGACATTGGACTCTGAATCCAGTAA
t0085155	22	10	GGGGATATGGCGAAATTGGTCG
t0085493	24	10	AGTGGTAGAGCATTTGACTGCATA
t0085498	19	10	GGTAGACGCTACGGACTTA
t0085587	20	10	GGACTAGACGACAATGGAAA

t0085611	24	10	GGGGATTTAGCTCAGATGGTAGAG
t0085619	19	10	GGTAGAGCATTTGACTGCA
t0085689	23	10	GGGGATGTAGCTCAAACGGTAGC
t0085809	24	10	GGTGGCTGTACTTTAGTGGTTAGA
t0085832	21	10	TCCAAGCAATAGATCCGGGTT
t0085856	21	10	GTCGCTTTGGCCGAGTGGTTA
t0085967	20	10	CGAAAGGGCGTGGGTTCAAA
t0086376	21	10	TGACACACTGAAGGTCTCCGG
t0086625	24	10	GGGGATGTAGCTCAGATGGTACAG
t0086626	25	10	GGTGGCTGTATTTTAGTGGTTAGAA
t0086678	21	10	GGGGATGTAGCTCATGGTAGA
t0086735	21	10	GCAGAAAGTCGTAGGTTCGGC
t0086764	21	10	GCGGGTATAGTTTAGTGGTAA
t0086873	24	10	GGGGATGTAGCTCAGATGGGAGAG
t0086940	18	10	GGGATGTAGCTCAGATGG
t0086987	19	10	TCTAGCGGTTAGGACATTG
t0087155	27	10	GGCCTGATGGTCTAGCGGTATGATTCT
t0087196	22	10	GGGGATGTAGCTCAGAGGGTAG
t0087250	20	10	GTTGGTAGAGCGGAGGACTG
t0087276	22	10	TCCGGTATGGTGTAGTGGCTAA
t0087363	20	10	GCGCCTGTAGCTCAGTGGAC
t0087404	27	10	GGGGATGTAGCTCAGATGGTAGAGCCT
t0087485	22	10	GGGCCTGTAGCTCAGAGGATAG
t0087503	19	10	GCGGATATGGTCGAATGGA
t0087542	25	10	GGAGATGTAGCTCAAATGGTAGAGC
t0087719	22	10	GGGGATGTAGCTCAGATGGTAC
t0087724	26	10	GGGGATGTAGCTCAGATGGTAGAGAA
t0087768	22	10	TAGAGCGGAGGACTGTAGATCC
t0087828	21	10	TCGTGAGAGGGCGTGGGTTCA
t0087839	24	10	GTAGAGCATTTGACTGCAGATCAA
t0087842	21	10	GTGATGTAGCTCAGATGGTAG
t0087896	28	10	TGGTAGAGCAGAGGACTGTAGATCCTTA
t0087980	23	10	CTCCGGAGCGGGAGATTGTGGGT
t0088102	24	10	CAGCGGAAGCGTGGTGGGCCCATA
t0088314	24	10	GACCGCATAGCGCAGTGGATTAGC
