# Supplementary material for: Transfer RNA Derived Small RNAs Targeting Defense Responsive Genes Are Induced during Phytophthora capsici Infection in Black Pepper (Piper nigrum L.)
Source: Front Plant Sci. 2016 Jun 1;7:767. doi: 10.3389/fpls.2016.00767 (PMC4887504; doi:10.3389/fpls.2016.00767)
Supplement: Supplementary file 4 [file DataSheet3.PDF]

Transfer RNA derived small RNAs targeting defence responsive genes are induced during *Phytophthora capsici* infection in black pepper (*Piper nigrum* L.)

Supplementary Data 3

TRNA mapped small RNAs from *Phytophthora capsici* infected root library (Pn IR) of black pepper

Seq. ID	Length	Read Count	Sequence
t0000005	20	74230	GGGGATGTAGCTCAGATGGT
t0000006	21	66777	GGGGATGTAGCTCAGATGGTA
t0000009	22	41290	GGGGATGTAGCTCAGATGGTAG
t0000010	23	33826	GGGGATGTAGCTCAGATGGTAGA
t0000016	24	27481	GGGGATGTAGCTCAGATGGTAGAG
t0000018	19	25249	GCGTTTGTAGTCCAACGGT
t0000026	19	19598	GGGGATGTAGCTCAGATGG
t0000031	22	15048	GGGGATGTAGCTCAAACGGTAG
t0000039	23	11540	GGGGATGTAGCTCAAACGGTAGA
t0000041	22	11375	GGTGTCGTGGTGTAGTTGGTTA
t0000048	21	9864	GGTGTCGTGGTGTAGTTGGTT
t0000053	24	9133	GGGGATGTAGCTCAAACGGTAGAG
t0000063	23	6941	GGTGTCGTGGTGTAGTTGGTTAT
t0000067	25	6322	GGGGATGTAGCTCAGATGGTAGAGC
t0000071	24	5752	GGGGGTGTAGCTCATATGGTAGAG
t0000077	25	5639	GGTGTCGTGGTGTAGTTGGTTATCA
t0000084	20	5371	GGTGTCGTGGTGTAGTTGGT
t0000092	24	5032	GGGGTTGTAGCTCAAATGGTAGAG
t0000093	24	4939	GGTGTCGTGGTGTAGTTGGTTATC
t0000101	22	4630	GGGGTTGTAGCTCAAATGGTAG
t0000103	23	4600	GGGGTTGTAGCTCAAATGGTAGA
t0000109	23	4427	GGGGGTGTAGCTCATATGGTAGA
t0000111	21	4405	GGGGATGTAGCTCAGATGGTC
t0000115	20	4300	GCGTTTGTAGTCCAACGGTT
t0000127	23	3885	AGCGGGGTAGAGGAATTGGTCAA
t0000128	24	3882	GGGGATGTAGCTCAAATGGTAGAG
t0000129	22	3863	GGGGGTGTAGCTCATATGGTAG
t0000133	22	3721	GGGGATGTAGCTCAGATGGTCG
t0000139	23	3579	GGGGATGTAGCTCAAATGGTAGA
t0000142	21	3483	GGGGGTGTAGCTCATATGGTA
t0000145	21	3244	AGCGGGGTAGAGGAATTGGTC
t0000151	22	3106	GGGGATGTAGCTCAAATGGTAG
t0000163	21	2861	GGGGATGTAGCTCAAATGGTA
t0000167	25	2811	GGGGATGTAGCTCAAACGGTAGAGC
t0000177	21	2619	GCGTTTGTAGTCCAACGGTTA
t0000195	21	2357	GGTGGCTGTAGTTTAGTGGTA
t0000196	22	2322	AGCGGGGTAGAGGAATTGGTCA
t0000200	22	2297	GTCTGGGTGGTGTAGTTGGTTA
t0000203	22	2275	GGTGGCTGTAGTTTAGTGGTTA
t0000206	28	2243	GCGGACGTAGCTCAGTTGGTAGAGCGCA
t0000218	20	2153	GCGTTTGTAGTCCAACGGTA
t0000233	21	1940	GGGATTGTAGTTCAATCGGTC
t0000235	21	1932	GTCTGGGTGGTGTAGTTGGTT
t0000236	20	1930	GGTGGCTGTAGTTTAGTGGT
t0000238	20	1916	AGCGGGGTAGAGGAATTGGT
t0000248	24	1853	GGTGGCTGTAGTTTAGTGGTTAGA

t0000249	25	1841	GGTGTCGTCGTGTAGTTGGTTATCA
t0000253	25	1821	GGGGGTGTAGCTCATATGGTAGAGC
t0000255	22	1817	GGTGTCGTCGTGTAGTTGGTTA
t0000265	21	1747	GGTGGCTGTAGTTTAGTGGTT
t0000268	23	1722	GGTGGCTGTAGTTTAGTGGTTAG
t0000269	29	1706	GGGGCTGTAGCTCAGCTGGGAGAGCACCT
t0000270	26	1696	GGTGGCTGTAGTTTAGTGGTTAGAAT
t0000281	20	1650	GTCTGGGTGGTGTAGTTGGT
t0000282	29	1648	GCGGACGTAGCTCAGTTGGTAGAGCGCAA
t0000298	24	1563	GGTGTCGTCGTGTAGTTGGTTATC
t0000299	29	1560	CGGGGTGTAGCTTAGCCTGGTAGAGCGCT
t0000301	19	1557	GTCGTTGTAGTATAGTGGT
t0000312	29	1515	GGGTCTGTAGCTCAGTCGGTTAGAGCACC
t0000317	25	1465	GGTGGCTGTAGTTTAGTGGTTAGAA
t0000322	25	1449	AGCGGGGTAGAGGAATTGGTCAACT
t0000354	28	1310	GGGGCTGTAGCTCAGTTGGGAGAGCGCT
t0000379	19	1225	GCGGGGATAGCTCAGTTGG
t0000381	22	1213	GTCTGGGTGGTGTAGTCGGTTA
t0000426	20	1093	GGGGATGTAGCTCAAATGGT
t0000456	20	1058	GGGGGTGTAGCTCATATGGT
t0000489	25	1002	GGGGTTGTAGCTCAAATGGTAGAGC
t0000490	30	1001	CGGGGTGTAGCTTAGCCTGGTAGAGCGCTA
t0000492	22	1000	GGTGTCGTTGTGTAGTTGGTTA
t0000499	23	983	GGTGTCGTCGTGTAGTTGGTTAT
t0000529	28	938	GGGTCTGTAGCTCAGTCGGTTAGAGCAC
t0000530	20	937	GGGATTGTAGTTCAATCGGT
t0000560	19	891	AGCGGGGTAGAGGAATTGG
t0000568	28	874	GGCTTTGTGGTCTAGCGGTATGATTCTC
t0000571	21	870	GGAGATGTAGCTCAGATGGTA
t0000576	21	866	TCCATTGTCGTCTAGTCCGGT
t0000578	20	859	GGAGATGTAGCTCAGATGGT
t0000601	29	836	GGAGGTATGGCTGAGTGGCTTAAGGCATT
t0000612	25	826	GGGGATGTAGCTCAAATGGTAGAGC
t0000634	19	799	GGTGTCGTGGTGTAGTTGG
t0000649	19	781	GGTGGCTGTAGTTTAGTGG
t0000664	20	772	GCGGGGATAGCTCAGTTGGG
t0000679	24	756	AGCGGGGTAGAGGAATTGGTCAAC
t0000708	25	725	GGTGTCGTTGTGTAGTTGGTTATCA
t0000725	23	711	GTCTGGGTGGTGTAGTTGGTTAT
t0000740	29	702	GGGGCTGTAGCTCAGTTGGGAGAGCGCTA
t0000748	22	696	TCCATTGTCGTCTAGTCCGGTT
t0000824	22	646	GGGATTGTAGTTCAATCGGTCA
t0000865	26	621	GGGTCTGTAGCTCAGTCGGTTAGAGC
t0000871	21	617	TGGGATGTAGCTCAGATGGTA
t0000885	22	609	GTGGACGTGCCGGAGTGGTTAT
t0000897	28	604	GGTCTGGTGGTCTAGCGGTATGATTCTC
t0000899	22	601	GGAGATGTAGCTCAGATGGTAG
t0000909	21	592	GCGTTTGTAGTCCAACGGTAA
t0000918	26	591	GGTGTCGTGGTGTAGTTGGTTATCAC
t0000923	27	588	GCCCAGGTGGCGGAATTGGTAGACGCA

t0000951	31	573	GCACCAGTGGTCTAGTGGTAGAATAGTACCC
t0000965	21	567	GTCTGGGTGGTGTAGTTGGTC
t0000966	23	567	GGTGTCGTTGTGTAGTTGGTTAT
t0000972	27	564	GGGTCTGTAGCTCAGTCGGTTAGAGCA
t0000999	25	553	GGTGGCTGTAGTTTAGTGGTAAGAA
t0001038	24	534	GGTGTCGTTGTGTAGTTGGTTATC
t0001040	28	533	AGCGGGGTAGAGGAATTGGTCAACTCAT
t0001064	27	525	GCACTCGTAGCTTAACGGATAGAGCAT
t0001069	22	524	GTCTGGGTGGTGTAGTTGGTCA
t0001158	28	494	GGGGCTGTAGCTCAGCTGGGAGAGCACC
t0001173	23	490	GGAGATGTAGCTCAGATGGTAGA
t0001174	25	489	GGGGATGTAGCTCAGATGGTCGAGC
t0001183	19	484	GGGATTGTAGTTCAATCGG
t0001193	30	482	GGGGCTGTAGCTCAGCTGGGAGAGCACCTG
t0001206	20	478	TGGGATGTAGCTCAGATGGT
t0001218	29	474	GGGGCTGTAGCTCAGTTGGGAGAGCGCCT
t0001226	30	471	GCACCAGTGGTCTAGTGGTAGAATAGTACC
t0001317	22	446	TGTGTCGTGGTGTAGTTGGTTA
t0001320	31	445	GGGGCTGTAGCTCAGCTGGGAGAGCACCTGC
t0001377	20	432	GGGATGTAGCTCAGATGGTA
t0001402	20	426	GCTGGAATAGCTCAGTTGGT
t0001417	20	423	GCGGGGTAGAGGAATTGGTC
t0001454	28	413	CGGGGTGTAGCTTAGCCTGGTAGAGCGC
t0001472	29	409	GGGATTGTAGTTCAATCGGTCAGAGCACC
t0001479	22	407	GGAGAGATGGCTGAGTGGACTA
t0001508	27	402	GCGGACGTAGCTCAGTTGGTAGAGCGC
t0001515	23	399	GCGGGGATAGCTCAGTTGGGAGA
t0001561	22	389	GTGGTCGTGCCGGAGTGGTTAT
t0001570	22	388	GCGGGGATAGCTCAGTTGGGAG
t0001578	23	387	TCCATTGTCGTCTAGTCCGGTTA
t0001580	24	386	GGAGATGTAGCTCAGATGGTAGAG
t0001611	25	379	GTCGTTGTAGTATAGTGGTAAGTAT
t0001616	26	378	GGGGATGTAGCTCAGATGGTAGAGCT
t0001628	28	376	GCCCGGATGGTGGAATGCAGACACGGCG
t0001634	27	375	GGCTTTGTGGTCTAGCGGTATGATTCT
t0001682	23	367	GTCTGGGTGGTGTAGTCGGTTAT
t0001688	30	366	GCGGACGTAGCTCAGTTGGTAGAGCGCAAC
t0001717	22	360	GGGGGTGTAGCTCATATGGTCG
t0001720	23	360	GGGGGTGTAGCTCATATGGAAGA
t0001759	24	351	GTCTGGGTGGTGTAGTTGGTTATC
t0001793	30	346	GGGCCTGTAGCTCAGAGGATTAGAGCACGT
t0001794	22	346	TGGGATGTAGCTCAGATGGTAG
t0001835	19	340	GGGATGTAGCTCAGATGGT
t0001847	21	337	GCGGGGATAGCTCAGTTGGGA
t0001862	20	335	GTTGAGATGGCCGAGTTGGT
t0001865	20	335	GGTGATGTAGCTCAGATGGT
t0001918	26	326	GGGGATGTAGCTCAAACGGTAGAGCT
t0001926	25	326	GTCTGGGTGGTGTAGTTGGTTATCA
t0001937	22	324	GTCTGGGTTGTGTAGTTGGTTA
t0001957	28	321	GCACTCGTAGCTTAACGGATAGAGCATC

t0001971	21	318	GGAGAGATGGCTGAGTGGACT
t0001983	21	316	GGTGATGTAGCTCAGATGGTA
t0002155	20	298	GCGTTTGTAGTCCAACGGTC
t0002156	26	298	GGGGCTGTAGCTCAGCTGGGAGAGCA
t0002179	27	296	GGTCTGGTGGTCTAGCGGTATGATTCT
t0002219	27	291	AGCGGGGTAGAGGAATTGGTCAACTCA
t0002244	20	287	GTCGTTGTAGTATAGTGGTA
t0002249	18	287	GTTGAGATGGCCGAGTTG
t0002276	27	284	GGAGGTATGGCTGAGTGGCTTAAGGCA
t0002278	28	284	GGGGCTGTAGCTCAGCTGGGAGAGCGCT
t0002357	22	277	GGGGATGTAGCTCAAATGGTCG
t0002358	28	277	GCGTGAATGGTTTAGTGGTAAAATTCTC
t0002438	24	270	TGTGTCGTGGTGTAGTTGGTTATC
t0002445	21	270	GGGGATGTAGCTCAGATGGTT
t0002473	19	267	GCGGGGTAGAGGAATTGGT
t0002487	19	266	GTCTGGGTGGTGTAGTTGG
t0002546	23	261	TGTGTCGTGGTGTAGTTGGTTAT
t0002584	25	258	GTCGTTGTAGTATAGTGGTGAGTAT
t0002605	23	256	TGGGATGTAGCTCAGATGGTAGA
t0002666	25	251	GGGTCTGTAGCTCAGTCGGTTAGAG
t0002728	19	246	GCGCCTGTAGCTCAGTGGA
t0002731	21	246	GGGATGTAGCTCAGATGGTAG
t0002736	23	246	GGAGAGATGGCTGAGTGGACTAA
t0002738	22	245	GGGGATGTAGCTCAGATGGTAT
t0002743	24	245	TGGGATGTAGCTCAGATGGTAGAG
t0002756	26	244	GCGTTTGTAGTCCAACGGTTAGGATA
t0002869	29	237	GGGGCTGTAGCTCAGCTGGGAGAGCGCTA
t0002884	24	236	GGGGGTGTAGCTCATATGGAAGAG
t0002912	26	234	GCGCGGATGGCGGAATTGGTAGACGC
t0002918	27	234	GGGATTGTAGTTCAATCGGTCAGAGCA
t0002926	21	233	TGTGTCGTGGTGTAGTTGGTT
t0002947	19	232	GGAGATGTAGCTCAGATGG
t0002950	28	231	GGTGTCGTGGTGTAGTTGGTTATCACTT
t0002964	21	231	GTCGTTGTAGTATAGTGGTAA
t0002967	21	230	GGGGGTGTAGCTCATATGGTC
t0002988	20	229	GCACCAGTGGTCTAGTGGTA
t0002992	24	229	GCGGGGATAGCTCAGTTGGGAGAG
t0003031	26	227	AGCGGGGTAGAGGAATTGGTCAACTC
t0003072	26	224	GCCCGGATGGTGGAATGCAGACACGG
t0003200	25	217	CGGGGTGTAGCGCAGCTTGGCAGCG
t0003231	22	214	GGTGATGTAGCTCAGATGGTAG
t0003288	28	211	GGGCGTTTGGTCTAGTGGTATGATTCTC
t0003321	26	210	GCGGATGTGGCGGAACTGGTAGACGC
t0003351	19	208	GTGGCTGTAGTTTAGTGGT
t0003380	30	207	GGGATTGTAGTTCAATCGGTCAGAGCACCG
t0003388	21	206	GTGTCGTGGTGTAGTTGGTTA
t0003394	27	206	GCGTTTGTAGTCCAACGGTTAGGATAA
t0003409	21	205	GCGTTTGTAGTCCAACGGTCA
t0003423	22	204	TCCGTTGTAGTCTAGTTGGTTA
t0003491	21	200	GGTGGCTGTAGTTTAGTGGTC

t0003506	29	199	GCGGCTGTAGCTCAGTTGGATAGAGTACT
t0003564	24	197	GGGGATGTAGCTCAGATGGTAGAT
t0003568	26	197	GCCCAGGTGGCGGAATTGGTAGACGC
t0003611	21	195	GGGGATGTAGCTCAAATGGTC
t0003615	18	195	GCGGGGATAGCTCAGTTG
t0003683	19	191	TGGGATGTAGCTCAGATGG
t0003698	28	190	GCCCAGGTGGCGGAATTGGTAGACGCAC
t0003814	25	185	TGTGTCGTGGTGTAGTTGGTTATCA
t0003823	28	185	GGAGGTATGGCTGAGTGGCTTAAGGCAT
t0003844	23	184	GTGGACGTGCCGGAGTGGTTATC
t0003857	29	183	GGTGGCTGTAGCTCAGTTGGTAGAGTCCC
t0003866	21	183	GCGTTTGTAGTCCAACGGTTC
t0003870	21	183	GCGGGGTAGAGGAATTGGTCA
t0003925	23	181	GGTGATGTAGCTCAGATGGTAGA
t0003932	27	181	GGGCGTTTGGTCTAGTGGTATGATTCT
t0003954	29	179	GCACCAGTGGTCTAGTGGTAGAATAGTAC
t0004040	27	176	CGGGGTGTAGCTTAGCCTGGTAGAGCG
t0004073	22	175	GGGATGTAGCTCAGATGGTAGA
t0004100	25	173	TCCATTGTCGTCTAGTCCGGTTAGG
t0004135	24	172	GGAGAGATGGCTGAGTGGACTAAA
t0004180	28	170	GGGCCTGTAGCTCAGTTGGGAGAGCGCT
t0004187	27	170	GGTAGAGCTGAGGACTGTAGATCCTTA
t0004219	28	169	GCACCAGTGGTCTAGTGGTAGAATAGTA
t0004259	29	168	GCCCGGATGGTGGAATGCAGACACGGCGA
t0004261	20	168	TCCATTGTCGTCTAGTCCGG
t0004279	20	167	GTCGATATGTCCGAGTGGTT
t0004294	20	166	TGTGTCGTGGTGTAGTTGGT
t0004295	28	166	GGAGAGATGGCTGAGTGGACTAAAGCTG
t0004302	27	166	GCGGACGTAGCGCAGCTGGTAGCGCAT
t0004344	24	165	GGGGATGTAGCTCAGATGGTAGAA
t0004405	25	163	GGGGGTGTAGCTCATATGGAAGAGC
t0004412	26	162	GGCGGATGTAGCCAAGTGGATCAAGG
t0004452	27	161	TCCATTGTCGTCTAGTCCGGTTAGGAT
t0004457	18	161	GCGTTTGTAGTCCAACGG
t0004461	27	161	TGGGTTCGTGCCCCACGGTGGGCGCCA
t0004506	30	160	GGGGATGTAGCTCAGATGGTAGAGCGCTCG
t0004538	27	159	TGGTAGAGCATTTGACTGCAGATCAAG
t0004540	29	158	GGGCCTGTAGCTCAGTTGGGAGAGCGCTA
t0004556	28	158	TGGTAGAGCATTTGACTGCAGATCAAGA
t0004561	27	158	GTCCGGGTGGCGGAATGGCAGACGCGC
t0004632	22	155	GGTGTCGTAGTGTAGTTGGTTA
t0004641	28	155	TGGTAGAGCTGAGGACTGTAGATCCTTA
t0004653	28	155	GGTGGCTGTAGCTCAGTTGGTAGAGTCC
t0004767	25	152	GGGGGTGTAGCTCATATGGTCGAGC
t0004866	28	149	TCCGGTATGGTGTAGTGGCTAACATTTC
t0004869	23	149	GTGGTCGTGCCGGAGTGGTTATC
t0004918	28	148	GCGGACGTAGCTCAGTTGGTCGAGCGCA
t0004942	26	147	GCGGGTGTAGTTCAATGGCAGAACAT
t0004999	25	145	GCGCGGATGGCGGAATTGGTAGACG
t0005018	28	145	GGGGCTGTAGCTCAGTTGGGAGAGCGCC

t0005035	27	144	GGAGAGATGGCTGAGTGGACTAAAGCG
t0005140	25	142	GTCTGGGTGGTGTAGTTGGTCATCA
t0005168	24	141	TCCATTGTCGTCTAGTCCGGTTAG
t0005183	26	141	TGGTAGAGCATTTGACTGCAGATCAA
t0005184	20	141	GTCGTTGTAGTATAGTGGTG
t0005234	21	139	GCGGATATGGTCGAATGGTAA
t0005245	27	139	GGGCCTGTAGCTCAGAGGATTAGAGCA
t0005249	20	139	GTCAAGATGGCCGAGTTGGT
t0005259	20	139	GGCGATGTAGCTCAGATGGT
t0005291	25	138	GGGGCTGTAGCTCAGCTGGGAGAGC
t0005315	24	138	GGTGATGTAGCTCAGATGGTAGAG
t0005318	28	137	GCGGCTGTAGCTCAGTTGGATAGAGTAC
t0005335	29	137	GTCGTTGTAGTATAGTGGTGAGTATTCCC
t0005346	28	137	GGGATTGTAGTTCAATCGGTCAGAGCAC
t0005382	22	136	GGGGATGTAGCTCAGATGGTAA
t0005395	23	136	GGGGATGTAGCTCAGATGGTATA
t0005400	24	136	GGGGATGTAGCTCAAACGGTAGAA
t0005407	19	135	GCACCAGTGGTCTAGTGGT
t0005439	27	135	GGGGATGTAGCTCAGATGGTAGAGCTC
t0005455	25	134	GCGGGTGTAGCTCAGGGGTAGAGCA
t0005515	23	133	GGGATGTAGCTCAGATGGTAGAG
t0005553	20	132	GGCGGATGTGGCCAAGTGGA
t0005583	25	131	GGAGAGATGGCTGAGTGGACTAAAG
t0005613	27	130	GTGGTAGAGCATTTGACTGCAGATCAA
t0005641	20	130	GGCGGATGTAGCCAAGTGGA
t0005717	24	128	GTCGTTGTAGTATAGTGGTAAGTA
t0005748	29	127	GCGTTTGTAGTCCAACGGTTAGGATAATT
t0005750	27	127	GGGGATGTGGCGGAATGGCAGACGCGC
t0005772	19	127	GTTGAGATGGCCGAGTTGG
t0005851	23	125	GCGCCTGTAGCTCAGTGGATAGA
t0005867	20	125	GGGGATGTAGCTCAGATGGG
t0005937	25	123	GCCCAGGTGGCGGAATTGGTAGACG
t0005978	27	123	GCCCGGATGGTGGAATGCAGACACGGC
t0006013	19	122	GGGGGTGTAGCTCATATGG
t0006048	20	121	AGGGATATAACTCAGCGGTA
t0006062	20	121	GCGGATATGGTCGAATGGTA
t0006090	21	120	GGCGATGTAGCTCAGATGGTA
t0006105	28	120	GCGTTTGTAGTCCAACGGTTAGGATAAT
t0006108	26	120	GGGGATGTAGCTCAGATGGTAGATTT
t0006116	28	120	GGGGCTGTAGCTCAGTTGGGCGAGCGCT
t0006136	19	120	GGGGATGTAGCTCAAATGG
t0006154	29	119	GGGGCTGTAGCTCAGCTGGGCGAGCACCT
t0006189	25	119	GGGGATGTAGCTCAGATGGTAGATT
t0006206	28	118	GGGGATGTAGCTCAGATGGTAGAGCTCG
t0006212	26	118	GTCGTTGTAGTATAGTGGTAAGTATT
t0006237	28	118	CGGGGTGTAGCTCAGCCTGGTAGAGCGC
t0006286	28	117	GCGGGTGTAGTTTAATGGTAGAACATCA
t0006306	28	117	GGGCGGTTAGCTCAGCGGTAGAGCACTG
t0006376	21	115	GTCGATATGTCCGAGTGGTTA
t0006393	29	115	GTCGTTGTAGTATAGTGGTAAGTATTCCC

t0006395	29	115	AGCGGGGTAGAGGAATTGGTCAACTCATC
t0006398	29	115	GCGGACGTAGCTCAGTTGGTCGAGCGCAA
t0006425	28	115	GGCGTTTGGTCTAGTGGTATGATTCTCG
t0006441	22	114	GTCTGGGTCGTGTAGTTGGTTA
t0006466	21	114	CTAGCGGTTAGGACATTGGAC
t0006502	21	114	GTGGTAGAGCATTTGACTGCA
t0006538	27	113	GCGGAAATAGCTTAATGGTAGAGCATA
t0006574	27	113	GGGGCTGTAGCTCAGCTGGGAGAGCAC
t0006622	22	112	GCGGGGTAGAGGAATTGGTCAA
t0006631	19	112	GTGTCGTGGTGTAGTTGGT
t0006656	22	111	CCGACCTTAGCTCAGTTGGCAG
t0006699	21	111	GGGGATGTAGCTCAGAGGTAG
t0006714	20	110	GTTGGTTAGGATACTCGGCT
t0006788	22	109	GGGAATGTAGCTCAGATGGTAG
t0006798	20	109	GGGTCTGTAGCTCAGTCGGT
t0006847	23	108	AGCGGGGGAGAGGAATTGGTCAA
t0006852	22	108	AGTGGTAGAGCATTTGACTGCA
t0006881	21	108	GGCGGATGTAGCCAAGTGGAT
t0006891	29	108	GCGAGGATGGCGGAATTGGTAGACGCGCT
t0006900	19	108	GGAGAGATGGCTGAGTGGA
t0006906	20	107	GTGTCGTGGTGTAGTTGGTT
t0006936	23	107	GCGGACGTAGCGCAGCTGGTAGC
t0006938	20	107	GGAGAGATGGCTGAGTGGAC
t0007014	27	106	GGGGATGTAGCTCAGATGGTAGAGCGC
t0007026	23	106	GTCGTTGTAGTATAGTGGTAAGT
t0007101	28	105	GGGGCTGTAGCTCAGCTGGGAGAGCGCC
t0007109	26	105	GGTGTCGTCGTGTAGTTGGTTATCAC
t0007142	21	104	GTCGTTGTAGTATAGTGGTGA
t0007155	21	104	GGGGATGTAGCTCAGATGGGA
t0007169	30	104	GGGGATGTAGCTCAAACGGTAGAGCGCTCG
t0007191	22	103	GTGTCGTGGTGTAGTTGGTTAT
t0007216	24	103	GCGGGTGTAGCTCAGGGGTAGAGC
t0007263	27	102	GGCTCAGTGGTCTAGTGGTATGATTCT
t0007278	27	102	GTCGCTTTGGCCGAGTGGTTAAGGCTT
t0007286	22	102	GTCGTTGTAGTATAGTGGTAAG
t0007298	23	102	GTCTGGGTTGTGTAGTTGGTTAT
t0007341	22	101	GGTGTCGTGGTGTAGTTGGTTC
t0007342	25	101	GGAGATGTAGCTCAGATGGTAGAGC
t0007359	26	101	GAGTGGCGCAGCGGAAGCGTGGTGGG
t0007397	23	101	TGGGATGTAGCTCAAACGGTAGA
t0007449	27	100	GGGGATGTAGCTCAAACGGTAGAGCGC
t0007486	23	99	GCGTTTGTAGTCCAACGGTTAGG
t0007489	23	99	GGGAATGTAGCTCAGATGGTAGA
t0007497	20	99	AGTCCCGTAGCTCAGTTGGT
t0007504	22	99	GGGGATGTAGCTCAGATGGTTT
t0007519	26	99	GCGGGTGTAGTTTAATGGTAGAACAT
t0007555	24	99	GTCTGGGTGGTGTAGTCGGTTATC
t0007573	29	98	GGCGTTTGGTCTAGTGGTATGATTCTCGC
t0007578	22	98	TCCGTTGTAGTCTAGGTGGTTA
t0007614	28	98	GGTAGAGCTGAGGACTGTAGATCCTTAG

t0007623	27	98	CGGGGTGTAGCTCAGCCTGGTAGAGCG
t0007645	31	97	GGGGATGTAGCTCAGATGGTAGAGCGCTCGC
t0007682	28	97	GCCCAGATGGCGGAATTGGTAGACGCGC
t0007688	25	97	GCGGGGATAGCTCAGTTGGGAGAGC
t0007734	23	96	GGGGATGTAGCTCAGATGGTAGT
t0007756	28	96	GTCCGGGTGGCGGAATGGCAGACGCGCT
t0007791	22	96	AGCGGTGTAGCTCAGTCGGTAG
t0007815	20	96	CTAGCGGTTAGGACATTGGA
t0007850	20	95	GACCGCATAGCGCAGTGGAT
t0007862	22	95	GGGGATGTAGCTCAGAGGTAGA
t0007888	25	95	GTCTGGGTGGTGTAGTCGGTTATCA
t0007911	21	94	GGGGATATGGCGAAATTGGTA
t0007923	25	94	GCGCCTGTAGCTCAGTGGATAGAGC
t0007935	25	94	GGCGGATGTAGCCAAGTGGATCAAG
t0007990	20	94	TGGTAGAGCATTTGACTGCA
t0008017	27	93	CCGGTATGGTGTAGTGGCTAACATTTC
t0008036	26	93	GGGGATGTAGCTCAGATGGTAGAGCC
t0008059	29	93	GGGGCTGTAGCTCAGCTGGGAGAGCGCCG
t0008064	19	93	TTGGTTAGGATACTCGGCT
t0008110	24	92	GGGGATGTAGCTCAAACGGTAGAT
t0008148	23	92	GGCGGATGTAGCCAAGTGGATCA
t0008156	30	92	GGGGCTGTAGCTCAGTTGGGAGAGCGCCTG
t0008190	28	91	AGTGGTAGAGCATTTGACTGCAGATCAA
t0008192	21	91	GACCGCATAGCGCAGTGGATT
t0008208	23	91	GTGTCGTGGTGTAGTTGGTTATC
t0008244	25	91	GGTGTCGTAGTGTAGTTGGTTATCA
t0008262	24	90	GCCCAGGTGGCGGAATTGGTAGAC
t0008279	23	90	TCCGTTGTAGTCTAGTTGGTTAG
t0008304	22	90	GGGATGTAGCTCAAACGGTAGA
t0008326	24	90	GCGTTTGTAGTCCAACGGTTAGGA
t0008332	20	90	GTGGATGTAGCTCAGATGGT
t0008367	26	89	GCGGGTGTAGCTCAGTCGGTTAGAGT
t0008385	21	89	GTGGATGTAGCTCAGATGGTA
t0008429	24	88	GGTGTCGTAGTGTAGTTGGTTATC
t0008440	29	88	CGGGGTGTAGCTCAGCCTGGTAGAGCGCT
t0008507	26	88	GGGGATGTAGCTCAGATGGTAGAGCG
t0008508	22	88	GGCGATGTAGCTCAGATGGTAG
t0008534	25	87	GGCTTTGGTCTAGCGGTATGATTCT
t0008544	29	87	GTCCGGGTGGCGGAATGGCAGACGCGCTA
t0008561	28	87	GGGCCTGTAGCTCAGAGGATTAGAGCAC
t0008562	29	87	GCACTCGTAGCTTAACGGATAGAGCATCT
t0008764	20	85	GGGGATGTAGCTCAGATGGA
t0008828	28	85	GTGGTAGAGCATTTGACTGCAGATCAAG
t0008906	26	84	TCCATTGTCGTCTAGTCCGGTTAGGA
t0008923	25	84	GCGTTTGTAGTCCAACGGTTAGGAT
t0008934	23	84	GGTGTCGTAGTGTAGTTGGTTAT
t0008976	23	83	CCGACCTTAGCTCAGTTGGCAGA
t0008983	26	83	GGAGAGATGGCTGAGTGGACTAAAGC
t0008988	29	83	GGCTTTGTGGTCTAGCGGTATGATTCTCG
t0009026	27	83	AGCGGTGTAGCTCAGTCGGTAGAGCAA

t0009139	23	82	GCACCAGTGGTCTAGTGGTAGAA
t0009186	21	81	CGGGATGTAGCTCAGATGGTA
t0009235	24	81	GGCGGATGTAGCCAAGTGGATCAA
t0009265	22	81	GTCAGGATGGCCGAGTGGTCTA
t0009302	29	80	GCCCAGATGGCGGAATTGGTAGACGCGCA
t0009346	27	80	GGGGCTGTAGCTCAGCTGGGAGAGCGC
t0009427	22	79	GCGTTTGTAGTCCAACGGTTAG
t0009524	29	78	GGGCGTTTGGTCTAGTGGTATGATTCTCG
t0009634	22	78	GGTGATGTAGCTCAAACGGTAG
t0009643	19	77	GGTGATGTAGCTCAGATGG
t0009660	26	77	GTCGTTGTAGTATAGTGGTGAGTATT
t0009675	24	77	GTGGACGTGCCGGAGTGGTTATCG
t0009687	23	77	GTCGTTGTAGTATAGTGGTGAGT
t0009688	24	77	GTCGTTGTAGTATAGTGGTGAGTA
t0009690	28	77	GGAGAGATGGCTGAGTGGACTAAAGCGG
t0009704	26	77	GGGCGTTTGGTCTAGTGGTATGATTC
t0009748	27	77	GCGCTCGTGGCGGAATTGGTAGACGCG
t0009794	20	76	GCACTCGTAGCTTAACGGAT
t0009825	23	76	AGTGGTAGAGCATTTGACTGCAG
t0009832	24	76	AGATTGAGGTTCTGGTCCGAAAGG
t0009916	24	75	GGGGATGTAGCTCAGATGGTAGTT
t0009946	27	75	GGTGGTTGTAGCTCAGTTGGTAGAGTC
t0009951	28	75	GGATGGATGTCTGAGCGGTTGAAAGAGT
t0010001	23	75	GGGGATGTAGCTCAGAGGTAGAG
t0010009	27	75	AGTGGTAGAGCATTTGACTGCAGATCA
t0010017	27	75	GCGGGGTAGAGGAATTGGTCAACTCAT
t0010064	25	74	CCGGGTGGCGGAATGGCAGACGCGC
t0010072	19	74	GCGTTTGTAGTCCAACGGG
t0010078	24	74	GGGAATGTAGCTCAGATGGTAGAG
t0010091	18	74	GCGGGGTAGAGGAATTGG
t0010120	21	74	TGGGGTGTAGCTCATATGGTA
t0010123	26	74	GGGGATGTAGCTCAAACGGTAGAGCC
t0010204	27	73	GGTGGCTGTAGCTCAGTTGGTAGAGTC
t0010246	23	73	CTAGTTGGTTAGGATACTCGGCT
t0010252	27	73	GCGGGTGTGGCGGAACTGGTAGACGCA
t0010298	21	72	GCGGGTGTAGTTTAATGGTAG
t0010299	27	72	GGCGGATGTAGCCAAGTGGATCAAGGC
t0010301	20	72	CGGGATGTAGCTCAGATGGT
t0010329	30	72	GGGGCTATAGCTCAGTTGGTTAGAGCGCAT
t0010360	27	72	GTAGAGCTGAGGACTGTAGATCCTTAG
t0010386	21	72	GTGGCTGTAGTTTAGTGGTTA
t0010393	18	72	GGGATGTAGCTCAGATGG
t0010407	24	72	ACTTCTAATCAGGCGATTGTGGGT
t0010420	27	72	GGTGTCGTGGTGTAGTTGGTTATCACT
t0010428	22	72	TTCGAATCCTGCTGTCGACGCC
t0010478	23	71	GGAGTTGTAGCTCAAATGGTAGA
t0010480	19	71	TGGTTAGGATACTCGGCTC
t0010538	23	71	GGTAGAGCTGAGGACTGTAGATC
t0010616	20	70	GGTTCTATGGTGTAGTGGTT
t0010629	28	70	GCCCAGGTGGCGGAACTGGTAGACGCGC

t0010633	20	70	TGGGGCGTGGCCAAGCGGTA
t0010678	24	70	GCGGACGTAGCTCAGTTGGTAGAG
t0010726	24	69	TCCGGAGCGGGAGATTGTGGGTTC
t0010794	24	69	TGGGTTGTAGCTCAAATGGTAGAG
t0010798	24	69	TGGGATGTAGCTCAAACGGTAGAG
t0010806	25	69	GGGGATGTAGCTCAAATGGTCGAGC
t0010877	25	68	TGGGATGTAGCTCAGATGGTAGAGC
t0010918	20	68	AGGGATGTAGCGCAGCTTGG
t0010927	28	68	TCCATTGTCGTCTAGTCCGGTTAGGATA
t0010946	22	68	GTCTGGGTAGTGTAGTTGGTTA
t0010985	25	68	TGGTAGAGCATTTGACTGCAGATCA
t0011034	25	67	GGTGGCTGTAGTTTAGTGGTCAGAA
t0011040	30	67	GCGGCTGTAGCTCAGTTGGATAGAGTACTT
t0011051	21	67	CCGACCTTAGCTCAGTTGGCA
t0011057	23	67	GGTGTCGTGGTGTAGTTGGTTCT
t0011065	19	67	GGGTCTGTAGCTCAGTCGG
t0011131	21	67	GTCAGGATGGCCGAGTGGTCT
t0011135	25	67	GCGGACGTAGCTCAGTTGGTAGAGC
t0011158	31	67	GGGGCTGTAGCTCAGATGGGAGAGCGCTGCA
t0011229	24	66	GTGTCGTGGTGTAGTTGGTTATCA
t0011345	29	66	GCGGGTGGCGGAATAGGCAGACGCGCTGG
t0011359	25	66	GGGGATGTAGCTCAGATGGTAAAAA
t0011438	31	65	GGGATTGTAGTTCAATCGGTCAGAGCACCGC
t0011461	28	65	GCGGATATGGCGAAATTGGTAGACGTGC
t0011513	29	65	GAGGGCGTGGGTTCATCCCACTTCTGACA
t0011547	22	64	GGAGTTGTAGCTCAAATGGTAG
t0011571	24	64	TGGTAGAGCATTTGACTGCAGATC
t0011595	27	64	GCGGGTGTAGTTCAATGGCAGAACATC
t0011618	26	64	GGTGTCGTTGTGTAGTTGGTTATCAC
t0011625	26	64	GTGGTAGAGCATTTGACTGCAGATCA
t0011656	23	64	GGCGGATGTGGCCAAGTGGATCA
t0011792	24	63	AGGGATGTAGCGCAGCTTGGTAGC
t0011912	24	63	GGGTCTGTAGCTCAGTCGGTTAGA
t0011944	26	62	GGCGGATGTGGCCAAGTGGATCAAGG
t0011978	27	62	GGTCTGGTGGTCTAGTGGTATGATTCT
t0012004	26	62	GGTTCTATGGTCTAGCGGTTAGGACA
t0012022	25	62	GTCGATATGTCCGAGTGGTTAAGGA
t0012063	25	62	GGCGACCCGGGTTCATATCCCGGCA
t0012072	26	62	GGGCCTGTAGCTCAGAGGATTAGAGC
t0012096	29	62	GGGGATGTAGCTCAGATGGTAGAGCGCTC
t0012101	27	62	ACGGACTGTAAATTCGTTGACGATATG
t0012123	24	61	GTCGATATGTCCGAGTGGTTAAGG
t0012158	28	61	GGCGGATGTAGCCAAGTGGATCAAGGCA
t0012162	20	61	AGCAGAAGGCCGTAGGTTCG
t0012175	27	61	GCGGGTGTAGCTCAGTTGGTAGAGCGC
t0012282	31	61	GCACCAGTTGTCTAGTGGTAGAATAGTACCC
t0012339	26	60	CCGGATGGTGGAATGCAGACACGGCG
t0012342	24	60	GGAGTTGTAGCTCAAATGGTAGAG
t0012356	27	60	GGGGCTGTAGCTCAGTTGGGAGAGCGC
t0012368	28	60	GTCGTTGTAGTATAGTGGTGAGTATTCC

t0012385	24	60	GCACCAGTGGTCTAGTGGTAGAAT
t0012398	29	60	GGGGCTGTAGCTCAGTTGGGCGAGCGCTA
t0012453	18	60	GTCGTTGTAGTATAGTGG
t0012460	28	60	GCGGATATGGTCGAATGGTAAAATTTCT
t0012471	20	60	GGGGATATGGCGAAATTGGT
t0012475	23	60	TGGGTTGTAGCTCAAATGGTAGA
t0012481	21	60	GGAGGTATGGCTGAGTGGCTT
t0012482	26	60	GCGTTTGTAGTCCAACGGTAAGGATA
t0012488	19	59	GTCAGGATGGCCGAGTGGT
t0012508	22	59	GTGGTAGAGCATTTGACTGCAG
t0012522	23	59	GGTGGCTGTATTTTAGTGGTTAG
t0012593	25	59	GCGGATATGGCGAAATTGGTAGACG
t0012596	28	59	GGGGATGTAGCTCAAACGGTAGAGCGCT
t0012609	21	59	GTTGAGATGGCCGAGTTGGTC
t0012633	28	59	GGGCATTTGGTCTAGTGGTATGATTCTC
t0012643	21	59	GCCCGTCTAGCTCAGTTGGTA
t0012648	20	59	GGTGGCTGTAGTTTAGTGGA
t0012678	27	59	GCGGGCGGCCCGGGTTCGTTTCCCGGC
t0012681	29	58	GGGGATGTAGCTCAGATGGTAGAGCCTCG
t0012726	27	58	GCCCAGATGGCGGAATTGGTAGACGCG
t0012780	24	58	GGTAGAGCATTTGACTGCAGATCA
t0012799	26	58	GGATGGATGTCTGAGCGGTTGAAAGA
t0012809	22	58	GGCGGATGTAGCCAAGTGGATC
t0012831	28	58	GCGGGAGTAGCTCAGTTGGTAGAGCGCA
t0012898	21	57	CAAGCAAGGACTCTACCACGC
t0012931	26	57	GCGGGTGTAGTTCAATGGTAGAACGG
t0012943	22	57	TGTGGCTGTAGTTTAGTGGTTA
t0012966	26	57	GCGGGTGTAGTTCAATGGCAGAACGG
t0013032	25	57	GGGGATGTAGCTCAGATGGTAGATC
t0013033	28	57	GCCCAGGTGGCGGAATTGGTAGACGCGC
t0013061	22	57	GTTGTAGTATAGTGGTGAGTAT
t0013154	28	56	GGTCTGGTTGTCTAGCGGTATGATTCTC
t0013249	23	56	CTCAGTTGGTAGAGCTGAGGACT
t0013289	20	56	CTCTCGGTAGCTCAGTTGGT
t0013305	29	56	GCGGGAGTAGCTCAGTTGGTAGAGCGCAA
t0013320	23	56	CAGTGGTAGAGCATTTGACTGCA
t0013329	29	56	GCGTGAATGGTTTAGTGGTAAAATTCTCC
t0013361	26	56	GCCCAGATGGCGGAATTGGTAGACGC
t0013416	22	55	GGTAGAGCTGAGGACTGTAGAT
t0013455	22	55	TAAGCAGAAGGCCGTAGGTTCG
t0013471	29	55	TGGTAGAGCTGAGGACTGTAGATCCTTAG
t0013506	25	55	GGGCGTGTAGCTCAGCGGGAGAGCA
t0013507	26	55	GCGCCCTTCGTCTATCGGTTAGGACA
t0013514	27	55	GCGCGGATGGCGGAATTGGTAGACGCG
t0013517	30	55	GGGGATGTAGCTCAGATGGTAGAGCTCTCG
t0013542	22	55	GTGGATGTAGCTCAGATGGTAG
t0013553	23	55	GGGATGTAGCTCAAACGGTAGAG
t0013560	26	55	GGGGATGTAGCTCAGATGGTAGAGCA
t0013576	27	55	GGGCATTTGGTCTAGTGGTATGATTCT
t0013606	30	54	GCACCAGTTGTCTAGTGGTAGAATAGTACC

t0013650	23	54	TGGGGTGTAGCTCATATGGTAGA
t0013668	27	54	GCCCAGGTGGCGGAATTGGTCGACGCA
t0013680	23	54	GTCGATATGTCCGAGTGGTTAAG
t0013687	25	54	GGTAGAGCATTTGACTGCAGATCAA
t0013697	29	54	GCGGTCGTGGCGGAATTGGTAGACGCGCA
t0013705	24	54	AGCGGTGTAGCTCAGTCGGTAGAG
t0013715	31	54	GAGGGCGTGGGTTCATATCCCACTTCTGACA
t0013717	22	54	GTCGATATGTCCGAGTGGTTAA
t0013737	25	54	ACTTCTAATCAGGCGATTGTGGGTT
t0013765	23	54	GGGGATGTAGCTCAGATGGTAAA
t0013787	24	54	GTCTGGGTTGTGTAGTTGGTTATC
t0013796	21	54	AGCGGTGTAGCTCAGTCGGTA
t0013822	20	54	GGGGATGTAGCTCAGAGGTA
t0013849	22	53	GTCGTTGTAGTATAGTGGTGAG
t0013924	23	53	GGAGATGTAGCTCAAATGGTAGA
t0013995	26	53	GCGGACGTAGCTCAGTTGGTAGAGCG
t0014022	24	53	GCGGGGTAGAGGAATTGGTCAACT
t0014044	23	53	TCCGTTGTAGTCTAGGTGGTTAG
t0014051	24	53	TGGGGTGTAGCTCATATGGTAGAG
t0014069	27	53	TTGGTAGAGCTGAGGACTGTAGATCCT
t0014080	23	53	GCGGGTGTAGTTTAATGGTAGAA
t0014092	23	53	GGTGATGTAGCTCAAACGGTAGA
t0014096	24	53	TCCGTTGTAGTCTAGGTGGTTAGG
t0014170	30	52	GGGTCTGTAGCTCAGTCGGTTAGAGCACCG
t0014183	21	52	CCGACCTTAGCTCAGTTGGTA
t0014214	28	52	GCGGATGTGGCGGAACTGGTAGACGCGC
t0014250	27	52	GCGGGTGTAGTTCAGTAGTAGAACATC
t0014351	22	52	GCGGATATGGTCGAATGGTAAA
t0014372	29	52	TTGGTAGAGCTGAGGACTGTAGATCCTTA
t0014413	23	51	GTCAAGATGGCCGAGTTGGTCTA
t0014417	30	51	GGGGATGTAGCTCAGATGGTAGAGCCTCGC
t0014429	27	51	GACTTCTAATCAGGCGATTGTGGGTTC
t0014497	24	51	GGGGATGTAGCTCAAATGGTAGAA
t0014501	24	51	GGAGATGTAGCTCAAATGGTAGAG
t0014511	28	51	GCGGGTGTAGCTCAGTGGTAGAGCACGA
t0014522	29	51	GCGGATATGGTCGAATGGTAAAATTTCTC
t0014528	21	51	TGGTAGAGCATTTGACTGCAG
t0014538	29	51	GCGGGTGTAGCTCAGTTGGTAGAGCGCAA
t0014547	22	51	GCACCAGTGGTCTAGTGGTAGA
t0014561	28	51	CGGGATGTAGCTCAGCCTGGTAGAGCGT
t0014563	32	51	GCACCAGTGGTCTAGTGGTAGAATAGTACCCT
t0014586	25	51	GTCTGGGTTGTGTAGTTGGTTATCA
t0014617	22	51	TGGGGTGTAGCTCATATGGTAG
t0014675	27	50	GGCGTTTGGTCTAGTGGTATGATTCTC
t0014685	20	50	GTGGCTGTAGTTTAGTGGTA
t0014706	24	50	TCAGTGGTAGAGCATTTGACTGCA
t0014725	27	50	GCGGGTGTAGTTTAATGGTAGAACATC
t0014732	27	50	GCGGCTGTAGCTCAGTTGGATAGAGTA
t0014755	28	50	GGGGATGTAGCTCAGATGGTAGAGCGCT
t0014783	24	50	GGGGATGTAGCTCAAACGGTAGTT

t0014792	27	50	AGAGCTGAGGACTGTAGATCCTTAGGT
t0014954	24	49	AGTGGTAGAGCATTTGACTGCAGA
t0014979	28	49	GCGGGTGGCGGAATAGGCAGACGCGCTG
t0014998	29	49	GCTCGGATGGTGAAATTGGTAGACACGCT
t0015052	23	49	GGGGATGTAGCTCAAACGGTAGT
t0015087	27	49	GCCCAGGTGGCGGAATTGGTAGACGCG
t0015097	24	49	GGGCCTGTAGCTCAGAGGATTAGA
t0015107	24	49	CCGGATGGTGGAATGCAGACACGG
t0015119	22	49	CGGGATGTAGCTCAGATGGTAG
t0015131	18	49	GTGGCTGTAGTTTAGTGG
t0015286	27	48	GCGTTTGTAGTCCAACGGTAAGGATAA
t0015304	19	48	CTAGCGGTTAGGACATTGG
t0015313	22	48	GGGGGTGTAGCTCATATGGAAG
t0015323	26	48	GGGTCCATAGCTCAGTGGTAGAGCAT
t0015351	22	48	GCGGGTGTAGTTTAATGGTAGA
t0015358	23	48	CCGACCTTAGCTCAGTTGGTAGA
t0015393	28	48	GCGGACGTAGCGCAGCTGGTAGCGCATC
t0015432	24	48	TCCGTTGTAGTCTAGTTGGTTAGG
t0015433	21	48	TCCGGAGCGGGAGATTGTGGG
t0015450	27	48	AGGACATTGGACTCTGAATCCAGTAAC
t0015476	25	48	GGGCCTGTAGCTCAGAGGATTAGAG
t0015529	27	48	GGCGGTGTAGCTCAGATGGCAGAGCAA
t0015652	22	47	AGGGATGTAGCGCAGCTTGGTA
t0015668	31	47	GGGGCTGTAGCTCAGCTGGGCGAGCACCTGC
t0015682	18	47	GGTGTCGTGGTGTAGTTG
t0015735	19	47	GGGGATGTAGCTCAGAGGT
t0015777	22	47	CCGACCTTAGCTCAGTTGGTAG
t0015796	21	47	GCACTCGTAGCTTAACGGATA
t0015876	28	47	GGGGCTGTAGCTCAGCTGGGAGAGCGCG
t0015926	26	46	GGTGGCTGTAGCTCAGTTGGTAGAGT
t0015949	25	46	GGCGGATGTGGCCAAGTGGATCAAG
t0015976	26	46	GGGGATGTAGCTCAAACGGTAGAGCG
t0016075	25	46	GGGTCCATAGCTCAGTGGTAGAGCA
t0016100	28	46	GGCATTTGGTCTAGTGGTATGATTCTCG
t0016157	22	46	GGCGGATGTGGCCAAGTGGATC
t0016164	23	46	GGAGGTATGGCTGAGTGGCTTAA
t0016184	26	46	GGGCATTTGGTCTAGTGGTATGATTC
t0016186	23	46	GTGGTAGAGCATTTGACTGCAGA
t0016190	22	46	CAGTTGGTAGAGCTGAGGACTG
t0016278	26	45	ACTTCTAATCAGGCGATTGTGGGTTC
t0016284	28	45	GGGGCTGTAGCTCAGCTGGGAGAGCGCA
t0016294	21	45	TGGGATGTAGCTCAGATGGTC
t0016295	30	45	GCCCGGATGGTGGAATGCAGACACGGCGAG
t0016302	23	45	GTGGATGTAGCTCAGATGGTAGA
t0016325	24	45	GCGGGTGTAGCTCAATGGTAGAGC
t0016350	23	45	TGGGATGTAGCTCAAATGGTAGA
t0016444	23	45	TCCGGAGCGGGAGATTGTGGGTT
t0016526	27	45	GCGGATATGGCGAAATTGGTAGACGTG
t0016567	27	45	CGGGATGTAGCTCAGCCTGGTAGAGCG
t0016610	24	44	GTTCGTGCCCCACGGTGGGCGCCA

t0016614	24	44	GGGGTTGTAGCTCAAATGGTAGAA
t0016630	22	44	CTCGAGAGAGGGCGTGGGTTCA
t0016639	29	44	TGGTAGAGCATTTGACTGCAGATCAAGAG
t0016649	25	44	TGGGATGTAGCTCAAACGGTAGAGC
t0016817	25	44	CCAGTGGTCTAGTGGTAGAATAGTA
t0016837	21	44	AGCAGAAGGCCGTAGGTTCGT
t0016867	29	44	GGTCTGTAGTTCGTTCCTGCATGGGGGCA
t0016930	24	44	GTGGATGTAGCTCAGATGGTAGAG
t0016943	23	44	GCGCATCTGGTGTAGTGGTATCA
t0016992	26	43	TCCGGTATGGTGTAGTGGCTAACATT
t0017121	24	43	GGTGGCTGTAATTTAGTGGTTAGA
t0017127	22	43	TCCGGAGCGGGAGATTGTGGGT
t0017148	28	43	GTGGGTTCGTGCCCCACGGTGGGCGCCA
t0017160	24	43	GGGGATGTAGCTCAGATGGTAAAA
t0017197	27	43	TGGTAGAGCTGAGGACTGTAGATCCTT
t0017229	28	43	GCGGGTGTAGTTCAATGGCAGAACGGCA
t0017247	32	43	GGGGCTGTAGCTCAGCTGGGAGAGCACCTGCT
t0017307	25	43	GGCGACCCGGGTTCAGATCCCGGCA
t0017337	27	42	GGATGGATGTCTGAGCGGTTGAAAGAG
t0017395	18	42	TGGTTAGGATACTCGGCT
t0017399	22	42	GTCAAGATGGCCGAGTTGGTCT
t0017451	24	42	GCGCGGATGGCGGAATTGGTAGAC
t0017458	28	42	GTCGTTGTAGTATAGTGGTAAGTATTCC
t0017505	19	42	TCCATTGTCGTCTAGTCCG
t0017515	21	42	GCGTTTGTAGTCCAACGGTAC
t0017522	25	42	CTAGTTGGTTAGGATACTCGGCTCT
t0017578	26	42	GCGGGTGTAGCTCAATGGTAGAGCAG
t0017618	21	42	GGGGATGTAGCTCATATGGTA
t0017646	27	42	GCGGACGTAGCTCAGTTGGTCGAGCGC
t0017647	28	42	GTAGAGCTGAGGACTGTAGATCCTTAGG
t0017737	23	41	CTTCTAATCAGGCGATTGTGGGT
t0017845	28	41	GGGGCTATAGCTCAGCTGGGAGAGCGCT
t0017854	19	41	GGTTCTATGGTGTAGTGGT
t0017911	22	41	GGAGATGTAGCTCAAATGGTAG
t0017943	31	41	GCACCAGTAGTCTAGTGGTAGAATAGTACCC
t0017956	27	41	GGGTCCATAGCTCAGTGGTAGAGCATT
t0017972	26	41	GGTAGAGCATTTGACTGCAGATCAAG
t0018000	20	41	GGTCCCGTAGCTCAGTTGGT
t0018002	22	41	GGTGTCGTGGTGTAGTTGGTTT
t0018023	22	41	GTTGTAGTATAGTGGTAAGTAT
t0018097	23	41	GCGCCTGTAGCTCAGTGGACAGA
t0018170	25	40	GCACCAGTGGTCTAGTGGTAGAATA
t0018182	24	40	GGCGGATGTGGCCAAGTGGATCAA
t0018242	24	40	GTGGTAGAGCATTTGACTGCAGAT
t0018246	29	40	GGGCCTGTAGCTCAGAGGATAGAGCACGT
t0018268	21	40	TGTGGCTGTAGTTTAGTGGTA
t0018311	23	40	CGGGATGTAGCTCAGATGGTAGA
t0018325	18	40	GGGGATGTAGCTCAGATG
t0018365	24	40	GCGGGTGTAGTTCAATGGCAGAAC
t0018378	22	40	GGGGTGTAGCTCATATGGTAGA

t0018397	21	40	GGAGATGTAGCTCAAATGGTA
t0018416	28	40	GGTAGAGCATTTGACTGCAGATCAAGAG
t0018456	29	40	GGTAGAGCTGAGGACTGTAGATCCTTAGG
t0018503	28	40	GGCTCAGTGGTCTAGTGGTATGATTCTC
t0018510	22	40	GGGGATGTAGCTCATATGGTAG
t0018512	31	40	CCCCTGTAGCTCAATTGGCAGAGCAGCCGGC
t0018528	28	40	CGGGGTGTAGCTCAGCCTGGTAGAGCAC
t0018569	22	40	TGGTAGAGCTGAGGACTGTAGA
t0018592	25	39	GCGGGTGTAGTTCAATGGCAGAACA
t0018609	27	39	GGGGCGGTAGCTCAGCCGGTTAGAGCA
t0018658	28	39	GCGGGTGTAGCTCAGTTGGTAGAGCGCG
t0018704	22	39	CGTGTCGTGGTGTAGTTGGTTA
t0018712	26	39	GGCGTTTGGTCTAGTGGTATGATTCT
t0018741	22	39	GGGGATATGGCGAAATTGGTAG
t0018756	26	39	GGTGGTTGTAGCTCAGTTGGTAGAGT
t0018792	21	39	GCGCGGATGGCGGAATTGGTA
t0018843	20	39	TGGTTAGGATACTCGGCTCT
t0018899	29	39	GGGGCTGTAGCTCAGTTGGGCGAGCGCCT
t0018908	24	39	GCGGAAGTAGCTCAGTTGGTAGAG
t0018954	29	39	GGGGCTATAGCTCAGTTGGTTAGAGCGCA
t0018959	22	39	TCCGGTATGGTGTAGTGGCTAA
t0018988	23	39	GGGCCTGTAGCTCAGAGGATTAG
t0019024	26	38	GTAGAGCGGAGGACTGTAGATCCTTA
t0019029	22	38	TAGTTGGTTAGGATACTCGGCT
t0019052	28	38	GCAATAGACCCGGGTTCGGCTCCCGGCA
t0019183	26	38	GAGTTGCGCAGCGGAAGCGTGGTGGG
t0019217	23	38	GTCTGGGTCGTGTAGTTGGTTAT
t0019246	23	38	ACTTCTAATCAGGCGATTGTGGG
t0019295	22	38	TCAGTTGGTAGAGCTGAGGACT
t0019298	27	38	CCGGGTGGCGGAATGGCAGACGCGCTA
t0019302	23	38	CTAAGCAGAAGGCCGTAGGTTCG
t0019392	23	38	GCGGGTGTAGTTCAATGGCAGAA
t0019449	28	38	GCCCGGATGGCGGAATTGGTAGACGCGC
t0019516	30	37	GGGGCTGTGGCGCAGACTGGTAGCGCACCT
t0019602	19	37	GGCGATGTAGCTCAGATGG
t0019700	22	37	AGCGGGGGAGAGGAATTGGTCA
t0019705	23	37	GGGGATGTAGCTCATATGGTAGA
t0019716	28	37	GCGGGTGTAGTTCAATGGCAGAACATCA
t0019721	28	37	GCGGGTGTAGTTCAATGGTAGAACGCTA
t0019724	21	37	AGGGATGTAGCTCAGATGGTA
t0019818	31	37	GGGGATGTAGCTCAGATGGTAGAGCTCTCGC
t0019831	20	37	GCAGAAGGCCGTAGGTTCGT
t0019914	24	37	CAGTGGTAGAGCATTTGACTGCAG
t0019915	24	37	GCGCGGGTGGCGGAATAGGCAGAC
t0019950	29	37	CGGGGTGTAGCTCAGCCTGGTAGAGCACT
t0019953	24	37	GGTGATGTAGCTCAAACGGTAGAG
t0019956	22	37	GGGGATGTAGCTCAGACGGTAG
t0019980	23	37	AGCGGGGTATAGGAATTGGTCAA
t0019989	25	37	AGTGGTAGAGCATTTGACTGCAGAT
t0020001	24	37	TGGGATGTAGCTCAAATGGTAGAG

t0020010	19	37	GCGAGAGGTACGGGGATCG
t0020047	24	37	TGTGGCTGTAGTTTAGTGGTTAGA
t0020093	25	36	GGGATTGTAGTTCAATCGGTCAGAG
t0020151	22	36	GTGGCTGTAGTTTAGTGGTTAG
t0020232	23	36	GGGGATATGGCGAAATTGGTAGA
t0020279	28	36	GTTGGTAGAGCTGAGGACTGTAGATCCT
t0020322	28	36	GGGGCTATAGCTCAGTTGGAAGAGCGCT
t0020349	28	36	GGGGGTATAGCTCAGCTGGGAGAGCGCT
t0020358	23	36	GGGGATGTAGCTCAGACGGTAGA
t0020359	28	36	GTGGACGTGCCGGAGTGGTTATCGGGCA
t0020372	25	36	GGTGGCTGTAATTTAGTGGTTAGAA
t0020417	28	36	GGGGCTGTAGCTCAGCTGGGCGAGCACC
t0020432	27	36	GATTTAGGCTCTGGTCCGAAAGGGCGT
t0020482	23	36	AGCGGGGTAGAGGAATTGGTCCA
t0020520	31	36	GGTACAGACCCGGGTTCGTTTCCCGGCTGGT
t0020525	23	36	TGGTAGAGCATTTGACTGCAGAT
t0020539	27	36	AGGGGTGTAGCTCAATTGGCAGAGCAG
t0020569	23	36	GCGGACGTAGCTCAGTTGGTAGA
t0020575	28	36	CGGACGTAGCTCAGTTGGTAGAGCGCAA
t0020645	29	36	GTTGGTAGAGCTGAGGACTGTAGATCCTT
t0020655	20	35	AGCGGGGTAGAGGAATTGGA
t0020659	29	35	GGGGATGTAGCTCAAACGGTAGAGCGCTC
t0020703	23	35	TTCGTGCCCCACGGTGGGCGCCA
t0020724	20	35	GGGGATGTAGCTCAGATGGC
t0020730	24	35	CTTCTAATCAGGCGATTGTGGGTT
t0020775	26	35	GGGGATGTAGCTCAAACGGTAGAGCA
t0020780	27	35	GCGGGTGTAGTTCAATGGCAGAACGGC
t0020784	26	35	GTGGACGTGCCGGAGTGGTTATCGGT
t0020798	30	35	GGGGATGTAGCTCAAACGGTAGAGCTCTCG
t0020830	29	35	TCCATTGTCGTCTAGTCCGGTTAGGATAC
t0020940	25	35	GCGCCCTTCGTCTATCGGTTAGGAC
t0020975	29	35	CGGGGTGTAGCTCAGTCTGGTAGAGCGCT
t0020985	25	35	GCGGGTGTAGTTCAATGGCAGAACG
t0021025	28	35	GCGGGTGACCCGGGTTCGTTCCCCGGCA
t0021044	22	35	GCGGGGATAGCTCAGTTGGGCG
t0021047	27	35	GCGGTCGTGGCGGAATTGGTAGACGCG
t0021095	27	35	GTCTGGTGGTCTAGCGGTATGATTCTC
t0021130	28	35	GGGGCGGTAGCTCAGCCGGTTAGAGCAG
t0021133	26	35	AGGGGTGTAGCTCAATTGGCAGAGCA
t0021152	28	35	GGGGCTGTAGCTCAGATGGGAGAGCGCT
t0021158	25	35	GGGGATATGGCGAAATTGGTAGACG
t0021162	28	35	GCGTTTGTAGTCCAACGGTAAGGATAAT
t0021194	28	35	GCGGGTGTAGCTCAGTTGGTAGAGCGCA
t0021312	23	34	GGTTAGGATACTCGGCTCTCACC
t0021331	27	34	GCCGGTGTAGCTCAGTTGGTAGAGCAG
t0021343	27	34	GAGCTGAGGACTGTAGATCCTTAGGTC
t0021351	23	34	GGGGATGTAGCTCAGATGGTGAG
t0021416	21	34	TCGAATCCTGCTGTCGACGCC
t0021418	22	34	GCGGACGTAGCGCAGCTGGTAG
t0021434	24	34	CTCAGTTGGTAGAGCTGAGGACTG

t0021447	27	34	CCGGATGGTGGAATGCAGACACGGCGA
t0021474	21	34	TAAGCAGAAGGCCGTAGGTTC
t0021518	23	34	AGCGGTGTAGCTCAGTCGGTAGA
t0021579	25	34	CCGGATGGTGGAATGCAGACACGGC
t0021718	24	34	GGGGATGTAGCTCATATGGTAGAG
t0021742	25	34	GAGCGGAGGACTGTAGATCCTTAGG
t0021759	30	34	CGGGGTGTAGCTCAGCCTGGTAGAGCACTA
t0021775	24	34	GCGCCTGTAGCTCAGTGGATAGAG
t0021780	24	34	GGGGGTGTAGCTCATATGGTAGAA
t0021781	22	34	GGAGGTATGGCTGAGTGGCTTA
t0021784	29	34	GGGCCTGTAGCTCAGAGGATTAGAGCACG
t0021859	27	33	GGGCCTGTAGCTCAGTTGGGAGAGCGC
t0021985	24	33	GGGGATATGGCGAAATTGGTAGAC
t0022043	25	33	GTGGCTGTAGTTTAGTGGTTAGAAT
t0022071	23	33	GGGTCCATAGCTCAGTGGTAGAG
t0022099	23	33	TGGTAGAGCTGAGGACTGTAGAT
t0022121	29	33	CAGTGGTAGAGCATTTGACTGCAGATCAA
t0022160	29	33	GGGGCTGTAGCTCAGCTGGGAGAGCGCCT
t0022186	24	33	GGGATGTAGCTCAGATGGTAGAGC
t0022284	21	33	GCGAGAGGTACGGGGATCGTT
t0022287	21	33	GTGGCTGTAGTTTAGTGGTAA
t0022397	29	33	GGAGTCGTGACCGAGTGGCCGAAGGTGCT
t0022470	27	33	GGTAGAGCGGAGGACTGTAGATCCTTA
t0022482	23	33	TTGCGAGAGGTACGGGGATCGTT
t0022526	28	32	GCCCAGGTGGCGGAATTGGTAGACGCAT
t0022631	26	32	GGTAGAGCGGAGGACTGTAGATCCTT
t0022636	26	32	GCGGGTGTAGCTCAGGGGTAGAGCAC
t0022665	28	32	GGGTCTGTAGCTCAGTTGGTCAGAGCGC
t0022679	30	32	CGGGGTGTAGCTCAGCCTGGTAGAGCGCTA
t0022720	26	32	TGGTAGAGCGGAGGACTGTAGATCCT
t0022784	27	32	GCACTCGTAGCTTAACGGATCGAGCAT
t0022814	25	32	GCGGATATGGTCGAATGGTAAAATT
t0022836	21	32	GGGGATGTAGCTCAGATGGTG
t0022838	25	32	CTCAGTTGGTAGAGCTGAGGACTGT
t0022860	29	32	CGGGCTGTAGCGCAGCTTGGTAGCGCACT
t0022894	24	32	CCGACCTTAGCTCAGTTGGCAGAG
t0022907	21	32	GTTGTCGTGGTGTAGTTGGTT
t0022983	21	32	CCGGAGCGGGAGATTGTGGGT
t0023019	30	32	GCCCTTTTAACTCAGCGGTAGAGTAACGCC
t0023111	19	32	CGGGGTAGAGGAATTGGTC
t0023122	20	32	GTGGCTGTAGTTTAGTGGTT
t0023202	27	31	GCCCCAATAGCTCAGTCGGCAGAGCGT
t0023247	28	31	GGAGTGGTAGTTCAGTCGGTTAGAATAC
t0023358	23	31	GGTGGCTGTAATTTAGTGGTTAG
t0023394	29	31	GTGGTAGAGCATTTGACTGCAGATCAAGA
t0023417	21	31	TGGGATGTAGCTCAAATGGTA
t0023428	26	31	GCGGATATGGTCGAATGGTAAAATTT
t0023468	31	31	GGGGATGTAGCTCAAACGGTAGAGCGCTCGC
t0023502	18	31	GGGGTGGTGGCGCAGTTG
t0023506	25	31	GACTTCTAATCAGGCGATTGTGGGT

t0023517	27	31	GGAGTCGTGACCGAGTGGCCGAAGGTG
t0023556	23	31	GGGGTGTAGCTCATATGGTAGAG
t0023618	27	31	GCGGGCGTGGCGGAATTGGTAGACGCG
t0023667	21	31	GCGCCTGTAGCTCAGTGGAGA
t0023680	27	31	TCCGGAGTAGCTCAGTGGTAGAGCAGG
t0023691	27	31	GCGGGTGTAGTTCAATGGTAGAACGGC
t0023806	28	31	AGGGGTGTAGCTCAATTGGCAGAGCAGC
t0023866	25	31	AGCGGTGTAGCTCAGTCGGTAGAGC
t0023934	26	31	AGCGGTGTAGCTCAGTCGGTAGAGCA
t0023953	30	30	CGGGGTGTAGCTCAGTCTGGTAGAGCGCTA
t0023982	23	30	GGGGATGTAGCTCAGATGGGAGA
t0024041	26	30	CGGGGTGTAGCGCAGCTTGGCAGCGC
t0024054	29	30	GGGTCTGTAGCTCAGTTGGTTAGAGCACC
t0024067	29	30	GGGGCTGTAGCTCAGTTGGGAGAGCGTCT
t0024151	29	30	GCCCAGGTGGCGGAACTGGTAGACGCGCT
t0024279	27	30	GGTAGAGCATTTGACTGCAGATCAAGA
t0024380	30	30	GCGGACGTAGCTCAGTTGGTCGAGCGCAAC
t0024416	19	30	GGGGTGGTGGCGCAGTTGG
t0024424	26	30	TGGTAGAGCATTTGACTGCATATCAA
t0024436	20	30	GGACTAGACGACAATGGAAA
t0024455	24	30	AGCGGGGTAGAGGAATTGGTCAAA
t0024497	24	30	GTCTGGGTCGTGTAGTTGGTTATC
t0024503	22	30	GGGGTTGTAGCTCAAATGGTAT
t0024519	21	30	AGCGGGGTAGAGGAATTGGTT
t0024548	23	30	AGATTTGAAATCTGTTGGGCTTC
t0024554	20	30	CCGACCTTAGCTCAGTTGGC
t0024559	21	30	CGTGTCGTGGTGTAGTTGGTT
t0024563	27	30	GCGGATGTGGCGGAACTGGTAGACGCG
t0024656	26	30	GACTTCTAATCAGGCGATTGTGGGTT
t0024665	25	30	GGAGTCGTGACCGAGTGGCCGAAGG
t0024698	20	30	CCGAAAGGGCGTGGGTTCAT
t0024803	28	29	GCCCCCGTTGTGTAGCGGCCTAGCACGC
t0024844	22	29	GTTGTCGTGGTGTAGTTGGTTA
t0024948	25	29	GGGGATGTAGCTCAAACGGTAGAGT
t0024966	29	29	TCCCCTGTAGCTCAATTGGCAGAGCAGCC
t0024977	21	29	GCGGGTGTAGTTCAGTGGTAG
t0024999	23	29	AGTTGGTAGAGCTGAGGACTGTA
t0025016	22	29	TGGGATGTAGCTCAAATGGTAG
t0025030	20	29	GGGGTGTAGCTCATATGGTA
t0025067	20	29	GCCCGTCTAGCTCAGTTGGT
t0025148	29	29	GGGGATATAGCTCAGCTGGGAGAGCGCGT
t0025154	26	29	GGAGTCGTGACCGAGTGGCCGAAGGT
t0025202	27	29	GTGGACGTGCCGGAGTGGTTATCGGGC
t0025224	24	29	GGGATTGTAGTTCAATCGGTCAGA
t0025262	29	29	GCGGGTGTAGTTTAATGGTAGAACATCAG
t0025266	30	29	AGGGGCATAGCTCAATTGGCAGAGCGTCGG
t0025284	21	29	GGGTTGTAGCTCAAATGGTAG
t0025309	22	29	AGTTGGTAGAGCTGAGGACTGT
t0025322	26	29	GGGTTCGTGCCCCACGGTGGGCGCCA
t0025350	29	29	GGGTCTGTAGCTCAGCTGGTTAGAGCACC

t0025353	28	29	GGTGGTTGTAGCTCAGTTGGTAGAGTCC
t0025547	23	29	GCGGATATGGTCGAATGGTAAAA
t0025564	25	29	GCCCAGATGGCGGAATTGGTAGACG
t0025578	25	29	GGGGATGTAGCTCAGATGGTAGAAA
t0025603	22	28	CGGGGTGTAGCGCAGCTTGGCA
t0025652	28	28	TGGTAGAGCGGAGGACTGTAGATCCTTA
t0025657	20	28	GCACCAGTTGTCTAGTGGTA
t0025678	24	28	CTAGTTGGTTAGGATACTCGGCTC
t0025733	26	28	GTCCGGGTGGCGGAATGGCAGACGCG
t0025735	20	28	GCGGGTGTAGTTTAATGGTA
t0025747	28	28	GTCTGTAGTTCGTTCCTGCATGGGGGCA
t0025792	24	28	AGCGGGGTAGAGGAATTGGTCAAT
t0025808	25	28	AGCGGGGTAGAGGAATTGGTCAATT
t0025853	23	28	TAAGCAGAAGGCCGTAGGTTCGT
t0025922	23	28	AGGGATGTAGCGCAGCTTGGTAG
t0025931	27	28	TAGAGCTGAGGACTGTAGATCCTTAGG
t0025940	24	28	GGGTGTAGTTTAATGGTAGAACAT
t0026097	26	28	GCTCAGTGGTAGAGCATTTGACTGCA
t0026108	26	28	AGCGGGGTAGAGGAATTGGTCAACTT
t0026130	26	28	GCCCTCATCGTCTAGCGGCCTAGGAC
t0026173	20	28	AGCGGTGTAGCTCAGTCGGT
t0026187	23	28	ATTTGAAATCTGTTGGGCTTCGC
t0026203	25	28	TCCGGTATGGTGTAGTGGCTAACAT
t0026269	24	28	GGGGACGTAGCTCAGCTGGGAGAG
t0026290	21	28	GGGGATGTAGCTCAGATGGAA
t0026317	24	28	GGGATGTAGCTCAAACGGTAGAGC
t0026372	21	28	GCGGAATAGGCAGACGCGCTG
t0026396	22	28	AGTTGGTTAGGATACTCGGCTC
t0026398	23	28	GCGGGGTAGAGGAATTGGTCAAC
t0026474	29	28	GCGGCTGTGGTGGAACTGGTAGACACGCT
t0026487	25	27	GGGGATGTAGCTCAGATGGTAGAGT
t0026499	22	27	CTAGTTGGTTAGGATACTCGGC
t0026538	20	27	GGTAGAGCATTTGACTGCAG
t0026544	20	27	GGAGGTATGGCTGAGTGGCT
t0026656	29	27	CGGGTGGCGGAATGGCAGACGCGCTAGCT
t0026669	23	27	GTCTGGGTAGTGTAGTTGGTTAT
t0026702	24	27	GCGGATATGGTCGAATGGTAAAAT
t0026734	22	27	CTTCTAATCAGGCGATTGTGGG
t0026777	24	27	GCGGGAGTAGCTCAGTTGGTAGAG
t0026948	26	27	GGGGATGTAGCTCAGATGGTCGAGCT
t0026960	19	27	CGAAAGGGCGTGGGTTCAT
t0026993	27	27	GCTTTGTGGTCTAGCGGTATGATTCTC
t0026995	26	27	GGGGACGTAGCTCAGCTGGGAGAGCG
t0027035	25	27	CGCGGGGTAGAGCAGTTTGGTAGCT
t0027103	20	27	AGGATACTCGGCTCTCACCC
t0027144	25	27	GCGGGTGTAGCTCAGTTGGTAGAGC
t0027145	19	27	ACACTGAAGGTCTCCGGTT
t0027151	26	27	GCGCTCGTGGCGGAATTGGTAGACGC
t0027164	24	27	GGTGTTGTAGCTCAAATGGTAGAG
t0027197	23	27	GTTGAGATGGCCGAGTTGGTCTA

t0027232	19	27	CGGGATGTAGCTCAGATGG
t0027243	22	27	GTTGGTAGAGCTGAGGACTGTA
t0027369	22	27	CTAGCGGTTAGGACATTGGACT
t0027437	21	27	GGTAGAGCATTTGACTGCAGA
t0027464	22	26	CTCAGTTGGTAGAGCTGAGGAC
t0027483	25	26	GGCGACCCGGGTTCAAATCCCGGCA
t0027509	27	26	GGGCGGTTAGCTCAGCGGTAGAGCACT
t0027512	26	26	GTCTGGGTGGTGTAGTTGGTCATCAC
t0027515	30	26	GCCGGTGTAGCTCAGCTGGCTAGAGCAGCT
t0027624	23	26	TCAGTTGGTAGAGCTGAGGACTG
t0027702	28	26	GGCCTGGTGGTCTAGTGGTATGATTGTC
t0027754	26	26	GCGGATATGGCGAAATTGGTAGACGT
t0027807	25	26	GTGGACGTGCCGGAGTGGTTATCGG
t0027842	25	26	GGTAGAGCGGAGGACTGTAGATCCT
t0027915	23	26	GGGTTGTAGCTCAAATGGTAGAG
t0027942	26	26	GTGGCTGTAGTTTAGTGGTTAGAATT
t0027983	26	26	GGGTGTAGTTTAATGGTAGAACATCA
t0028046	25	26	TCCGGAGCGGGAGATTGTGGGTTCG
t0028071	20	26	CGAAAGGGCGTGGGTTCATA
t0028083	19	26	GACCGCATAGCGCAGTGGA
t0028106	22	26	GCGGGTGTAGTTCAGTAGTAGA
t0028117	24	26	CCGACCTTAGCTCAGTTGGTAGAG
t0028134	25	26	CAGTGGTAGAGCATTTGACTGCAGA
t0028200	22	26	GGGGATGTAGCTCAGATGGTGG
t0028202	19	26	GGGGATATGGCGAAATTGG
t0028230	26	26	GGGGCTGTAGCTCAGCTGGGCGAGCA
t0028257	22	26	GGCGTCGTGGTGTAGTTGGTTA
t0028277	24	26	GCGGGTGTAGTTTAATGGTAGAAC
t0028292	19	26	GGATGTAGCTCAGATGGTA
t0028313	27	26	GGGGATGTAGCTCAGATGGTAGAGCCT
t0028318	27	26	CCCCGTTGTGTAGCGGCCTAGCACGCC
t0028340	27	26	GGGGCTGTAACTCAGTGGTAGAGTTCT
t0028383	19	26	TGTGTCGTGGTGTAGTTGG
t0028393	26	26	GTCTGGTGGTCTAGCGGTATGATTCT
t0028517	26	25	GCGCGGGTGGCGGAATAGGCAGACGC
t0028577	28	25	GGGTCTGTAGCTCAGTTGGTTAGAGCAC
t0028588	24	25	AGTTCGAATCTCTCAGGCGACGCC
t0028608	23	25	GGGATTGTAGTTCAATCGGTCAG
t0028615	29	25	GGGGCGGTAGCTCAGCCGGTTAGAGCAGC
t0028639	21	25	GGGATGTAGCTCAAATGGTAG
t0028644	26	25	GCGGACGTAGCGCAGCTGGTAGCGCA
t0028672	30	25	GCGGAAATAGCTTAATGGTAGAGCATAGCC
t0028684	23	25	GGGGATGTAGCTCAGATGGTAGC
t0028686	26	25	GGCATTTGGTCTAGTGGTATGATTCT
t0028765	25	25	GGTTCTATGGTCTAGCGGTTAGGAC
t0028782	28	25	GGCCTTGTGGTCTAGTAGTATGATTCTT
t0028815	29	25	GGGCATTTGGTCTAGTGGTATGATTCTCG
t0028834	24	25	CAGTTGGTAGAGCTGAGGACTGTA
t0028909	31	25	GGGGATGTAGCTCAAACGGTAGAGCTCTCGC
t0029023	27	25	GCGCATCTGGTGTAGTGGTATCATAGT

t0029100	22	25	TGGTAGAGCATTTGACTGCAGA
t0029129	26	25	GGGATTGTAGTTCAATCGGTCAGAGC
t0029153	28	25	GCCCGGATGGTGGAATGCAGCCACGGCG
t0029172	21	25	TGTGGCTGTAGTTTAGTGGTT
t0029186	27	25	GCGGATATGGTCGAATGGTAAAATTTC
t0029257	24	25	CGGGATGTAGCTCAGATGGTAGAG
t0029280	23	25	GGGGGTGTAGCTCAGCTGGGAGA
t0029305	28	25	ACGGACTGTAAATTCGTTGACGATATGT
t0029310	31	25	GAGGGCGTGGGTTCAGATCCCACTTCTGACA
t0029311	24	25	GCCCAGATGGCGGAATTGGTAGAC
t0029332	29	25	AGTTGGTAGAGCTGAGGACTGTAGATCCT
t0029345	29	25	GGGGCTATAGCTCAGCTGGGAGAGCGCTT
t0029370	24	25	GGGGATGTAGCTCAGACGGTAGAG
t0029381	27	25	GGAGTGGTAGTTCAGTCGGTTAGAATA
t0029432	25	25	GTAGAGCGGAGGACTGTAGATCCTT
t0029442	29	25	GGGGATGTAGCTCAGATGGTAGAGCTCTC
t0029448	24	25	GCGTTTGTAGTCCAACGGTAAGGA
t0029457	26	25	GCACCAGTGGTCTAGTGGTAGAATAG
t0029483	26	25	GCACTCGTAGCTTAACGGATAGAGCA
t0029542	24	25	TGGTAGAGCTGAGGACTGTAGATC
t0029589	28	25	GCGGGTGTAGTTCAATGGTAGAACGGCA
t0029659	26	24	GGTGGGTGTAGCTCAGCTGGTAGAGC
t0029700	28	24	GCGGCTGTGGTGGAACTGGTAGACACGC
t0029709	20	24	AGGTGGTTAGGATACTCGGC
t0029716	26	24	GCGGATGTGGCGGAACTGGTCGACGC
t0029793	27	24	GCACCAGTGGTCTAGTGGTAGAATAGT
t0029824	25	24	TCCGTTGTAGTCTAGTTGGTTAGGA
t0029882	26	24	GGGGCTGTAGCTCAGCTGGGAGAGCG
t0029896	25	24	GTCGTTGTAGTATAGTGGTACGTAT
t0029931	20	24	TGCGGATGTAGCCAAGTGGA
t0029949	24	24	GGGGATGTAGCTCAGATGGTAAAG
t0029952	20	24	AAGCAAGGACTCTACCACGC
t0029963	23	24	CCGGAGCGGGAGATTGTGGGTTC
t0029979	21	24	GGTTCTATGGTGTAGTGGTTA
t0030017	30	24	GCCCAGATGGCGGAATTGGTAGACGCGCAG
t0030021	28	24	GCGTGAATGGTTTAGTGGTACAATTCTC
t0030043	25	24	GCGCATCTGGTGTAGTGGTATCATA
t0030058	26	24	GGAGGTATGGCTGAGTGGCTTAAGGC
t0030087	21	24	GCCCCAATAGCTCAGTCGGCA
t0030185	20	24	TCCGGAGCGGGAGATTGTGG
t0030200	26	24	TGGTAGAATAGTACCCTGCCACGGTA
t0030206	27	24	GGGGATATGGCGAAATTGGTAGACGCT
t0030277	28	24	GGAGAGATGGCTGAGTGGACTAAAGCAG
t0030337	20	24	GCGCCTGTAGCTCAGTGGAT
t0030363	24	24	GTAGAGCGGAGGACTGTAGATCCT
t0030418	28	24	GCGTTTGTAGTCCAACGGTCAGGATAAT
t0030634	20	24	GGATGTAGCTCAGATGGTAG
t0030670	27	24	GCGGAAGCGTGGTGGGCCCATAACCCA
t0030678	22	24	GGTAGAGCATTTGACTGCAGAT
t0030745	22	24	GGCGGAATAGGCAGACGCGCTG

t0030786	24	24	GCGGGTGTAGCTCAGTTGGTAGAG
t0030794	25	24	GGTGATGTAGCTCAGATGGTAGAGC
t0030890	28	23	GGCGCGGTAGCAAAGCGGTTATGCCCCG
t0030950	29	23	GGGTCTGTAGCTCAGCTGGTTAGAGCACT
t0030961	19	23	GGGGATGTAGCTCATGGTA
t0030977	29	23	GGGTGTAGTTTAATGGTAGAACATCAGCT
t0030981	25	23	GGTTCGTGCCCCACGGTGGGCGCCA
t0031013	21	23	GCGGGGATAGCTCAGTTGGGC
t0031051	28	23	GGGGCTGTAGCTCAGCTGGGCGAGCGCT
t0031091	19	23	GCGGGGATAACTCAGTTGG
t0031245	24	23	AGATTTGAAATCTGTTGGGCTTCG
t0031301	22	23	AGCGGGGTAGAGGAATTGGTCC
t0031306	19	23	GGGGATGTAGTTCAGATGG
t0031311	24	23	GTGGCTGTAGTTTAGTGGTTAGAA
t0031332	27	23	GCGGGCGTAGCTCAGGGGTAGAGCACA
t0031397	24	23	GGTCCGGTGGTGTAGTTGGTTATC
t0031435	30	23	GGGGCTGTAGCTCAGCTGGGAGAGCGCTAG
t0031487	26	23	GCGCATCTGGTGTAGTGGTATCATAG
t0031521	29	23	GGGTCTGTAGCTCAGTTGGTCAGAGCGCA
t0031557	25	23	GGTGGTTGTAGCTCAGTTGGTAGAG
t0031589	26	23	GGGGATGTAGCTCAAACGGTAGAGTT
t0031607	27	23	GAGTGGCGCAGCGGAAGCGTGGTGGGC
t0031626	27	23	GGCGCGGTAGCAAAGCGGTTATGCCCC
t0031635	25	23	GCGTTTGTAGTCCAACGGTAAGGAT
t0031709	24	23	TCTAGTTGGTTAGGATACTCGGCT
t0031767	19	23	AGGGATATAACTCAGCGGT
t0031793	21	23	TGGTTAGGATACTCGGCTCTC
t0031800	28	23	GGGTGTAGTTTAATGGTAGAACATCAGC
t0031803	20	23	GGGTCCATAGCTCAGTGGTA
t0031848	24	23	TGTGTCGTTGTGTAGTTGGTTATC
t0031900	23	23	AGGGATATAACTCAGCGGTAGAG
t0031905	28	23	TCCGGTGGTGTAGTTGGTTATCACGTCA
t0031943	20	23	GGAGTTGTAGCTCAAATGGT
t0031970	25	23	GGGGATGTAGCTCAGATGGTAGAAT
t0031974	21	23	TCGAGAGAGGGCGTGGGTTCA
t0032046	20	23	GTTAGGATACTCGGCTCTCA
t0032063	26	23	GCGGGTGTAGTTCAGTAGTAGAACAT
t0032089	27	23	GGTTCTATGGTCTAGCGGTTAGGACAT
t0032127	24	23	GGTTCTATGGTGTAGTGGTTAGCA
t0032144	23	22	TGTGGCTGTAGTTTAGTGGTTAG
t0032154	22	22	GGGGATGGAGCTCAGATGGTAG
t0032177	23	22	GGTGTTGTAGCTCAAATGGTAGA
t0032197	22	22	GGGGATGTAGCTCAGATGGTCT
t0032234	30	22	GCACCAGTCGTCTAGTGGTAGAATAGTACC
t0032248	27	22	GCGTTTGTAGTCCAACGGTCAGGATAA
t0032317	22	22	GGGGATGTAGCTCAGATGGGAG
t0032351	25	22	GGGTCTGTAGCTCAGTTGGTTAGAG
t0032380	19	22	GCGTTTGTAGTCCAACGGA
t0032383	27	22	GCGCGGGTGGCGGAATAGGCAGACGCG
t0032392	27	22	GTCGTTGTAGTATAGTGGTGAGTATTC

t0032410	25	22	GTGGCTGTAGTTTAGTGGTAAGAAT
t0032450	23	22	GCGGGTGTAGTTCAGTAGTAGAA
t0032474	28	22	GGTGTCGTGGTGTAGTTGGTTATCACAT
t0032481	24	22	TGGGTTCGTTTCCCACAGACGGCG
t0032581	23	22	GGTAGAGCATTTGACTGCAGATC
t0032582	25	22	TCTAGTTGGTTAGGATACTCGGCTC
t0032614	27	22	GTTGTAGTATAGTGGTAAGTATTCCCG
t0032643	28	22	GGGGATGTAGCTCAGATGGTAGAGCTCT
t0032654	20	22	GCGGAAATAGCTTAATGGTA
t0032659	26	22	TCAGTGGTAGAGCATTTGACTGCAGA
t0032677	23	22	TAGTTGGTTAGGATACTCGGCTC
t0032690	22	22	TGCGAGAGGTACGGGGATCGTT
t0032723	25	22	CTCCGGAGCGGGAGATTGTGGGTTC
t0032742	26	22	CAGTTGGTAGAGCTGAGGACTGTAGA
t0032750	26	22	CGGGTGGCGGAATGGCAGACGCGCTA
t0032832	28	22	GGGGCTGTAGCTCAGTTGGGAGAGCGTT
t0032943	22	22	GCCCCAATAGCTCAGTCGGCAG
t0032984	26	22	GGTTCCATGGTCTAGCGGTTAGGACA
t0033068	30	22	GGGGCTGTAGCTCAGCTGGGAGAGCGCCGC
t0033091	30	22	AGTTGGTAGAGCTGAGGACTGTAGATCCTT
t0033104	26	22	GGGGATGTGGCGGAATGGCAGACGCG
t0033138	30	22	GCCGAAGTGGTGGAACTGGTAGACACGTAC
t0033150	25	22	TGGGGTGTAGCTCATATGGTAGAGC
t0033174	27	22	GGGCGATTAACTCAGCGGTAGAGTGCC
t0033194	21	22	TCTAGCGGTTAGGACATTGGA
t0033196	28	22	GGGGCTGTGGCGCAGACTGGTAGCGCAC
t0033290	22	22	GCGCCTGTAGCTCAGTGGATAG
t0033292	28	22	GCCCAGGTGGCGGAATTGGTCGACGCAC
t0033297	27	22	CGCGGGGTGGAGCAGTTCGGTAGCTCG
t0033330	20	22	GGTGCTGTGGTGTAGTGGTT
t0033380	26	22	GGGGATATGGCGAAATTGGTAGACGC
t0033385	25	22	GGATGGATGTCTGAGCGGTTGAAAG
t0033450	22	22	GGGGATGTAGCTCAGATGGTAC
t0033497	22	22	GACCGCATAGCGCAGTGGATTA
t0033520	24	22	TCAGTTGGTAGAGCTGAGGACTGT
t0033526	27	22	GCGGACGTGGCGGAATTGGTAGACGCA
t0033546	23	22	GGGTCTGTAGCTCAGTCGGTTAG
t0033708	24	21	CGGGTGGCGGAATGGCAGACGCGC
t0033768	22	21	GTTGAGATGGCCGAGTTGGTCT
t0033804	26	21	TCTAGTTGGTTAGGATACTCGGCTCT
t0033878	26	21	GGGGCTGTGGCGCAGACTGGTAGCGC
t0033903	22	21	GGGGATTTAGCTCAGATGGTAG
t0033941	21	21	GGCGCGGTAGCAAAGCGGTTA
t0033999	20	21	TGTGGCTGTAGTTTAGTGGT
t0034056	26	21	GCGGGTGTGGCGGAACTGGTAGACGC
t0034148	28	21	GTCGGGATAGCTCAGCTGGTAGAGCAGA
t0034197	29	21	GGGGCTATAGCTCAGTTGGTAGAGCGCCT
t0034226	20	21	GGGGATGTAGCTCATATGGT
t0034230	27	21	CTCAGTGGTAGAGCATTTGACTGCAGA
t0034249	26	21	GGGCCTGTAGCTCAGAGGATAGAGCA

t0034258	21	21	ATCAGAGTGGCGCAGCGGAAG
t0034265	20	21	TTGGTTAGGATACTCGGCTC
t0034275	24	21	TAGTTGGTTAGGATACTCGGCTCT
t0034287	27	21	ACGGGCATAGTTCAACGGTAGAATAGA
t0034288	26	21	GTCTGGGTGGTGTAGTTGGTTATCAC
t0034336	26	21	GCCCGGATGGTGGAATGCAGCCACGG
t0034359	28	21	GCGGCTGTAGCTCAGTGGATAGAGTATT
t0034360	27	21	GTCGTTGTAGTATAGTGGTAAGTATTC
t0034377	23	21	GCGGAAATAGCTTAATGGTAGAG
t0034533	23	21	GGTGATGTAGCTCAAATGGTAGA
t0034569	30	21	GGGCGTTTGGTCTAGTGGTATGATTCTCGC
t0034577	27	21	GCGGGTGTAGCTCAATGGTAGAGCAGA
t0034591	25	21	GCTCGAGTGGTGGAATTGGTAGACA
t0034659	25	21	AGATTTGAAATCTGTTGGGCTTCGC
t0034694	26	21	GCGGGGTAGAGGAATTGGTCAACTCA
t0034712	28	21	GCGGGTGTAGCTCAATGGTAGAGCAGAA
t0034821	22	21	GGTGTTGTAGCTCAAATGGTAG
t0034839	21	21	GGCGTCGTGGTGTAGTTGGTT
t0034863	24	21	GGCAAGATGGCGGAGTGGTTAACG
t0034894	26	21	GCCGAAGTGGTGGAACTGGTAGACAC
t0034910	32	21	GGGGCTGTAGCTCAGTTGGGAGAGCGCCTGAC
t0035047	23	21	CTCGAGAGAGGGCGTGGGTTCAT
t0035065	25	21	CGGATGGTGGAATGCAGACACGGCG
t0035068	29	21	GGTGGCTGTAGCTCAGTTGGCAGAGCACC
t0035070	26	21	CGGGCGGCCCGGGTTCGTTTCCCGGC
t0035105	26	21	GCGGGTGTAGTTCAATGGTAGAACGC
t0035243	23	20	GTTAGGATACTCGGCTCTCACCC
t0035281	25	20	TGGGTTGTAGCTCAAATGGTAGAGC
t0035306	28	20	TTGGTAGAGCTGAGGACTGTAGATCCTT
t0035411	29	20	GTGGACGTGCCGGAGTGGTTATCGGGCAT
t0035471	26	20	GGGGCTGTAGCTCAGTTGGGAGAGCG
t0035481	23	20	GTTGTCGTGGTGTAGTTGGTTAT
t0035492	26	20	CGGGGTGTAGCTTAGCCTGGTAGAGC
t0035550	28	20	GGAGCTGTAGCTCAGTTGGGAGAGCGCT
t0035559	28	20	GGGGTTGTAGCTCAAATGGTAGAGCTCG
t0035579	29	20	GGCCCGTTCGTCTATCGGTTAGGACGCCA
t0035670	21	20	GGATGGCGGAATTGGTAGACG
t0035710	21	20	CTAGGTGGTTAGGATACTCGG
t0035728	29	20	CGGGGTGTAGCTTAGTCTGGTAGAGCGCT
t0035754	23	20	GGGGATGTAGCTTAGATGGTAGA
t0035834	27	20	GGGCGCGTGGTCTAGTGGTATGATTTT
t0035872	22	20	GGGGATGTAGCTTAGATGGTAG
t0035908	23	20	AGCGGGGTGTAGGAATTGGTCAA
t0035927	30	20	CCCCTGTAGCTCAATTGGCAGAGCAGCCGG
t0035985	27	20	TCCGGTATGGTGTAGTGGCTAACATTT
t0035989	23	20	GGTTCGTTTCCCGGCTGGTGCAC
t0036027	24	20	CGCGGGGTAGAGCAGTTTGGTAGC
t0036031	21	20	TGGATTGTAGTTCAATCGGTC
t0036118	27	20	GCGCCTGTAGCTCAGTGGATAGAGCTT
t0036122	20	20	GGGGATGTAGCTCAGAGGGT

t0036190	24	20 GGTGGCTGTATTTTAGTGGTTAGA
t0036261	25	20 GCGGGTGTAGCTCAGTCGGTTAGAG
t0036301	27	20 TCCATTGTCGTCTAGTCCGGTCAGGAT
t0036325	25	20 GGGAATGTAGCTCAGATGGTAGAGC
t0036359	24	20 TGTGTCGTCGTGTAGTTGGTTATC
t0036405	25	20 GCCCGGATGGCGGAATTGGTAGACG
t0036442	21	20 GGGGTGTAGCTCATATGGTAG
t0036467	31	20 GCGGACGTAGCTCAGTTGGTAGAGCGCAACC
t0036477	24	20 GGGGTTGTAGCTCAAATGGTAGAT
t0036570	24	20 CGCGGGATAGAGCAGTAGGCAGCT
t0036590	25	20 GCGGGTGTAGCTCAATGGTAGAGCA
t0036613	21	20 GGCGGAATGGCAGACGCGCTA
t0036616	29	20 GGTGGCTGTAGCTCAGTTGGTAGAGTCCA
t0036633	22	20 GGGATGTAGCTCAAATGGTAGA
t0036680	28	20 GCGGAAATAGCTTAATGGTAGAGCATAG
t0036682	23	20 AGATTGAGGTTCTGGTCCGAAAG
t0036762	25	20 GGTGGGTGTAGCTCAGCTGGTAGAG
t0036822	28	20 GCGCCTGTAGCTCAGTGGATAGAGCTTC
t0036835	21	20 GTCGTTGTAGTATAGTGGTAC
t0036906	31	19 GCCCTTTTAACTCAGCGGTAGAGTAACGCCA
t0036917	25	19 GCGGGTGTAGCTCAGTGGTAGAGCA
t0037030	18	19 GTCTGGTGTAGTTGGTTA
t0037105	21	19 GGGATTGTAGTTCAATCGGTT
t0037147	21	19 TGGTAGAGCATTTGACTGCAT
t0037253	26	19 GGGGGTGTAGCTCATATGGTAGAGCT
t0037299	27	19 GCCCAGGTGGCGGAACTGGTAGACGCG
t0037339	20	19 TCCTCAGTAGCTCAGTGGTA
t0037367	26	19 CCGGGTGGCGGAATGGCAGACGCGCT
t0037406	26	19 AGTGGTAGAGCATTTGACTGCAGATC
t0037463	28	19 GGGGATATAGCTCAGCTGGGAGAGCGCG
t0037487	29	19 GCGTTTGTAGTCCAACGGTAAGGATAATT
t0037582	23	19 TCCATTGTCGTCTAGTCCGGTAG
t0037586	30	19 CGGGCTGTAGCGCAGCTTGGTAGCGCACTT
t0037654	19	19 GTAGAGCGGAGGACTGTAG
t0037697	20	19 TAAGCAGAAGGCCGTAGGTT
t0037709	22	19 GGTGTCGTGGTGTAGTTGGTAT
t0037782	22	19 GGGTCCATAGCTCAGTGGTAGA
t0037843	29	19 GCCCTCATCGTCTAGCGGCCTAGGACGCC
t0037874	29	19 TCCGGCGTAGCTCAGTCGGTAGAGCGGGT
t0037886	26	19 GGGGCTATAGCTCAGCTGGGAGAGCG
t0037929	27	19 CGGACGTAGCTCAGTTGGTAGAGCGCA
t0037973	23	19 GGCGTCGTGGTGTAGTTGGTTAT
t0038009	22	19 CTCGTGAGAGGGCGTGGGTTCA
t0038026	25	19 TCAGTGGTAGAGCATTTGACTGCAG
t0038095	29	19 GGTGTAGTTTAATGGTAGAACATCAGCTT
t0038114	26	19 GCGTTTGTAGTCCAACGGTCAGGATA
t0038134	22	19 GGTGGCTGTAGTTTAGTGGTTC
t0038159	27	19 CCCGGATGGTGGAATGCAGACACGGCG
t0038248	20	19 GGTTAGGATACTCGGCTCTC
t0038315	21	19 GGCGGATGTGGCCAAGTGGAT

t0038406	24	19	GGGGATGTAGCTCAGATGGTAGGC
t0038453	26	19	GCCCGGATGGCGGAATTGGTAGACGC
t0038481	20	19	GTTGGTAGAGCGGAGGACTG
t0038525	23	19	GGGATGTAGCTCAAATGGTAGAG
t0038571	21	19	GGGTCCATAGCTCAGTGGTAG
t0038603	26	19	GGGGATGTAGCTCAGATGGTAGAGAA
t0038662	29	19	GCGTTTGTAGTCCAACGGTAGGATAATTG
t0038672	22	19	AGCGGGGTATAGGAATTGGTCA
t0038755	20	19	GGTGGTTAGGATACTCGGCT
t0038774	21	19	AAGCAGAAGGCCGTAGGTTCG
t0038792	27	18	GCCCGGATGGTGGAATGCAGCCACGGC
t0038827	21	18	CTCGAGAGAGGGCGTGGGTTC
t0038863	22	18	GCGGGTGTAGTTTAGTAGTAGA
t0038894	21	18	GCGTTTGTAGTCCAACGGTCC
t0038942	21	18	GGGGATGTAGCTCAGTTGGTA
t0038949	25	18	CAGTTGGTAGAGCTGAGGACTGTAG
t0038969	28	18	CGGGGTGTAGCGCAGCTTGGCAGCGCGC
t0038983	24	18	CGTGTCGTGGTGTAGTTGGTTATC
t0039008	24	18	GGGGATGTAGCTTAGATGGTAGAG
t0039019	22	18	GGGGGGGTAGCTCATATGGTAG
t0039024	27	18	GCCGGAGCGGTGGAATTGGCAGACACG
t0039026	24	18	GCGCATCTGGTGTAGTGGTATCAT
t0039041	27	18	GGAGAGATGGCTGAGTGGACTAAAGCT
t0039064	21	18	GTGGATCAAGGCAGTGGATTG
t0039123	23	18	GCCCCAATAGCTCAGTCGGCAGA
t0039163	19	18	TCAAGGCAGTGGATTGTGA
t0039192	30	18	CCGGGTGGCGGAATGGCAGACGCGCTAGCT
t0039200	23	18	CGTGTCGTGGTGTAGTTGGTTAT
t0039314	25	18	GGCGTCGTGGTGTAGTTGGTTATCA
t0039336	28	18	GCACTCGTAGCTTAACGGATCGAGCATC
t0039392	22	18	AGAGCGGAGGACTGTAGATCCT
t0039402	24	18	GGGGATGTAGCTCAGATGGTAATT
t0039438	31	18	GCACCAGTCGTCTAGTGGTAGAATAGTACCC
t0039601	27	18	GTTGTAGTATAGTGGTGAGTATTCCCG
t0039610	20	18	CCGACCTTAGCTCAGTTGGT
t0039619	26	18	GGGGGTGTAGCTCAGCTGGGAGAGCA
t0039642	30	18	GCACCAGTAGTCTAGTGGTAGAATAGTACC
t0039798	29	18	AGTGGTAGAGCATTTGACTGCAGATCAAG
t0039832	19	18	GGGGAGATACTCAAGCGGC
t0039953	28	18	TCCATTGTCGTCTAGTCCGGTCAGGATA
t0039982	28	18	GCGGAAGTAGCTCAGTTGGTAGAGCGCA
t0039999	20	18	GGGATGTAGCTCAAATGGTA
t0040040	30	18	GGGGCGGTAGCTCAGCCGGTTAGAGCAGCG
t0040068	20	18	GGTTCCATGGTCTAGCGGTT
t0040072	29	18	GCCCAGGTGGCGGAATTGGTAGACGCACT
t0040150	29	18	GCCCCAATAGCTCAGTCGGCAGAGCGTCT
t0040163	25	18	GGGTCTGTAGCTCAGCTGGTTAGAG
t0040171	28	18	GTGGTCGTGCCGGAGTGGTTATCGGGCA
t0040196	20	18	AGGTGGCGGAATTGGTAGAC
t0040254	27	18	TGGTAGAGCATTTGACTGCATATCAAG

t0040257	20	18	CGCGGGATAGAGCAGTAGGC
t0040287	28	18	GCGACTGTGGTGGAACTGGTAGACACGC
t0040296	27	18	GCGGGTGTAGCTCAGTCGGTTAGAGTG
t0040377	30	18	GGGGCTGTAGCTCAGCTGGGAGAGCGCCTG
t0040393	20	18	GGGTTGTAGCTCAAATGGTA
t0040432	20	18	GCGGATGTAGCTCAGATGGT
t0040456	23	18	GCGCCTGTAGCTCAGTGGAGAGC
t0040463	27	18	CTCAGTTGGTAGAGCTGAGGACTGTAG
t0040464	25	18	GGGTGTAGTTTAATGGTAGAACATC
t0040573	25	18	GTCGTTGTAGTATAGTGGTGCGTAT
t0040588	25	18	CGCGGGGTGGAGCAGTTCGGTAGCT
t0040642	27	18	GGGGCTATAGCTCAGTTGGTAGAGCGC
t0040653	28	18	ATTGGACTCTGAATCCAGTAACCCGAGT
t0040716	25	18	GTCTGGGTCGTGTAGTTGGTTATCA
t0040734	27	18	GGGGACGTAGCTCAGCTGGGAGAGCGT
t0040738	22	18	GGTGATGTAGCTCAAATGGTAG
t0040888	27	18	GGGTCTGTAGCTCAGCTGGTTAGAGCA
t0040938	29	18	GGGGCTGTAGCTCAGATGGGAGAGCGCTG
t0041020	21	17	CGGTACTGACCGGGGTTCGTT
t0041035	28	17	GCCCCAATAGCTCAGTCGGCAGAGCGTC
t0041098	20	17	GCGGGTGTAGCTCAGTCGGT
t0041105	26	17	GCGGCTGTAGCTCAGTTGGATAGAGT
t0041222	22	17	AGCGGGGTAGAGGAATTGGTCT
t0041249	24	17	GGTTAGGATACTCGGCTCTCACCC
t0041264	27	17	GGTGTCGTCGTGTAGTTGGTTATCACT
t0041272	28	17	CGGGGTGTAGCTTAGTCTGGTAGAGCGC
t0041276	28	17	GCACCAGTTGTCTAGTGGTAGAATAGTA
t0041302	22	17	CGCGGGGTGGAGCAGTTCGGTA
t0041339	24	17	CTCAGTGGTAGAGCATTTGACTGC
t0041352	26	17	TCCGGGTGGCGGAATGGCAGACGCGC
t0041428	27	17	CCGGTGGTGTAGTTGGTTATCACGTCA
t0041443	28	17	GGGGCTGTAGCTCAGTTGGGAGAGCGCA
t0041523	29	17	GCGGATATGGCGAAATTGGTAGACGTGCC
t0041543	23	17	CAGTTGGTAGAGCTGAGGACTGT
t0041575	20	17	AGGGATGTAGCTCAGATGGT
t0041725	29	17	GGACGATTAGCTCAGCTGGGAGAGCACCT
t0041753	23	17	GTGGCTGTAGTTTAGTGGTTAGA
t0041773	22	17	GCGGGGTAGAGGAATTGGTCCA
t0041804	21	17	CTTGCGAGAGGTACGGGGATC
t0041869	22	17	GTGGTAGAGCATTTGACTGCAT
t0041960	26	17	TGTGTCGTGGTGTAGTTGGTTATCAC
t0042014	28	17	GGTCTGGTCGTCTAGCGGTATGATTCTC
t0042028	25	17	GGGCGTTTGGTCTAGTGGTATGATT
t0042031	30	17	GCGTTTGTAGTCCAACGGTTAGGATAATTG
t0042042	28	17	GGGGATGTAGCTCAGATGGTAGAGCCTC
t0042133	28	17	GGTACAGACCCGGGTTCGTTTCCCGGCT
t0042182	24	17	AGGGTCGTAGCTCAATTGGTAGAG
t0042184	28	17	ACGGGCGTAGTTCAAGGGTAGAATAGCG
t0042203	28	17	CCGAGAGACCCGGGTTCAGGTCCCGGCA
t0042226	30	17	GTTAGGACATTGGACTCTGAATCCAGTAAC

t0042249	25	17	GAGTGGCGCAGCGGAAGCGTGGTGG
t0042263	21	17	AGGGATATAACTCAGCGGTAG
t0042273	26	17	TCCGTTGTAGTCTAGTTGGTTAGGAT
t0042279	29	17	AGGTCTGTAGTTCGTTCCTGCATGGGGGC
t0042312	25	17	CTCAGTGGTAGAGCATTTGACTGCA
t0042349	25	17	GGTGATGTAGCTCAAACGGTAGAGC
t0042353	22	17	GCCCGTCTAGCTCAGTTGGTAG
t0042364	21	17	CTCGTGAGAGGGCGTGGGTTC
t0042371	25	17	CGTGTCGTGGTGTAGTTGGTTATCA
t0042415	26	17	GCGCGGATGGCGGAATTGGTCGACGC
t0042442	20	17	GGGGGTGTAGCTCATGGTAG
t0042610	22	17	TTGGTAGAGCTGAGGACTGTAG
t0042633	20	17	CGTGTCGTGGTGTAGTTGGT
t0042685	23	17	GCGGAAGCGTGGTGGGCCCATAA
t0042690	22	17	GGGGATGTAGCTCAGATGGTGA
t0042701	30	17	GGGGCATAGCTCAATTGGCAGAGCGTCGGT
t0042717	25	17	GCGCCTGTAGCTCAGTGGACAGAGC
t0042719	27	17	CGGGTGGCGGAATAGGCAGACGCGCTG
t0042744	27	17	CCAGTGGTCTAGTGGTAGAATAGTACC
t0042833	19	17	AGTCCCGTAGCTCAGTTGG
t0042849	30	17	GCGGGTGGCGGAATAGGCAGACGCGCTGGA
t0042861	25	17	AGCGGGGGAGAGGAATTGGTCAACT
t0042888	24	17	GGTGATGTAGCTCAAATGGTAGAG
t0042928	20	17	GCGTTTGTAGTCCAACGGGT
t0042961	26	17	ACCAGTGGTCTAGTGGTAGAATAGTA
t0043115	20	17	TGCGGATGTGGCCAAGTGGA
t0043198	23	17	GGGGATGTAGCTCAGATGGTATT
t0043207	27	17	GGGGCTGTAGCTCAGCTGGGCGAGCAC
t0043358	24	16	GGGGTTGTAGCTCAAATGGTATAG
t0043359	29	16	TGGTCTGTAGCTCAGTCGGTTAGAGCACC
t0043394	23	16	ACACACTGAAGGTCTCCGGTTCG
t0043422	28	16	ACGGACTGTAAATTCGTTGACGATATGA
t0043463	22	16	TTCGAACCCTGCTGTCGACGCC
t0043469	19	16	GGACTAGACGACAATGGAA
t0043550	25	16	CGGGATGTAGCTCAGATGGTAGAGC
t0043635	30	16	GGGGCTGTAGCTCAGCTGGGCGAGCACCTG
t0043639	25	16	GGTGGCTGTAGCTCAGTTGGTAGAG
t0043749	19	16	GACAGTTTGGCCGAGTGGT
t0043762	29	16	GCGGGTGTAGCTCAGTTGGTAGAGCGCGA
t0043764	24	16	GGGGATGTAGCTCAGATGGTAGAC
t0043810	24	16	GGGGATGTAGCTCAGAGGTAGAGC
t0043850	29	16	GGGTGGTTAGCTCAGCGGTAGAGCACTGC
t0043856	26	16	TGGTAGAGCATTTGACTGCACATCAA
t0043890	19	16	GGGGATGTAGCTCAGGGTA
t0043931	26	16	GCCCAGGTGGCGGAATTGGTCGACGC
t0043937	22	16	GGGTATGTAGCTCAGATGGTAG
t0043939	20	16	GCGGGTATAGTTTAGTGGTA
t0043950	21	16	GTTGGTTAGGATACTCGGCTC
t0043969	25	16	GCACTCGTAGCTTAACGGATAGAGC
t0044103	28	16	TGGGCTGTAGCTCAGTTGGGAGAGCGCT

t0044110	18	16	GGATGTAGCTCAGATGGT
t0044136	21	16	GTCCGAAAGGGCGTGGGTTCA
t0044212	23	16	GGGGATGGAGCTCAGATGGTAGA
t0044217	28	16	CTCAGTTGGTAGAGCTGAGGACTGTAGA
t0044221	27	16	GGGTGTAGTTTAATGGTAGAACATCAG
t0044377	22	16	AGGGATATAACTCAGCGGTAGA
t0044385	28	16	GCCGGTGTAGCTCAGTTGGTAGAGCAGC
t0044412	20	16	CGAAAGGGCGTGGGTTCAGA
t0044415	28	16	GCGGGGTAGAGGAATTGGTCAACTCATC
t0044477	27	16	GCGGGTGTAGCTCAATGGTAGAGCAGC
t0044481	27	16	GGCGCGGTAGCAAAGCGGTTATGCACC
t0044518	22	16	GGGCGTTTGGTCTAGTGGTATG
t0044527	28	16	GCGCATCTGGTGTAGTGGTATCATAGTA
t0044598	22	16	CCGGAGCGGGAGATTGTGGGTT
t0044647	24	16	GACTTCTAATCAGGCGATTGTGGG
t0044730	27	16	GGTGGGTGTAGCTCAGCTGGCAGAGCA
t0044799	21	16	TTGCGAGAGGTACGGGGATCG
t0044806	20	16	CGCGGGGTAGAGCAGTTTGG
t0044815	20	16	CGGGGTAGAGGAATTGGTCA
t0044871	24	16	GGAGGTATGGCTGAGTGGCTTAAG
t0044878	22	16	GCGGGTGTAGTTCAATGGCAGA
t0044925	22	16	TTTGAAATCTGTTGGGCTTCGC
t0044987	24	16	GGCGCGGTAGCAAAGCGGTTATGC
t0045048	24	16	GTGGTCGTGCCGGAGTGGTTATCG
t0045179	21	16	GGTGCTGTGGTGTAGTGGTTA
t0045193	29	16	TCCGGTATGGTGTAGTGGCTAACATCGCT
t0045266	26	16	GGGGTTGTAGCTCAAATGGTAGAGCT
t0045272	28	16	GGCGGATGTGGCCAAGTGGATCAAGGCA
t0045307	27	16	GCCCGGATGGCGGAATTGGTAGACGCG
t0045339	28	16	GGTCTGTAGCTCAGTCGGTTAGAGCACC
t0045342	20	16	CGCGTTTGTAGTCCAACGGT
t0045368	28	16	TGGTAGAGCATTTGACTGCACATCAAGA
t0045394	23	16	GGGGATGTAGCTCAAAGGTAGAG
t0045448	21	16	CAGTTGGTAGAGCGGAGGACT
t0045457	25	16	TGTGTCGTTGTGTAGTTGGTTATCA
t0045504	21	16	AGTCCCGTAGCTCAGTTGGTT
t0045540	20	16	GGCGTCGTGGTGTAGTTGGT
t0045576	21	16	GTTAGGATACTCGGCTCTCAC
t0045580	27	16	GGTCTGGTTGTCTAGCGGTATGATTCT
t0045604	30	16	TCCATTGTCGTCTAGTCCGGTTAGGATACC
t0045618	27	16	GGCAGAGCGGAGGACTGTAGATCCTTA
t0045651	26	16	GGGGCCGTAGCTCAGCTGGGAGAGCG
t0045664	22	16	GGGGATGTAGCTCAGATGGCAG
t0045711	20	16	GGGGATGTAGCTCAAGCGGT
t0045816	24	16	GGGGCTGTAGCTCAGCTGGGAGAG
t0045940	24	16	GGGCGTTTGGTCTAGTGGTATGAT
t0045991	28	16	AGTGGTAGAGCATTTGACTGCATATCAA
t0046070	28	15	GGGCTGTAGCTCAGCTGGGAGAGCACCT
t0046095	31	15	GGCCTCGTGGCGGAGTGGTGACGCAGAGGAC
t0046146	26	15	GCGGAAATAGCTTAATGGTAGAGCAT

t0046174	25	15	GGGGACGTAGCTCAGCTGGGAGAGC
t0046184	29	15	TTGGTAGAGCGGAGGACTGTAGATCCTTA
t0046188	18	15	CGAAAGGGCGTGGGTTCA
t0046240	23	15	TCCGGTATGGTGTAGTGGCTAAC
t0046250	26	15	TATGGTGTAGTGGTTAGCACTCTGGA
t0046349	29	15	GGGGGATTAGCTCAGCTGGGAGAGCACCT
t0046394	22	15	GGGTTGTAGCTCAAATGGTAGA
t0046427	25	15	GCGTTTGTAGTCCAACGGTCAGGAT
t0046461	22	15	GGGGGTGTAGCTCATATGGTAT
t0046472	20	15	AATTCCCGTCGTTCGCCCCA
t0046489	24	15	GGGTGTATAGCTCAGTTGGTAGAG
t0046497	29	15	GCGGACGTAGCGCAGCTGGTAGCGCATCA
t0046563	26	15	GGGCCTGTAGCTCAGTTGGTTAGAGC
t0046634	20	15	GGAAATGTAGCTCAGATGGT
t0046639	26	15	TCCGTAGCTCAATTGGCAGAGCAGCC
t0046667	23	15	AGGGATGTAGCTCAGATGGTAGA
t0046687	29	15	GGATGGATGTCTGAGCGGTTGAAAGAGTC
t0046712	21	15	GGGGGTGTAGCTCATGGTAGA
t0046750	24	15	TTGGTAGAGCTGAGGACTGTAGAT
t0046754	27	15	TTGGTAGAGCGGAGGACTGTAGATCCT
t0046774	27	15	ACGGGAATAGTTCAACGGTAGAATAGA
t0046822	19	15	TCTCGGTAGCTCAGTTGGT
t0046849	26	15	GCAGTGGATTAGCGCTTTTGACTTCG
t0046903	20	15	GCGAGAGGTACGGGGATCGT
t0046905	28	15	GGTCTGGTGGTCTAGTGGTATGATTCTC
t0046981	20	15	GGGGATGTAGCTCAGGGTAG
t0047036	20	15	GCGCGGATGGCGGAATTGGT
t0047038	22	15	CTAGGTGGTTAGGATACTCGGC
t0047045	32	15	TCCCCTGTAGCTCAATTGGCAGAGCAGCCGGC
t0047073	24	15	AGGCACGTAGCTCAGTGGGAGAGC
t0047119	28	15	ACGGGAATAGTTCAACGGTAGAATAGAG
t0047129	28	15	AGGACATTGGACTCTGAATCCAGTAACC
t0047156	28	15	CAGAAGGTTGCGTGTTCGTTTCACGTCG
t0047159	28	15	GGTGGGTGTAGCTCAGCTGGTAGAGCAC
t0047188	22	15	ACGGACTGTAAATTCGTTGACG
t0047200	21	15	GTAGAGCGGAGGACTGTAGAT
t0047228	25	15	CGGGACGTGGCGCAGCTTGGTAGCG
t0047293	25	15	ATGATTGGAAATCATGTGGGCTTTG
t0047329	21	15	GGGGATGTAGCTCAGATGGGG
t0047333	28	15	AGATTTAGGCTCTGGTCCGAAAGGGCGT
t0047350	23	15	GGGGACGTAGCTCAGCTGGGAGA
t0047373	26	15	CTCAGTTGGTAGAGCTGAGGACTGTA
t0047414	27	15	GGGCGGTTAGCTCAGGGGTAGAGCACG
t0047458	21	15	AGCTGGTTAGGATACTCGGCT
t0047468	30	15	GGGTCTGTAGCTCAGCTGGTTAGAGCACTG
t0047485	28	15	ACGGGCATAGTTCAACGGTAGAATAGAG
t0047566	22	15	GGGGATGTAGCTCATGGTAGAG
t0047587	21	15	TCAAGCAAGGACTCTACCACG
t0047602	24	15	GCCCCAATAGCTCAGTCGGCAGAG
t0047619	21	15	CAGGTGGCGGAATTGGTAGAC

t0047683	19	15	TCGAGAGAGGGCGTGGGTT
t0047760	19	15	CCGACCTTAGCTCAGTTGG
t0047811	20	15	GCCCCTATAGCTCAGTGGTA
t0047854	24	15	GATTTGAAATCTGTTGGGCTTCGC
t0047858	23	15	GGATGGATGTCTGAGCGGTTGAA
t0047932	20	15	TGGGGTGTAGCTCATATGGT
t0048030	25	15	TGGTAGAGCATTTGACTGCACATCA
t0048111	28	15	GGTTCTATGGTCTAGCGGTTAGGACATT
t0048133	21	15	AGCGGAAGCGTGGTGGGCCCA
t0048143	23	15	GCGCGGGTGGCGGAATAGGCAGA
t0048157	29	15	GTTGTAGTATAGTGGTAAGTATTCCCGCC
t0048259	26	15	GCGGGTGTAGCTCAGTTGGTAGAGCG
t0048271	25	15	GCAGTGGATTAGCGCTTTTGACTTC
t0048278	28	15	GGATCTGTAGCTCAGTCGGTTAGAGCAC
t0048288	21	15	GGTGTCGTGGTGTAGTTGGGT
t0048294	25	15	GGTAGAGCAGAGGACTGTAGATCCT
t0048370	26	15	GCGGGTGTAGTTTAGTAGTAGAACAA
t0048429	27	15	TCAGTGGTAGAGCATTTGACTGCAGAT
t0048432	28	15	GCGGGCGTAGTTCAATGGTAGAACGGCA
t0048480	20	15	GGTTCGAGTCCGGTCGGGGG
t0048526	27	15	TCTGACTTCTAATCAGGCGATTGTGGG
t0048560	19	15	GGGGATGTAGCTCAATGGT
t0048568	30	15	GGTGGCTGTAGCTCAGTTGGTAGAGTCCCA
t0048575	29	15	GGGGCTGTAGCTCAGCTGGGCGAGCGCTA
t0048579	26	15	GGGTCTGTAGCTCAGTTGGTTAGAGC
t0048594	20	15	GTTGTCGTGGTGTAGTTGGT
t0048643	28	15	GGGGGTGTAGCTCATATGGTAGAGCGCT
t0048690	27	15	ACTTCTAATCAGGCGATTGTGGGTTCG
t0048736	20	15	AGTGGTATGATTCTCGCTTT
t0048747	28	15	GCGGGCGTAGCTCAGGGGTAGAGCACAA
t0048800	25	15	TGTGTCGTCGTGTAGTTGGTTATCA
t0048814	26	15	GGGGATGTAGCTCAAATGGTAGAGCT
t0048874	22	15	TCCATTGTCGTCTAGTCCGGTA
t0048888	28	15	GGTAGAGCGGAGGACTGTAGATCCTTAG
t0048901	27	15	GCGGGTGTAGCTCAGGGGTAGAGCATA
t0048958	25	15	GCGCGGATGGCGGAATTGGTCGACG
t0049008	28	15	GGTGTAGTTTAATGGTAGAACATCAGCT
t0049047	24	15	GCGCCTGTAGCTCAGTGGAAGAGC
t0049086	21	15	CGAAAGGGCGTGGGTTCAAAT
t0049143	26	15	GCGGGTGTAGCTCAGGGGTAGAGCAT
t0049176	27	15	GGGGATGTAGCTCAAACGGTCGAGCGC
t0049191	22	15	GGATGGATGTCTGAGCGGTTGA
t0049227	27	15	AGATTTAGGCTCTGGTCCGAAAGGGCG
t0049241	20	15	CGAATCCTGCTGTCGACGCC
t0049291	25	14	GTGGTAGAGCATTTGACTGCAGATC
t0049325	27	14	GGCTTGGTGGTCTAGTGGTATGATTCT
t0049347	25	14	GGGGATGTAGCTCAGATGGTAGACC
t0049435	21	14	GCGCTCTTAGTTCAGTTCGGT
t0049439	23	14	AGCGGGGTAGAGGAATTGGTCAT
t0049443	23	14	GCGGGTGTAGCTCAGGGGTAGAG

t0049462	28	14	GCGGGTGTAGCTCAGGGGTAGAGCACAA
t0049502	29	14	GGGGGTATAGCTCAGCTGGGAGAGCGCTT
t0049560	20	14	ACACTGAAGGTCTCCGGTTC
t0049597	24	14	GCGGATGTGGCGGAACTGGTAGAC
t0049651	21	14	GGGGATGTAGTTCAGATGGTA
t0049688	30	14	TCCCCTGTAGCTCAATTGGCAGAGCAGCCG
t0049701	27	14	GTGGTAGAGCATTTGACTGCACATCAA
t0049756	27	14	AGTGGTAGAGCATTTGACTGCATATCA
t0049813	26	14	CAGTGGTAGAGCATTTGACTGCAGAT
t0049822	19	14	TGGTAGAGCGGAGGACTGT
t0049892	22	14	GCGCGGGTGGCGGAATAGGCAG
t0049937	24	14	GGTAGAGCGGAGGACTGTAGATCC
t0049947	24	14	GCGGATATGGCGAAATTGGTAGAC
t0049978	27	14	GCGGGTGTAGTTCAATGGTAGAACGCT
t0049980	18	14	GGTTCTATGGTGTAGTGG
t0050208	22	14	CGCGGGGTAGAGCAGTTTGGTA
t0050257	26	14	CTAGTTGGTTAGGATACTCGGCTCTC
t0050289	23	14	GGTTCGATTCCGGTCGGGGGTAC
t0050456	31	14	GGGGCTGTAGCTCAGCTGGGAGAGCGCCGCA
t0050487	20	14	GGGGATTAGCTCAGATGGTA
t0050504	25	14	GCGGGTGTAGTTTAATGGTAGAACA
t0050667	28	14	GGAGTCGTGACCGAGTGGCCGAAGGTGC
t0050680	26	14	GTGGTAGAGCATTTGACTGCACATCA
t0050743	27	14	GCGGGGTAGAGGAATTGGTCCACTCAT
t0050815	20	14	GAGGATGTAGCTCAGATGGT
t0050866	25	14	GGGGCTGTAGCTCAGTTGGGAGAGC
t0050920	30	14	GTCGTTGTAGTATAGTGGTGAGTATTCCCG
t0050950	22	14	GGTTCCATGGTCTAGCGGTTAG
t0051053	21	14	TTCAAGCAAGGACTCTACCAC
t0051075	21	14	GGGGGTGTAGCTCATATGGAA
t0051177	22	14	GGGGATGTAGCTCAATGGTAGA
t0051243	29	14	GCCCCCGTTGTGTAGCGGCCTAGCACGCT
t0051251	32	14	CCGACCTTAGCTCAGTTGGTAGAGCTGAGGAC
t0051313	28	14	TCCCCTGTAGCTCAATTGGCAGAGCAGC
t0051366	21	14	GCGGGGTAGAGGAATTGGTCC
t0051376	20	14	GGGGATGTAGCTCAATGGTA
t0051382	30	14	GGGGTTGTAGCTCAAATGGTAGAGCGCTCG
t0051403	20	14	TTGCGAGAGGTACGGGGATC
t0051408	24	14	GCGGGTGTAGCTCAGTGGTAGAGC
t0051475	23	14	TAGAGCGGAGGACTGTAGATCCT
t0051489	30	14	GCCGATGTAGCTCAGCTGGCTAGAGCAGCT
t0051502	28	14	GGGGCTGTAACTCAGTGGTAGAGTTCTT
t0051505	19	14	GGTAGAGCATTTGACTGCA
t0051550	23	14	GGGGGTGTAGCTCATAGGTAGAG
t0051570	29	14	GGGGCTGTAGCTCAGCTGGGAGAGCGCAA
t0051597	27	14	GCGGACGTAGCGCAGCTGGTCGCGCAT
t0051607	21	14	ACACTGAAGGTCTCCGGTTCG
t0051615	29	14	AGGACATTGGACTCTGAATCCAGTAACCC
t0051655	24	14	CGGGACGTGGCGCAGCTTGGTAGC
t0051664	28	14	CTCGGTTAGTATAGTGGTTAGTATCCCC

t0051705	24	14	GGATGGATGTCTGAGCGGTTGAAA
t0051856	24	14	GCTGGTTTGCCCGAGAGGTTAAGG
t0051867	21	14	CGGGGTGTAGCGCAGCTTGGC
t0051944	29	14	GCGGGTGTAGTTCAATGGTAGAACGCTAG
t0051998	21	14	GTGGTAGAGCATTTGACTGCC
t0052049	30	14	GGGTCTGTAGCTCAGGTGGTTAGAGCGCAC
t0052051	24	14	GGTGTCGTGGTGTAGTTGGTTATT
t0052083	20	14	GGGGATGTAGTTCAGATGGT
t0052112	28	14	GCCCAGATGGCGGAATTGGTCGACGCGC
t0052115	26	14	CGCGGGGTGGAGCAGTTCGGTAGCTC
t0052197	23	14	GCGGACGTGGCGGAATTTGGTAG
t0052292	21	14	AGTGGTAGAGCATTTGACTGC
t0052293	19	14	TTGCGAGAGGTACGGGGAT
t0052330	25	14	AGGGGTGTAGCTCAATTGGCAGAGC
t0052333	27	14	CAGTGGTAGAGCATTTGACTGCAGATC
t0052361	27	14	GGGGATGTAGCTCAGATGGTAGAGCAC
t0052419	23	14	GGGGTTGTAGCTCAAATGGTATA
t0052439	22	14	ACTTCTAATCAGGCGATTGTGG
t0052562	27	14	GGGGATGTAGCTCAAACGGTAGAGCAC
t0052566	30	14	GGGTCTGTAGCTCAGTTGGTCAGAGCGCAC
t0052644	21	14	GGGGATGTAGCTCAGATGGCA
t0052681	27	14	GCCTGGTGGTCTAGTGGTATGATTGTC
t0052689	22	14	GCGCATCTGGTGTAGTGGTATC
t0052724	28	14	GACTTCTAATCAGGCGATTGTGGGTTCG
t0052732	27	14	TCCGGCGTAGCTCAGTCGGTAGAGCGG
t0052747	20	14	GCGGGGATAACTCAGTTGGG
t0052761	23	14	TCCATTGTCGTCTAGTCCGGTCA
t0052770	28	14	GGGGCTGTAGCTCAGCTGGGAGAGCGTT
t0052787	21	14	GGGGATGTATCTCAGATGGTA
t0052832	25	14	GCGGGAGTAGCTCAGTTGGTAGAGC
t0052837	24	13	GGGGGTGTAGCTCAGCTGGGAGAG
t0052907	22	13	GCCCAGGTGGCGGAATTGGTAG
t0052918	24	13	AGGGGTATAGCTCAATTGGCAGAG
t0052946	25	13	GCGGATGTGGCGGAACTGGTAGACG
t0053058	21	13	GGTTAGGATACTCGGCTCTCA
t0053088	28	13	GCAATAGACCCGGGTTCGTCTCCCGGCA
t0053194	27	13	CGCTGGTTCGAATCCGGCAGGTCGGAC
t0053229	26	13	TCCGGAGTAGCTCAGTGGTAGAGCAG
t0053249	24	13	GTTGTCGTGGTGTAGTTGGTTATC
t0053288	24	13	AGTGGTAGAGCATTTGACTGCACA
t0053327	27	13	GGGCGTGTAGCTCAGCGGGAGAGCACT
t0053424	25	13	GGGGATGTAGCTCAGATGGGAGAGC
t0053462	20	13	GTAGAGCGGAGGACTGTAGA
t0053532	22	13	GGGGATGTAGCTCAAAGGTAGA
t0053655	22	13	AGGGATGTAGCTCAGATGGTAG
t0053696	25	13	AGATTGAGGTTCTGGTCCGAAAGGG
t0053705	29	13	GCGGATGTGGCGGAACTGGTAGACGCGCA
t0053746	23	13	GCGGGTGTAGTTTAGTAGTAGAA
t0053763	30	13	AGGTCTGTAGTTCGTTCCTGCATGGGGGCA
t0053797	20	13	GTCAGGATGGCCGAGTGGTC

t0053889	23	13	GGGATTGTAGCTCAAATGGTAGA
t0053899	27	13	GCGTTTGTAGTCCAACGGTTCGGATAA
t0053964	19	13	GGTAGAGCGGAGGACTGTA
t0053988	23	13	CGCGGGGTAGAGCAGTTTGGTAG
t0054027	25	13	GGAGGTATGGCTGAGTGGCTTAAGG
t0054110	30	13	GGTGGTTGTAGCTCAGCTGGTTAGAGCATC
t0054168	23	13	AGTGTCGTGGTGTAGTTGGTTAT
t0054171	20	13	CGGAATAGGCAGACGCGCTG
t0054191	22	13	CAGGTGGCGGAATTGGTAGACG
t0054204	20	13	GGAGATGTAGCTCAAATGGT
t0054247	23	13	GCACCAGTTGTCTAGTGGTAGAA
t0054263	27	13	GTGGTAGAGCATTTGACTGCATATCAA
t0054347	26	13	GAGCGGAGGACTGTAGATCCTTAGGT
t0054441	22	13	GGTGTCGTGGTGTAGCTGGTTA
t0054552	24	13	TGGACTCTGAATCCAGTAACCCGA
t0054554	31	13	GGGGTTGTAGCTCAAATGGTAGAGCGCTCGC
t0054576	29	13	CGGGGTGTAGCGCAGCTTGGCAGCGCGCT
t0054614	20	13	TGTCGTGGTGTAGTTGGTTA
t0054681	26	13	GTAGAGCAGAGGACTGTAGATCCTTA
t0054714	28	13	CCCCCGTTGTGTAGCGGCCTAGCACGCC
t0054768	19	13	GGTCCCATGGTCTAGTGGT
t0054809	22	13	GCGGTCGTGGTGGAATTGGTAG
t0054937	25	13	GGTCCGGTGGTGTAGTTGGTTATCA
t0054947	30	13	AGGGGTATAGCTCAATTGGCAGAGCGTCGG
t0054979	28	13	GGCGGAGTAGCTCAGCTGGTTAGAGCGG
t0055106	20	13	CCGGAGCGGGAGATTGTGGG
t0055115	20	13	GGGGTGGTGGCGCAGTTGGC
t0055168	21	13	CGGAGCGGGAGATTGTGGGTT
t0055211	23	13	GGGGATGTAGTTCAGATGGTAGA
t0055343	24	13	TGGTAGAGCATTTGACTGCACATC
t0055349	27	13	GGGTCTGTAGCTCAGTTGGTTAGAGCA
t0055357	26	13	CGGAAGCGTGGTGGGCCCATAACCCA
t0055420	21	13	AGGCACGTAGCTCAGTGGGAG
t0055444	30	13	TCCGTAGCTCAATTGGCAGAGCAGCCGGCT
t0055462	20	13	GGGGGTGTAGCTCATATGGA
t0055471	22	13	GTCTGGGTGGTGTAGTTGGTTC
t0055474	28	13	CCCGGATGGTGGAATGCAGACACGGCGA
t0055492	19	13	CAGTTGGTAGAGCAAGGCT
t0055496	21	13	GGGAGAGTGGCCGAGTGGTTA
t0055525	29	13	GTCGTTGTAGTATAGTGGTACGTATTCCC
t0055539	23	13	GGGGATGTAGCTCAAACGGTAGC
t0055578	29	13	GGCGCGGTAGCAAAGCGGTTATGCCCCGG
t0055583	30	13	GGGGCTGTAGCTCAGTTGGGAGAGCGCTAG
t0055610	29	13	GCACCAGTTGTCTAGTGGTAGAATAGTAC
t0055656	24	13	GGGGGGGTAGCTCATATGGTAGAG
t0055690	27	13	GTGGTCGTGCCGGAGTGGTTATCGGGC
t0055726	28	13	GGGCGTGTAGCTCAGCGGGAGAGCACTA
t0055740	21	13	GCGGGTGGCGGAATAGGCAGA
t0055842	23	13	GGGTATGTAGCTCAGATGGTAGA
t0055870	23	13	GGGCCTGTAGCTCAGTTGGGAGA

t0055898	19	13	GGCGGATGTAGCCAAGTGG
t0055936	22	13	TCGTGCCCCACGGTGGGCGCCA
t0055971	25	13	GTAGAGCAGAGGACTGTAGATCCTT
t0055979	28	13	GGGTCTGTAGCTCAGCTGGTTAGAGCAC
t0055985	24	13	GCTCAGTCGGCAGAGCGTCTCCAT
t0055998	22	13	GGTGTCGTGGGGTAGTTGGTTA
t0056016	27	13	AGTGGTAGAGCATTTGACTGCACATCA
t0056022	25	13	GTGGATGTAGCTCAGATGGTAGAGC
t0056023	21	13	GGGGATGTAGCTCATGGTAGA
t0056038	26	13	GTGGTAGAGCATTTGACTGCATATCA
t0056050	21	13	GCAGAAAGTCGTAGGTTCGGC
t0056075	27	13	GCGGGTGTAGTTCAGTAGCAGAACATC
t0056207	19	13	ATCAGGCGATTGTGGGTTC
t0056234	28	13	AGCGGGGTAGAGGAATTGGTCCACTCAT
t0056241	23	13	AGTGGTAGAGCATTTGACTGCAT
t0056302	29	13	GCGGGTGTAGTTCAATGGCAGAACGGCAG
t0056308	24	13	GTCTGGGTAGTGTAGTTGGTTATC
t0056328	25	13	GGGCATTTGGTCTAGTGGTATGATT
t0056329	29	13	GCGCGGATGGCGGAATTGGTAGACGCGCT
t0056348	22	13	TCCGGTGGTGTAGTTGGTTATC
t0056423	20	13	GGGGATGTAGCTCAGGTGGT
t0056443	22	13	TTCGGTAGCTCGTCGGGCTCAT
t0056450	25	13	GCGGGCGTAGCTCAATGGTAGAGCC
t0056479	30	13	GCGGTCGTGGCGGAATTGGTAGACGCGCAG
t0056489	25	13	GCGCCTGTAGCTCAGTGGATCGAGC
t0056561	22	13	ACTGATAATGTAGGGGTCGGCA
t0056621	21	13	GGCGGATGTGGCCAAGTGGAA
t0056821	25	13	GGCAAGATGGCGGAGTGGTTAACGC
t0056844	21	13	GGGCGTTTGGTCTAGTGGTAT
t0056867	27	13	TCAGTTGGTAGAGCTGAGGACTGTAGA
t0056920	27	13	GCGGGTGTAGCTCAGGGGTAGAGCACG
t0056974	23	13	GCCCAGGTGGCGGAATTGGTAGA
t0057123	23	12	GGGGCTGTAGCTCAGTTGGGAGA
t0057158	20	12	GGGCGTTTGGTCTAGTGGTA
t0057257	28	12	GCCCCTATCGTCTAGTGGTCAGGACATC
t0057279	28	12	GTTGTAGTATAGTGGTAAGTATTCCCGC
t0057346	21	12	GCGCGGGTGGCGGAATAGGCA
t0057349	18	12	GGAGGTATGGCTGAGTGG
t0057438	22	12	GATTTGAAATCTGTTGGGCTTC
t0057499	24	12	GTTAGGATACTCGGCTCTCACCCG
t0057524	23	12	GCGGGAGTAGCTCAGTTGGTAGA
t0057555	25	12	GGTGTCGTGGTGTAGTTGGTTATCT
t0057649	29	12	GGGGCTGTAGCTCAGCTGGGAGAGCGCTG
t0057750	22	12	GGGGATGTAGCTCAGATAGTAG
t0057836	20	12	CGAGAGGTACGGGGATCGTT
t0057931	23	12	GCGGATGGCGGAATTGGTAGACG
t0057933	25	12	GACATTGGACTCTGAATCCAGTAAC
t0057986	21	12	GGCGGAATTGGTAGACGCGCT
t0057987	27	12	ATTGGACTCTGAATCCAGTAACCCGAG
t0058033	26	12	GCGGGCGTAGCTCAATGGTAGAGCCC

t0058079	20	12	GGGGATATAGCTCAGATGGT
t0058129	27	12	GCCGAGGTGGCGGAATTGGTAGACGCA
t0058141	29	12	AGGCGCGTAGCTCAGTTGGTTAGAGCACC
t0058146	21	12	GGGGGTGTAGCTCAGATGGTA
t0058148	28	12	GGCTCAGTGGTCTAGTGGTATGATTCTT
t0058164	21	12	GTCGTTGTAGTATAGTGGTGC
t0058204	29	12	GACCCTATCGTCTAGAGGCCTAGGACATC
t0058210	30	12	CGGGGTGTAGCGCAGCCTGGTAGCGCACTT
t0058270	22	12	TGGTAGAGCATTTGACTGCACA
t0058312	27	12	GGGGCTGTAACTCAGTGGTAGAGTGCT
t0058316	20	12	TCTAGCGGTTAGGACATTGG
t0058359	24	12	ACATTGGACTCTGAATCCAGTAAC
t0058387	26	12	GCGGGCGTCGTTCAATGGCAGGACCT
t0058436	28	12	GCGGGTGACCCGGGTTCGATCCCCGGCA
t0058452	24	12	AGGGATGTAGCTCAGATGGTAGAG
t0058488	20	12	GCGGCTGTAGCTCAGTTGGA
t0058496	19	12	GTCGTGGTGTAGTTGGTTA
t0058564	28	12	GATTTAGGCTCTGGTCCGAAAGGGCGTG
t0058585	22	12	TCGAGAGAGGGCGTGGGTTCAT
t0058588	27	12	TCTAGTTGGTTAGGATACTCGGCTCTC
t0058684	21	12	GCGGTCGTGGCGGAATTGGTA
t0058689	22	12	GATTGTGATTCTGGTCGTCGTG
t0058708	23	12	CGGGATGTAGCTCAAACGGTAGA
t0058720	21	12	GCTGGAATAGCTCAGTTGGTT
t0058728	19	12	TGTCGTGGTGTAGTTGGTT
t0058808	28	12	GCGGTCGTGGCGGAATTGGTAGACGCGC
t0058886	26	12	AGTTGGTAGAGCTGAGGACTGTAGAT
t0058893	27	12	CAGAAGGTTGCGTGTTCGTTTCACGTC
t0058904	21	12	AAGCGTTTGTAGTCCAACGGT
t0058948	26	12	TGTGGCTGTAGTTTAGTGGTTAGAAT
t0058973	20	12	GCAGAAAGTCGTAGGTTCGT
t0059088	24	12	GGGGATGGAGCTCAGATGGTAGAG
t0059092	24	12	CGGGATGTAGCTCAAACGGTAGAG
t0059100	22	12	CAGTGGTAGAGCATTTGACTGC
t0059151	27	12	GGGACGTTAGCTCAGTCGGTAGAGCAG
t0059164	23	12	AGGATACTCGGCTCTCACCCGAG
t0059180	22	12	GCGGGTGGCGGAATAGGCAGAC
t0059204	30	12	GGAGCTGTAGCTCAGCTGGGAGAGCACCTG
t0059243	21	12	AGTGGTATGATTCTCGCTTTG
t0059247	23	12	GGTAGAGCGGAGGACTGTAGATC
t0059286	25	12	TGTGGCTGTAGTTTAGTGGTTAGAA
t0059289	29	12	GCGGGCGTGGCGGAACTGGTAGACGTGCT
t0059333	23	12	TTGGTAGAGCTGAGGACTGTAGA
t0059374	27	12	GGGGCTGTAGCTCAGTTGGGCGAGCGC
t0059441	19	12	GTTGGTAGAGCGGAGGACT
t0059490	22	12	AGCGGGGTGTAGGAATTGGTCA
t0059574	18	12	GCGCCTGTAGCTCAGTGG
t0059587	26	12	GCGGACGTGGTGGAATTGGTAGACAC
t0059610	27	12	TGGGTTCGAGCCCCACGGTGGGCGCCA
t0059612	29	12	AGGGGCATAGCTCAATTGGCAGAGCGTCG

t0059662	26	12	GCGGGTGGCGGAATAGGCAGACGCGC
t0059664	29	12	GCCCGGATGGTGGAATGCAGCCACGGCGA
t0059712	21	12	GGGTCTGTAGCTCAGTCGGTT
t0059745	19	12	GTGGTTAGGATACTCGGCT
t0059793	25	12	GTTGTCGTGGTGTAGTTGGTTATCA
t0059797	27	12	GGCGGATGTGGCCAAGTGGATCAAGGC
t0059873	28	12	TCAGTGGTAGAGCATTTGACTGCAGATC
t0059960	19	12	AGACTGTAAATCTGCCGGC
t0060003	21	12	GGATGGATGTCTGAGCGGTTG
t0060008	22	12	CGGATGGCGGAATTGGTAGACG
t0060012	28	12	AGGCACGTAGCTCAGTGGGAGAGCACTA
t0060040	27	12	GCAGTGGATTAGCGCTTTTGACTTCGG
t0060102	27	12	GGCGGAGTAGCTCAGTAGGTCAGAGCA
t0060109	20	12	GCTGGTTAGGATACTCGGCT
t0060159	29	12	GGGCCTGTAGCTCAGTCGGTTAGAGCACC
t0060184	30	12	ATTGGACTCTGAATCCAGTAACCCGAGTTC
t0060193	21	12	TCCAAGCAATAGATCCGGGTT
t0060201	29	12	GGTCTGTAGTTCGGTCCTGCATGGGGGCA
t0060235	25	12	GGAGTTGTAGCTCAAATGGTAGAGC
t0060386	25	12	TGGTAGAATAGTACCCTGCCACGGT
t0060419	27	12	GTAGAGCGGAGGACTGTAGATCCTTAG
t0060469	22	12	TTGAAATCTGTTGGGCTTCGCC
t0060477	23	12	GGGGATTTAGCTCAGATGGTAGA
t0060532	21	12	GTGTCGTGGTGTAGTTGGTTC
t0060573	25	12	TGGTTAGGATACTCGGCTCTCACCC
t0060580	20	12	GGGGATGTAGCTCATGGTAG
t0060584	23	12	GCGGGTGTAGTTCAGTAGCAGAA
t0060638	20	12	GGCGCGGTAGCAAAGCGGTT
t0060655	29	12	CGGGGTGTAGCGCAGCCTGGTAGCGCACT
t0060684	19	12	GCGGATGTGGCCAAGTGGA
t0060747	25	12	GGGGGTGTAGCTCATATGGTAGATC
t0060754	28	12	CGGGTGGCGGAATAGGCAGACGCGCTGG
t0060810	24	12	GGGGATGTAGCTCAGATGGGAGAG
t0060852	23	12	TGCGAGAGGTACGGGGATCGTTA
t0060947	24	12	GCGCCTGTAGCTCAGTGGGAGAGC
t0060962	24	12	GAGCGGAGGACTGTAGATCCTTAG
t0060965	22	12	GTCTGGGGGGTGTAGTTGGTTA
t0061056	26	12	GCCGGAGTGGTGGAACTGGCAGACAC
t0061057	27	12	GGGGCTGTAGCTCAGCTGGGCGAGCGC
t0061089	21	12	GTCGATATGTCCGAGTGGTTC
t0061111	25	12	GCGGGGATAGCTCAGTTGGGCGAGC
t0061152	30	12	GGGGATGTAGCTCAGATGGTCGAGCGCTCG
t0061168	27	12	GCGGCTGTAGCTCAGTGGATAGAGTAT
t0061234	25	12	TTGGTAGAGCTGAGGACTGTAGATC
t0061305	23	12	GTGGTAGAGCATTTGACTGCACA
t0061344	27	12	GCCGGAGTGGCGAAATCGGTAGACGCA
t0061421	26	12	CCCCGTTGTGTAGCGGCCTAGCACGC
t0061438	28	12	CAGTGGTAGAGCATTTGACTGCAGATCA
t0061467	23	12	CGGATGGTGGAATGCAGACACGG
t0061498	22	12	ATTTGAAATCTGTTGGGCTTCG

t0061598	26	12	TGGACTCTGAATCCAGTAACCCGAGT
t0061794	29	12	GCCCAGGTGGCGGAATTGGTAGACGCGCT
t0061803	30	12	GGGGATGTAGCTCAAATGGTAGAGCGCTCG
t0061865	26	11	GCGTTTGTAGTCCAACGGTTCGGATA
t0061905	27	11	ACGGGCGTAGTTCAAGGGTAGAATAGC
t0061923	21	11	GAGGATGTAGCTCAGATGGTA
t0061941	27	11	GGTGTCGTGGTGTAGTTGGTTATCACA
t0061942	22	11	GGGGATGTAGCTCAGATGGTCA
t0061989	20	11	GGTAGAGCATTTGACTGCAT
t0062063	27	11	GGCATTTGGTCTAGTGGTATGATTCTC
t0062064	21	11	AGCAGAAAGTCGTAGGTTCGT
t0062077	18	11	GGGGATGTAGCTCAGGGT
t0062108	27	11	GGTGGGTGTAGCTCAGCTGGTAGAGCA
t0062137	28	11	GTTGGTAGAGCGGAGGACTGTAGATCCT
t0062167	28	11	GCCTTGATGGTGAAATGGTAGACACGCG
t0062174	29	11	GGAGGTATGGCTGAGTGGCTTCAGGCATT
t0062194	25	11	GCGGGTGTAGTTCAATGGTAGAACG
t0062196	25	11	GCGAGGATGGCGGAATTGGTAGACG
t0062203	24	11	ATGATTGGAAATCATGTGGGCTTT
t0062294	28	11	CGGGGTGTAGCGCAGCCTGGTAGCGCAC
t0062371	22	11	GGGAGTGTAGCTCATATGGTAG
t0062413	20	11	GGGGATGTATCTCAGATGGT
t0062450	24	11	CAGTGGTAGAGCATTTGACTGCAT
t0062452	21	11	GGTTCGATTCCGGTCGGGGGT
t0062499	26	11	CTCAGTGGTAGAGCATTTGACTGCAG
t0062522	22	11	GGGGATGTAGCTCTGATGGTAG
t0062569	23	11	GGGGATGTAGCTCAGATGTAGAG
t0062591	28	11	GGGGTTGTAGCTCAAATGGTAGAGCGCT
t0062593	28	11	CTCAGTGGTAGAGCATTTGACTGCAGAT
t0062766	25	11	GCGGACGTAGCGCAGCTGGTAGCGC
t0062767	29	11	GGGATATAACTCAGCGGTAGAGTGTCACC
t0062769	23	11	GGTTCCATGGTCTAGCGGTTAGG
t0062800	20	11	TGGATCAAGGCAGTGGATTG
t0062882	19	11	TCGTTTGTAGTCCAACGGT
t0062930	29	11	GGGGCTGTAGCTCAGCTGGGAGAGCGCGG
t0062962	25	11	GCGCGGGTGGCGGAATAGGCAGACG
t0063037	23	11	GGTTCGAGTCCGGTCGGGGGTAC
t0063188	22	11	GGAGTGGTTATCGGGCATGATT
t0063260	24	11	GACACACTGAAGGTCTCCGGTTCG
t0063261	28	11	GGTGGCTGTAGCTCAGTTGGCAGAGCAC
t0063283	21	11	GGAGAGATGGCCGAGTGGTTG
t0063291	27	11	GCCCGGGTGGCGGAATTGGTAGACGCA
t0063340	25	11	GGAGAAATGGCAGAGCGGTCGAATG
t0063380	23	11	AGGCACGTAGCTCAGTGGGAGAG
t0063412	21	11	AAGCGGGAGGTCTTGAGTTCA
t0063430	29	11	AGGGGTATAGCTCAATTGGCAGAGCGTCG
t0063472	23	11	GTCAGGATGGCCGAGTGGTCTAA
t0063559	27	11	GCCCCTGTGGCGGAACTGGTAGACGCG
t0063624	27	11	GCGGGAGTAACTCAGTGGTAGAGTGCA
t0063651	22	11	AGCGGAAGCGTGGTGGGCCCAT

t0063704	23	11	AGAGCGGAGGACTGTAGATCCTT
t0063732	22	11	GGTTCGTTTCCCGGCTGGTGCA
t0063738	23	11	GTAGAGCGGAGGACTGTAGATCC
t0063760	20	11	GGGCTTATAGTTTAATTGGT
t0063773	21	11	GGATTGTAGTTCAATCGGTCA
t0063856	27	11	GGGGTGTAGCTTAGCCTGGTAGAGCGC
t0063860	27	11	GCTCAGTGGTAGAGCATTTGACTGCAG
t0063879	20	11	AGTGGCTGTAGTTTAGTGGT
t0063918	20	11	AAGCAGAAGGCCGTAGGTTC
t0063927	20	11	GTTGGCTGTAGTTTAGTGGT
t0064009	28	11	CCAGTGGTCTAGTGGTAGAATAGTACCC
t0064208	27	11	ACCAGTGGTCTAGTGGTAGAATAGTAC
t0064241	30	11	AGGAGTGTAGCTCAATTGGTAGAGCACCGG
t0064252	23	11	AGTTGGTTAGGATACTCGGCTCT
t0064257	25	11	CTCAGTTGGTAGAGCGAGGACTGTA
t0064284	20	11	TGCGAGAGGTACGGGGATCG
t0064344	22	11	TTCCAAGCAATAGATCCGGGTT
t0064368	21	11	TCAGTTGGTAGAGCTCAAGGC
t0064397	30	11	GGGGATGTAGCTCAAACGGTAGAGCACTCG
t0064403	23	11	GGGGATGTAGCTCTGATGGTAGA
t0064418	30	11	GCCCAGGTGGCGGAATTGGTAGACGCACTA
t0064442	25	11	GGGGCTGTAGCTCAGCTGGGCGAGC
t0064524	19	11	GCGGATATGGTCGAATGGT
t0064578	31	11	GCGGGTGGCGGAATAGGCAGACGCGCTGGAT
t0064579	31	11	GGGGCTGTAGCTCAGTTGGGAGAGCGCCTGA
t0064603	24	11	GTGGCTGTAGTTTAGTGGTAAGAA
t0064673	24	11	GCGGGTGTAGTTCAGTAGTAGAAC
t0064714	22	11	CGGAGCGGGAGATTGTGGGTTC
t0064793	28	11	TCCGGCGTAGCTCAGTCGGTAGAGCGGG
t0064840	28	11	CCGGATGGTGGAATGCAGACACGGCGAG
t0064862	23	11	TAGAATAGTACCCTGCCACGGTA
t0064919	26	11	GCCCAGATGGCGGAATTGGTCGACGC
t0065054	26	11	AGGACATTGGACTCTGAATCCAGTAA
t0065126	28	11	GGGTCTGTAGCTCAGGTGGTTAGAGCGC
t0065217	29	11	GGGGCTGTAGCTCAACTGGGAGAGCGCCA
t0065238	20	11	ATCAGAGTGGCGCAGCGGAA
t0065252	21	11	GCGGATATGGTCGAATGGTAC
t0065275	22	11	GTTAGGATACTCGGCTCTCACC
t0065329	21	11	TCGAACCCTGCTGTCGACGCC
t0065402	28	11	TGGTAGAGCATTTGACTGCATATCAAGA
t0065415	25	11	GGGGGTGTAGCTCAGCTGGGAGAGC
t0065440	27	11	CGGGGTGTAGCTCAGCCTGGTAGAGCA
t0065473	24	11	GGGGATGTAGCTCAGTTGGTAGAG
t0065516	23	11	GACCGCATAGCGCAGTGGATTAG
t0065527	23	11	GGGGTTGTAGCTCAAAGGTAGAG
t0065571	24	11	GGAGTCGTGACCGAGTGGCCGAAG
t0065575	23	11	GCGGGGATAACTCAGTTGGGAGA
t0065596	28	11	GGTGTCGTGGTGTAGTTGGTTATCACGT
t0065611	20	11	GGCGGAATAGGCAGACGCGC
t0065614	27	11	GGGCCTGTAGCTCAGTTGGTTAGAGCA

t0065627	29	11	GCGAACGTAGCTCAGTTGGTAGAGCGCAA
t0065639	28	11	GCCCTCATCGTCTAGCGGCCTAGGACGC
t0065700	27	11	GCCGGTGTAGCTCAGGGGTAGAGTGCT
t0065723	28	11	GCGGGTGTAGCTCAGGGGTAGAGCACGA
t0065817	21	11	TAGGATACTCGGCTCTCACCC
t0065835	19	11	CCGAAAGGGCGTGGGTTCA
t0065865	22	11	GGTTAGGATACTCGGCTCTCAC
t0065956	30	11	GTTGGTAGAGCTGAGGACTGTAGATCCTTA
t0065972	28	11	GGGGCTATAGCTCAGTTGGTTAGAGCGC
t0065973	18	11	AGCGGGAGATTGTGGGTT
t0066025	28	11	GGGTCTGTAGCTCAGTCGGTTCGAGCAC
t0066031	28	11	GCCGGAGCGGTGGAATTGGCAGACACGC
t0066037	25	11	TCCCTGGTAGCTCAGCGGTAGAGCA
t0066045	27	11	AGCGGAAGCGTGGTGGGCCCATAACCC
t0066151	28	11	GGCTTTTTGGTCTAGCGGTATGATTCTC
t0066182	26	11	GGGGGTGTAGCTCATATGGTAGAGAA
t0066219	23	11	TGGTAGAGCATTTGACTGCATCA
t0066280	20	11	AGAGCGGAGGACTGTAGATC
t0066316	28	11	GGGGCTGTAGCTCAGTTGGTAGAGCGGT
t0066330	28	11	GGGGCGGTAGCTCAGCTGGGAGAGCGTC
t0066350	23	11	GGGGATGTAGCTCAGATGGTGGA
t0066421	23	11	CTCGTGAGAGGGCGTGGGTTCAT
t0066436	19	11	TCTAGCGGTTAGGACATTG
t0066483	20	11	GGGGATGTAGCTCAGAGGGG
t0066492	22	11	GCGCTCTTAGTTCAGTTCGGTA
t0066560	20	11	TAGGTGGTTAGGATACTCGG
t0066587	26	11	GCGGACGTGGCGGAATTGGTAGACGC
t0066604	18	11	GGGGGTGTAGCTCATATG
t0066628	26	11	CGGACGTAGCTCAGTTGGTAGAGCGC
t0066646	22	11	GAGGATGTAGCTCAGATGGTAG
t0066695	28	11	TAGAGCTGAGGACTGTAGATCCTTAGGT
t0066709	19	11	GCACCAGTTGTCTAGTGGT
t0066715	25	11	CCAGTTGTCTAGTGGTAGAATAGTA
t0066739	24	11	GGGTCCATAGCTCAGTGGTAGAGC
t0066803	22	11	GGAGAGGTGGATGAGCGGTTTA
t0066974	25	11	GGGGGTGTAGCTCATATGGTAGAAA
t0067091	21	11	TCGTGAGAGGGCGTGGGTTCA
t0067125	28	11	TGGTAGAGCAGAGGACTGTAGATCCTTA
t0067176	29	11	GCGGTCGTGGCGGAATTGGTAGACGCGCT
t0067196	29	11	GCGCCTGTAGCTCAGTGGATAGAGCGTCT
t0067229	25	11	GACACACTGAAGGTCTCCGGTTCGT
t0067232	20	11	GTGATGTAGCTCAGATGGTA
t0067245	23	11	CTCAGTTGGTAGAGCGGAGGACT
t0067309	27	11	GGGGCTGTAACTCAGTGGTAGAGTCCT
t0067336	21	11	GGGGCTATAGCTCAGTCGGTT
t0067339	19	11	GCAGGAGGTCAGCGGTTCG
t0067340	26	11	GCGGAAGCGTGGTGGGCCCATAACCC
t0067391	29	11	GCGGTCGTGGCGGAACTGGTAGACGCGCA
t0067398	21	11	GGGGATATAGCTCAGATGGTA
t0067452	26	11	GGTCTGTAGCTCAGTCGGTTAGAGCA

t0067532	25	11	GCCCTCATCGTCTAGCGGCCTAGGA
t0067570	20	10	TGGGATGTAGCTCAAATGGT
t0067578	22	10	GTCTGGGTGGTGTAGTTGGTTT
t0067784	20	10	TCAGTTGGTAGAGCAAGGCT
t0067838	29	10	GCGTTTGTAGTCCAACGGTTCGGATAATT
t0067843	19	10	GCGGAAATAGCTTAATGGT
t0067884	18	10	TGGTAGAGCGGAGGACTG
t0067922	29	10	GGGGCTGTAGCTCAGCTGGGAGAGCGCCA
t0067932	22	10	CCGACCTTAGCTCAGTTGGCCG
t0067974	18	10	GGGGATGTAGCTCAGGGG
t0067996	21	10	CCGGTGGTGTAGTTGGTTATC
t0068056	30	10	GGCCTCGTGGCGGAGTGGTGACGCAGAGGA
t0068058	27	10	GCCCGGGTGGCGAAATCGGTAGACGCA
t0068098	27	10	GGTAGAGCTGAGGACTGTAGCTCCTTA
t0068205	20	10	AAGCGGGAGGTCTTGAGTTC
t0068259	30	10	AGGACATTGGACTCTGAATCCAGTAACCCG
t0068262	29	10	GGGGATGTAGCTCAAATGGTAGAGCCTCG
t0068270	27	10	GGTCTGTAGCTCAGTCGGTTAGAGCAC
t0068379	27	10	GAGTTGCGCAGCGGAAGCGTGGTGGGC
t0068389	23	10	ACACTGAAGGTCTCCGGTTCGTG
t0068400	23	10	GGGAATGTAGCTCAAATGGTAGA
t0068437	25	10	GGTGTAGTTTAATGGTAGAACATCA
t0068441	18	10	TCAAGGCAGTGGATTGTG
t0068461	25	10	GGGGATGTGGCGGAATGGCAGACGC
t0068499	25	10	GCTCGGGTGGCGGAATTGGTAGACG
t0068512	21	10	GGTGTCGTGGTGTAGTTGGTA
t0068515	20	10	CGGTGTAGCTCAGTCGGTAG
t0068558	22	10	GCCTGATAAGCGTGAGGTCGGA
t0068625	19	10	TTGGTAGAGCGGAGGACTG
t0068626	23	10	TTAGGATACTCGGCTCTCACCCG
t0068633	25	10	GGCGCGGTAGCAAAGCGGTTATGCA
t0068818	24	10	GCGGGAGTAGTTCAATGGTAGAAC
t0068974	28	10	GGGGCTGTAGCTCAGCTGGGCGAGCGCC
t0069052	24	10	GCGTTTGTAGTCCAACGGTCAGGA
t0069180	22	10	GGGGATGTAGCTCAAATGGTAA
t0069234	24	10	GCGGAATAGGCAGACGCGCTGGAT
t0069341	29	10	GGTAGAGCGGAGGACTGTAGATCCTTAGG
t0069392	20	10	ACTTCTAATCAGGCGATTGT
t0069490	24	10	GGTGTAGTTTAATGGTAGAACATC
t0069637	28	10	TCTGACTTCTAATCAGGCGATTGTGGGT
t0069776	21	10	AGCAGAAGGCCGTAGGTTCGA
t0069782	24	10	GGGATTGTAGCTCAAATGGTAGAG
t0069784	22	10	GGTCTCGTGGTGTAGTTGGTTA
t0069789	21	10	CCGAAAGGGCGTGGGTTCATC
t0069796	25	10	GCGGAATAGGCAGACGCGCTGGATT
t0069827	31	10	GAGGGCGTGGGTTCAAATCCCACTTCTGACA
t0069848	21	10	TCCCCTGTAGCTCAATTGGCA
t0069857	20	10	CGTGATGTAGCTCAGATGGT
t0069869	21	10	TGTAGTGGTTAGCACTCTGGA
t0069919	22	10	GCTCAGTCGGTAGAGCAAGCGG

t0069922	24	10 GGGGGTGTAGCTCATATGGTAGAT
t0069927	30	10 GGGATGTAGCTCAGATGGTAGAGCGCTCGC
t0069929	20	10 TGGTAGAGCGGAGGACTGTA
t0069949	25	10 TCCGTTGTAGTCTAGGTGGTTAGGA
t0070029	28	10 GGGCGCGTGGTCTAGTGGTATGATTTTC
t0070168	20	10 GAAAGGGCGTGGGTTCAAAT
t0070305	25	10 GGTGGGTGTAGCTCAGCTGGCAGAG
t0070390	19	10 TCAGTTGGTAGAGCAAGGC
t0070427	24	10 GGGGATGTAGTTCAGATGGTAGAG
t0070431	24	10 GCCCTTCGTCTATCGGTTAGGACA
t0070456	22	10 GGTTCGATTCCGGTCGGGGGTA
t0070653	19	10 GAAAGGGCGTGGGTTCAAA
t0070666	29	10 AGGCGCGTAGCTCAGCTGGTTAGAGCACC
t0070760	18	10 GTGTCGTGGTGTAGTTGG
t0070825	29	10 GGGCGTTTTGTCTAGTGGTATGATTCTCG
t0070845	27	10 TGGTAGAGCGGAGGACTGTAGATCCTT
t0070992	27	10 GCGGGCGTGGCGGAACTGGTAGACGCG
t0071005	21	10 GGGGATGAAGCTCAGATGGTA
t0071027	23	10 TGTGTCGTTGTGTAGTTGGTTAT
t0071044	23	10 CGGGGTGTAGCTCATATGGTAGA
t0071050	31	10 GCGGTCGTGGCGGAATTGGTAGACGCGCAGC
t0071075	29	10 GCGGAAATAGCTTAATGGTAGAGCATAGC
t0071138	19	10 TCAGTTGGTAGAGCGCTGT
t0071166	24	10 CTCCGGAGCGGGAGATTGTGGGTT
t0071217	23	10 GCGGAAGTAGCTCAGTTGGTAGA
t0071269	28	10 GCGGGAGTAACTCAGTGGTAGAGTGCAA
t0071324	23	10 GGGGGTGTAGCTCATATGGTATA
t0071389	22	10 GTGTCGTTGTGTAGTTGGTTAT
t0071418	22	10 GGGGATGTAGCTCAAATGGTAT
t0071436	24	10 AGCGGGGGAGAGGAATTGGTCAAC
t0071447	26	10 GTTGTAGTATAGTGGTGAGTATTCCC
t0071493	21	10 GCGCCTGTAGCTCAGTGGATA
t0071503	28	10 GCACCAGTAGTCTAGTGGTAGAATAGTA
t0071556	29	10 GGGGCCGTAGCTCAGCTGGGAGAGCGTCT
t0071629	21	10 GCGGTGAGCGTGGATCGAACA
t0071706	24	10 GCTCGGATGGTGAAATTGGTAGAC
t0071727	27	10 GGCTCAGTTGTCTAGTGGTATGATTCT
t0071739	20	10 GCGTTTGTAGTCCAACGGGA
t0071856	28	10 GGTCTGGTAGTCTAGCGGTATGATTCTC
t0071942	25	10 GGAGGATGTAGCTCAGTTGGTAGAG
t0071970	21	10 AGGTGGCGGAATTGGTAGACG
t0072034	26	10 TGCCTTCCAAGCAATAGATCCGGGTT
t0072154	22	10 GGGGGTGTAGCTCAGATGGTAG
t0072189	22	10 ACACACTGAAGGTCTCCGGTTC
t0072192	29	10 GGGTCTGTAGCTCAGTCGGTTCGAGCACC
t0072208	21	10 TGGGGCGTGGCCAAGCGGTAA
t0072223	23	10 GGGCGTTTGGTCTAGTGGTATGA
t0072307	22	10 GGGCATTTGGTCTAGTGGTATG
t0072565	22	10 GGGGGTGTAGCTCATGGTAGAG
t0072586	21	10 GTCAAGATGGCCGAGTTGGTC

t0072613	20	10	GGATTGTAGTTCAATCGGTC
t0072625	23	10	ATCGGGGTAGAGGAATTGGTCAA
t0072644	28	10	CCCCTGTAGCTCAATTGGCAGAGCAGCC
t0072729	24	10	GGTAGAGCATTTGACTGCACATCA
t0072742	24	10	GGCGTCGTGGTGTAGTTGGTTATC
t0072825	23	10	GTTGGTAGAGCTGAGGACTGTAG
t0072843	21	10	GCGCCTGTAGCTCAGGGGAGA
t0072893	24	10	GGGGATGTAGCTCAGATGGTACAG
t0072900	28	10	AGAGCTGAGGACTGTAGATCCTTAGGTC
t0072954	25	10	GGGGATGTAGCTCAGATGGTAGAGA
t0073002	29	10	GGCGGATGTAGCCAAGTGGATCAAGGCAG
t0073155	29	10	GGTAGAGCATTTGACTGCAGATCAAGAGG
t0073161	29	10	GCGAGGATGGCGGAATTGGTCGACGCGCT
t0073223	29	10	CAGAAGGTTGCGTGTTCGTTTCACGTCGG
t0073252	23	10	TGTGTCGTCGTGTAGTTGGTTAT
t0073295	23	10	GGGGGGGTAGCTCATATGGTAGA
t0073307	30	10	GGGGCTGTAGCTCAGCTGGGAGAGCGCGGG
t0073309	19	10	TGCGAGAGGTACGGGGATC
t0073419	22	10	GGGGATGTAGCTCAGAGGGTAG
t0073427	23	10	GTCCGAAAGGGCGTGGGTTCAAA
t0073438	25	10	TCAGTGGTAGAGCATTTGACTGCAT
t0073451	21	10	TCCTCAGTAGCTCAGTGGTAG
t0073454	25	10	AGTTGGTAGAGCTGAGGACTGTAGA
t0073480	20	10	GGGGATGTAGCTCAGATGTT
t0073487	21	10	CCGAAAGGGCGTGGGTTCATA
t0073503	22	10	GGGGATGTAGCTCAGATGTTAG
t0073530	26	10	GTCGATATGTCCGAGTGGTTAAGGGA
t0073699	22	10	GGGGATGTATCTCAGATGGTAG
t0073707	25	10	GGAGATGTAGCTCAAATGGTAGAGC
t0073861	30	10	GGCGCGGTAGCAAAGCGGTTATGCCCCGGA
t0073935	29	10	CTCAGTGGTAGAGCATTTGACTGCAGATC
t0073940	29	10	GGTCTGTAGTTCGATCCTGCATGGGGGCA
t0073996	22	10	GGGGATGTAGCTCAGAAGGTAG
t0074005	23	10	GGGGATGTAACTCAGATGGTAGA
t0074014	21	10	ACTGATAATGTAGGGGTCGGC
t0074039	24	10	GGTGCTGTGGTGTAGTGGTTATCA
t0074089	25	10	GGGTCTGTAGCTCAGTTGGTCAGAG
t0074161	19	10	GTTAGGATACTCGGCTCTC
t0074283	27	10	GTCGATATGTCCGAGTGGTTAAGGATA
t0074318	24	10	GCGGGTGTAGTTCAATGGTAGAAC
t0074323	29	10	GGTGGTTGTAGCTCAGTTGGTAGAGTCCA
t0074359	31	10	CGGGGTGTAGCTTAGCCTGGTAGAGCGCTAC
t0074390	28	10	GCCGCTGTAGCTCAGGGGTAGAGCAACG
t0074391	23	10	GACTTCTAATCAGGCGATTGTGG
t0074404	22	10	TTGGCAGAGCTGAGGACTGTAG
