# Supplementary material for: Transfer RNA Derived Small RNAs Targeting Defense Responsive Genes Are Induced during Phytophthora capsici Infection in Black Pepper (Piper nigrum L.)
Source: Front Plant Sci. 2016 Jun 1;7:767. doi: 10.3389/fpls.2016.00767 (PMC4887504; doi:10.3389/fpls.2016.00767)
Supplement: Supplementary file 5 [file DataSheet4.PDF]

Transfer RNA derived small RNAs targeting defence responsive genes are induced during *Phytophthora capsici* infection in black pepper (*Piper nigrum* L.)

Supplementary Data 4: 5'Ala tRNA variants identified from the leaf sRNA datasets (Illumina GA) other plant species

Plant sp	small RNA library	Tissue	5'Ala tRFs	Sequence	Read count
Aabidopsis thaliana	GSM707679	Leaf	5'AlaAGC tRF1 Variants	GGGGATGTAGCTCAGATGGTAGAGC	789
				GGGGATGTAGCTCAGATGGT	685
				GGGGATGTAGCTCAGATGGTAGA	412
				GGGGATGTAGCTCAGATGG	355
				GGGGATGTAGCTCAGATGGTA	328
				GGGGATGTAGCTCAGATGGTAGAG	263
				GGGGATGTAGCTCAGATGGTAG	202
				GGGGATGTAGCTCAGATGGTAGAGCT	17
				GGGGATGTAGCTCAGATGGTAGAGCGCT	11
				GGGGATGTAGCTCAGATGGTAGAGCCT	10
			5'AlaAGC tRF2 Variants	GGGGATGTAGCTCAAATGGT	453
				GGGGATGTAGCTCAAATGGTAGAGC	293
				GGGGATGTAGCTCAAATGGTAGA	241
				GGGGATGTAGCTCAAATGGTA	238
				GGGGATGTAGCTCAAATGGTAGAG	209
				GGGGATGTAGCTCAAATGG	178
				GGGGATGTAGCTCAAATGGTAG	139
				GGGGATGTAGCTCAAATGGTAGAGCT	16
			5'AlaCGC tRF1 Variants	GGGGGTGTAGCTCATATGGT	117
				GGGGGTGTAGCTCATATGG	70
				GGGGGTGTAGCTCATATGGTA	56
				GGGGGTGTAGCTCATATGGTAG	28
				GGGGGTGTAGCTCATATGGTAGA	63
				GGGGGTGTAGCTCATATGGTAGAG	43
				GGGGGTGTAGCTCATATGGTAGAGC	76
			5'AlaCGC tRF2 Variants	GGGGATGTAGCTCATATGGT	107
				GGGGATGTAGCTCATATGGTAGAGC	57
				GGGGATGTAGCTCATATGGTA	52
				GGGGATGTAGCTCATATGGTAGA	51
				GGGGATGTAGCTCATATGG	44
				GGGGATGTAGCTCATATGGTAGAG	38
				GGGGATGTAGCTCATATGGTAG	22
			5'AlaTGC tRF1 Variants	GGGGATGTAGCTCAAATGGT	453
				GGGGATGTAGCTCAAATGGTAGAGC	293
				GGGGATGTAGCTCAAATGGTAGA	241
				GGGGATGTAGCTCAAATGGTA	238
				GGGGATGTAGCTCAAATGGTAGAG	209
				GGGGATGTAGCTCAAATGG	178
				GGGGATGTAGCTCAAATGGTAG	139
				GGGGATGTAGCTCAAATGGTAGAGCT	16

Plant sp	Small RNA library	Tissue	5'Ala tRFs	Sequence	Read count
Zea mays	GSM433620	Leaf	5'AlaAGC tRF1 Variants	GGGGATGTAGCTCAGATGGT	29590
				GGGGATGTAGCTCAGATGGTA	25274
				GGGGATGTAGCTCAGATGG	15919
				GGGGATGTAGCTCAGATGGTAGA	7519
				GGGGATGTAGCTCAGATGGTAG	7268
				GGGGATGTAGCTCAGATGGTAGAG	5367
				GGGGATGTAGCTCAGATGGTAA	1064
				GGGGATGTAGCTCAGATGGTAGAGC	1030
				GGGGATGTAGCTCAGATGGTAGAA	699
				GGGGATGTAGCTCAGATGGTAAA	465
			5'AlaAGC tRF1 Variants	GGGGATGTAGTTCAGATGGTA	10145
				GGGGATGTAGTTCAGATGGTAGAA	3139
				GGGGATGTAGTTCAGATGGTAGA	3121
				GGGGATGTAGTTCAGATGGTAG	2337
				GGGGATGTAGTTCAGATGGTAGAACA	200
				GGGGATGTAGTTCAGATGGTAGAACAA	191
				GGGGATGTAGTTCAGATGGTAGAAC	160
				GGGGATGTAGTTCAGATGGTAA	148
				GGGGATGTAGTTCAGATGGTAGAAA	128
			5'AlaCGC tRF1 Variants	GGGGGTGTAGCTCATATGGTA	4521
				GGGGGTGTAGCTCATATGGT	2288
				GGGGGTGTAGCTCATATGGTAGA	1441
				GGGGGTGTAGCTCATATGGTAGAG	869
				GGGGGTGTAGCTCATATGGTAG	606
				GGGGGTGTAGCTCATATGG	190
				GGGGGTGTAGCTCATATGGTAGAA	147
				GGGGGTGTAGCTCATATGGTAGAGC	109
				GGGGGTGTAGCTCATATGGTAGAAA	69
				GGGGGTGTAGCTCATATGGTAA	45
			5'AlaCGC tRF2 Variants	GGGGACGTAGCTCATATGGTA	2238
				GGGGACGTAGCTCATATGGT	1537
				GGGGACGTAGCTCATATGGTAGA	897
				GGGGACGTAGCTCATATGGTAGAG	480
				GGGGACGTAGCTCATATGGTAG	298
				GGGGACGTAGCTCATATGG	270
				GGGGACGTAGCTCATATGGTAGAA	96
				GGGGACGTAGCTCATATGGTAGAGC	72
				GGGGACGTAGCTCATATG	56
		Leaf	5'AlaTGC tRF1 Variants	GGGGATGTAGCTCAAATGGTA	16784
				GGGGATGTAGCTCAAATGGT	9664
				GGGGATGTAGCTCAAATGGTAGA	5206
				GGGGATGTAGCTCAAATGGTAG	4896
				GGGGATGTAGCTCAAATGGTAGAG	3463
				GGGGATGTAGCTCAAATGG	753
				GGGGATGTAGCTCAAATGGTAGAA	513
				GGGGGTGTAGCTCAAATGGTA	429
				GGGGATGTAGCTCAAATGGTAA	338
				GGGGATGTAGCTCAAATGGTAGAGC	306
			5'AlaTGC tRF2 Variants	GGGGGTGTAGCTCATATGGTA	4521
				GGGGGTGTAGCTCATATGGT	2288
				GGGGGTGTAGCTCATATGGTAGA	1441
				GGGGGTGTAGCTCATATGGTAGAG	869
				GGGGGTGTAGCTCATATGGTAG	606
				GGGGGTGTAGCTCATATGG	190
				GGGGGTGTAGCTCATATGGTAGAA	147
				GGGGGTGTAGCTCATATGGTAGAGC	109
			5'AlaTGC tRF3 Variants	GGGGACGTAGCTCAAATGGTA	1271
				GGGGACGTAGCTCAAATGGT	767
				GGGGACGTAGCTCAAATGGTAGA	390
				GGGGACGTAGCTCAAATGGTAG	309
				GGGGACGTAGCTCAAATGGTAGAG	228
				GGGGACGTAGCTCAAATGG	91
				GGGGACGTAGCTCAAATGGTAGAGC	41
				GGGGACGTAGCTCAAATGGTAGAA	37
				GGGGACGTAGCTCAAATGGTAA	26
				GGGGACGTAGCTCAAATGGTAAA	19

Plant sp	Small RNA library	Tissue	5'Ala tRFs	Sequence	Read count
Medicago truncatula	GSM769277	leaf	5'AlaAGC tRF1 Variants	GGGGATGTAGCTCAGATGGT	1526
				GGGGATGTAGCTCAGATGGTA	880
				GGGGATGTAGCTCAGATGG	786
				GGGGATGTAGCTCAGATGGTAGA	592
				GGGGATGTAGCTCAGATGGTAG	522
				GGGGATGTAGCTCAGATGGTAGAA	16
				GGGGATGTAGCTCAGATGGTAGAAA	12
			5'AlaCGC tRF1 Variants	GGGGACGTAGCTCATATGGTA	11
				GGGGACGTAGCTCATATGGT	7
				GGGGACGTAGCTCATATGGTAGA	7
				GGGGACGTAGCTCATATGGTAG	4
				GGGGACGTAGCTCATATGG	2
				GGGGACGTAGCTCATATGGTAGAG	2
				GGGGATGTAGCTCATATGGTA	2
			5'AlaCGC tRF2 Variants	GGGGTTGTAGCTCATATGGTA	75
				GGGGTTGTAGCTCATATGGTAG	34
				GGGGTTGTAGCTCATATGGT	32
				GGGGTTGTAGCTCATATGGTAGA	27
				GGGGTTGTAGCTCATATGGTAGAG	8
				GGGGTTGTAGCTCATATGG	7
				GGGGTTGTAGCTCATATGGGA	2
				GGGGTTGTAGCTCATATGGTAGCG	2
			5'AlaCGC tRF3 Variants	GGGGGTGTAGCTCATATGGTA	43
				GGGGGTGTAGCTCATATGGT	27
				GGGGGTGTAGCTCATATGGTAGA	26
				GGGGGTGTAGCTCATATGGTAG	17
			5'AlaTGC tRF1 Variants	GGGGATGTAGCTCAAATGGTA	285
				GGGGATGTAGCTCAAATGGTAGA	270
				GGGGATGTAGCTCAAATGGTAG	241
				GGGGATGTAGCTCAAATGGTAGAG	156
				GGGGATGTAGCTCAAATGGT	145
				GGGGATGTAGCTCAAATGGTAGAGC	69
				GGGGATGTAGCTCAAATGG	18
				GGGGATGTAGCTCAAATGGTAGAA	13
				GGGGATGTAGCTCAAATGGTAGCG	12
			5'AlaTGC tRF2 Variants	GGGGATGTAGCTCACATGGTAGA	98
				GGGGATGTAGCTCACATGGTA	94
				GGGGATGTAGCTCACATGGTAG	92
				GGGGATGTAGCTCACATGGTAGAG	66
				GGGGATGTAGCTCACATGGT	33
				GGGGATGTAGCTCACATGGTAGAGC	23
				GGGGATGTAGCTCACATGG	12
				GGGGATGTAGCTCACATGGTAGC	11

Plant sp	nall RNA libra	Tissue	5'Ala tRFs	Sequence	Read count
Oryza sativa	GSM361264	Leaf	5'AlaAGC tRF1 Variants	GGGGATGTAGCTCAGATGGT	5924
				GGGGATGTAGCTCAGATGGTAGA	3077
				GGGGATGTAGCTCAGATGG	2743
				GGGGATGTAGCTCAGATGGTA	2263
				GGGGATGTAGCTCAGATGGTAGAG	1413
				GGGGATGTAGCTCAGATGGTAG	1315
				GGGGATGTAGCTCAGATGGTAGAGC	505
				GGGGATGTAGCTCAGATGGA	188
				GGGGATGTAGCTCAGATGGTT	73
				GGGGATGTAGCTCAGATGGTAGAGCT	64
			AlaAGC tRF2 Variar	GGGGGTGTAGCTCAGATGG	69
				GGGGGTGTAGCTCAGATGGT	289
				GGGGGTGTAGCTCAGATGGTA	129
				GGGGGTGTAGCTCAGATGGTAG	76
				GGGGGTGTAGCTCAGATGGTAGA	181
				GGGGGTGTAGCTCAGATGGTAGAG	66
				GGGGGTGTAGCTCAGATGGTAGAGC	33
			5'AlaCGC tRF1Variants	GGGGGTGTAGCTCATATGGTAGA	370
				GGGGGTGTAGCTCATATGGTAGAG	244
				GGGGGTGTAGCTCATATGGTAGAGC	98
				GGGGGTGTAGCTCATATGGTA	97
				GGGGGTGTAGCTCATATGGTAG	49
				GGGGGTGTAGCTCATATGGT	35
				GGGGGTGTAGCTCATATGG	21
				GGGGGTGTAGCTCATATGGTAGAA	19
				GGGGGTGTAGCTCATATGGTAGAGAA	12
				GGGGGTGTAGCTCATATGGTAGAAA	10
			5'AlaCGC tRF2 Variants	GGGGACGTAGCTCATATGGTAGA	236
				GGGGACGTAGCTCATATGGTAGAG	133
				GGGGACGTAGCTCATATGGTAGAGC	86
				GGGGACGTAGCTCATATGGTA	49
				GGGGACGTAGCTCATATGGTAG	38
				GGGGACGTAGCTCATATGGT	31
				GGGGACGTAGCTCATATGG	26
				GGGGACGTAGCTCATATGGTAGAAA	14
				GGGGACGTAGCTCATATGGTAGAA	13
			5'AlaTGC tRF1 Variants	GGGGATGTAGCTCAAATGGTAGAG	504
				GGGGATGTAGCTCAAATGGTAGA	388
				GGGGATGTAGCTCAAATGGTAG	165
				GGGGATGTAGCTCAAATGGTAGAGC	161
				GGGGATGTAGCTCAAATGGTA	117
				GGGGATGTAGCTCAAATGGT	61
				GGGGATGTAGCTCAAATGG	32
				GGGGATGTAGCTCAAATGGTAGAA	16
				GGGATGTAGCTCAAATGGTAGA	14
				GGGGATGTAGCTCAAATGGTAGAAA	13

Plant sp	Small RNA library	Tissue	5'Ala tRFs	Sequence	Read count
Populus trichocarpa	GSM717875	Leaf	5'AlaAGC tRF1 Variants	GGGGATGTAGCTCAGATGGTA	44611
				GGGGATGTAGCTCAGATGGT	44438
				GGGGATGTAGCTCAGATGGTAG	16606
				GGGGATGTAGCTCAGATGGTAGA	15697
				GGGGATGTAGCTCAGATGG	13698
				GGGGATGTAGCTCAGATGGTAGAG	11806
				GGGGATGTAGCTCAGATGGTAGAGC	2760
			5'AlaCGC tRF1 Variants	GGGGATGTAGCTCATATGGTA	1271
				GGGGATGTAGCTCATATGGTAGA	769
				GGGGATGTAGCTCATATGGTAGAG	448
				GGGGATGTAGCTCATATGGT	417
				GGGGATGTAGCTCATATGGTAG	313
				GGGGATGTAGCTCATATGGTAGAA	92
				GGGGATGTAGCTCATATGGTAGAGC	71
				GGGGATGTAGCTCATATGG	52
				GGGGATGTAGCTCATATGGTAGAAA	21
				GGGGATGTAGCTCATATGGTAGAGAA	20
			5'AlaCGC tRF2 Variants	GGGGTTGTAGCTCATATGGTA	2379
				GGGGTTGTAGCTCATATGGTAGA	1291
				GGGGTTGTAGCTCATATGGTAGAG	981
				GGGGTTGTAGCTCATATGGTAG	688
				GGGGTTGTAGCTCATATGGT	604
				GGGGTTGTAGCTCATATGGTAGAGC	126
				GGGGTTGTAGCTCATATGGTAGAA	108
				GGGGTTGTAGCTCATATGGTAGAAA	54
				GGGGTTGTAGCTCATATGG	48
			5'AlaCGC tRF3 Variants	GGGGGTGTAGCTCATATGGTA	642
				GGGGGTGTAGCTCATATGGTAGA	366
				GGGGGTGTAGCTCATATGGTAGAG	201
				GGGGGTGTAGCTCATATGGTAG	173
				GGGGGTGTAGCTCATATGGT	169
				GGGGGTGTAGCTCATATGGTAGAA	44
				GGGGGTGTAGCTCATATGG	26
				GGGGGTGTAGCTCATATGGTAGAGC	26
				GGGGGTGTAGCTCATATGGTAGAAA	14
			5'AlaTGC tRF1 Variants	GGGGATGTAGCTCAAATGGTA	19138
				GGGGATGTAGCTCAAATGGTAGA	10173
				GGGGATGTAGCTCAAATGGTAGAG	7620
				GGGGATGTAGCTCAAATGGTAG	7063
				GGGGATGTAGCTCAAATGGT	5981
				GGGGATGTAGCTCAAATGGTAGAA	991
				GGGGATGTAGCTCAAATGGTAGAGC	946
				GGGGATGTAGCTCAAATGG	637
				GGGGATGTAGCTCAAATGGTAGAAA	557
				GGGGATGTAGCTCAAATGGTAGAGAA	185
			5'AlaTGC tRF2 Variants	GGGGATGTAGCACAAATGGTA	30
				GGGGATGTAGCACAAATGGT	10

Plant sp	Small RNA library	Tissue	5'Ala tRFs	Sequence	Read count
Solanum tuberosum	GSM803582	leaf	5'AlaAGC tRF1 Variants	GGGGATGTAGCTCAGATGGTAGA	6978
				GGGGATGTAGCTCAGATGGT	6716
				GGGGATGTAGCTCAGATGGTA	4669
				GGGGATGTAGCTCAGATGGTAG	4197
				GGGGATGTAGCTCAGATGGTAGAG	3218
				GGGGATGTAGCTCAGATGG	2236
				GGGGATGTAGCTCAGATGGTAGAGC	490
				GGGGATGTAGCTCAGATGGTAGAAA	205
				GGGGATGTAGCTCAGATGGTAGAA	138
			5'AlaCGC tRF1 Variants	GGGGGTGTAGCTCATATGGTAGA	866
				GGGGGTGTAGCTCATATGGTAGAG	499
				GGGGGTGTAGCTCATATGGTA	310
				GGGGGTGTAGCTCATATGGTAG	265
				GGGGGTGTAGCTCATATGGT	106
				GGGGGTGTAGCTCATATGGTAGAGC	57
				GGGGGTGTAGCTCATATGG	39
				GGGGGTGTAGCTCATATGGTAGAA	37
				GGGGGTGTAGCTCATATGGTAGAAA	35
			5'AlaCGC tRF2 Variants	GGGGATGTAGCTCATATGGT	19
				GGGGATGTAGCTCATATGGTA	14
				GGGGATGTAGCTCATATGGTAG	17
				GGGGATGTAGCTCATATGGTAGA	18
			5'AlaTGC tRF1 Variants	GGGGATGTAGCTCAAATGGTAGA	3374
				GGGGATGTAGCTCAAATGGTAGAG	2170
				GGGGATGTAGCTCAAATGGTAG	1704
				GGGGATGTAGCTCAAATGGTA	1366
				GGGGATGTAGCTCAAATGGT	563
				GGGGATGTAGCTCAAATGGTAGAGC	318
				GGGGATGTAGCTCAAATGG	130
				GGGGATGTAGCTCAAATGGTAGAAA	129
				GGGGATGTAGCTCAAATGGTAGAA	114
				GGGGATGTAGCTCAAATGGTAGTCGTAT[illegible]	40
			5'AlaTGC tRF1 Variants	GGGGGTGTAGCTCATATGGTAGA	866
				GGGGGTGTAGCTCATATGGTAGAG	499
				GGGGGTGTAGCTCATATGGTA	310
				GGGGGTGTAGCTCATATGGTAG	265
				GGGGGTGTAGCTCATATGGT	106
				GGGGGTGTAGCTCATATGGTAGAGC	57
				GGGGGTGTAGCTCATATGG	39
				GGGGGTGTAGCTCATATGGTAGAA	37

Plant sp	small RNA library	Tissue	5'Ala tRFs	Sequence	Read count
Sorghum bicolor	GSM803128	leaf	5'AlaAGC tRF1 Variants	GGGGATGTAGCTCAGATGGTA	103696
				GGGGATGTAGCTCAGATGGT	97919
				GGGGATGTAGCTCAGATGGTAG	46108
				GGGGATGTAGCTCAGATGGTAGA	39535
				GGGGATGTAGCTCAGATGG	24608
				GGGGATGTAGCTCAGATGGTAGAG	23082
				GGGGATGTAGCTCAGATGGTAGAGC	6489
				GGGGATGTAGCTCAGATGGTAGAA	1513
				GGGGATGTAGCTCAGATGGTAA	1166
				GGGGATGTAGCTCAGATGGTAGAAA	1130
			5'AlaAGC tRF2 Variants	GGGGATGTAGCTCAAATGGTA	27164
				GGGGATGTAGCTCAAATGGTAGA	20008
				GGGGATGTAGCTCAAATGGTAG	16137
				GGGGATGTAGCTCAAATGGTAGAG	13742
				GGGGATGTAGCTCAAATGGT	9211
				GGGGGTGTAGCTCAAATGGTA	2690
				GGGGATGTAGCTCAAATGGTAGAGC	2088
				GGGGGTGTAGCTCAAATGGTAGA	1474
				GGGGGTGTAGCTCAAATGGTAG	1433
				GGGGATGTAGCTCAAATGGTAGAA	1252
				GGGGATGTAGCTCAAATGG	1182
				GGGGGTGTAGCTCAAATGGTAGAG	1120
			5'AlaCGC tRF1 Variants	GGGGGTGTAGCTCATATGGTA	8330
				GGGGGTGTAGCTCATATGGTAGA	5485
				GGGGGTGTAGCTCATATGGTAG	3870
				GGGGGTGTAGCTCATATGGTAGAG	3594
				GGGGGTGTAGCTCATATGGT	2152
				GGGGGTGTAGCTCATATGGTAGAGC	500
				GGGGGTGTAGCTCATATGGTAGAA	436
				GGGGGTGTAGCTCATATGG	360
				GGGGGTGTAGCTCATATTGTA	197
			5'AlaCGC tRF1 Variants	GGGGGTGTAGCTCATATGGTAGAAA	189
				GGGGGTGTAGCTCATATTGTAGA	113
				GGGGACGTAGCTCATATGGTA	6424
				GGGGACGTAGCTCATATGGTAGA	5365
				GGGGACGTAGCTCATATGGTAGAG	3507
				GGGGACGTAGCTCATATGGTAG	2578
				GGGGACGTAGCTCATATGGT	2183
				GGGGACGTAGCTCATATGGTAGAGC	878
				GGGGATGTAGCTCATATGGTA	564
				GGGGATGTAGCTCATATGGT	447
				GGGGACGTAGCTCATATGGTAGAA	418
			5'AlaTGC tRF1 Variants	GGGGATGTAGCTCAAATGGTA	27164
				GGGGATGTAGCTCAAATGGTAGA	20008
				GGGGATGTAGCTCAAATGGTAG	16137
				GGGGATGTAGCTCAAATGGTAGAG	13742
				GGGGATGTAGCTCAAATGGT	9211
				GGGGGTGTAGCTCAAATGGTA	2690
				GGGGATGTAGCTCAAATGGTAGAGC	2088
				GGGGGTGTAGCTCAAATGGTAGA	1474
				GGGGGTGTAGCTCAAATGGTAG	1433
				GGGGATGTAGCTCAAATGGTAGAA	1252
