# Supplementary material for: Transfer RNA Derived Small RNAs Targeting Defense Responsive Genes Are Induced during Phytophthora capsici Infection in Black Pepper (Piper nigrum L.)
Source: Front Plant Sci. 2016 Jun 1;7:767. doi: 10.3389/fpls.2016.00767 (PMC4887504; doi:10.3389/fpls.2016.00767)
Supplement: Supplementary file 6 [file Image1.PDF]

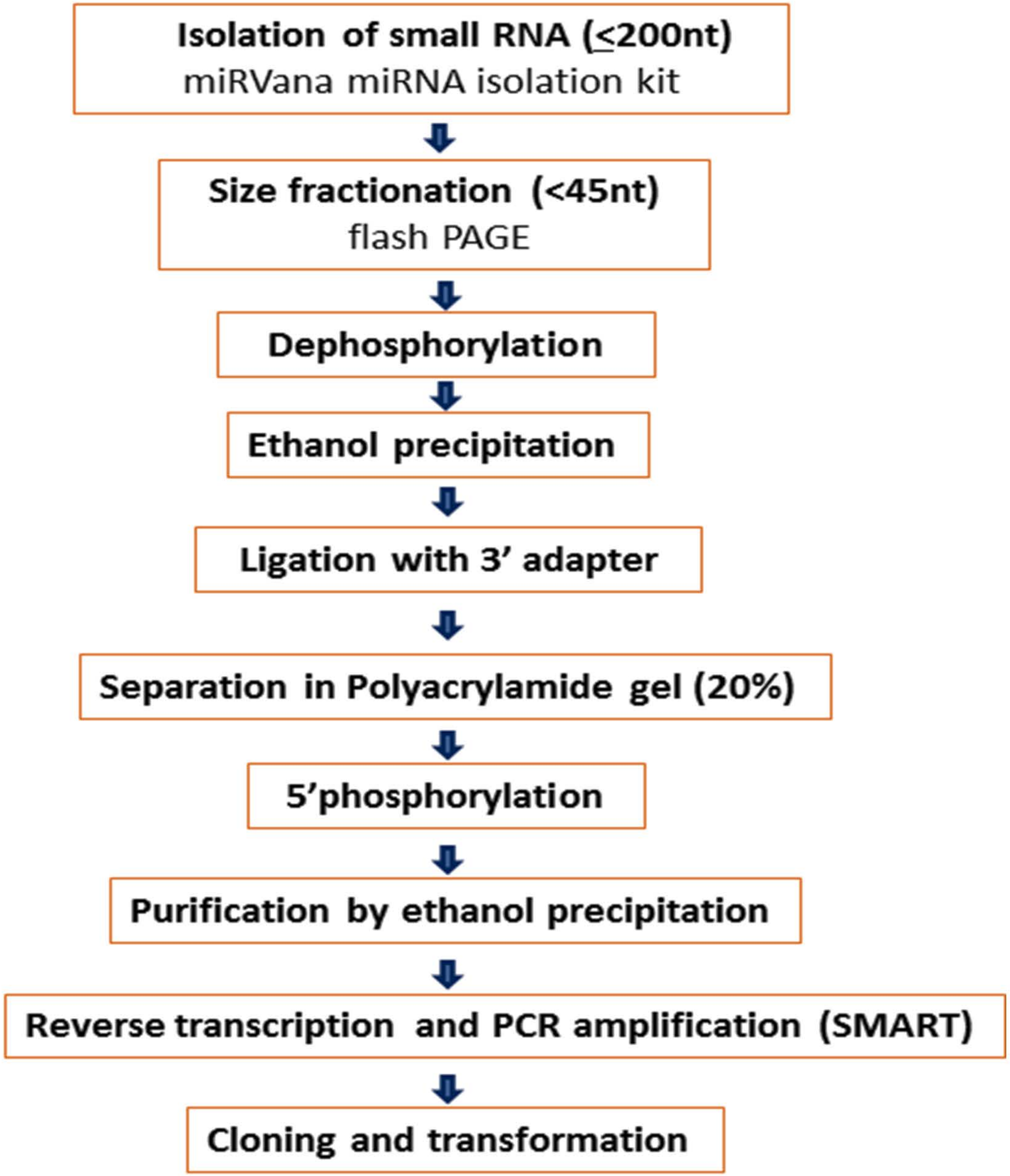

Figure S1: The overall work flow of the small RNA cloning from pathogen stressed black pepper plants.

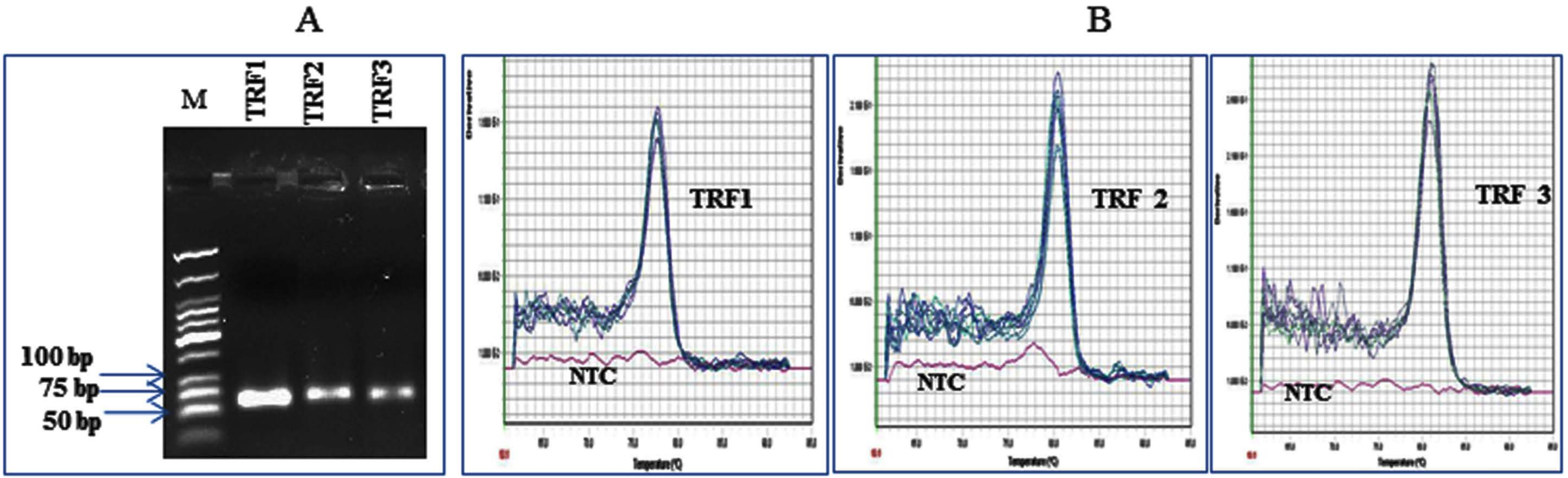

Figure S2: Validation of black pepper small RNAs: 5′Ala^{CGC} tRF (TRF1) and 5′Arg^{TCG} tRF (TRF2) and 3′ Gly^{TCC} tRF (TRF3) by stem-loop reverse transcription polymerase chain reaction (RT-PCR). The sizes of the PCR products were approximately 60bp. M indicates low molecular weight DNA Ladder (NEB). (B) Dissociation curve of real-time PCR amplification of primers of TRF1, TRF2 and TRF3.

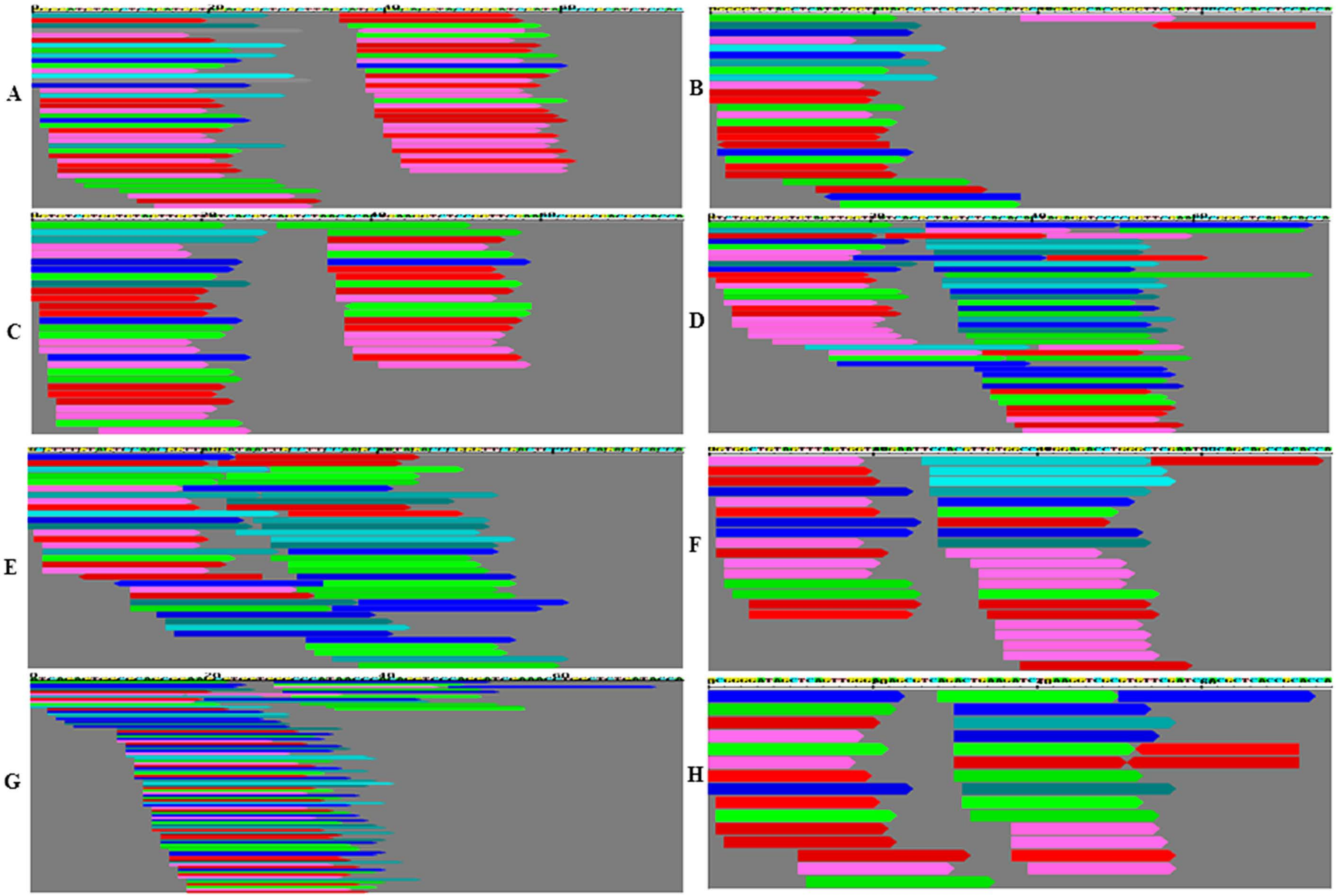

Figure S3: The mapping of tRFs in the mature tRNAs of (A) Ala tRNA^{AGC} (B) Ala tRNA^{CGC} (C) Val tRNA^{AAC} (D) Val tRNA^{CAC} (E) Gly tRNA^{TCC} (F) His tRNA^{GTG} (G) Met tRNA^{CAT} and (H) Phe tRNA^{GAA}

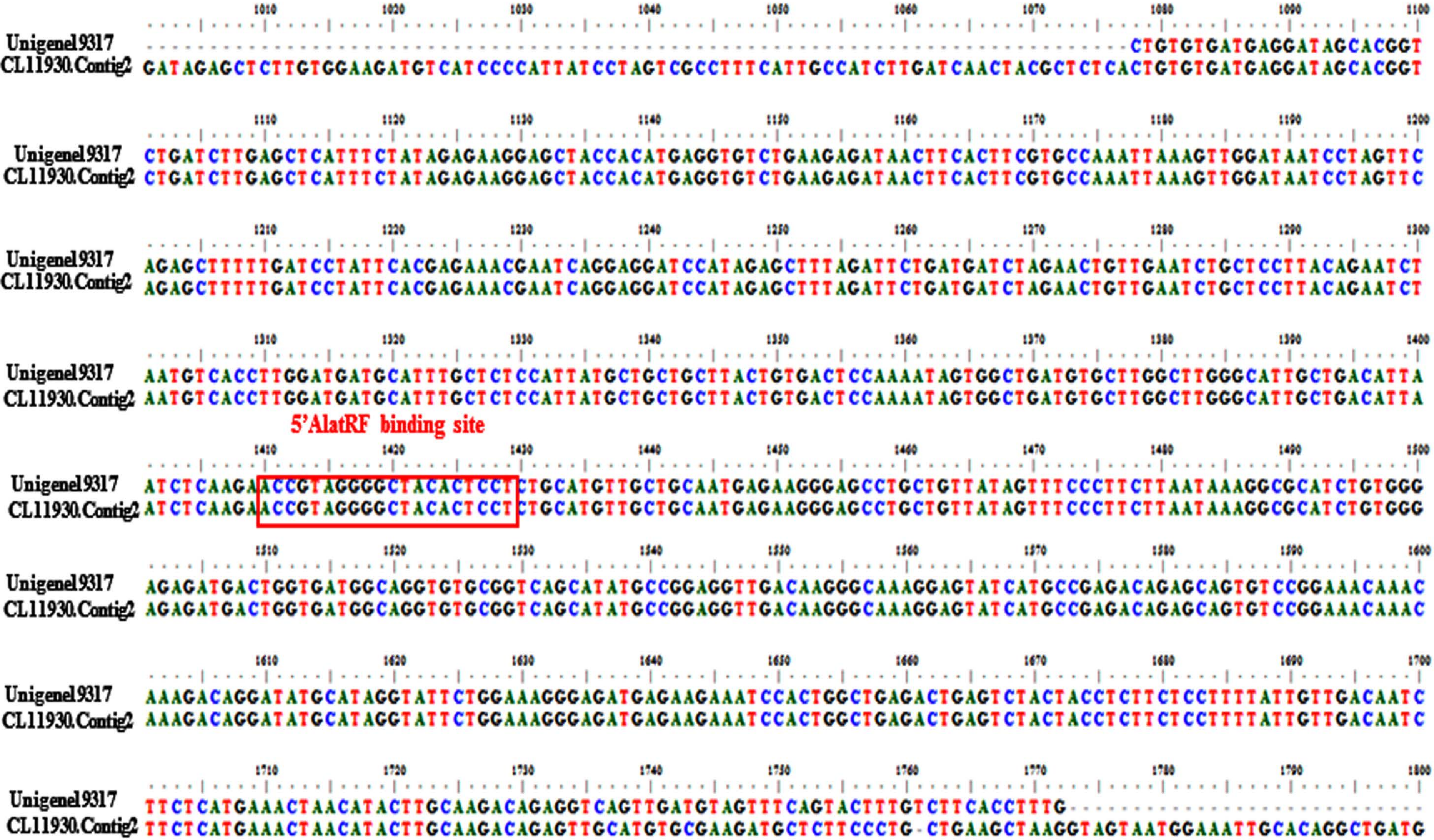

Figure S4: Sequence alignment of NPR1 variants (CL11930Contig 2, Unigene 19317) identified as targets of 5′Ala tRFs from black pepper. Both the homologs possessed sequence conservation at the tRF binding site.
